# The development and validation of an easy to use automatic QT-interval algorithm

**DOI:** 10.1371/journal.pone.0184352

**Published:** 2017-09-01

**Authors:** Ben J. M. Hermans, Arja S. Vink, Frank C. Bennis, Luc H. Filippini, Veronique M. F. Meijborg, Arthur A. M. Wilde, Laurent Pison, Pieter G. Postema, Tammo Delhaas

**Affiliations:** 1 Department of Biomedical Engineering, Maastricht University, Maastricht, The Netherlands; 2 Cardiovascular Research Institute Maastricht (CARIM), Maastricht University, Maastricht, The Netherlands; 3 Department of Cardiology, Maastricht University Medical Centre, Maastricht, The Netherlands; 4 Heart Centre, Department of Clinical and Experimental Cardiology, Academic Medical Center, Amsterdam, the Netherlands; 5 MHeNS School for Mental Health and Neuroscience, Maastricht University, Maastricht, The Netherlands; 6 Juliana Children’s Hospital, The Hague, The Netherlands; 7 Netherlands Heart Institute, Holland Heart House, Utrecht, The Netherlands; University of Adelaide, AUSTRALIA

## Abstract

**Background:**

To evaluate QT-interval dynamics in patients and in drug safety analysis, beat-to-beat QT-interval measurements are increasingly used. However, interobserver differences, aberrant T-wave morphologies and changes in heart axis might hamper accurate QT-interval measurements.

**Objective:**

To develop and validate a QT-interval algorithm robust to heart axis orientation and T-wave morphology that can be applied on a beat-to-beat basis.

**Methods:**

Additionally to standard ECG leads, the root mean square (ECG_RMS_), standard deviation and vectorcardiogram were used. QRS-onset was defined from the ECG_RMS_. T-wave end was defined per individual lead and scalar ECG using an automated tangent method. A median of all T-wave ends was used as the general T-wave end per beat.

Supine-standing tests of 73 patients with Long-QT syndrome (LQTS) and 54 controls were used because they have wide ranges of RR and QT-intervals as well as changes in T-wave morphology and heart axis orientation. For each subject, automatically estimated QT-intervals in three random complexes chosen from the low, middle and high RR range, were compared with manually measured QT-intervals by three observers.

**Results:**

After visual inspection of the randomly selected complexes, 21 complexes were excluded because of evident noise, too flat T-waves or premature ventricular beats. Bland-Altman analyses of automatically and manually determined QT-intervals showed a bias of <4ms and limits of agreement of ±25ms. Intra-class coefficient indicated excellent agreement (>0.9) between the algorithm and all observers individually as well as between the algorithm and the mean QT-interval of the observers.

**Conclusion:**

Our automated algorithm provides reliable beat-to-beat QT-interval assessment, robust to heart axis and T-wave morphology.

## Introduction

Prolongation of the QT-interval on the electrocardiogram (ECG) has been associated with *Torsade de Pointes*, a potentially lethal cardiac arrhythmia.[1,2] A prolonged QT-interval can be caused by Long-QT syndrome (LQTS), which can be either inherited or acquired due to an underlying medical condition or medication.[2] The measurement of the QT-interval is used world-wide on a daily basis in the diagnosis of LQTS or in the evaluation of possible effects of a new drug on the QT-interval.[3]

Although the value of a prolonged QT-interval for risk assessment of future malignant arrhythmias is widely understood [1], most physicians, including cardiologists, have difficulties to correctly identify a prolonged QT-interval.[4] Additionally to measurement difficulties, diagnosing LQTS is challenging since there is a considerable overlap of the QT-interval between LQTS patients and healthy controls.[5,6] Because of this overlap in QT-intervals, additional measurements like QT dispersion [7,8] and QT variability [9] were introduced and assessed on their value to diagnose LQTS. Because these relatively new parameters are used to study QT dynamics, they require evaluation of large numbers of RR- and QT-intervals. QT variability, for example, is typically determined from 256–512 beats or 256–512 seconds duration ECG.[9] Furthermore, supine-standing tests are introduced to study QT-interval adaptation [10,11] and changes in T-wave morphologies [12] due to heart rate changes induced by brisk standing. In these tests, QT-interval dynamics are assessed based on a small number of QT-intervals. [10,11] Beat-to-beat analysis of supine-standing tests might give more insight in the dynamic behaviour of the QT-interval and therefore improve its diagnostic value. Measuring these large numbers of RR- and QT-intervals manually is very time consuming and therefore automated QT-interval algorithms are necessary.

Currently, automatic algorithms for measuring the QT-interval embedded in commercial ECG systems measure the QT-interval on an average or median complex over time (cf. Appendix Kligfield et al.[13]). As a consequence, beat-to-beat detection algorithms which include the QT-interval dynamicity have been published, but often use only a single ECG lead (mostly II or V5), which makes the QT-interval susceptible to heart axis orientation and electrode placement.[14] In this article we present and validate an automatic QT-interval algorithm based on the tangent method [15] which is unaffected by heart axis orientation and that can be applied on a beat-to-beat basis regardless of the T-wave morphology.

## Methods

### Population and ECG recordings

Five minutes long ECGs from supine-standing tests recorded between December 2008 and February 2016 of 73 LQTS patients and 54 controls were included in this study. These recordings were performed in the initial evaluation of individuals referred to the department of Cardiology and Cardiogenetics of the Academic Medical Centre in Amsterdam, The Netherlands, in the work-up during family screening for LQTS (i.e. after a diagnosis was made in an index patient). LQTS patients had a confirmed pathogenic mutation in either the KCNQ1, KCNH2 or SCN5A gene resulting in LQTS type 1 (LQT1), type 2 (LQT2) or type 3 (LQT3) respectively. Controls were genotype-negative family members or healthy volunteers. We obtained a waiver from the local ethical committee for ethical approval for the conduct of this study.

ECG recordings during supine-standing tests were used to validate the algorithm since these recordings consist of a wide range of RR and QT-intervals as well as changes in T-wave morphology and heart axis orientation. [10–12]

### Development of an automatic QT-interval detection algorithm

#### Data acquisition and pre-processing

**Fig 1:**
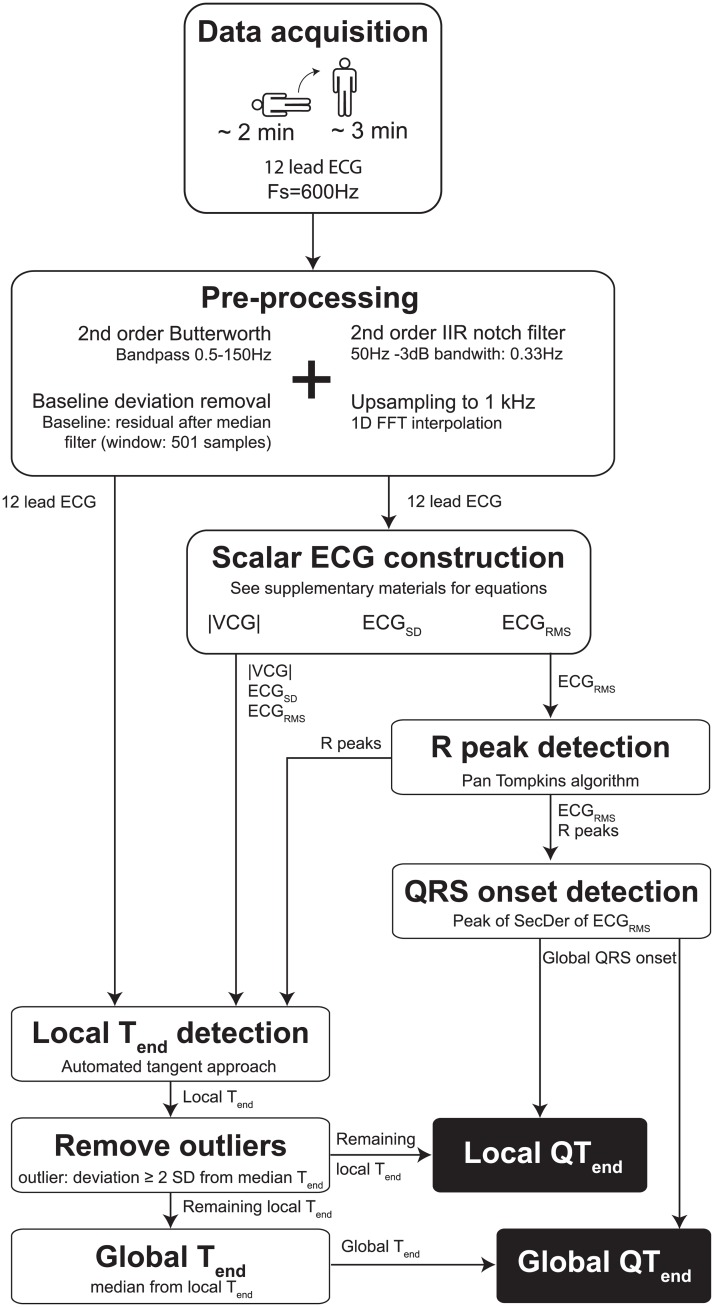
Schematic representation the algorithm’s steps. A detailed description is given in the main text. Fs = sample frequency, Hz = hertz, IIR = infinite impulse response, FFT = fast Fourier transform, |VCG| = magnitude of the vectorcardiogram, SD = standard deviation, RMS = root mean square, SecDer = second derivative, T_end_ = T wave end.

All individual (pre-)processing steps of the algorithm are shown in Fig 1. All ECGs were recorded with a 600Hz sample frequency using Welch Allyn CardioPerfect (Welch Allyn, Skaneateles Falls, NY, USA). Data analysis was performed offline using a custom-made Matlab (2015b, The MathWorks, Natick, MA, USA) program. After acquisition, ECG data were filtered using a 2^nd^ order bidirectional Butterworth band pass filter (0.5-100Hz [16]) and a 2^nd^ order infinite impulse response notch filter (50Hz) with a -3dB bandwidth of 0.33Hz. For all individual leads, the residuals of a median filter with a 501 samples window were regarded as baseline deviations and were subtracted from the individual ECG leads to correct for baseline wander. The filtered ECGs were thereafter upsampled to 1000Hz to make the analysis sample frequency independent so it can be applied to ECGs recorded with different sample frequencies as well.

#### Scalar ECG construction

Three types of scalar ECGs are constructed to emulate ECG signals unaffected by heart axis orientation. The root mean square (ECG_RMS_) and standard deviation (ECG_SD_) are calculated as follows:

$${\text{ECG}}_{\text{RMS}}\left(t\right)=\sqrt{\frac{1}{9}\sum_{i=1}^{9}\left({\text{ ECG}}_{i}^{2}\left(t\right)\;\right)}$$ (1)

$${\text{ECG}}_{\text{SD}}\left(t\right)=\sqrt{\frac{1}{9}\sum_{i=1}^{9}{\left({\text{ ECG}}_{i}\left(t\right)-\bar{\text{ECG}}\left(t\right)\;\right)}^{2}}$$ (2)

where ECG_*i*_(*t*) is the ECG signal at time *t* from lead *i* and $\bar{\text{ECG}}\left(t\right)$ is the mean ECG in time over the various leads. Note that there are only nine leads used in this calculation. Ideally, one would use unipolar precordial leads *and* unipolar limb leads to reconstruct a scalar ECG from. Unfortunately, true unipolar limb leads are not recorded (or saved) in a standard 12-lead ECG. Mathematically, augmented limb leads are scaled true unipolar ECG leads. For example, the unipolar foot electrode (VF) would be calculated by:

$$VF=\phi_{f}-\phi_{WCT}=\phi_{f}-\frac{\phi_{f}+\phi_{r}+\phi_{l}}{3}=\frac{2\phi_{f}-\phi_{r}-\phi_{l}}{3}$$ (3)

with *ϕ*_*f*_, *ϕ*_*r*_ and *ϕ*_*l*_ the potential recorded at the foot, right arm and left arm respectively and *ϕ*_*WCT*_ the Wilson central terminal.

The augmented limb lead aVF is calculated by:

$$aVF=\phi_{f}-\frac{\phi_{r}+\phi_{l}}{2}=\frac{2\phi_{f}-\phi_{r}-\phi_{l}}{2}$$ (4)

So, VF can be calculated from aVF by scaling aVF with 2/3.

$$\frac{2}{3}\cdot \frac{\left(2\phi_{f}-\phi_{r}-\phi_{l}\right)}{2}=\frac{2\phi_{f}-\phi_{r}-\phi_{l}}{3}$$ (5)

We used these calculated unipolar limb leads (2/3 aVR, 2/3 aVL, 2/3 aVF) and the unipolar precordial leads (V1-V6) to construct the ECG_RMS_ and ECG_SD_.

Lastly, a vectorcardiogram (VCG) was constructed using the method described by Kors et al.[17] The magnitude of this VCG (|VCG|) was used as the third scalar ECG.

#### R peak and QRS onset detection

**Fig 2:**
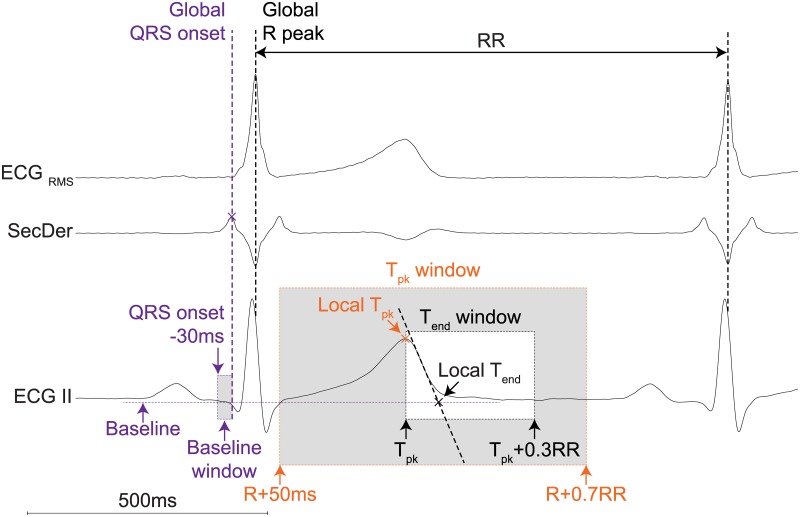
Illustration of global QRS onset and local T wave landmarks detection. Global R peak is detected using a Pan-Tompkins algorithm on the ECG_RMS_ signal. The global QRS onset is thereafter detected as a peak in the second derivative of the ECG_RMS_ within a certain window preceding the global R peak. The local T peak (T_pk_) is detected as the maximum or minimal peak between R+50ms and R+0.7RR. Thereafter, the tangent trough the point of maximum deflection between T_pk_ and T_pk_+0.3RR is calculated from the first derivative. The intersection between this tangent and the baseline is detected as the local end of the T wave (T_end_). Tpk = T-wave peak, Tend = T-wave end, RMS = root mean square, SecDer = second derivative, ms = milliseconds.

R peaks were detected from the ECG_RMS_ signal using the Pan Tompkins algorithm.[18] The largest peak in the second derivative of ECG_RMS_ (calculated using a simple numerical differentiation) within a window of 100 to 20 milliseconds (ms) preceding the R peak was regarded to indicate the onset of the QRS complex, see Fig 2.

#### T-wave landmarks

The peak of the T-wave (T_peak_) and the end of the T-wave (T_end_) are estimated for every individual ECG lead as well as for the constructed scalar ECG signals. T-wave landmarks obtained from individual scalar ECG signals or ECG leads are called local T-wave landmarks. Since individual ECG leads are affected by heart axis orientation and scalar ECGs may blur information which is only present in one or two individual ECG leads, local T-wave landmarks are determined from both the individual ECG leads and the scalar ECGs. These effects are minimized by determining one global T-wave landmark from the local T-wave landmarks obtained from ECG leads and scalar ECGs. To detect the local T-wave landmarks, all individual ECG leads and the scalar ECG signals were smoothed using a 2^nd^ order Savitzky Golay filter with a 50ms window. First, the local peak of the T-wave (local T_peak_) was detected as the maximum or minimum peak between the preceding R peak +50ms and the preceding R peak +70% RR of the smoothed signal (see Fig 2). Second, the slope of the maximum deflection between local T_peak_ and local T_peak_ +30% RR was calculated. A tangent through the point with the maximal slope in the final limb of the T-wave was estimated using a simple numerical differentiation within a ten ms window (dVdt(t) = (V(t+5)-V(t-5))/10). The intersection of this tangent and the baseline was used to detect the local end of the T-wave (local T_end_). The baseline was defined as the median amplitude of the 30ms preceding the QRS onset of that particular complex. Local QT-intervals were calculated from the global QRS onset and local T_end_ and can be used for QT dispersion measures.

#### Global T-wave landmarks

From the local T-wave landmarks, a median T_peak_ and T_end_ location was calculated for every complex. Local T_peak_ and T_end_ landmarks that deviated more than two times the standard deviation (SD) from the median T_peak_ and T_end_ were considered to be outliers and excluded. Global T_peak_ and T_end_ locations were calculated as the median from the remaining local T_peak_ and T_end_ locations. Finally, QT-intervals were calculated by calculating the interval between QRS onset and global T_end_. Individual ECG leads with a local T-wave amplitude smaller than 50 μV were considered to be too small for accurate determination of local T_end_ and therefore were not taken into account for the determination of the global Tend of that particular complex. For example, if the T-wave amplitude is low in all limb leads, global T_end_ will be calculated from the local T_end_ of the precordial leads and the scalar ECGs only.

### Validation

From every ECG recording during a supine-standing test, one complex with an RR-interval below the 10^th^ percentile, one complex with an RR-interval above the 90^th^ percentile, and one complex with an RR-interval within the interquartile range were randomly selected by the computer. This resulted in three complexes with a wide range of RR-intervals per supine-standing test. From the randomly selected complexes, QT-intervals were calculated both automatically using the algorithm described above and manually by three independent observers (BH, FB, TD). The manual measurements of the QT-intervals were done on paper using the tangent approach in a lead of choice.[15] All observers measured the QT-interval with an accuracy of 0.5mm, which corresponded with 6ms. The observers were blinded for patient characteristics, QT-intervals determined by the algorithm and the measurements of the other observers. The algorithm was validated by determining the inter-method variability between the QT-interval measured by the algorithm (QTalg) and (I) the individual manual QT-interval measurements (QTobs1, QTobs2 and QTobs3) and (II) the mean QT-interval from all the individual measurements (μQTobs). In addition, the QT-interval measurements of the three observers were compared in order to assess the inter-observer variability.

#### Statistical analysis

Statistical analyses were performed in Matlab. Patient and ECG characteristics were presented in frequencies (percentage) for categorical variables and mean (± SD) for continuous variables with an approximately symmetric distribution. The inter-method variability and the inter-observer variability were expressed as correlation coefficients estimated by a Pearson correlation test, and the intra-class correlation coefficient (ICC) for single and averaged measurements based on a two-way mixed absolute agreement model.[19] Sample uncertainty was expressed as 95% confidence intervals (95% CI). Bland-Altman analyses were performed to assess the systematic bias and the limits of agreement for both the inter-method and the inter-observer variability.[20] A p-value < 0.05 was considered to be statistically significant.

## Results

### Population

The total study population of 127 subjects included 34 (26.8%) subjects with LQT1, 28 (22.0%) with LQT2, 11 (8.7%) LQT3, and 54 (42.5%) controls. The characteristics of the study population are shown in Table 1.

**Table 1:** Characteristics of the study population.

	Gender (M/F)	Age (years)	Number (-)	QTc at low RR (ms)	QTc at mid RR (ms)	QTc at high RR (ms)
LQT1	13 / 21	33.9 ± 13.9	34	486 ± 44	460 ± 37	439 ± 33
LQT2	17 / 11	40.5 ± 15.1	28	498 ± 49	449 ± 35	427 ± 34
LQT3	4 / 7	35.2 ± 15.2	11	472 ± 45	439 ± 35	422 ± 35
Control	31 / 23	40.8 ± 15.8	54	446 ± 38	410 ± 26	392 ± 25
Total	65 / 62	38.4 ± 15.2	127	469 ± 47	435 ± 39	415 ± 36

Data are given as mean ± standard deviation. M = male, F = female, RR = RR-interval, QTc = Corrected QT using Bazett’s formula.

### Validation

The randomly computer based selected complexes were visually inspected and eight complexes (2.1%) had to be excluded based on the presence of artefacts, three complexes because of too flat T-waves in all ECG leads (0.8%), and two (0.5%) because the randomly chosen complex was a premature ventricular complex.

In the remaining 358 complexes, the RR-intervals ranged from 470ms to 1419ms, with a mean RR of 849ms (± 194ms). The mean heart axis was 42° (± 42°) and the 95% percentile confidence interval (PCI: 2.5^th^ and 97.5^th^ percentile of the data) ranged from -45° to 119°. The mean T-wave axis was 26° (± 42°) with a 95% PCI ranging from -85° to 108°.

**Fig 3:**
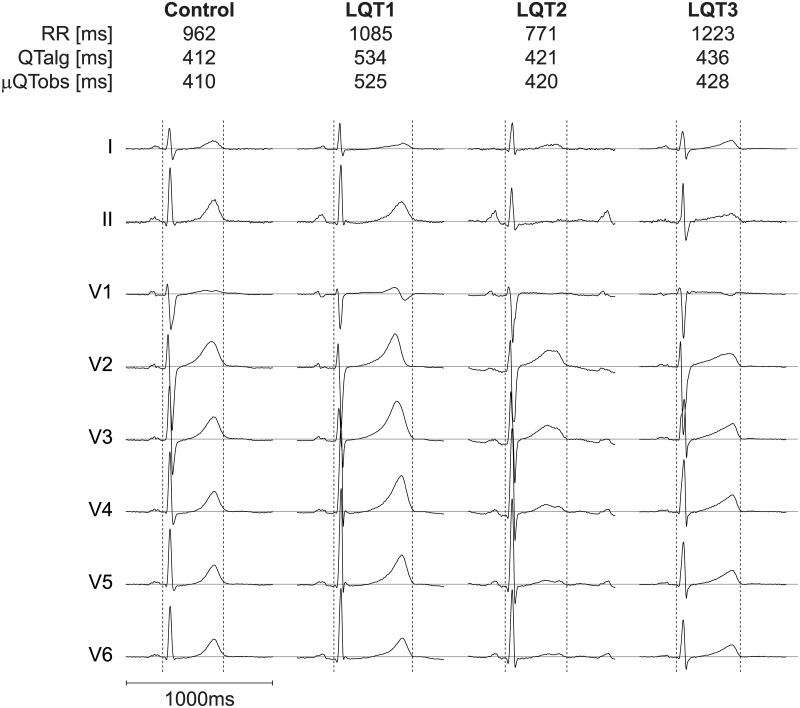
An example of the results of our algorithm. The QRS onset and global Tend detected by the algorithm is shown for a healthy control and patients with LQT-1, 2 and 3. QTalg = QT-interval determined by the algorithm, μQTobs = mean QT-interval determined by three observers, ms = milliseconds.

By visual inspection by one of the observers, 127 (35%) complexes had aberrant T-waves and/or prominent U-waves. Fig 3 shows an example of one complex with the QRS onset and global T_end_ detected by the algorithm for an LQT1, LQT2 and LQT3 patient as well as for a control.

#### Inter-method variability

**Fig 4:**
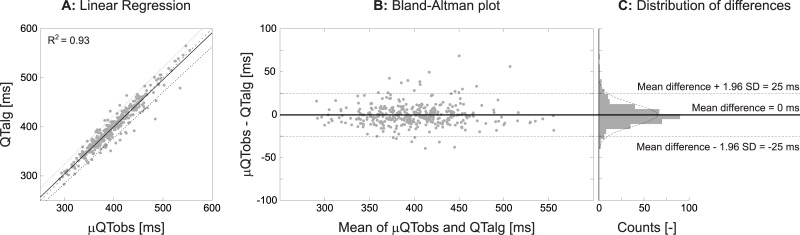
Validation results of the μQTobs VS QTalg. **A** linear regression between μQTobs and QTalg. **B** Bland-Altman analysis shows no bias (solid black line) and narrow limit of agreements (dashed lines). **C** The Distribution of differences shows that the differences are normally distributed around zero. All numbers corresponding with this figure can be found in Table 2. QTalg = QT-interval determined by the algorithm, μQTobs = mean QT-interval determined by three observers, SD = standard deviation, ms = milliseconds.

Results of the comparison between the QTalg and the individual observers are shown in Table 2. There was a strong correlation (Pearson’s r ranging from 0.935 to 0.959) and agreement (ICC ranging from 0.933 to 0.956) between the QTalg and the individual observers, with a systematic bias ranging between -1.88ms and 3.39ms. Fig 4 shows the Bland-Altman plot for the inter-method variability of QTalg and μQTobs. The correlation and agreement between QTalg and μQTobs was also strong (r = 0.962, ICC = 0.981).

**Table 2:** Inter-method variability.

	Pearson correlation		Intra-class coefficient		Bland-Altman	
	r (95% CI)	p	ICC (95% CI)	p	Mean difference (ms)	Limits of agreement (ms)
QTalg vs. QTobs1	0.959 (0.949–0.966)	< 0.001	0.956 (0.943–0.966)	< 0.001	3.39	-23.23: 30.01
QTalg vs. QTobs2	0.935 (0.920–0.946)	< 0.001	0.933 (0.917–0.945)	< 0.001	-2.65	-36.09: 30.79
QTalg vs. QTobs3	0.948 (0.936–0.957)	< 0.001	0.947 (0.935–0.957)	< 0.001	-1.88	-31.53: 27.78
QTalg vs. μQTobs	0.962 (0.954–0.969)	< 0.001	0.981 (0.976–0.984)	< 0.001	-0.38	-25.41: 24.65

95% CI = 95% confidence interval, obs = observer(s), r = Pearson’s r, p = p-value, ICC = intra-class coefficient, ms = milliseconds.

LQTS type specific validation showed similar agreements between QTalg and μQTobs for all LQTS types (ICC ranging from 0.934 (LQT2) to 0.989 (LQT1). See supporting information: S1 Fig and S1 Table).

#### Inter-observer variability

Results of the comparison between observers are shown in Table 3. The agreement between all observers was strong (Pearson’s r ranging from 0.945 to 0.964, ICC ranging from 0.939 to 0.958). The Bland-Altman analysis showed that the inter-observer bias ranged from -0.74ms to 6.28ms. The limits of agreement range from 26ms to 31ms.

**Table 3:** Inter-observer variability.

	Pearson correlation		Intra-class coefficient		Bland-Altman	
	r (95% CI)	p	ICC (95% CI)	p	Mean difference (ms)	Limits of agreement (ms)
QTobs1 vs. QTobs2	0.947 (0.935–0.957)	< 0.001	0.939 (0.908–0.958)	< 0.001	6.28	-24.67: 37.23
QTobs1 vs. QTobs3	0.964 (0.955–0.970)	< 0.001	0.958 (0.932–0.972)	< 0.001	5.54	-20.41: 31.49
QTobs2 vs. QTobs3	0.945 (0.934–0.956)	< 0.001	0.946 (0.934–0.956)	< 0.001	-0.74	-31.06: 29.58

95% CI = 95% confidence interval, obs = observer(s), r = Pearson’s r, p = p-value, ICC = intra-class coefficient, ms = milliseconds.

## Discussion

We have developed and validated an automatic QT-interval algorithm based on the tangent method which is unaffected by heart axis orientation and that can be applied on a beat-to-beat basis regardless of the T-wave morphology. There is a high agreement between the automatic algorithm and manual measurements of the QT-interval. Measuring errors between our algorithm and manual measurements are similar or even smaller than inter-observer measuring errors. In contrast to manual measurements, our algorithm enables users to study large amounts of complexes. Therefore, it can be used to study novel QT-interval parameters that require beat-to-beat QT-interval analysis.

### Measuring the QT-interval

Recognition of an abnormal QT-interval is an important element to gain an impression of the risk for malignant arrhythmias and it guides treatment. However, determination of the QT-interval can be challenging [4] and its result may frustrate treatment.[6] For manual QT assessment, the tangent method in lead II or V5 has been proposed. It has been suggested that with this method even inexperienced ECG readers can, after minimal education, accurately diagnose prolonged and normal QT-intervals.[15,21] However, manual assessment has considerable limitations. Proper manual QT-interval assessment is time consuming. Therefore, most physicians pick one lead and one complex to measure. Whether the QT-interval from the measured complex in the chosen lead is representative for the patient can be questionable. Measuring QT-intervals of multiple complexes over all leads is too time-consuming for daily clinical practice. Therefore, objective, standardized automated QT-interval algorithms unaffected by heart axis orientation are desirable.

#### Algorithms by manufacturers

All modern ECG machines provide users with automated measurements of ECG intervals. A general downside of these algorithms is that the QT-interval is determined on an averaged complex over time (cf. Appendix Kligfield et al.[13]). Therefore, temporal fluctuations in QT-interval are lost and the dynamicity and adaptation of the QT-interval to changes in heart rate cannot be studied using these algorithms. Another downside of these algorithms is that the details about the algorithms are often unavailable for their users. Despite the latter, many cardiologists do use and trust the QTc-interval provided by the ECG machine. Using a custom-made algorithm enables visualisation of the determined QRS-onset and T-wave end, making it easier to distinguish between correct and erroneous measurements.

#### Custom-made QT-interval algorithms

Custom-made (semi-)automated QT-interval algorithms were developed in order to study QT dynamics. Berger et al. [14] for example, described a template matching algorithm to study QT dynamics. In his algorithm, a template (which is selected by the user) is matched to all complexes in order to measure individual QT-intervals. A disadvantage of his algorithm is that it only uses one ECG lead (I or II) and is therefore susceptible to heart axis orientation. For example, an algorithm that uses only lead II will most likely be unable to define T_end_ in the ECG of the LQT2 patient as shown in Fig 3, due to the low T-wave amplitude in ECG lead II. Since our algorithm takes all leads into account, it is still able to define T_end_ as long as the T-wave is large enough in at least one lead (see Fig 3).

More sophisticated single- and multilead algorithms have also been reported.[22–26] However, a general downside of those techniques is that they have not yet been validated for LQTS patients with various T-wave morphologies. Therefore it remains unknown how well these algorithms perform in T-wave morphologies alternated by LQTS. Almeida et al. proposed a multi-lead ECG delineation algorithm which has been validated against multiple annotated databases. [26] Almeida et al. report mean differences of 7.5 ± 11.2 ms and 7.9 ± 21.7 ms for the QRS-onset and T-wave end detection, respectively. [26] Although the exact mean differences in QT-interval can’t be perceived from these results, the mean differences of QRS-onset and T-wave end detection suggest the differences in QT-interval to be similar to our validation results.

### Development

Our algorithm is an extensive automated version of the tangent method first described by Lepeschkin and Surawicz.[15] The tangent method has been shown to be an accurate and reproducible method for diagnosing prolonged QT-intervals, even by inexperienced ECG readers.[21] We applied this method to all 12 standard ECG leads as well as to the three constructed scalar ECGs (ECG_RMS_, ECG_SD_, |VCG|) to make it unaffected by heart axis orientation and applicable regardless of the T-wave morphology. Since prominent U-waves, notches, low T-waves and other altered T-wave morphologies often occur in only a few ECG leads, morphology-induced erroneous local T_end_ detections will not affect the global T_end_.

It is important to bear in mind that though our algorithm is applicable regardless of the T-wave morphology, the T-wave morphology on its own can still be useful for the diagnosis of LQTS.

The isoelectric baseline was defined as the median amplitude of the 30ms preceding a QRS onset. The P-Q segment was chosen instead of the T(U)-P segment because the PQ segment is less affected by heart rate changes since at high heartrates the P wave can coincide with the T-wave.

### Validation

The results of our validation study show good agreements between observers and our algorithm. The mean differences and limits of agreements between the observers and our algorithm are in the same range as the inter-observers differences in this study as well as in a previous study.[27] The same holds for the results from ICC. This suggests that our algorithm is as accurate in determining the QT-interval as the observers.

The QT-intervals measured by the observers and the algorithm had an approximately normal distribution. To be sure not to make mistakes by using parametric tests, Spearman’s correlation test and the Kendal’s W coefficient of concordance were also computed and the results were compared with the Pearson’s correlation test and the intra-class correlation coefficient. The differences between the parametric and non-parametric tests were small and the results of the non-parametric tests did not change the conclusion.

From our results we conclude that our algorithm is a good alternative for manual QT-interval measurements. Moreover, because the algorithm is unaffected by heart axis orientation and can provide beat-to-beat QT-intervals, it might have an additional value in diagnosing LQTS and evaluating new drugs.

### Advantages

Additional advantages of our algorithm are that we are the first to combine T-wave landmarks derived from individual ECG leads with landmarks derived from scalar ECGs. By doing so, the algorithm combines the better of two worlds. The scalar ECGs are independent to heart axis orientation but since they are a mean (ECG_RMS_), standard deviation (ECG_SD_) or weighted mean (VCG) of individual ECG leads, information which is only present in one or two ECG leads is blurred and would have been lost if our algorithm wouldn’t have used also the individual ECG leads. By calculating the median T_end_ after outlier removal, the global T_end_ is based on both the scalar ECGs and individual ECG leads. Secondly, the ECG_RMS_ and ECG_SD_ are calculated from unipolar ECG leads only. By doing so, all ECG leads contribute equally to the scalar ECGs. Another advantage is that our algorithm treats every complex individually and it does not require *a priori* knowledge. Methods like the one described by Ritsema van Eck [23] might run into problems by sudden changes in T-wave morphology because each individual complex is cross-correlated with the average of the remainder complexes. Lastly, we described all necessary details to rebuild the algorithm and kept it as simple as possible. By doing so, the algorithm is reproducible and understandable for future users and clinicians.

### Limitations

Although the algorithm had a high agreement with manual measurements, we acknowledge it has some limitations. First, low T-wave amplitude will result in a smaller signal-to-noise ratio and therefore might result in a larger error in T_peak_ and T_end_ detection. This is partially dealt with by excluding individual complexes on individual ECG leads if that particular complex has a T-wave amplitude smaller than 50 μV. However, by excluding individual complexes on certain ECG leads, the remaining ECG leads become more important in those complexes. If respiration affects T-wave amplitude, a different number of individual ECG signals might be used for every complex within one respiratory cycle. This might induce detected QT variability.

Second, T_end_ detection using the tangent approach is influenced by baseline deviations. Robust baseline determination techniques are rare and although the validation of our algorithm was successful, improving baseline determination might still improve the outcome.

Baumert et al. [28] stated that conventional QT algorithms are not the best choice to measure beat-to-beat QT-interval changes. However, the conventional QT algorithm that has been studied in this article is a threshold method on the first derivative of a single lead ECG. Since our algorithm is based on the tangent method and takes all leads into account, the statement from Baumert et al. [28] can’t be projected on our algorithm. To find out whether our algorithm can be used for beat-to-beat QT-interval parameters as described in the position paper from Baumert et al. [9], a validation focused on these parameters is required.

Lastly, the observers measured the QT-interval from one lead only, while the algorithm takes all leads into account. However, since there is no true gold standard in the measurement of the QT-interval, we chose to validate the algorithm against the most objective manual assessment available.

## Conclusion

Our validation results show that the QT-interval detection algorithm is as accurate in determining QT-intervals as instructed manual observers are. Since the algorithm is fast, objective, unaffected by heart axis orientation, applicable regardless of the T-wave morphology and can provide beat-to-beat QT-intervals, the algorithm might be useful to help improving the diagnosis of LQTS or the evaluation of QT-interval prolonging effects of new drugs.

## Supporting information

S1 Fig LQTS specific validation of μQTobs VS QTalg. Bland-Altman analysis shows the bias (solid black line) and the limit of agreements (dashed lines) per LQTS type. QTalg = QT-intervals determined by the algorithm, μQTobs = mean QT-interval determined by three observers, ms = milliseconds.
(EPS)

S1 Table LQTS specific validation results. 95% CI = 95% confidence interval, obs = observer(s), r = Pearson’s r, p = p-value, ICC = intra-class coefficient, ms = milliseconds.
(DOCX)

S1 Dataset Randomly selected complexes. (PDF)
