# Supplementary material for: The development and validation of an easy to use automatic QT-interval algorithm
Source: PLoS One. 2017 Sep 1;12(9):e0184352. doi: 10.1371/journal.pone.0184352 (PMC5581168; doi:10.1371/journal.pone.0184352)
Supplement: S1 Table — 95% CI = 95% confidence interval, obs = observer(s), r = Pearson’s r, p = p-value, ICC = intra-class coefficient, ms = milliseconds. (DOCX) [file pone.0184352.s002.docx]

S1 Table LQTS specific validation results

|  |  |  |  | **Pearson correlation** | | | **Intra-class coefficient** | | | **Bland-Altman** | |
| --- | --- | --- | --- | --- | --- | --- | --- | --- | --- | --- | --- |
|  |  |  |  | r (95% CI) | | p | ICC (95% CI) | | p | Mean difference (ms) | Limits of agreement (ms) |
| Control | QTalg | vs. | μQTobs | 0.953 | (0.936 – 0.966) | < 0.001 | 0.976 | (0.967 – 0.982) | < 0.001 | -0.26 | -20.78 : 20.26 |
|  | QTobs1 | vs. | QTobs2 | 0.923 | (0.896 – 0.944) | < 0.001 | 0.915 | (0.872 – 0.942) | < 0.001 | 4.90 | -22.64 : 32.45 |
|  | QTobs1 | vs. | QTobs3 | 0.959 | (0.944 – 0.970) | < 0.001 | 0.951 | (0.916 – 0.969) | < 0.001 | 4.32 | -15.55 : 24.19 |
|  | QTobs2 | vs. | QTobs3 | 0.903 | (0.869 – 0.928) | < 0.001 | 0.902 | (0.868 – 0.928) | < 0.001 | -0.58 | -31.14 : 29.97 |
| LQT1 | QTalg | vs. | μQTobs | 0.979 | (0.968 – 0.986) | < 0.001 | 0.989 | (0.983 – 0.993) | < 0.001 | -2.30 | -23.52 : 18.92 |
|  | QTobs1 | vs. | QTobs2 | 0.972 | (0.959 – 0.981) | < 0.001 | 0.960 | (0.877 – 0.982) | < 0.001 | 8.39 | -15.63 : 32.41 |
|  | QTobs1 | vs. | QTobs3 | 0.974 | (0.961 – 0.982) | < 0.001 | 0.966 | (0.919 – 0.982) | < 0.001 | 6.76 | -16.58 : 30.09 |
|  | QTobs2 | vs. | QTobs3 | 0.971 | (0.957 – 0.980) | < 0.001 | 0.971 | (0.957 – 0.980) | < 0.001 | -1.63 | -26.20 : 22.93 |
| LQT2 | QTalg | vs. | μQTobs | 0.879 | (0.815 – 0.922) | < 0.001 | 0.934 | (0.896 – 0.958) | < 0.001 | 3.33 | -31.66 : 38.33 |
|  | QTobs1 | vs. | QTobs2 | 0.868 | (0.798 – 0.914) | < 0.001 | 0.864 | (0.793 – 0.912) | < 0.001 | 3.89 | -34:87 : 42.66 |
|  | QTobs1 | vs. | QTobs3 | 0.891 | (0.832 – 0.930) | < 0.001 | 0.888 | (0.827 – 0.927) | < 0.001 | 3.79 | -31.18 : 38.76 |
|  | QTobs2 | vs. | QTobs3 | 0.889 | (0.830 – 0.928) | < 0.001 | 0.890 | (0.832 – 0.929) | < 0.001 | -0.11 | -35.86 : 35.65 |
| LQT3 | QTalg | vs. | μQTobs | 0.944 | (0.886 – 0.974) | < 0.001 | 0.969 | (0.934 – 0.986) | < 0.001 | -4.14 | -28.22 : 19.93 |
|  | QTobs1 | vs. | QTobs2 | 0.870 | (0.743 – 0.937) | < 0.001 | 0.844 | (0.652 – 0.928) | < 0.001 | 9.73 | -27.66 : 47.13 |
|  | QTobs1 | vs. | QTobs3 | 0.953 | (0.903 – 0.978) | < 0.001 | 0.929 | (0.719 – 0.974) | < 0.001 | 9.10 | -14.03 : 32.23 |
|  | QTobs2 | vs. | QTobs3 | 0.900 | (0.798 – 0.952) | < 0.001 | 0.901 | (0.802 – 0.952) | < 0.001 | -0.63 | -33.96 : 32.69 |

*95% CI = 95% confidence interval, obs = observer(s), r = Pearson’s r, p = p-value, ICC = intra-class coefficient, ms = milliseconds*
