# Supplementary material for: The development and validation of an easy to use automatic QT-interval algorithm
Source: PLoS One. 2017 Sep 1;12(9):e0184352. doi: 10.1371/journal.pone.0184352 (PMC5581168; doi:10.1371/journal.pone.0184352)
Supplement: S1 Dataset — (PDF) [file pone.0184352.s003.pdf]

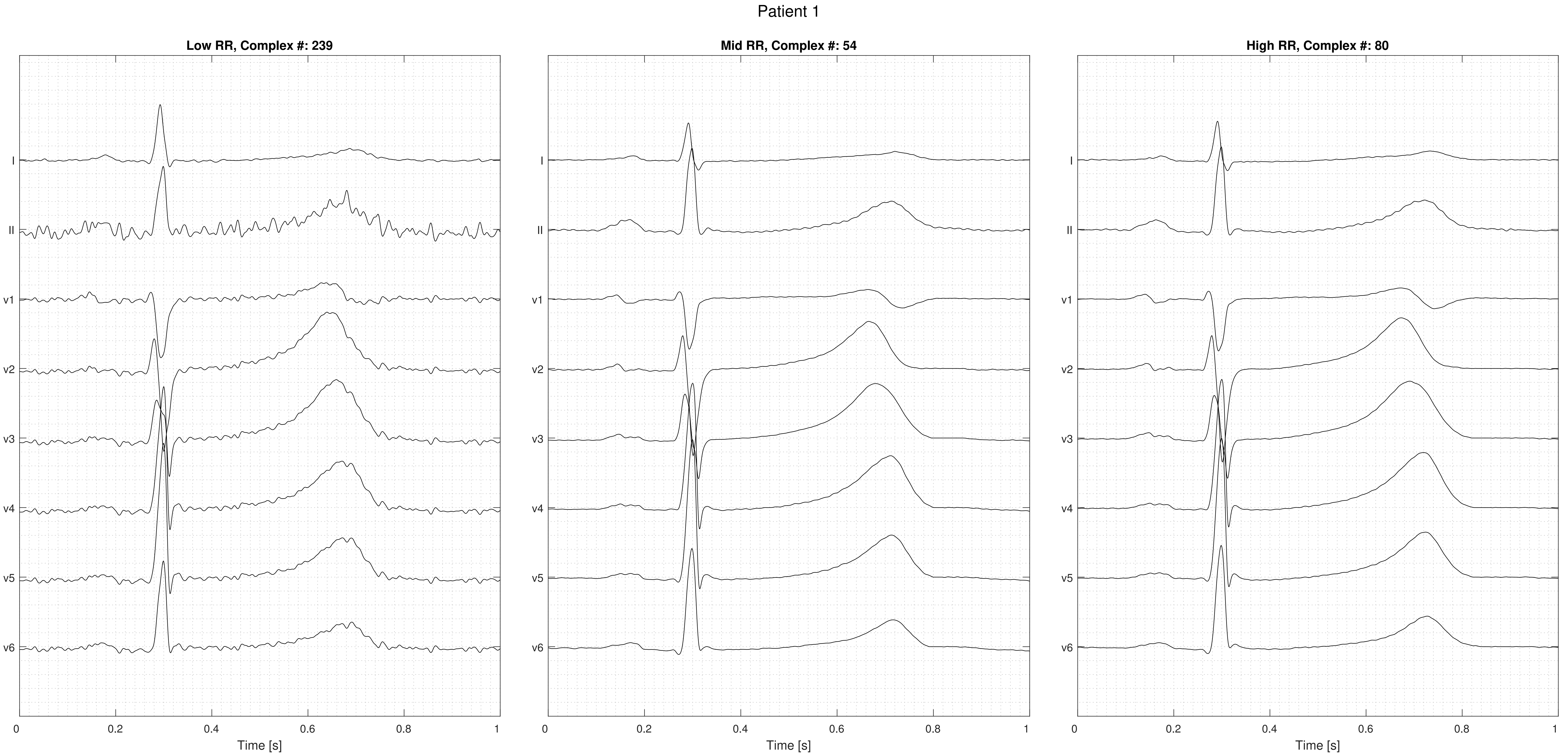

Patient 1
Low RR, Complex #: 239
Mid RR, Complex #: 54
High RR, Complex #: 80
I
II
v1
v2
v3
v4
v5
v6
0
0.2
0.4
0.6
0.8
1
Time [s]

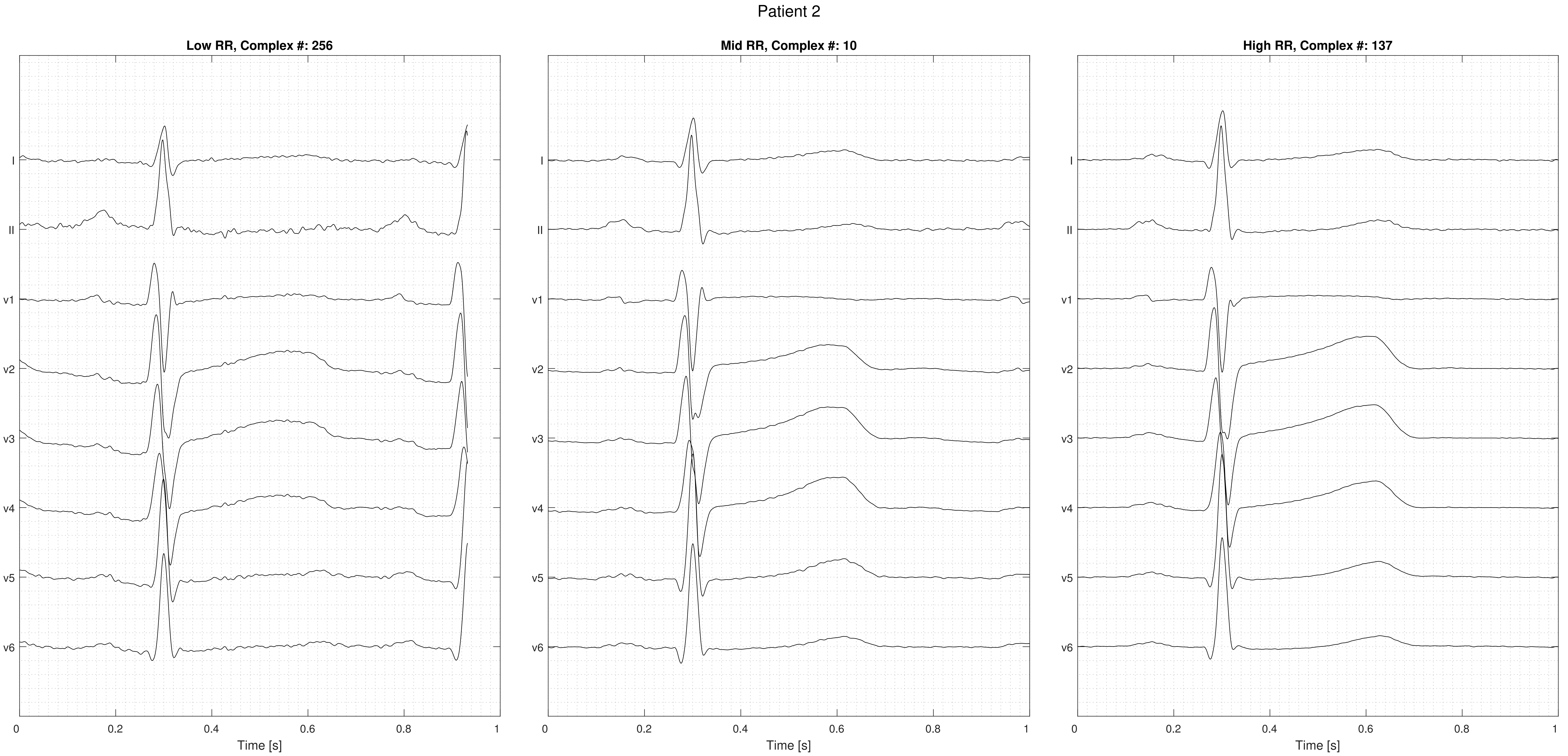

Patient 2
Low RR, Complex #: 256
Mid RR, Complex #: 10
High RR, Complex #: 137
I
II
v1
v2
v3
v4
v5
v6
0
0.2
0.4
0.6
0.8
1
Time [s]

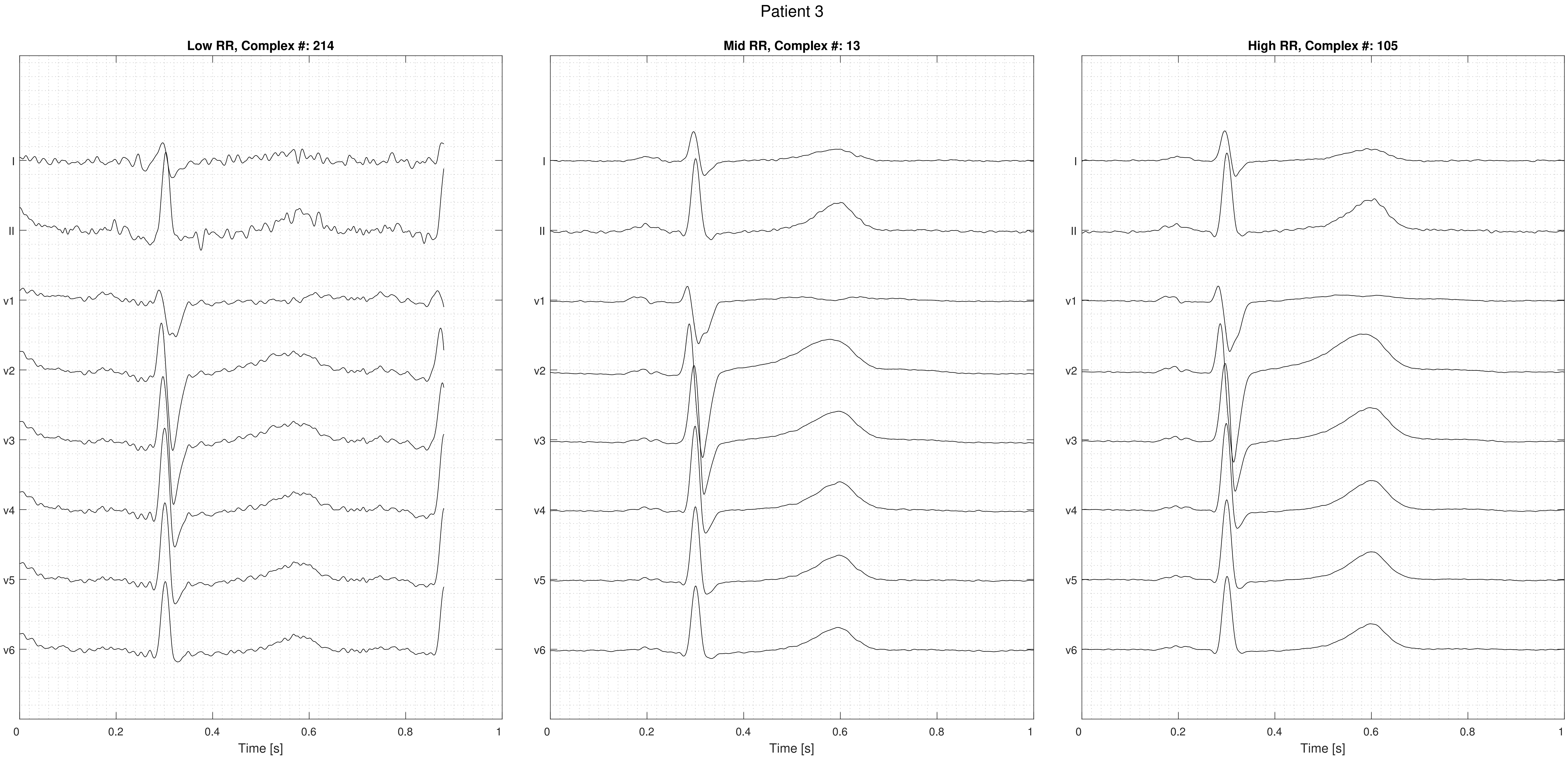

Patient 3
Low RR, Complex #: 214
Mid RR, Complex #: 13
High RR, Complex #: 105
I
II
v1
v2
v3
v4
v5
v6
0
0.2
0.4
0.6
0.8
1
Time [s]

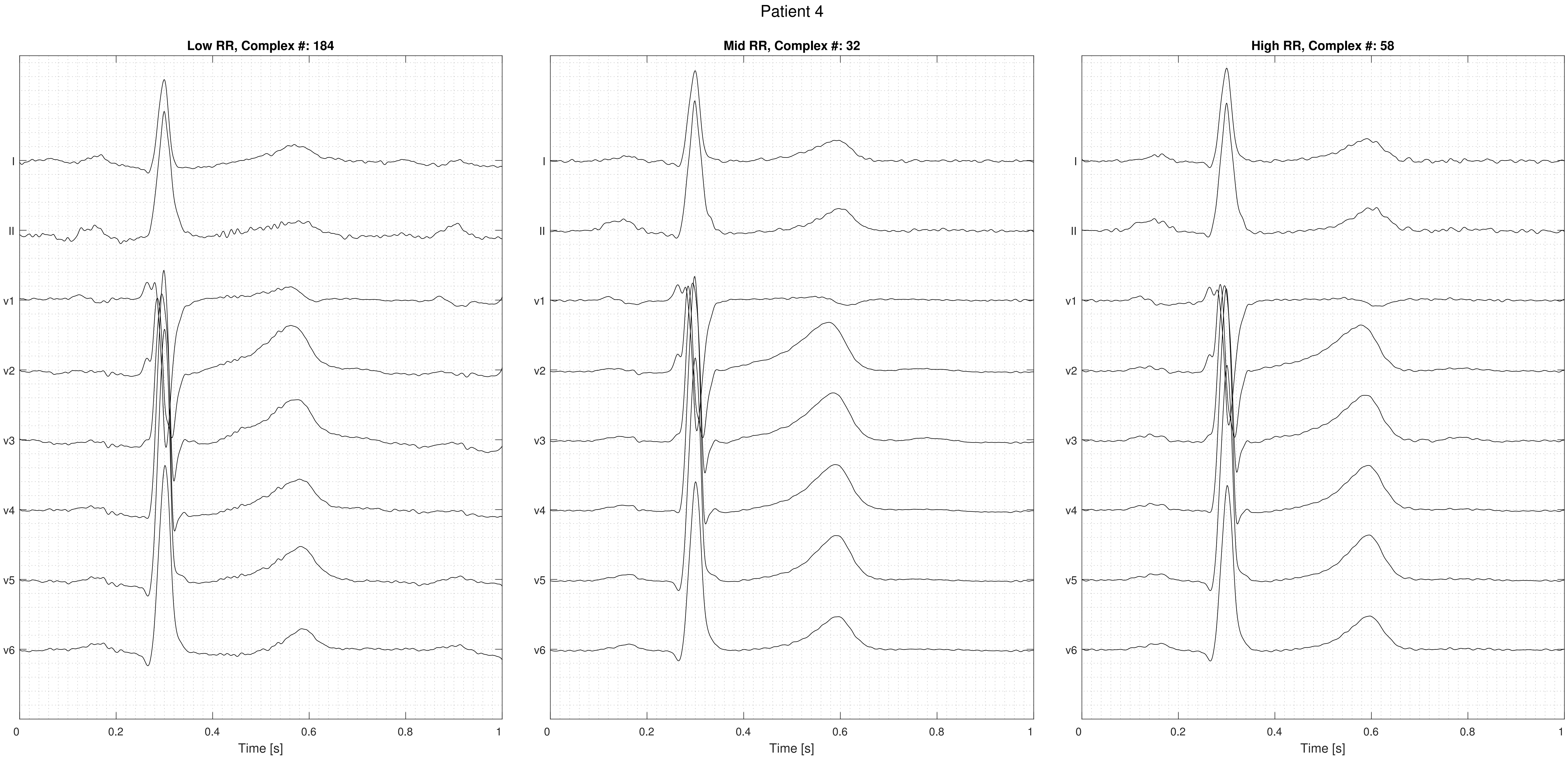
Patient 4
Low RR, Complex #: 184
Mid RR, Complex #: 32
High RR, Complex #: 58
I
II
v1
v2
v3
v4
v5
v6
0
0.2
0.4
0.6
0.8
1
Time [s]

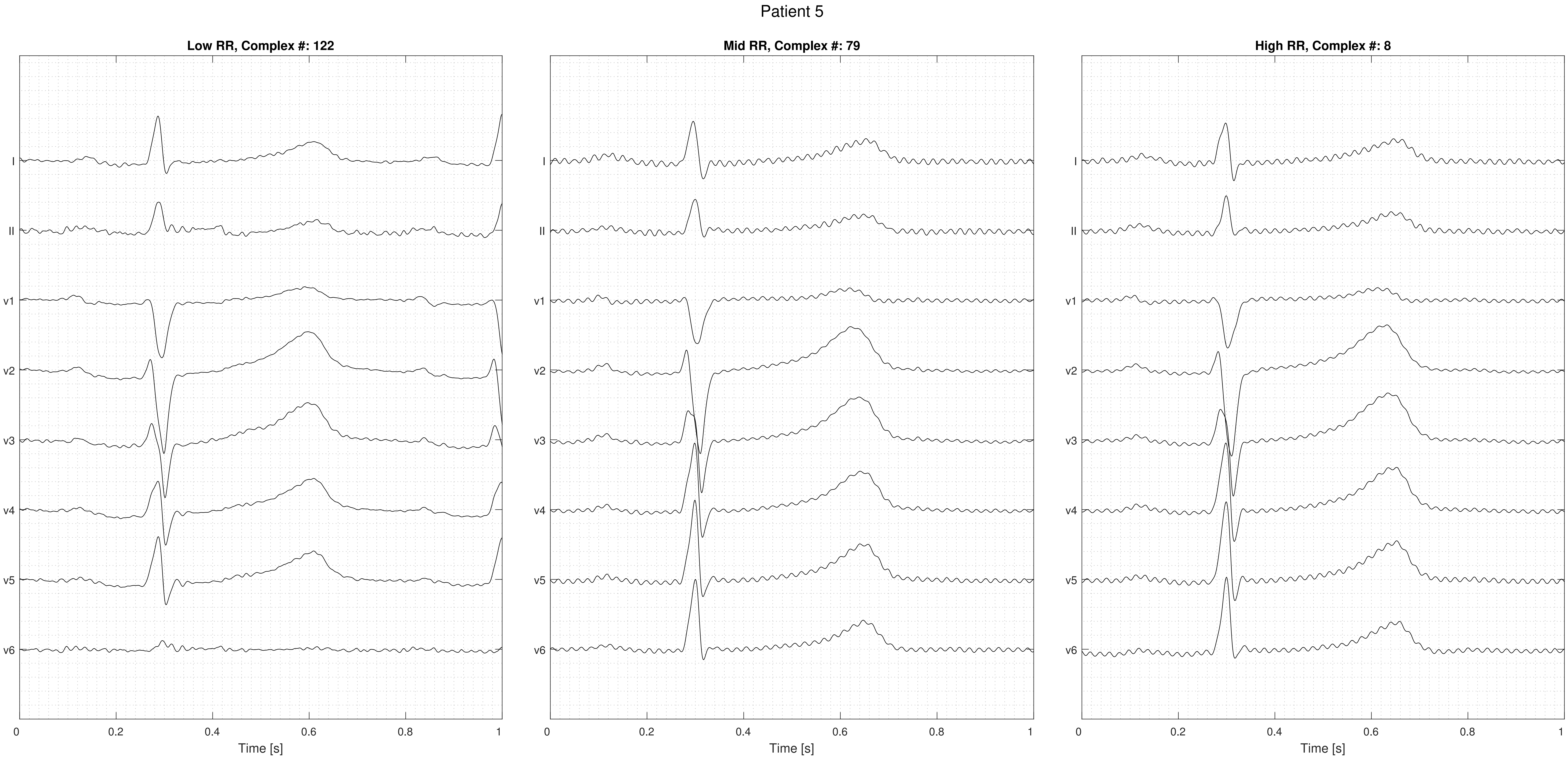
Patient 5
Low RR, Complex #: 122
Mid RR, Complex #: 79
High RR, Complex #: 8
I
II
v1
v2
v3
v4
v5
v6
0
0.2
0.4
0.6
0.8
1
Time [s]

Patient 6

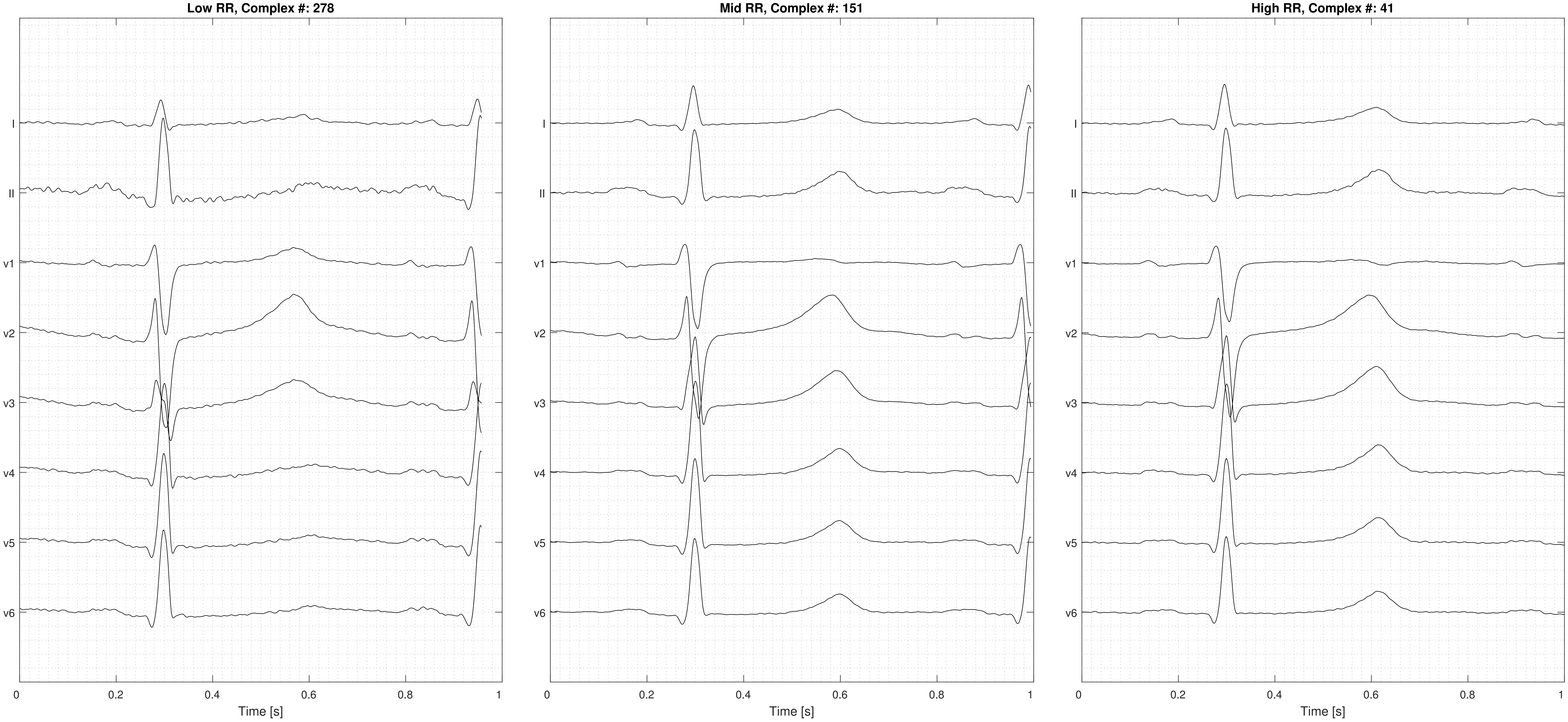

Low RR, Complex #: 278
Mid RR, Complex #: 151
High RR, Complex #: 41
I
II
v1
v2
v3
v4
v5
v6
0
0.2
0.4
0.6
0.8
1
Time [s]

Patient 7

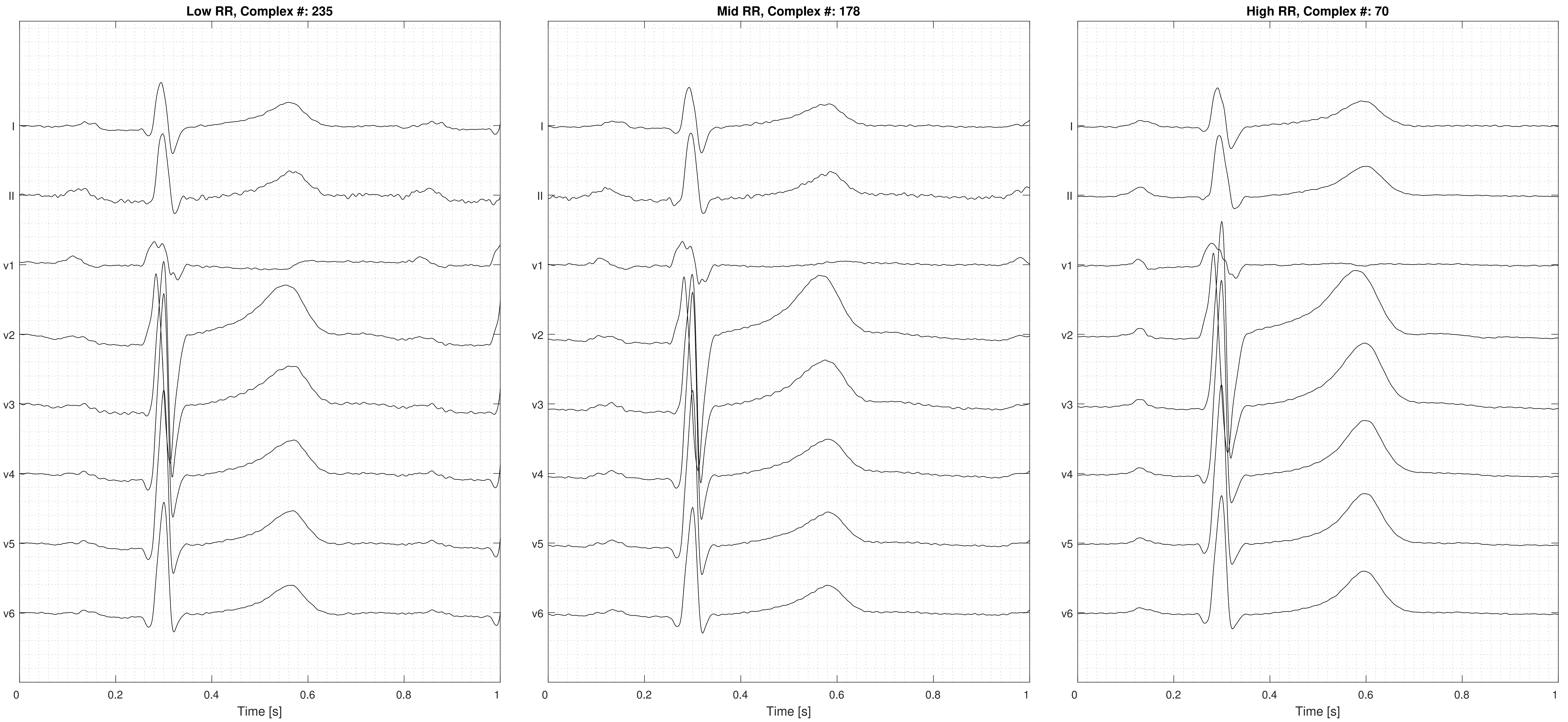

Low RR, Complex #: 235
Mid RR, Complex #: 178
High RR, Complex #: 70
I
II
v1
v2
v3
v4
v5
v6
0
0.2
0.4
0.6
0.8
1
Time [s]

Patient 8

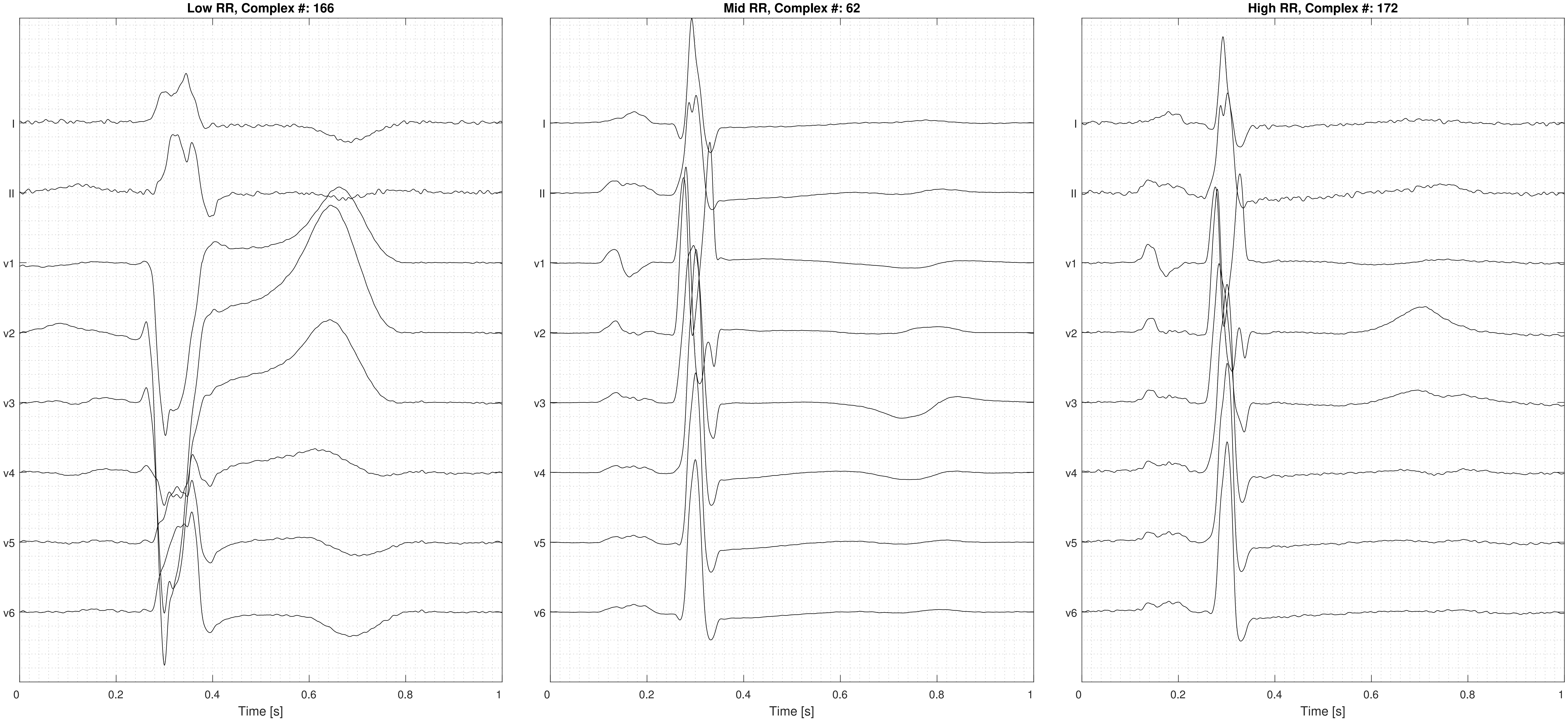

Low RR, Complex #: 166
Mid RR, Complex #: 62
High RR, Complex #: 172
I
II
v1
v2
v3
v4
v5
v6
0
0.2
0.4
0.6
0.8
1
Time [s]

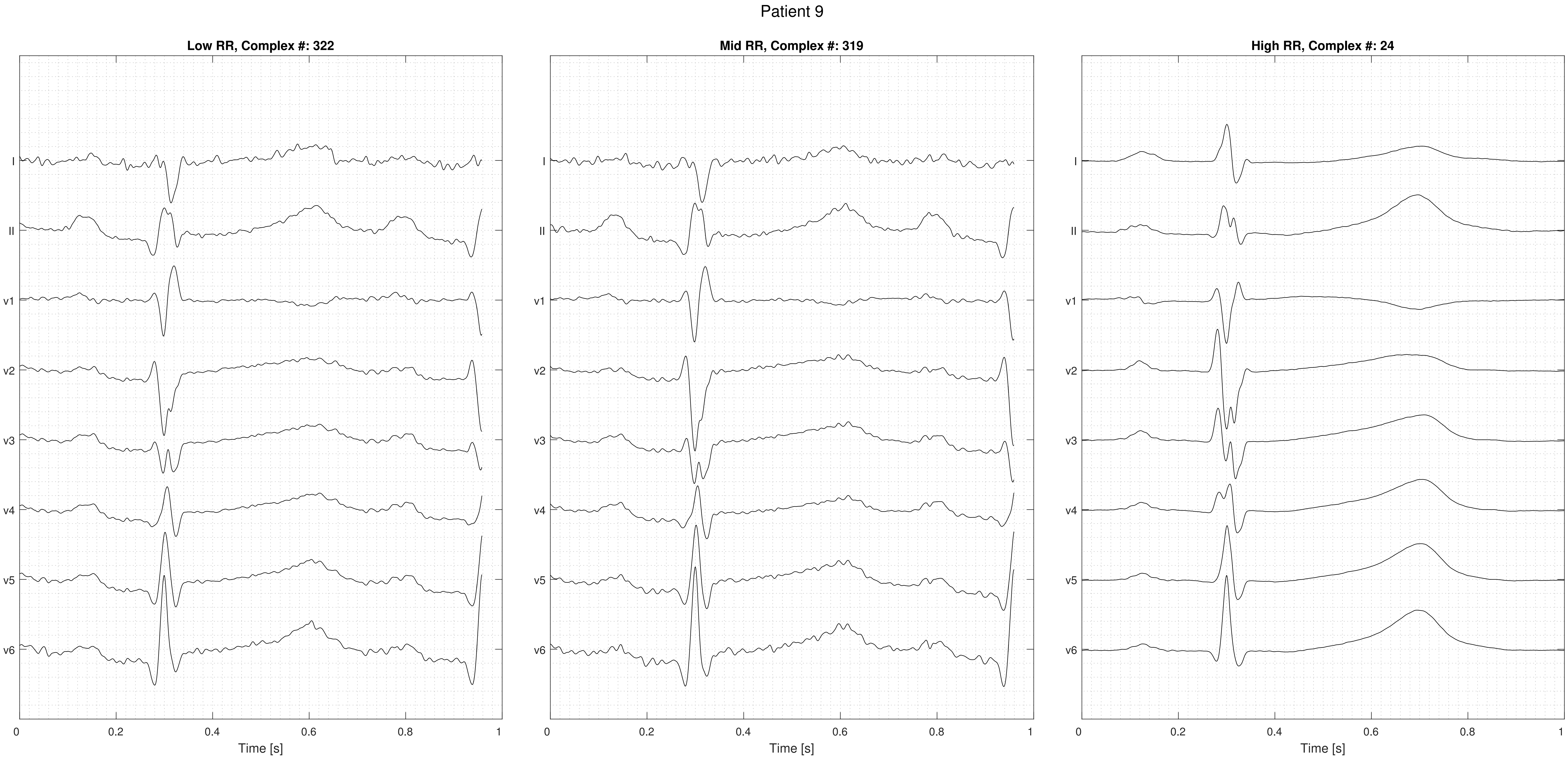
Patient 9
Low RR, Complex #: 322
Mid RR, Complex #: 319
High RR, Complex #: 24
I
II
v1
v2
v3
v4
v5
v6
0
0.2
0.4
0.6
0.8
1
Time [s]

Patient 10

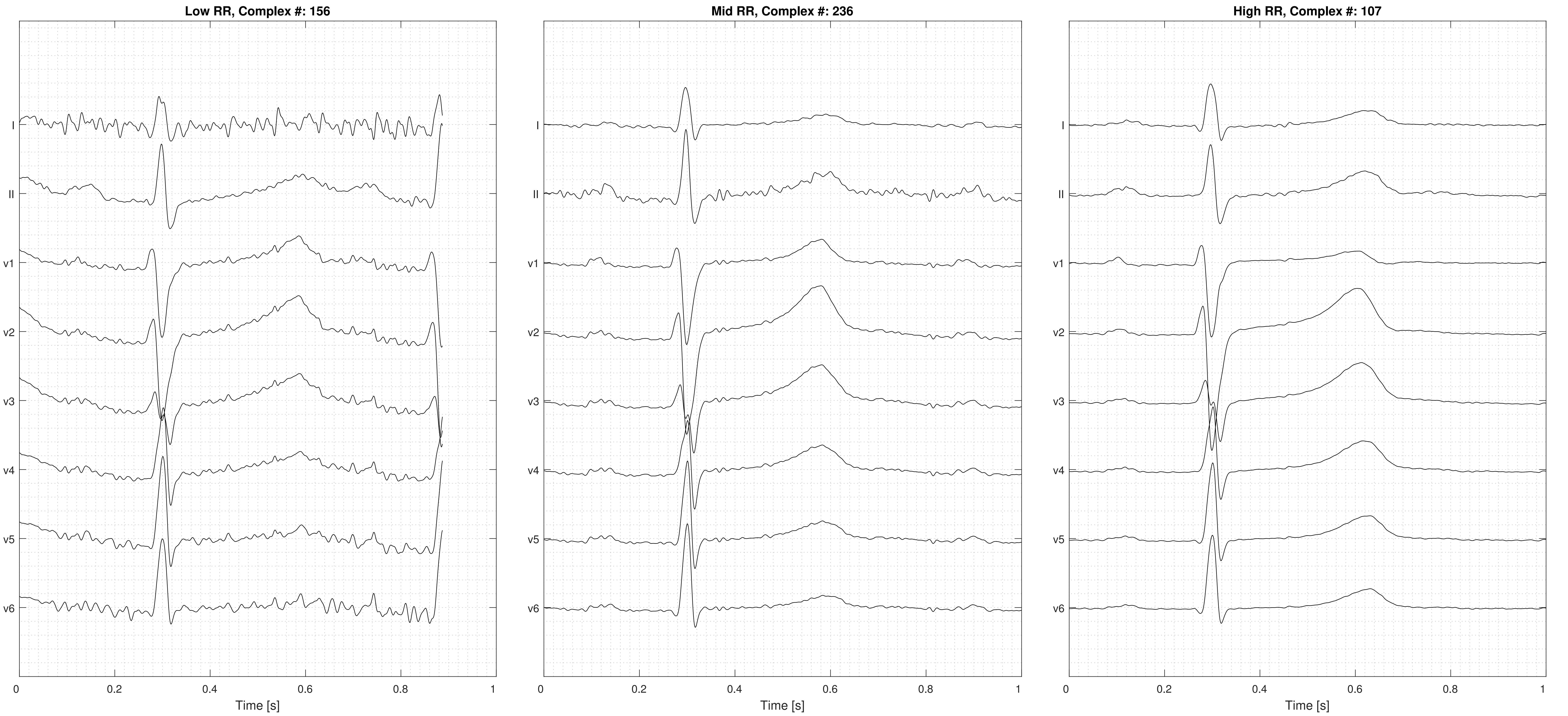

Low RR, Complex #: 156
Mid RR, Complex #: 236
High RR, Complex #: 107
I
II
v1
v2
v3
v4
v5
v6
0
0.2
0.4
0.6
0.8
1
Time [s]

Patient 11

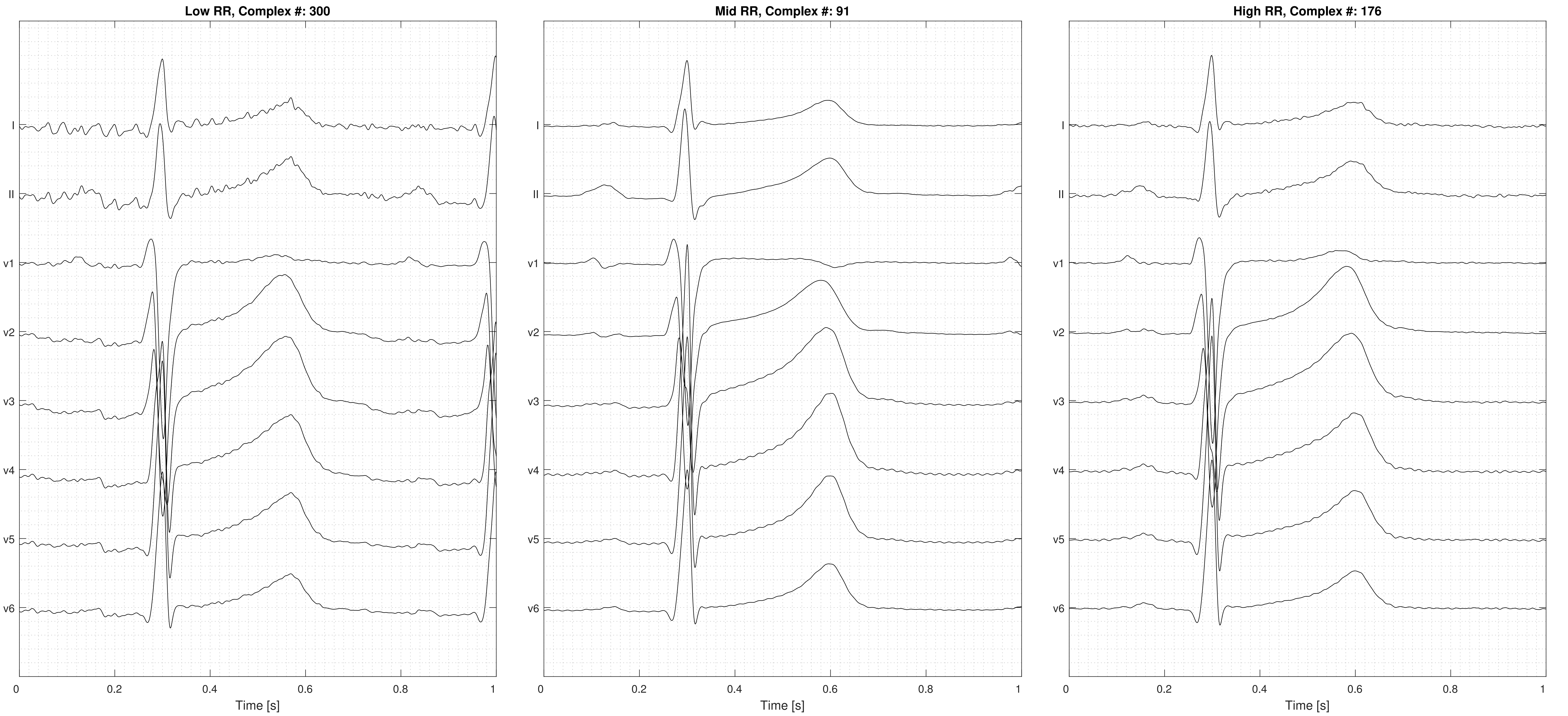
Low RR, Complex #: 300
Mid RR, Complex #: 91
High RR, Complex #: 176
I
II
v1
v2
v3
v4
v5
v6
0
0.2
0.4
0.6
0.8
1
Time [s]

Patient 12

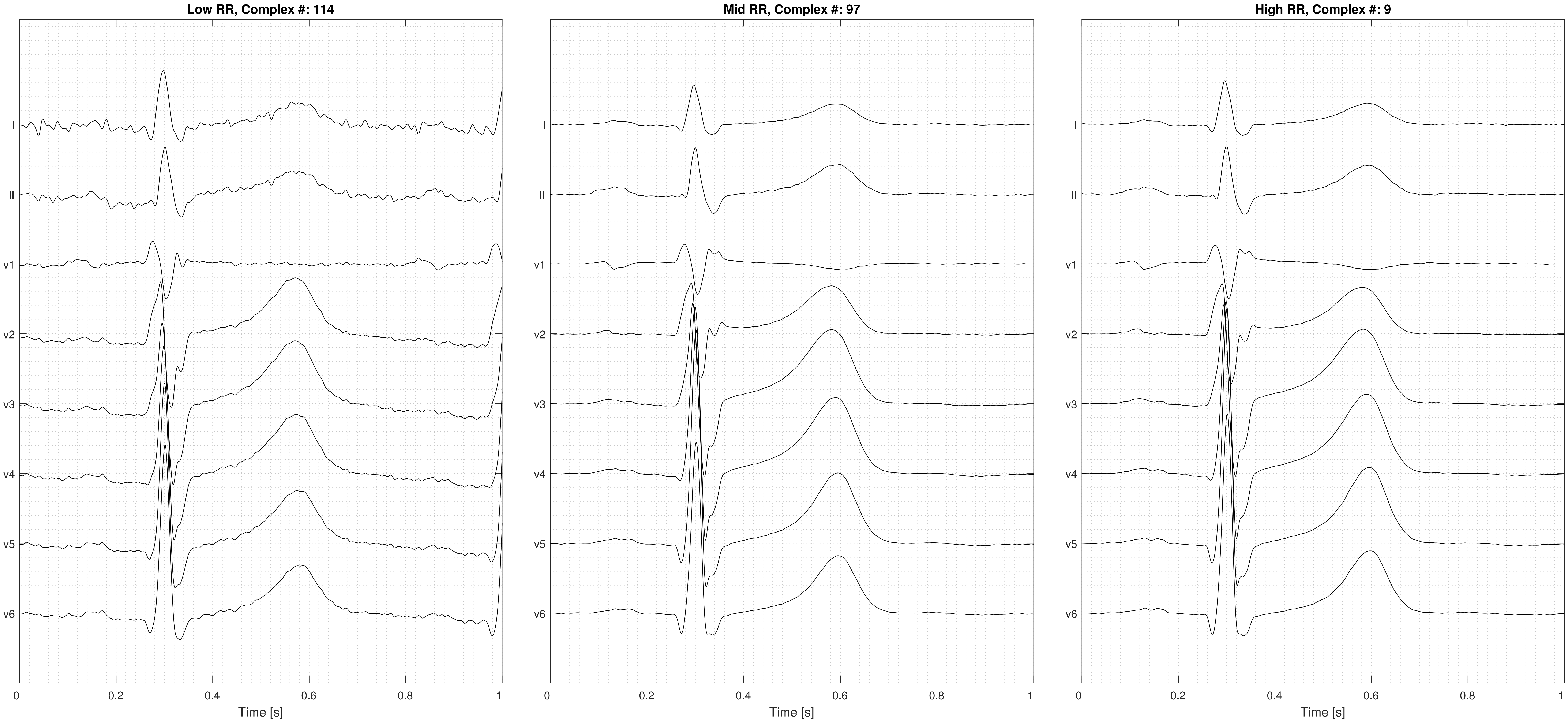

Low RR, Complex #: 114
Mid RR, Complex #: 97
High RR, Complex #: 9
I
II
v1
v2
v3
v4
v5
v6
0
0.2
0.4
0.6
0.8
1
Time [s]

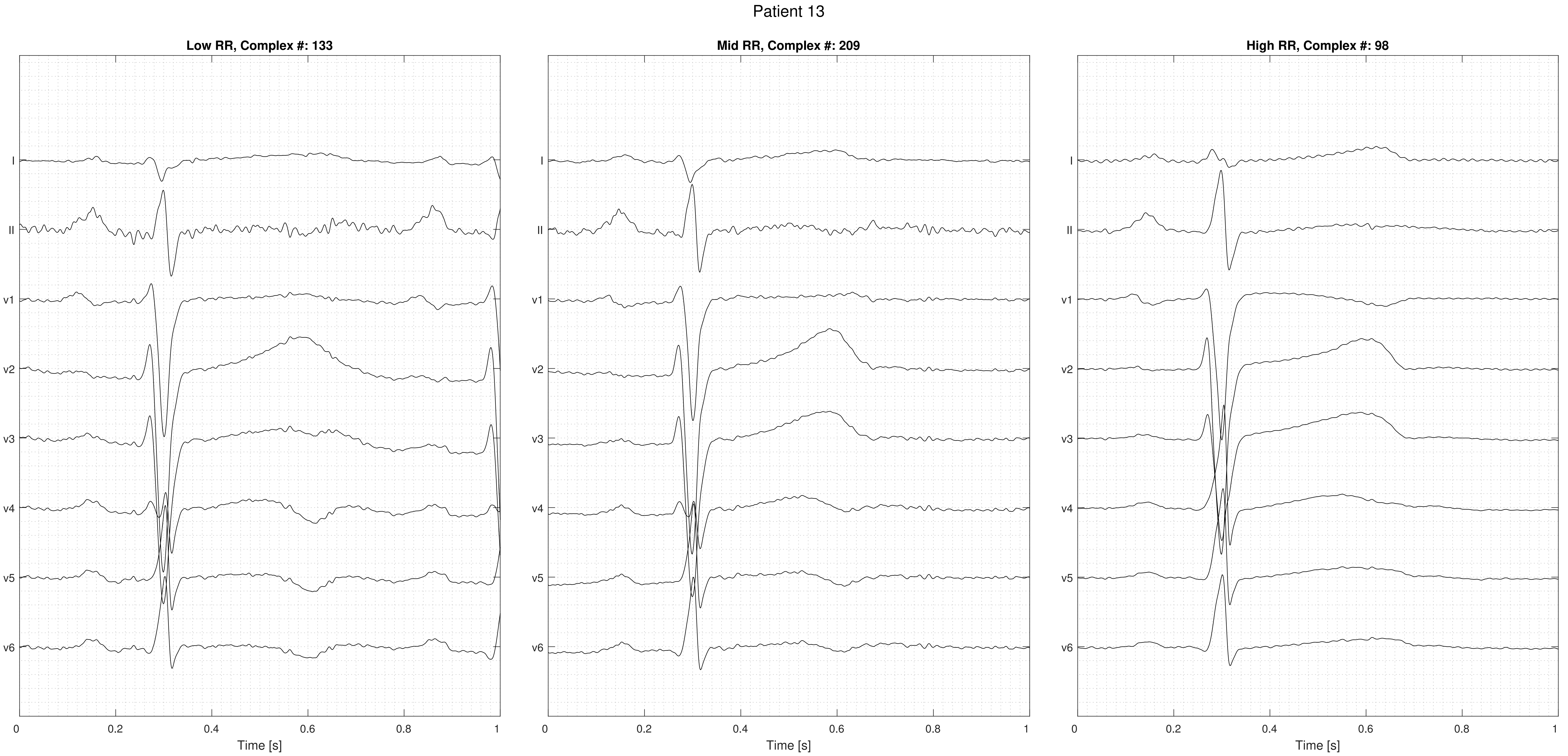
Patient 13
Low RR, Complex #: 133
Mid RR, Complex #: 209
High RR, Complex #: 98
I
II
v1
v2
v3
v4
v5
v6
0
0.2
0.4
0.6
0.8
1
Time [s]

Patient 14

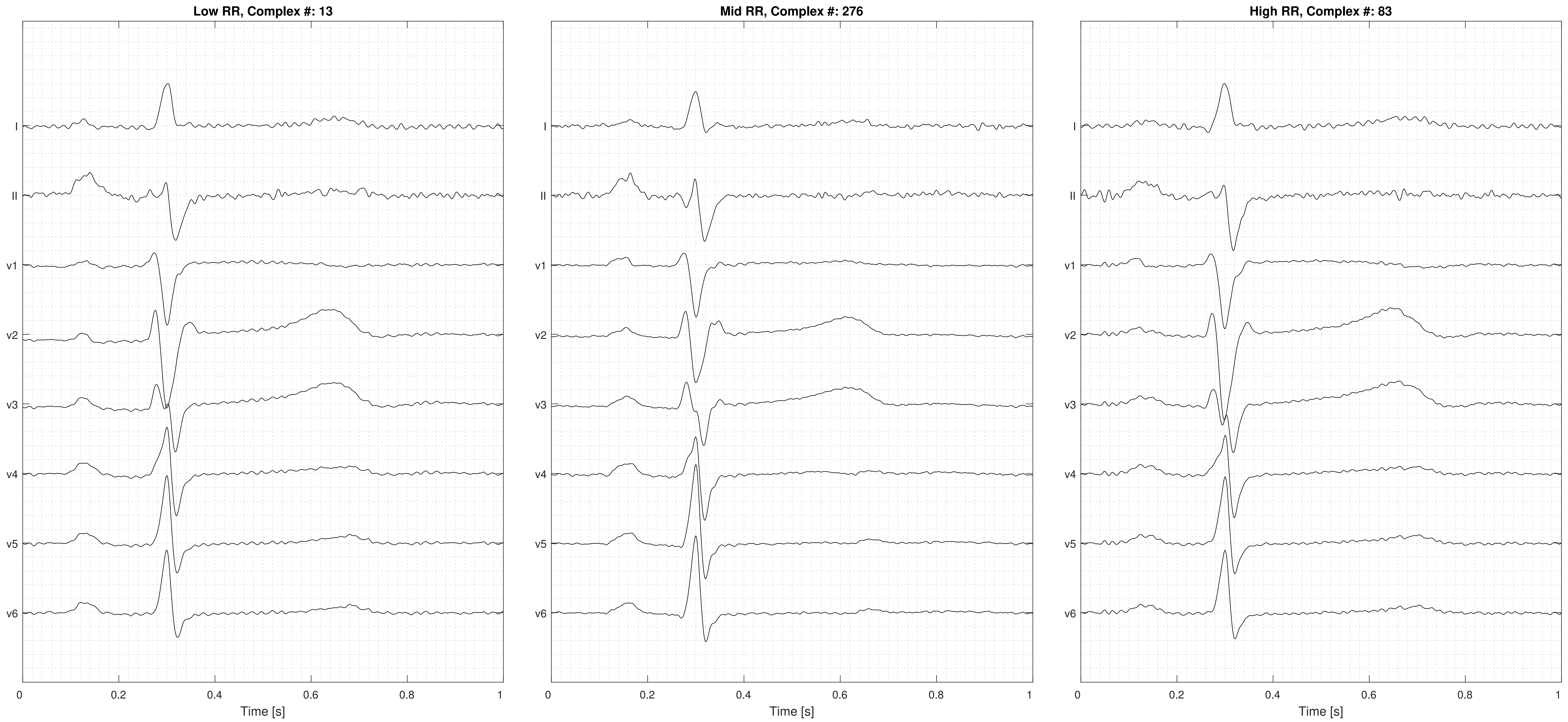

Low RR, Complex #: 13
Mid RR, Complex #: 276
High RR, Complex #: 83
I
II
v1
v2
v3
v4
v5
v6
0
0.2
0.4
0.6
0.8
1
Time [s]

Patient 15

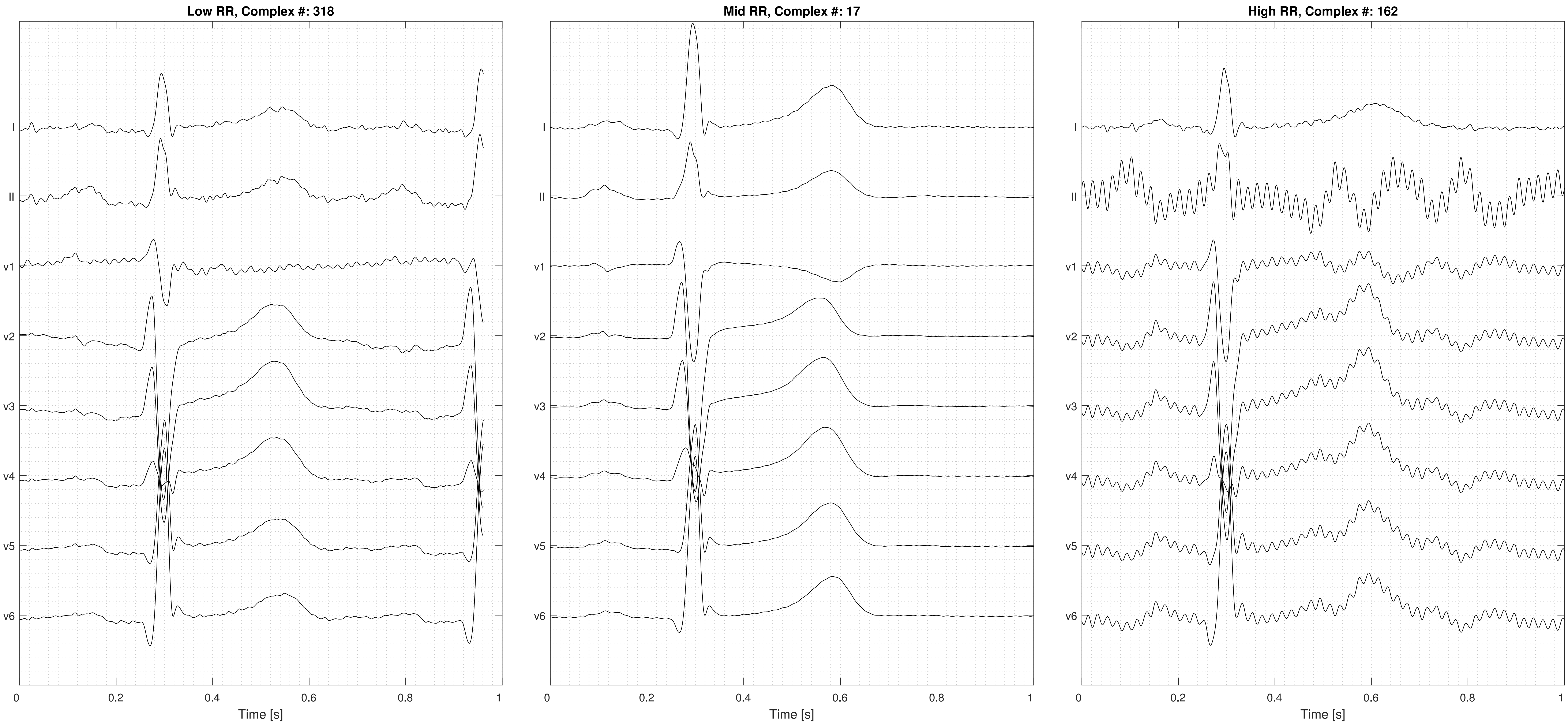

Low RR, Complex #: 318
Mid RR, Complex #: 17
High RR, Complex #: 162
I
II
v1
v2
v3
v4
v5
v6
0
0.2
0.4
0.6
0.8
1
Time [s]

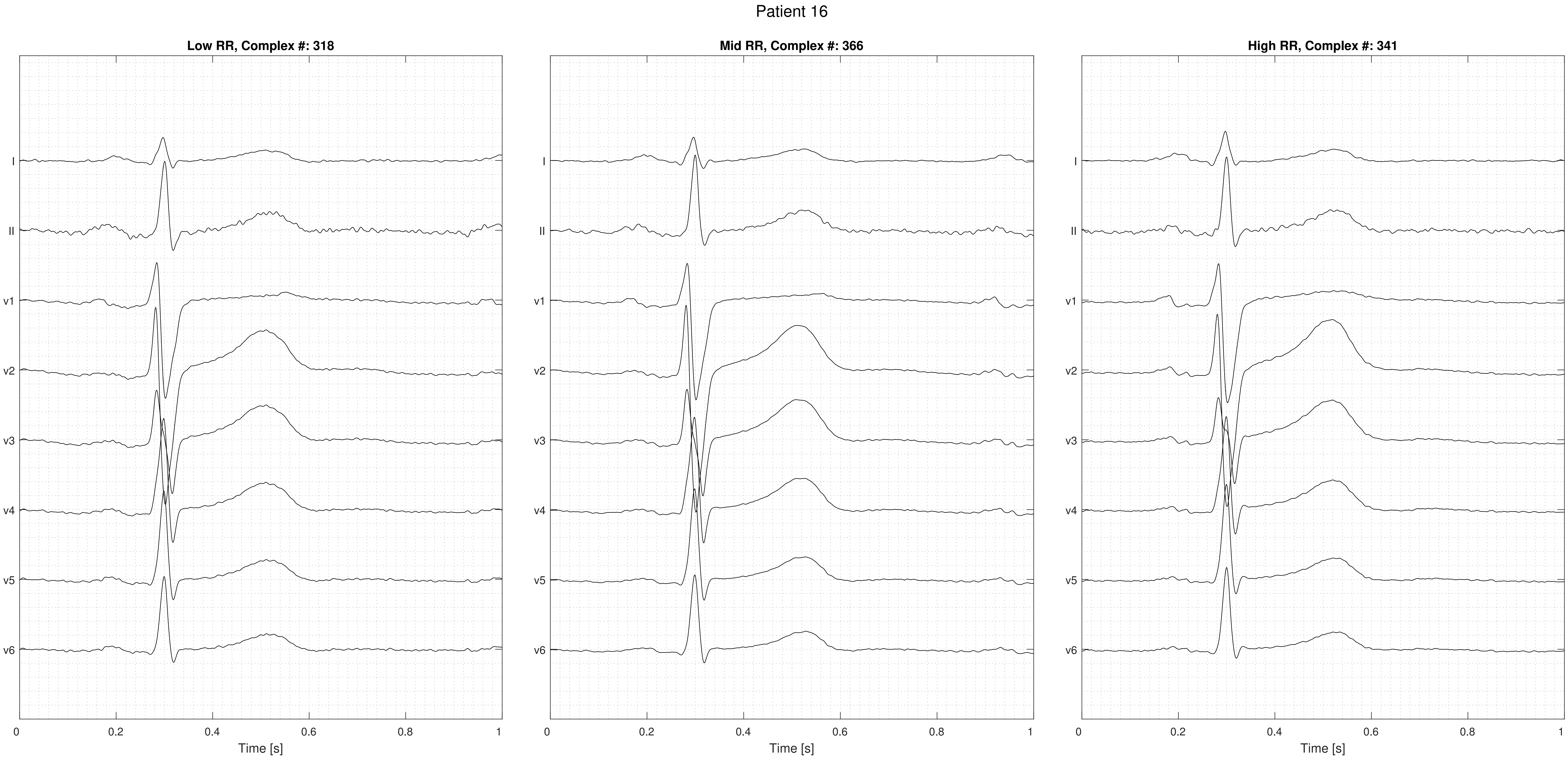

Patient 16
Low RR, Complex #: 318
Mid RR, Complex #: 366
High RR, Complex #: 341
I
II
v1
v2
v3
v4
v5
v6
0
0.2
0.4
0.6
0.8
1
Time [s]

Patient 17

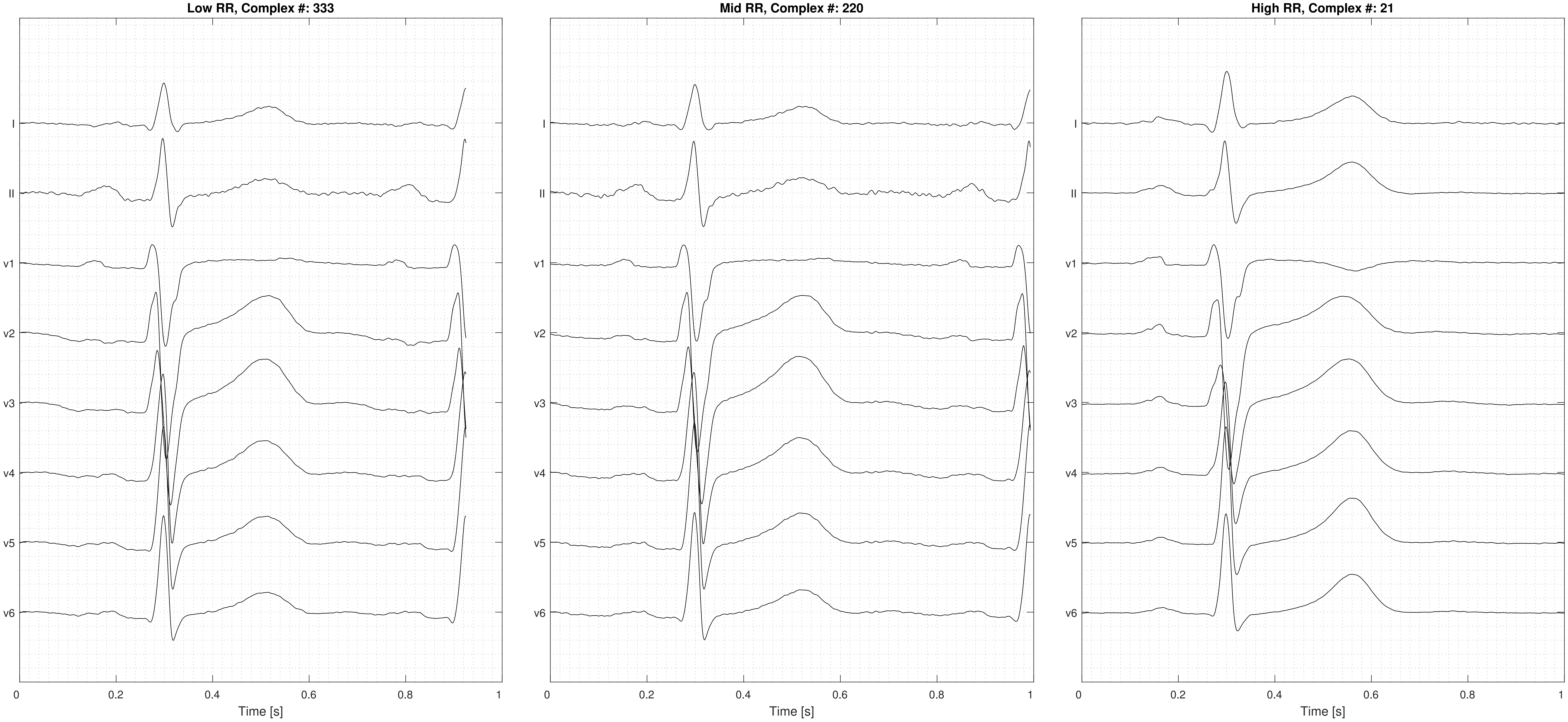

Low RR, Complex #: 333
Mid RR, Complex #: 220
High RR, Complex #: 21
I
II
v1
v2
v3
v4
v5
v6
0
0.2
0.4
0.6
0.8
1
Time [s]

Patient 18

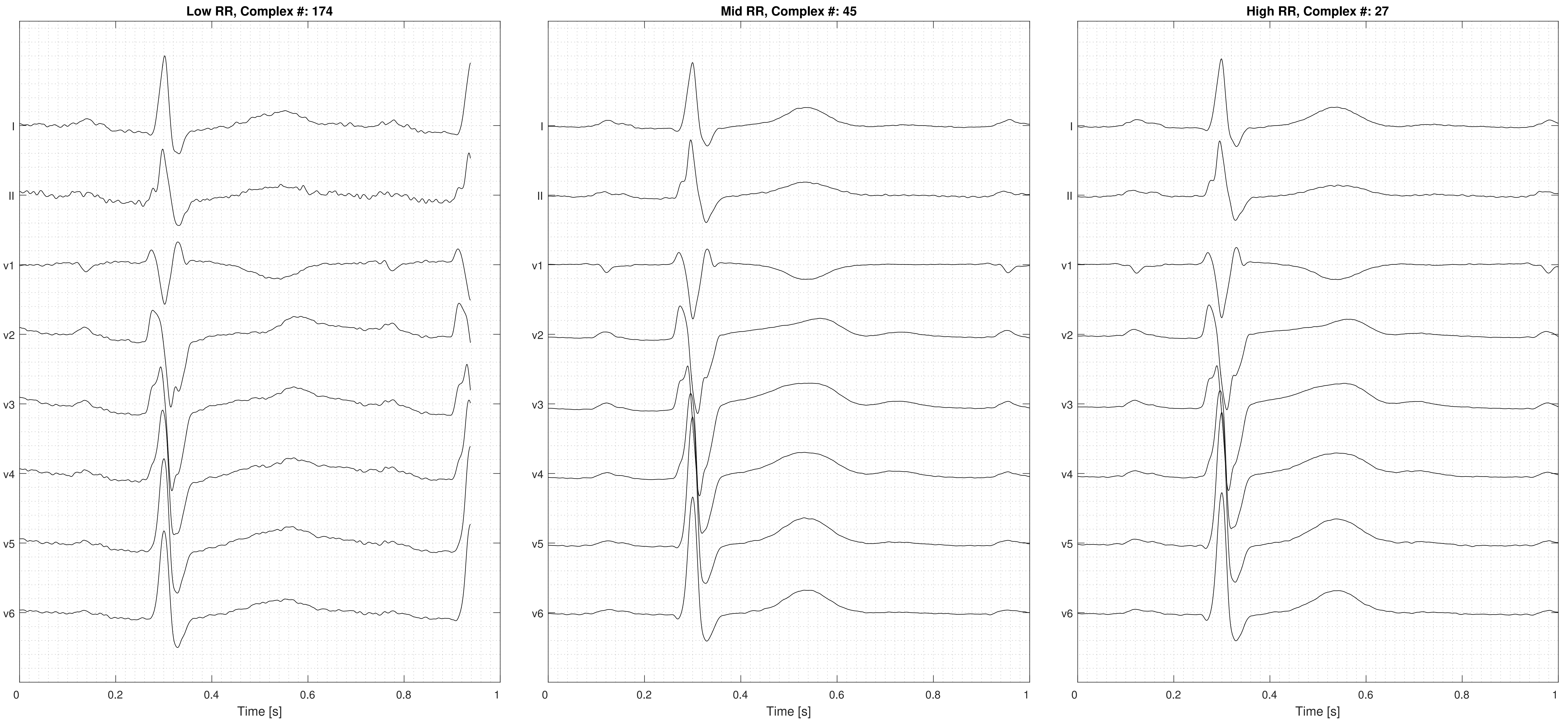

Low RR, Complex #: 174
Mid RR, Complex #: 45
High RR, Complex #: 27
I
II
v1
v2
v3
v4
v5
v6
0
0.2
0.4
0.6
0.8
1
Time [s]

Patient 19

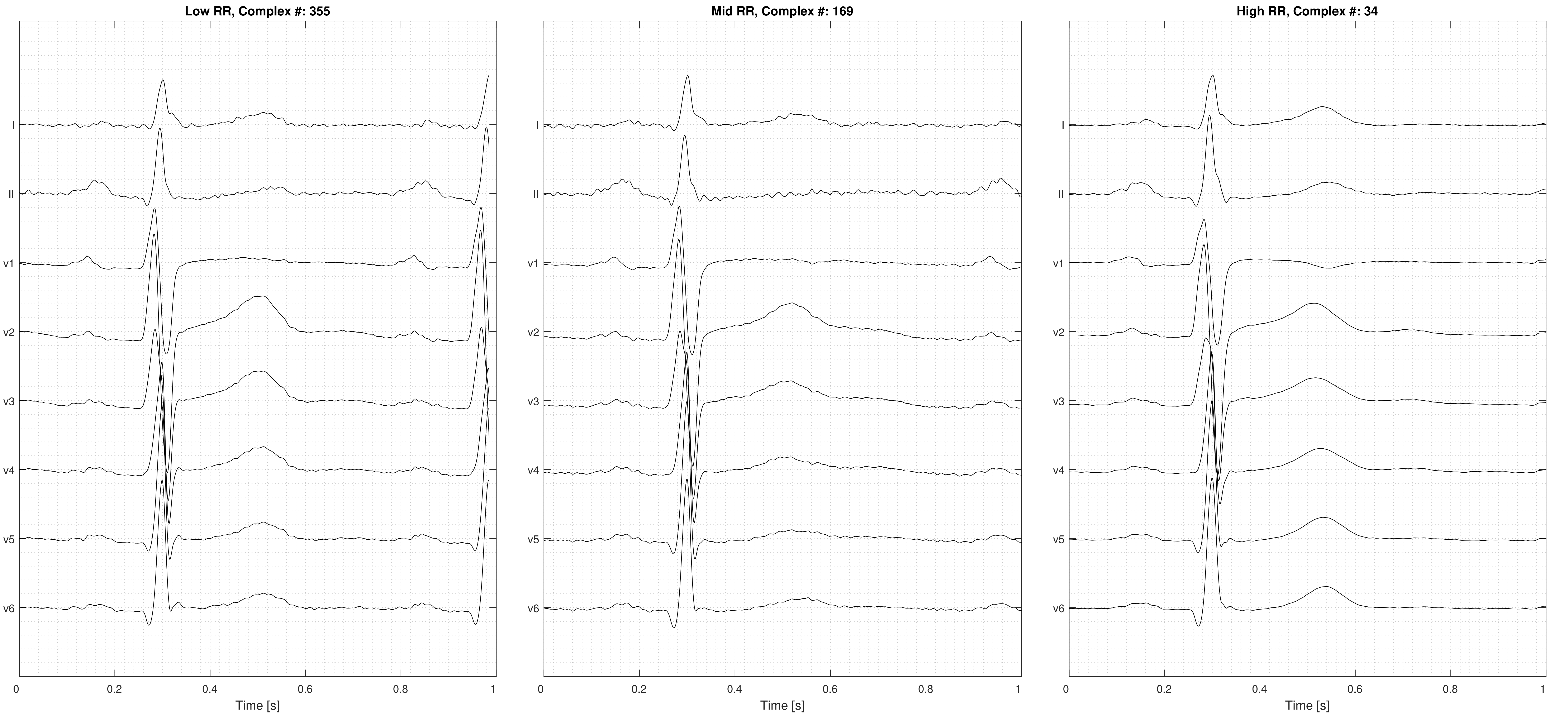

Low RR, Complex #: 355
Mid RR, Complex #: 169
High RR, Complex #: 34
I
II
v1
v2
v3
v4
v5
v6
0
0.2
0.4
0.6
0.8
1
Time [s]

Patient 20

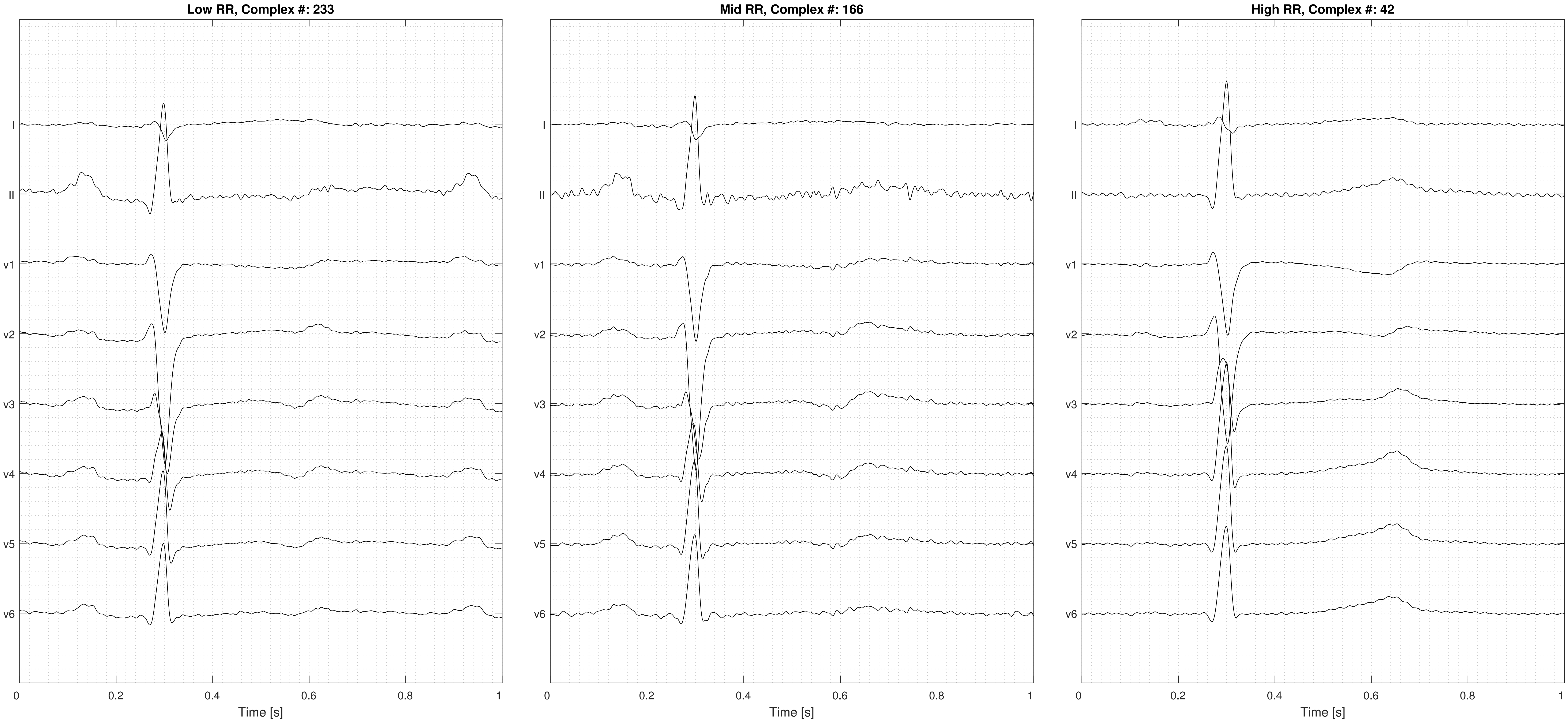

Low RR, Complex #: 233
Mid RR, Complex #: 166
High RR, Complex #: 42
I
II
v1
v2
v3
v4
v5
v6
0
0.2
0.4
0.6
0.8
1
Time [s]

Patient 21

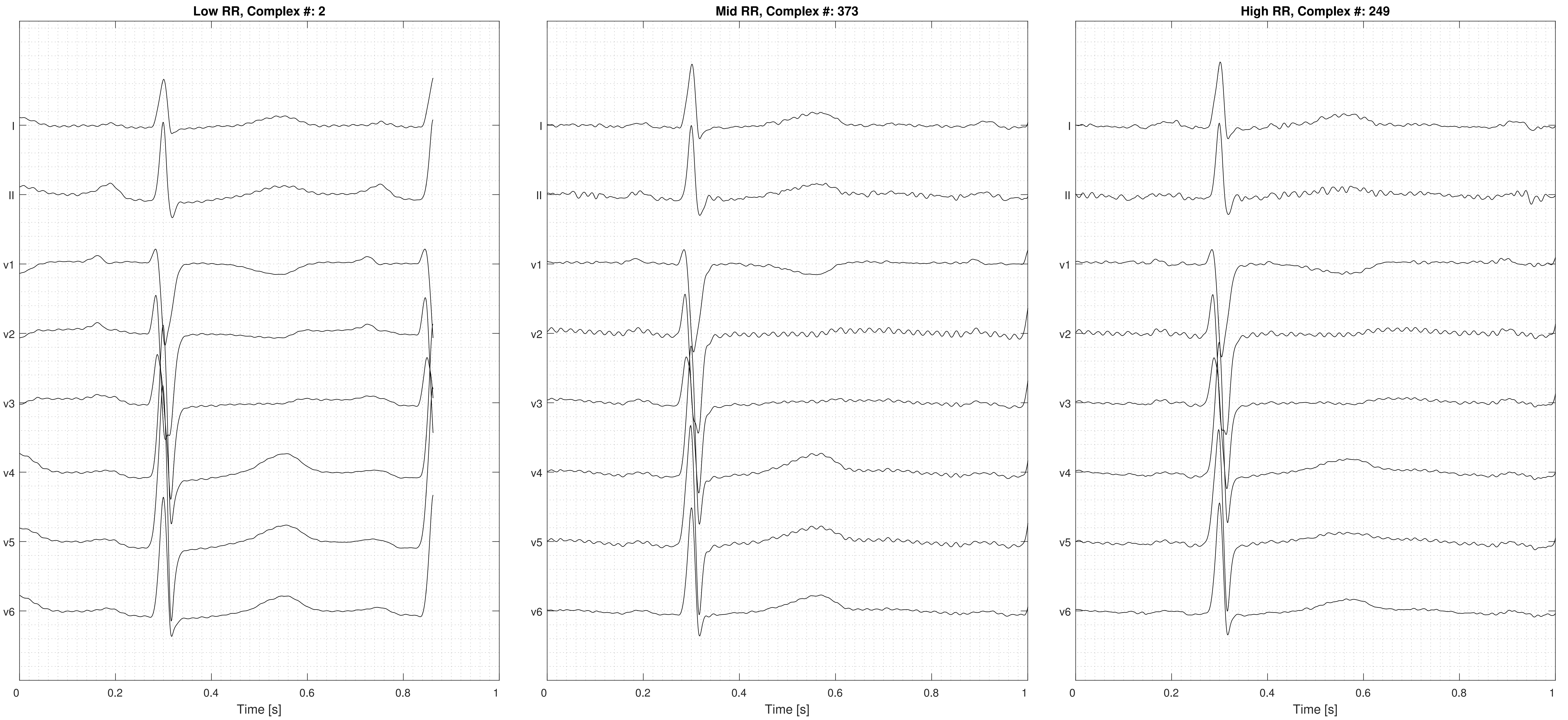

Low RR, Complex #: 2
Mid RR, Complex #: 373
High RR, Complex #: 249
I
II
v1
v2
v3
v4
v5
v6
0
0.2
0.4
0.6
0.8
1
Time [s]

Patient 22

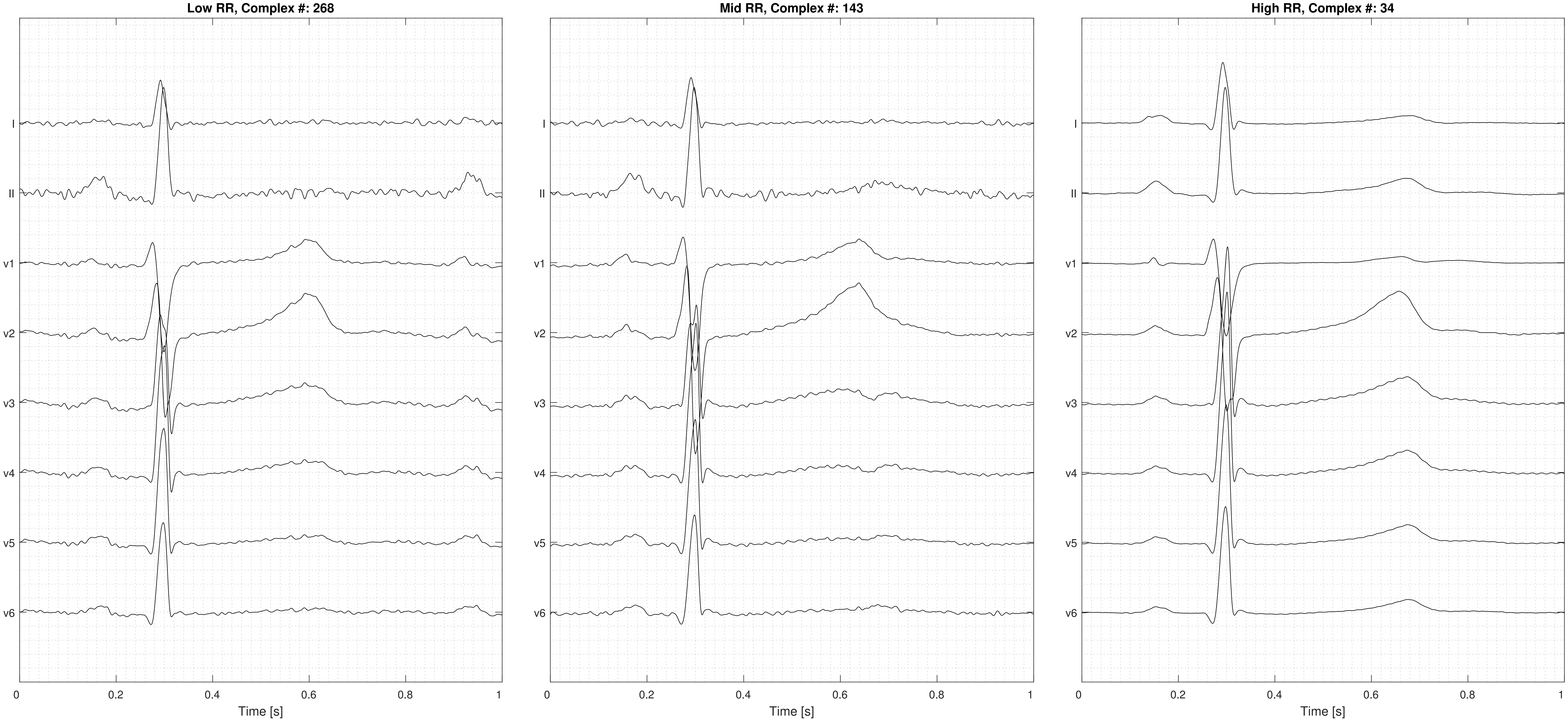

Low RR, Complex #: 268
Mid RR, Complex #: 143
High RR, Complex #: 34
I
II
v1
v2
v3
v4
v5
v6
0
0.2
0.4
0.6
0.8
1
Time [s]

Patient 23

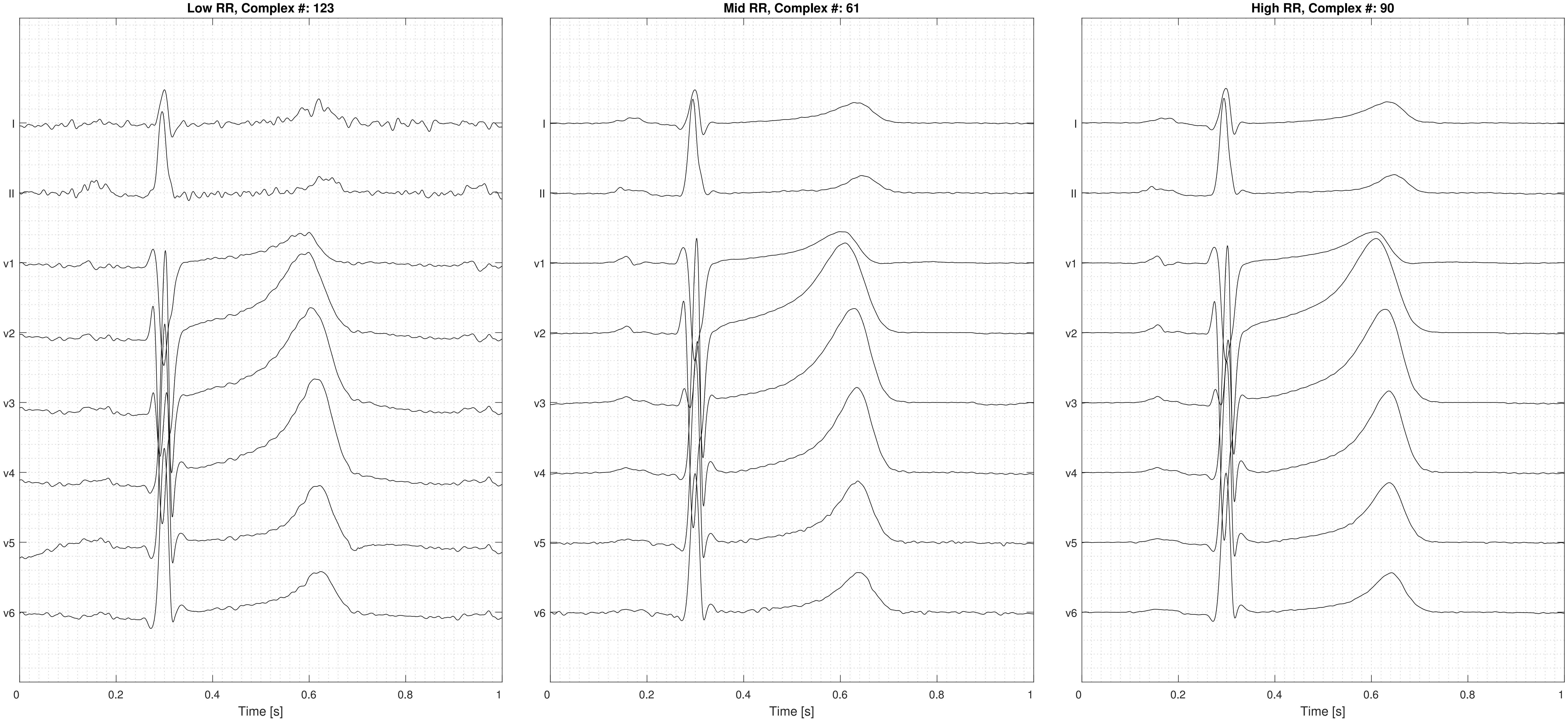

Low RR, Complex #: 123
Mid RR, Complex #: 61
High RR, Complex #: 90
I
II
v1
v2
v3
v4
v5
v6
0
0.2
0.4
0.6
0.8
1
Time [s]

Patient 24

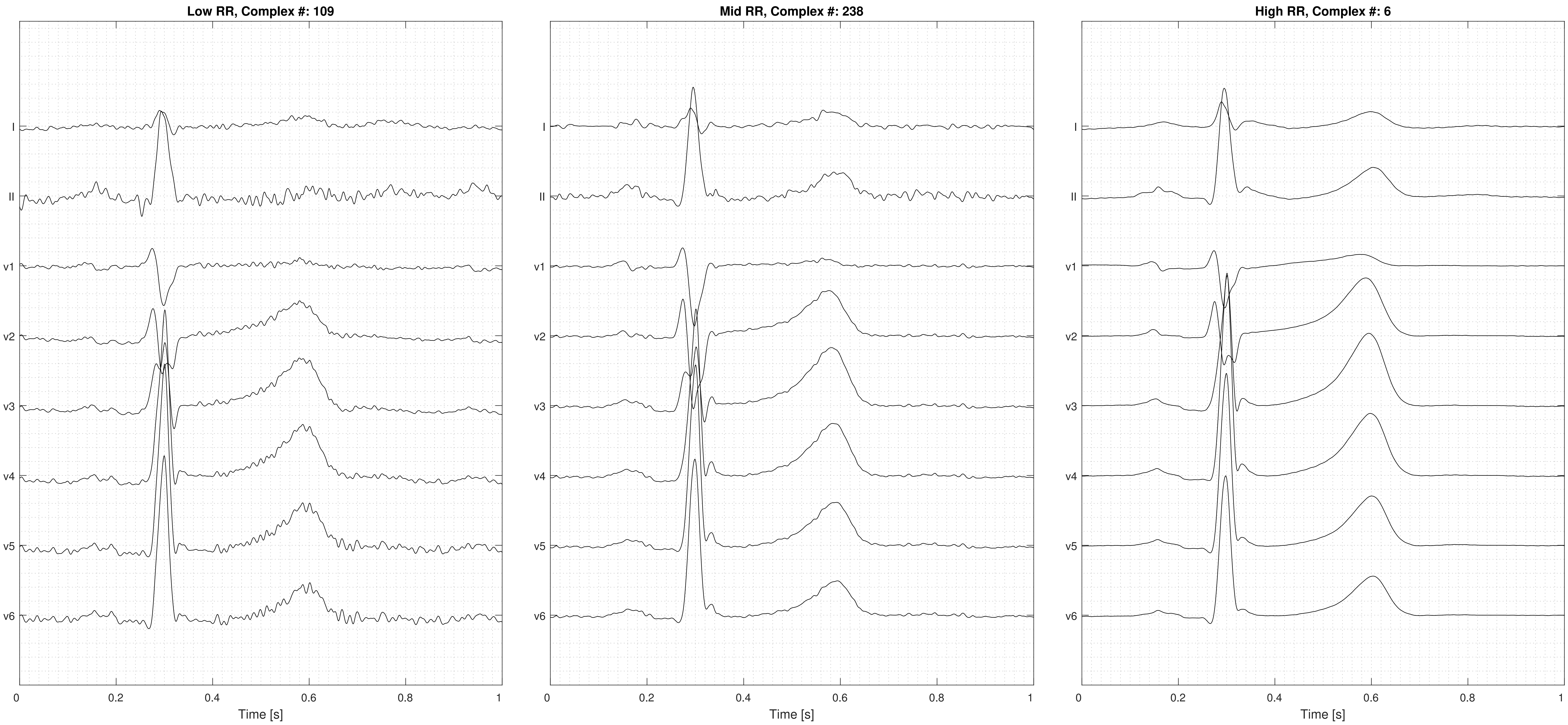

Low RR, Complex #: 109
Mid RR, Complex #: 238
High RR, Complex #: 6
I
II
v1
v2
v3
v4
v5
v6
0
0.2
0.4
0.6
0.8
1
Time [s]

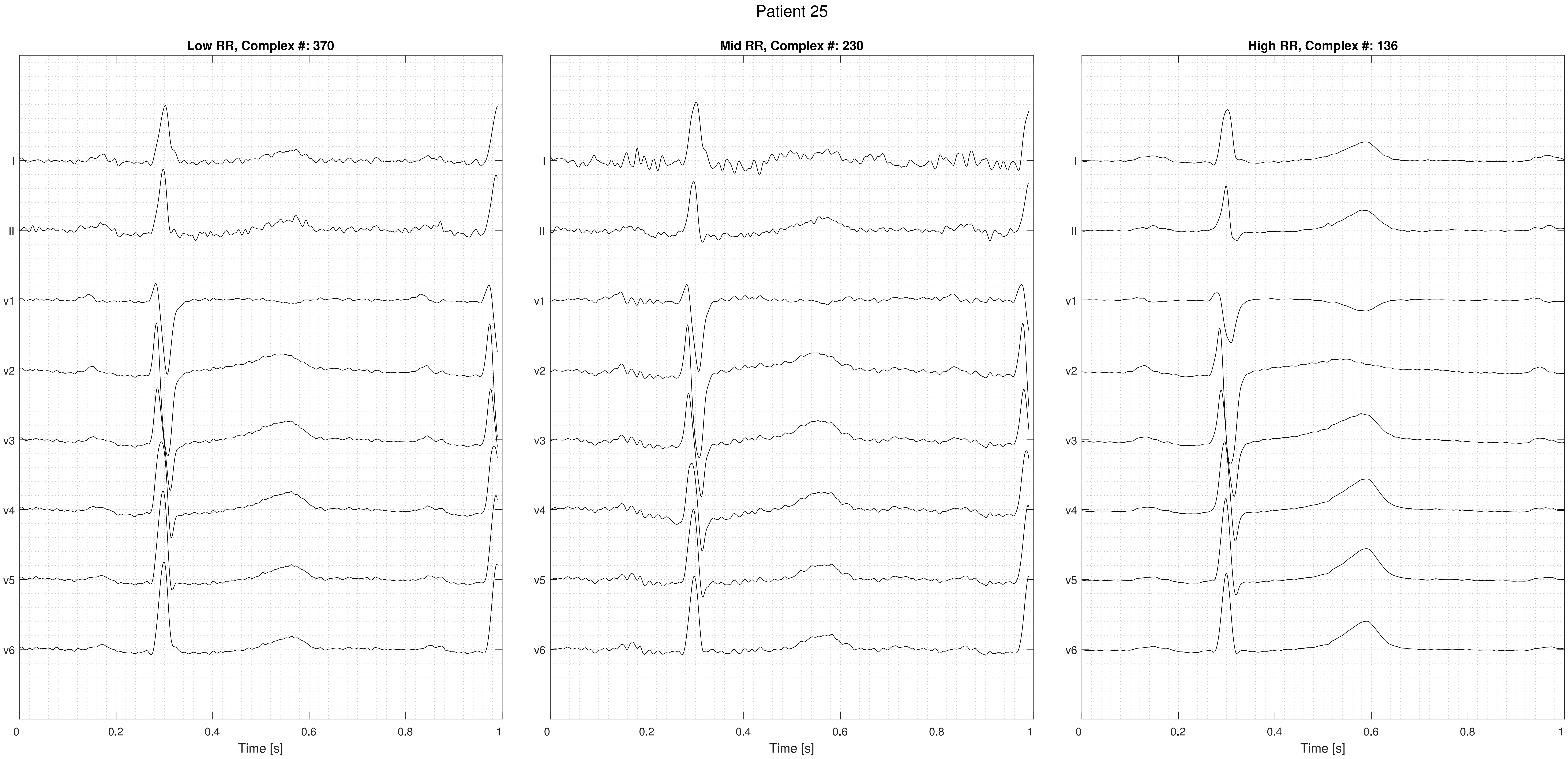

Patient 25
Low RR, Complex #: 370
Mid RR, Complex #: 230
High RR, Complex #: 136
I
II
v1
v2
v3
v4
v5
v6
0
0.2
0.4
0.6
0.8
1
Time [s]

Patient 26

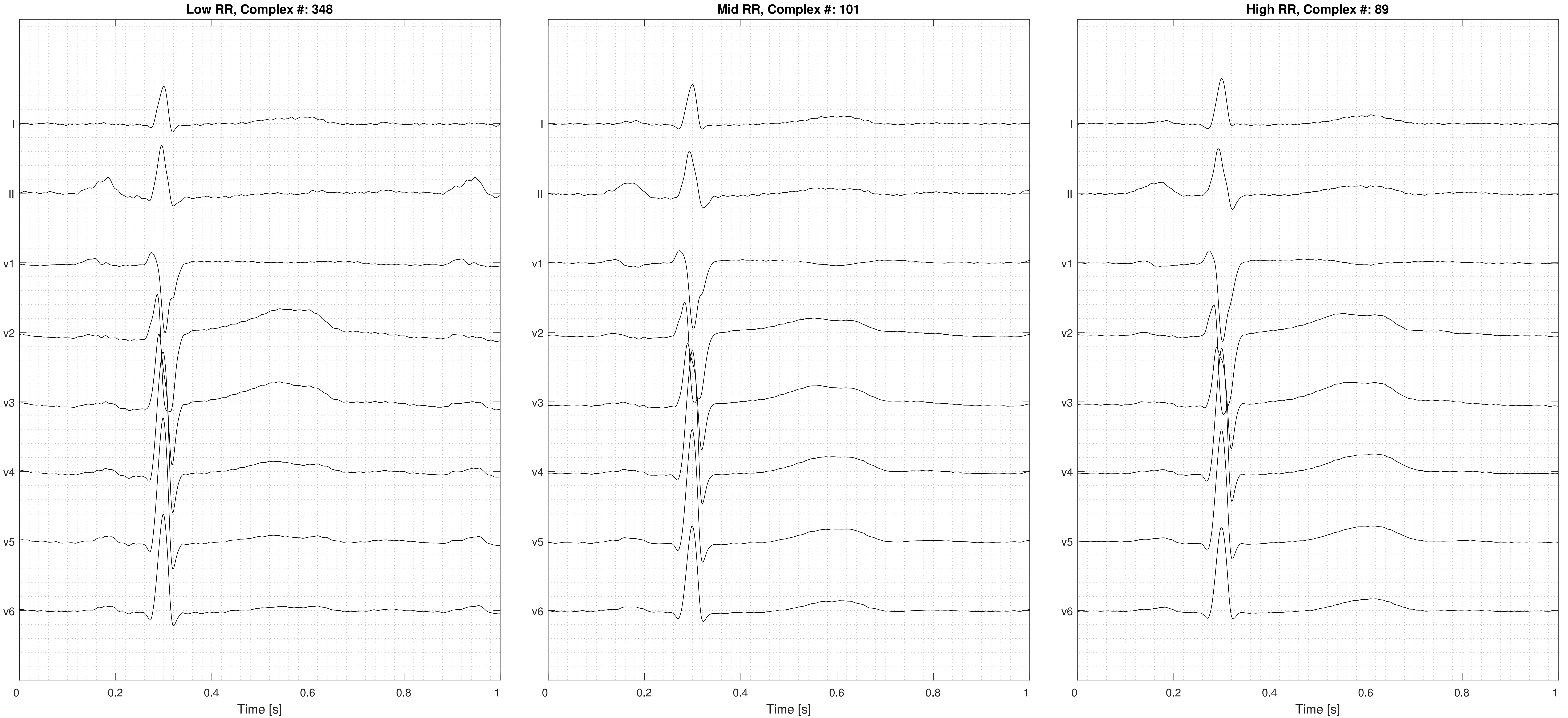

Low RR, Complex #: 348
Mid RR, Complex #: 101
High RR, Complex #: 89
I
II
v1
v2
v3
v4
v5
v6
0
0.2
0.4
0.6
0.8
1
Time [s]

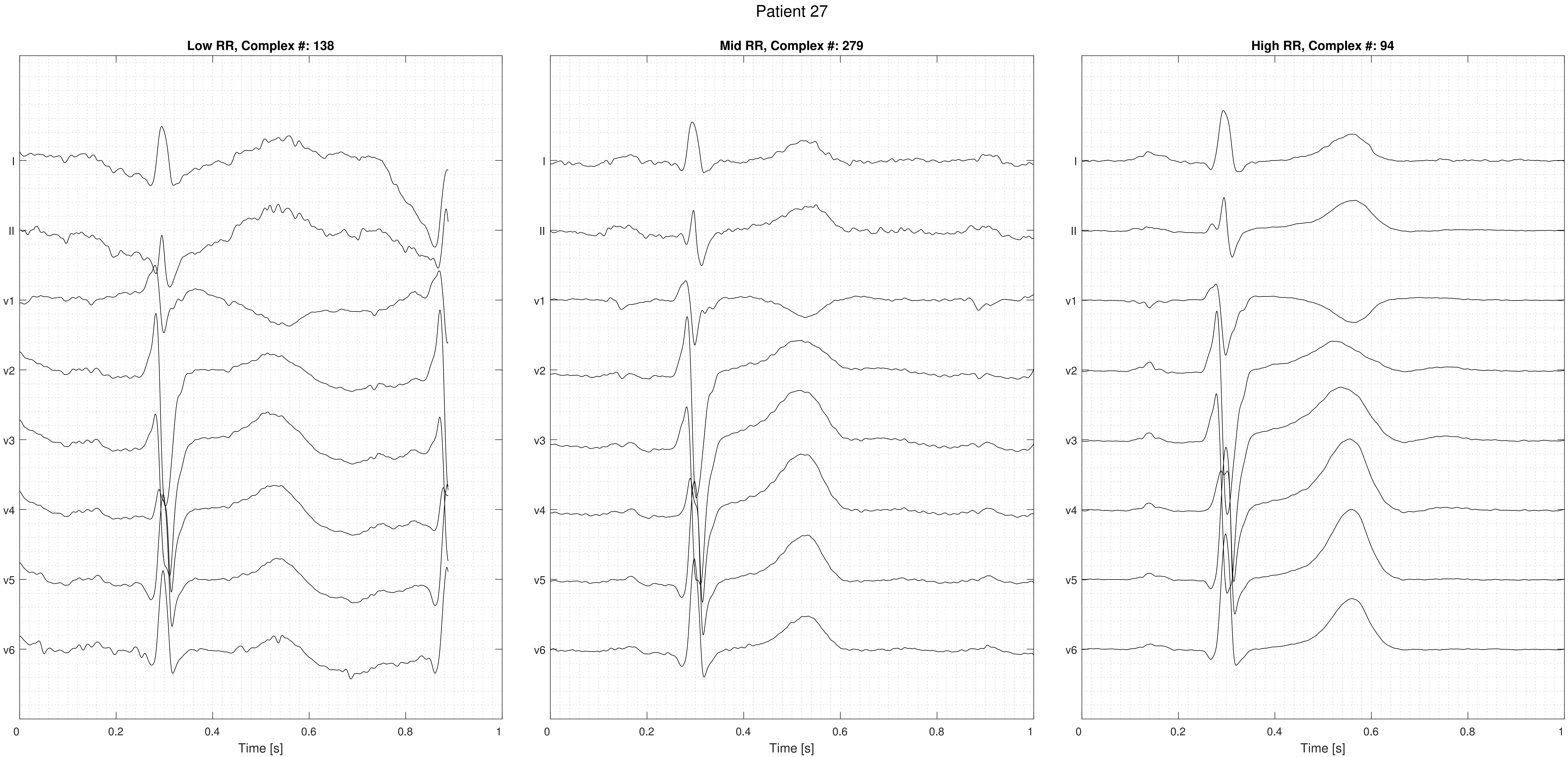
Patient 27
Low RR, Complex #: 138
Mid RR, Complex #: 279
High RR, Complex #: 94
I
II
v1
v2
v3
v4
v5
v6
0
0.2
0.4
0.6
0.8
1
Time [s]

Patient 28

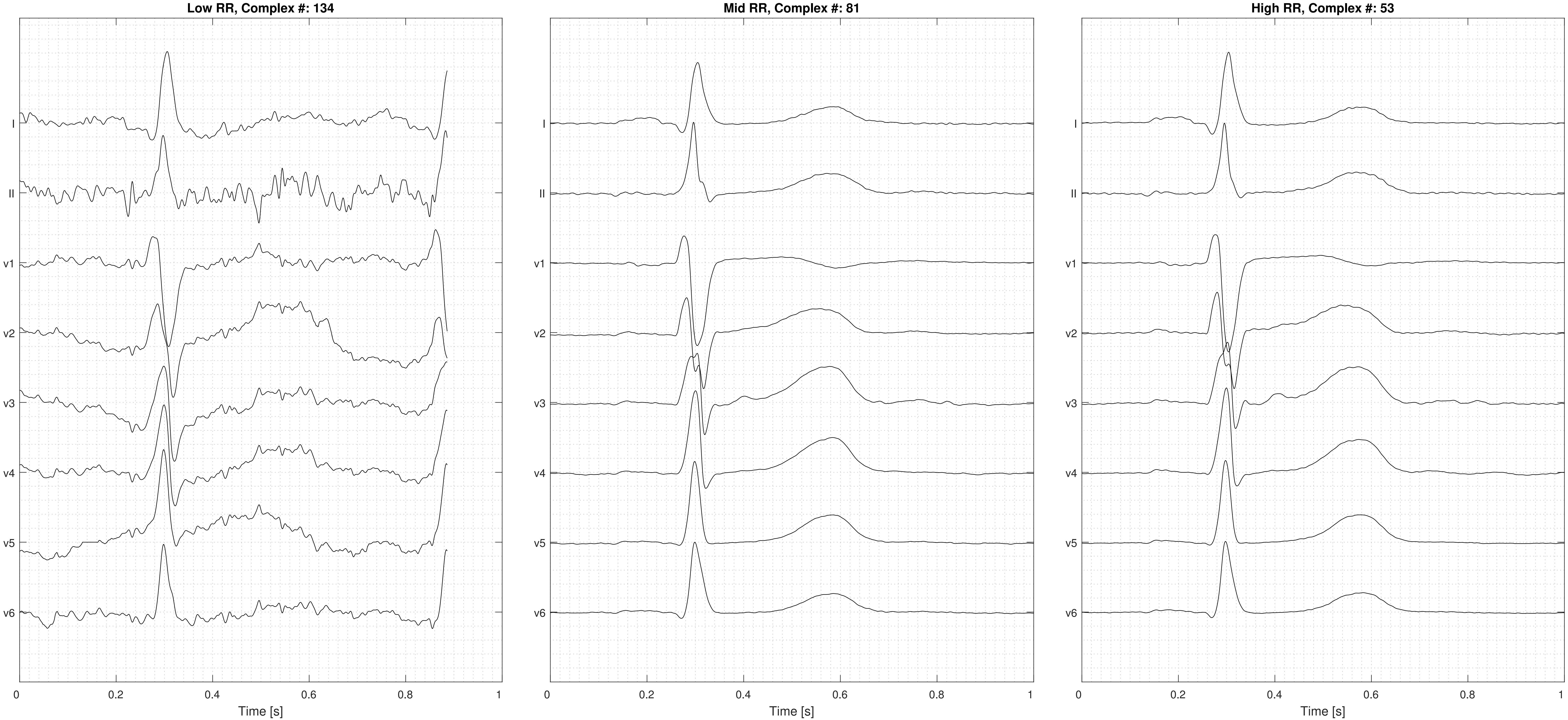

Low RR, Complex #: 134
Mid RR, Complex #: 81
High RR, Complex #: 53
I
II
v1
v2
v3
v4
v5
v6
0
0.2
0.4
0.6
0.8
1
Time [s]

Patient 29

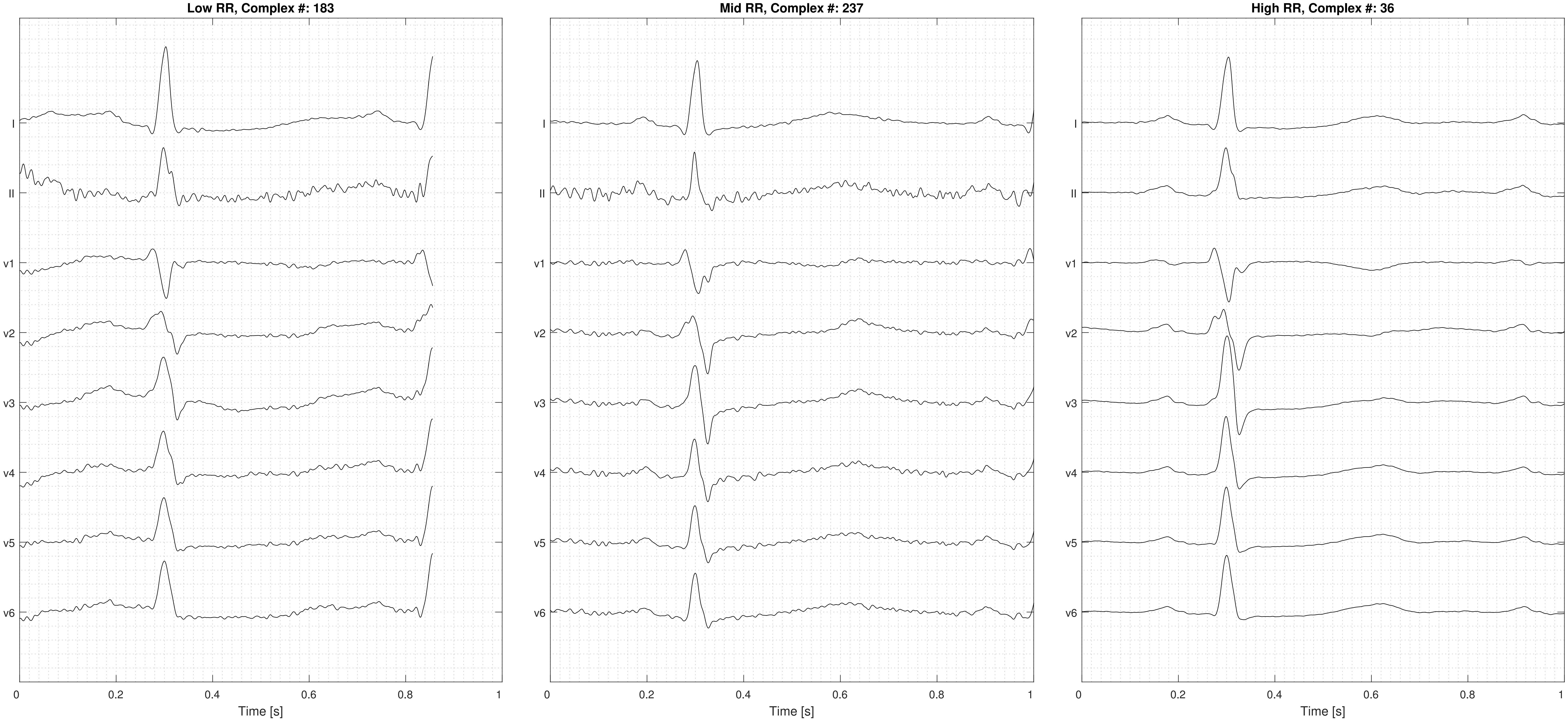

Low RR, Complex #: 183
Mid RR, Complex #: 237
High RR, Complex #: 36
I
II
v1
v2
v3
v4
v5
v6
0
0.2
0.4
0.6
0.8
1
Time [s]

Patient 30

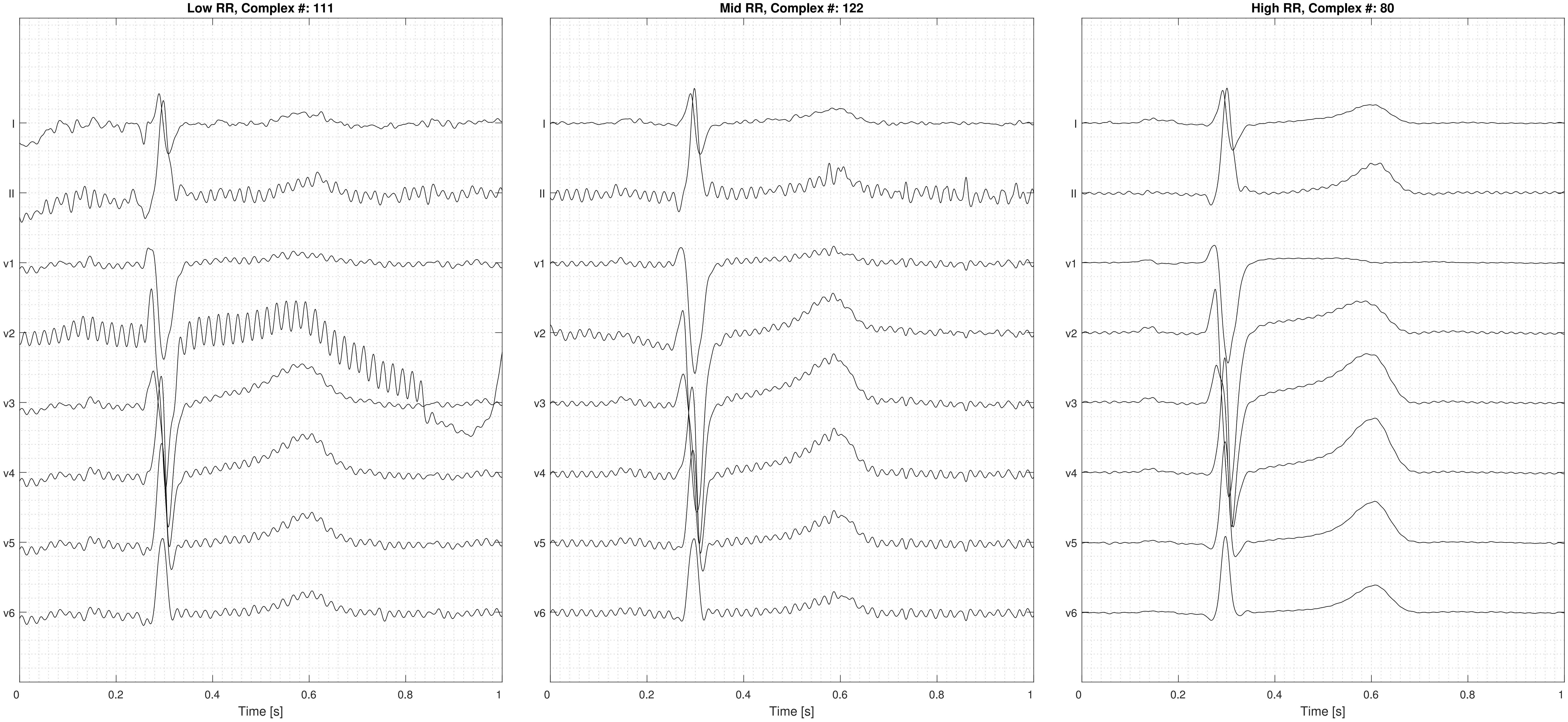

Low RR, Complex #: 111
Mid RR, Complex #: 122
High RR, Complex #: 80
I
II
v1
v2
v3
v4
v5
v6
0
0.2
0.4
0.6
0.8
1
Time [s]

Patient 31

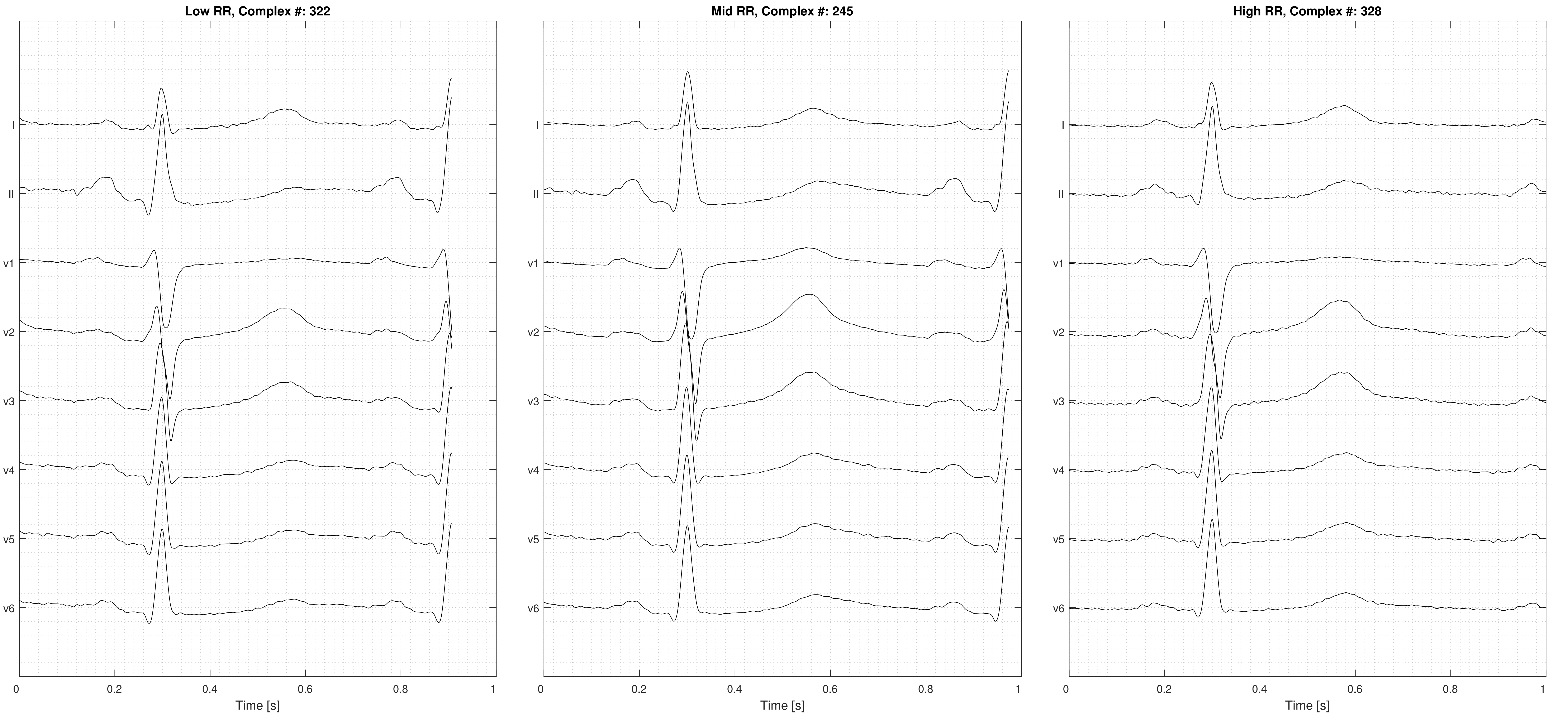

Low RR, Complex #: 322
Mid RR, Complex #: 245
High RR, Complex #: 328
I
II
v1
v2
v3
v4
v5
v6
0
0.2
0.4
0.6
0.8
1
Time [s]

Patient 32

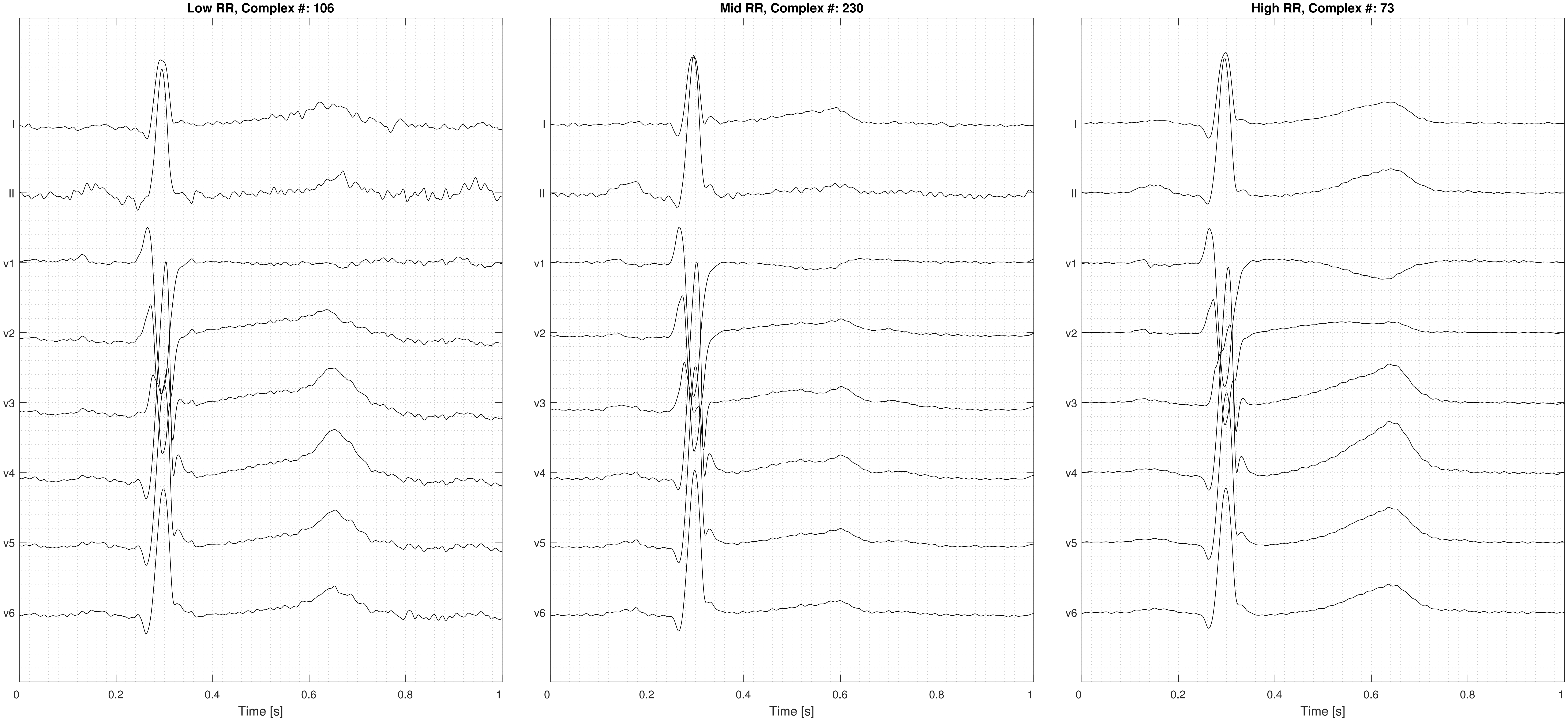

Low RR, Complex #: 106
Mid RR, Complex #: 230
High RR, Complex #: 73
I
II
v1
v2
v3
v4
v5
v6
0
0.2
0.4
0.6
0.8
1
Time [s]

Patient 33

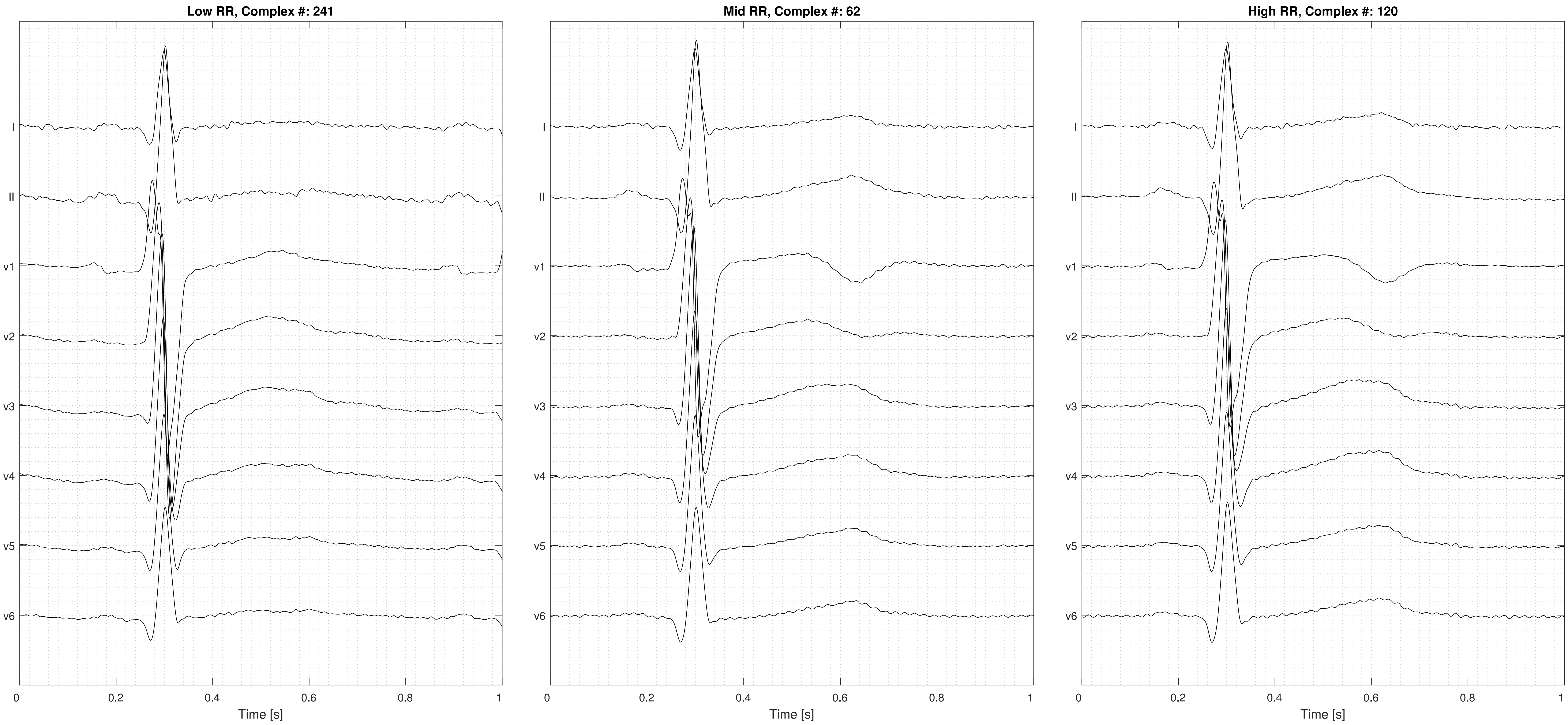

Low RR, Complex #: 241
Mid RR, Complex #: 62
High RR, Complex #: 120
I
II
v1
v2
v3
v4
v5
v6
0
0.2
0.4
0.6
0.8
1
Time [s]

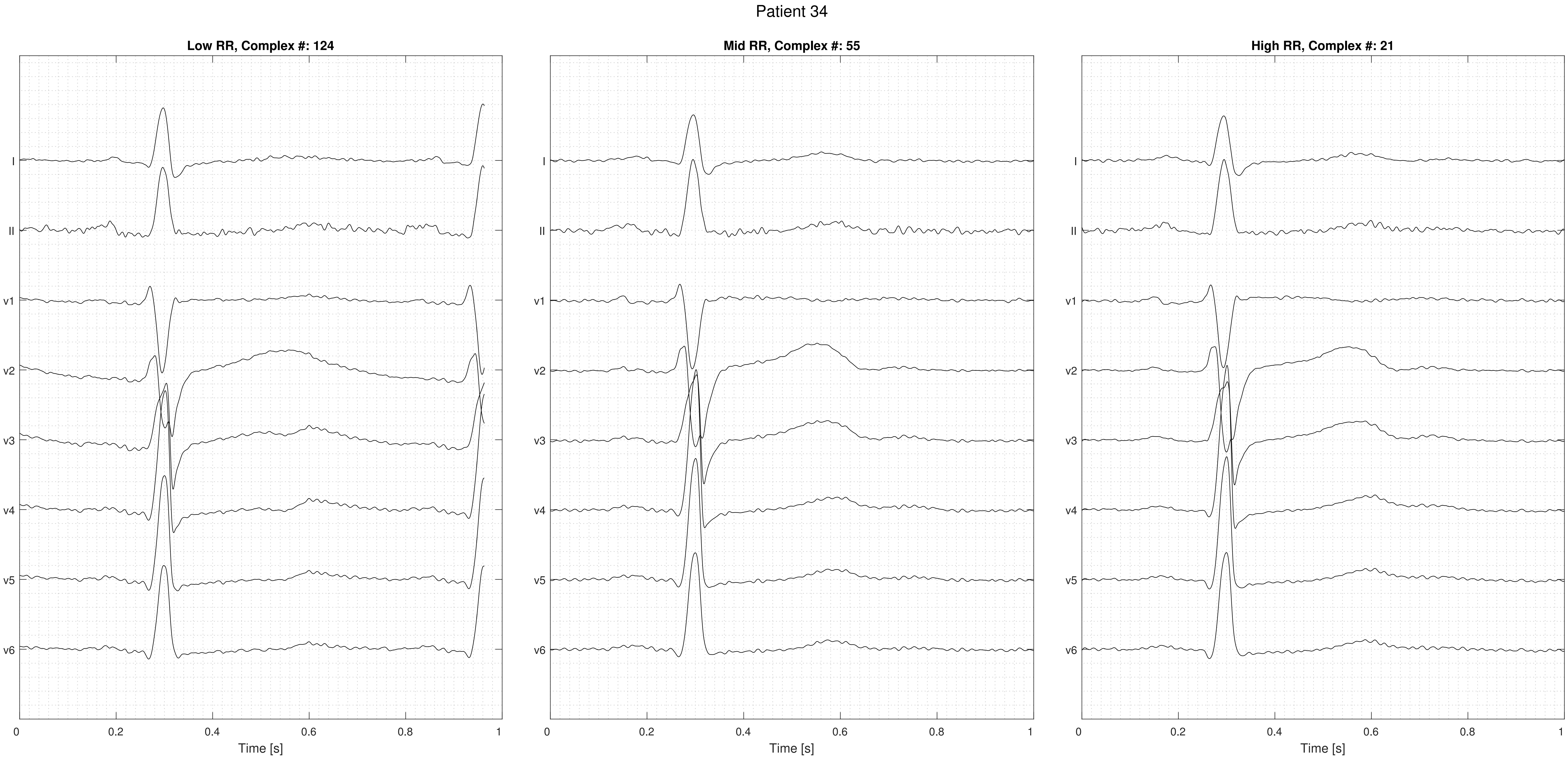

Patient 34
Low RR, Complex #: 124
Mid RR, Complex #: 55
High RR, Complex #: 21
I
II
v1
v2
v3
v4
v5
v6
0
0.2
0.4
0.6
0.8
1
Time [s]

Patient 35

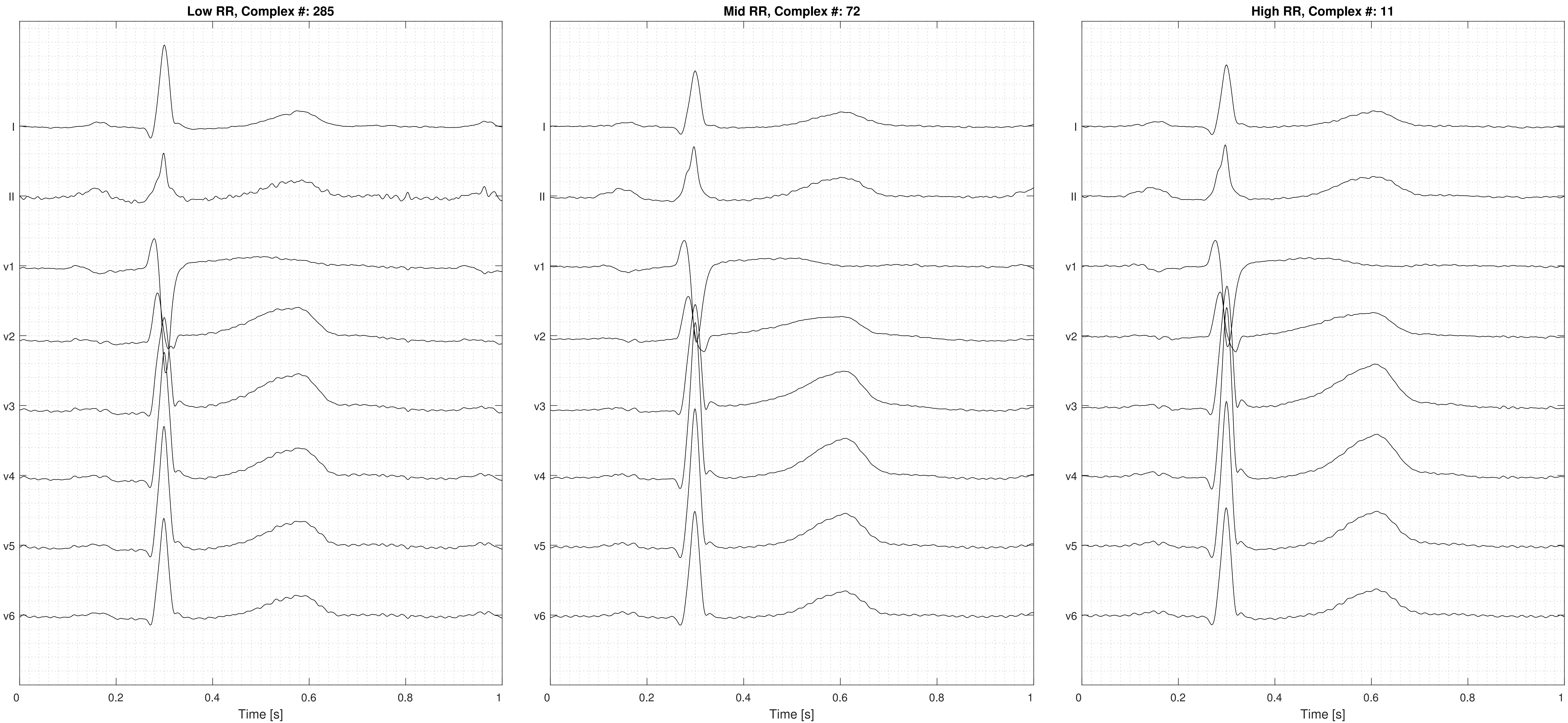
Low RR, Complex #: 285
Mid RR, Complex #: 72
High RR, Complex #: 11
I
II
v1
v2
v3
v4
v5
v6
0
0.2
0.4
0.6
0.8
1
Time [s]

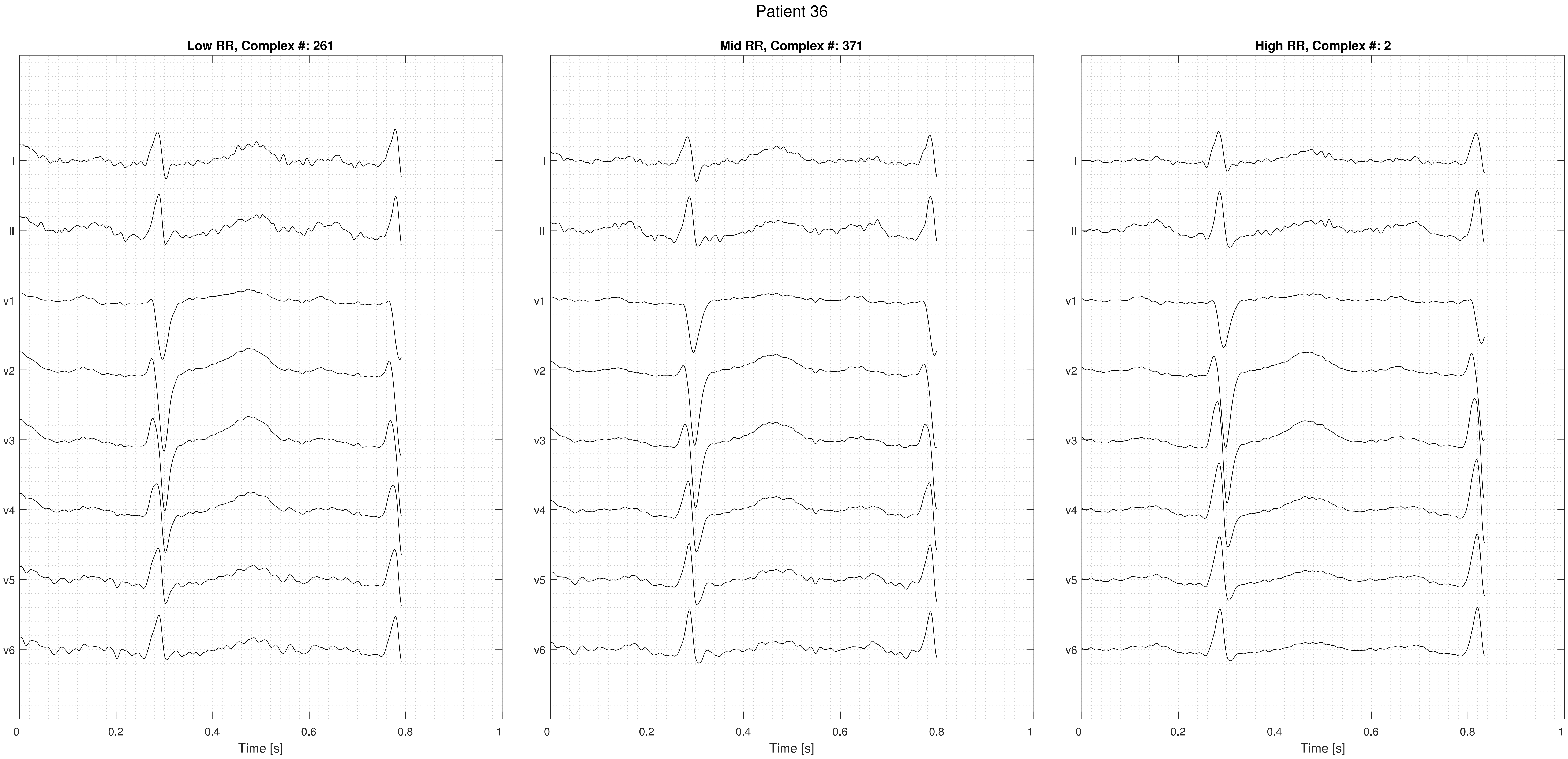
Patient 36
Low RR, Complex #: 261
Mid RR, Complex #: 371
High RR, Complex #: 2
I
II
v1
v2
v3
v4
v5
v6
0
0.2
0.4
0.6
0.8
1
Time [s]

Patient 37

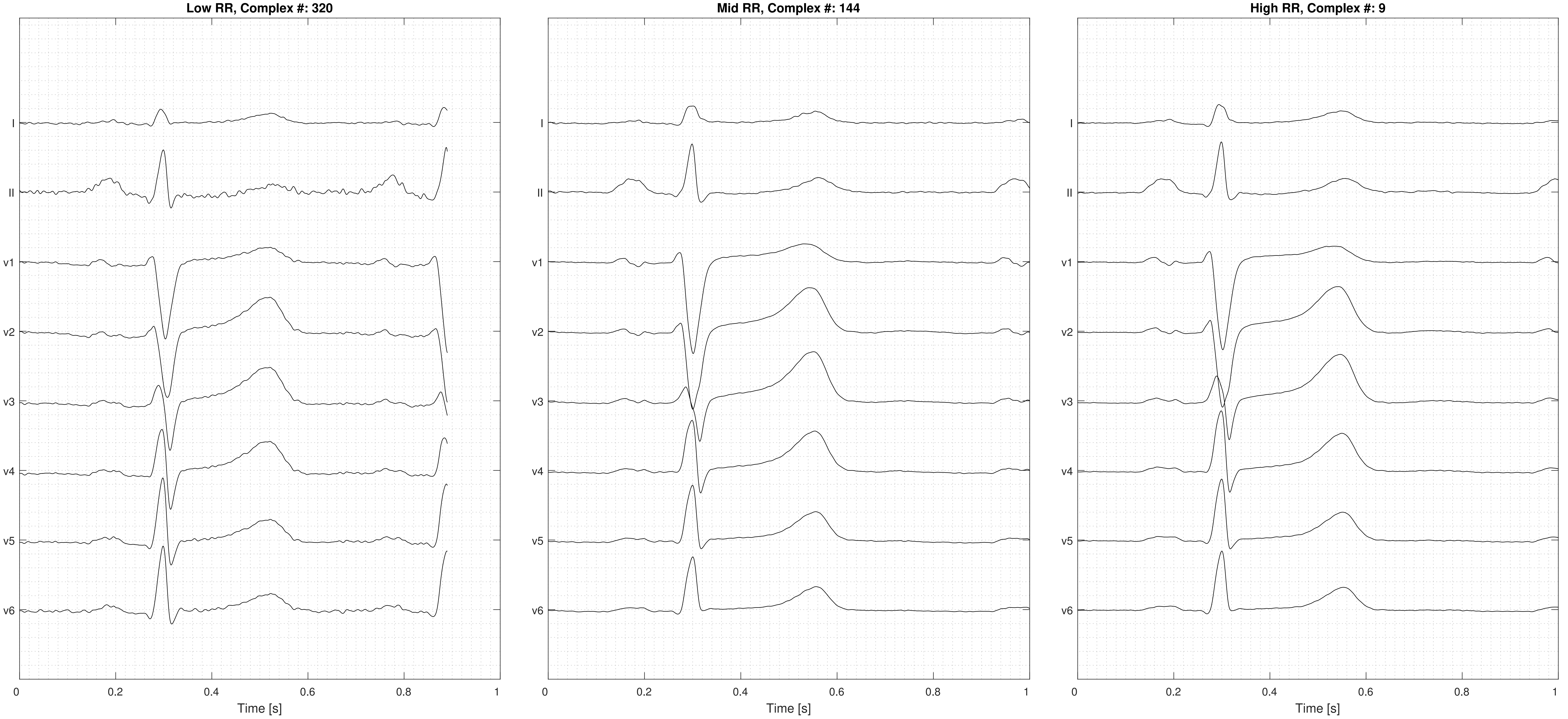

Low RR, Complex #: 320
Mid RR, Complex #: 144
High RR, Complex #: 9
I
II
v1
v2
v3
v4
v5
v6
0
0.2
0.4
0.6
0.8
1
Time [s]

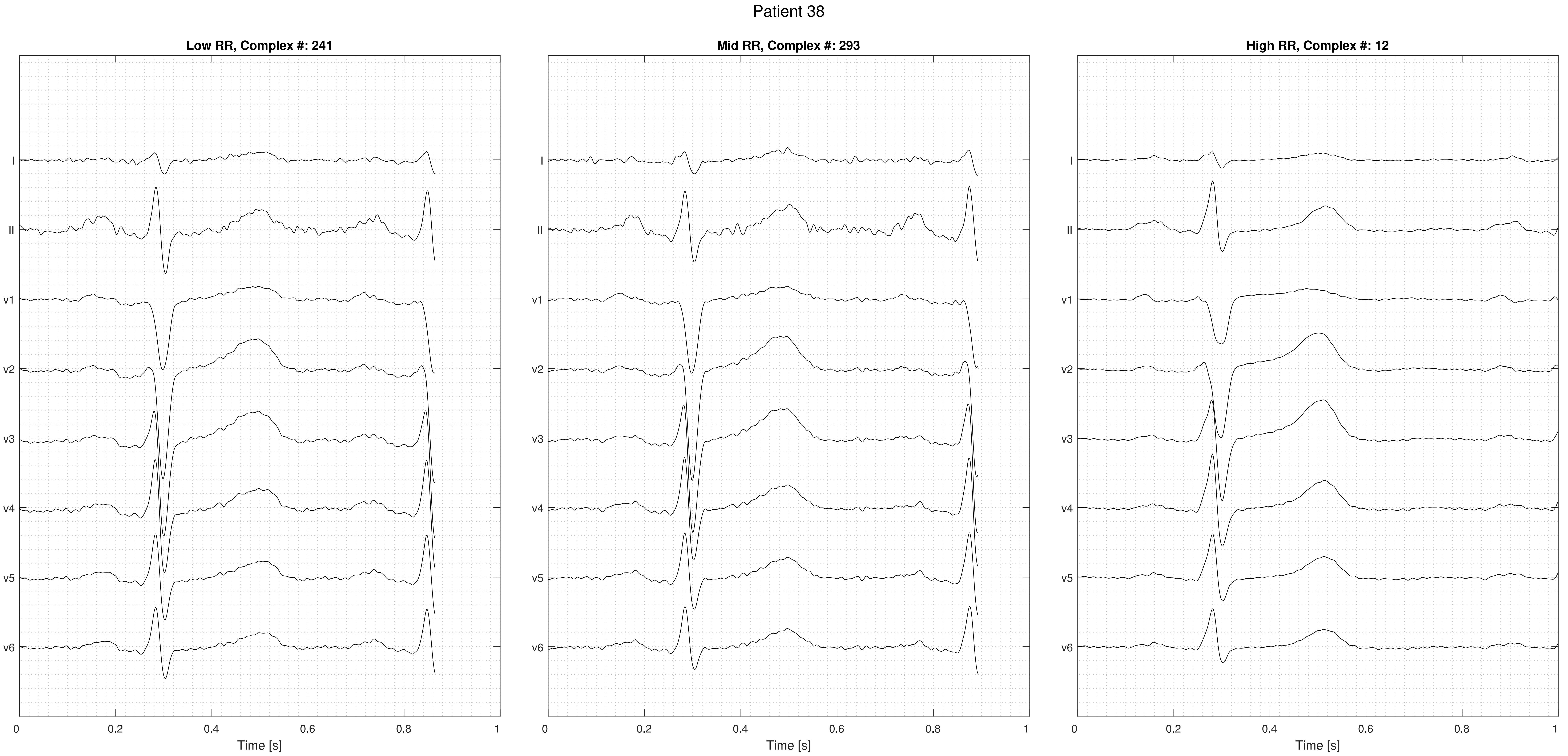

Patient 38
Low RR, Complex #: 241
Mid RR, Complex #: 293
High RR, Complex #: 12
I
II
v1
v2
v3
v4
v5
v6
0
0.2
0.4
0.6
0.8
1
Time [s]

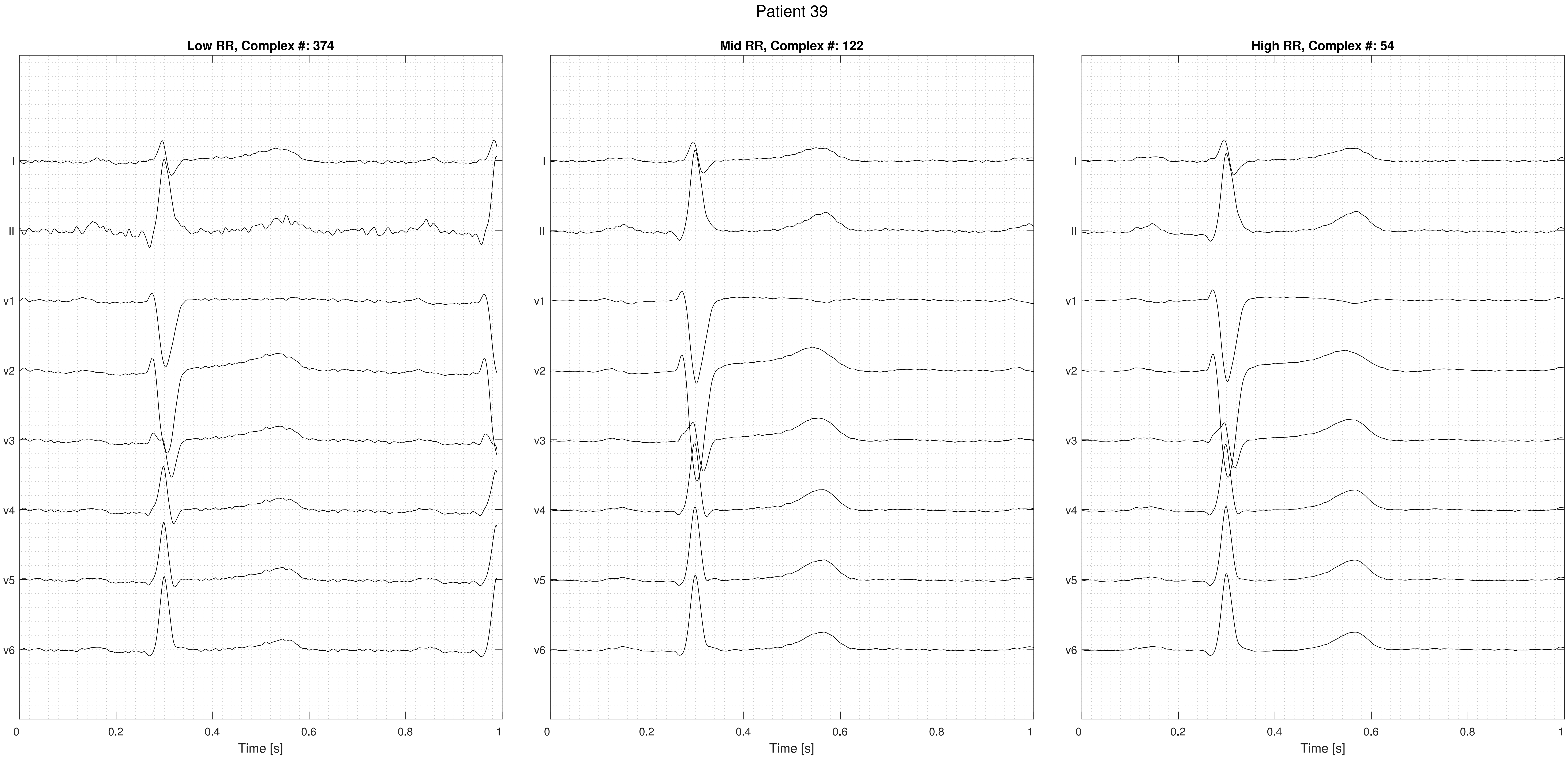
Patient 39
Low RR, Complex #: 374
Mid RR, Complex #: 122
High RR, Complex #: 54
I
II
v1
v2
v3
v4
v5
v6
0
0.2
0.4
0.6
0.8
1
Time [s]

Patient 40

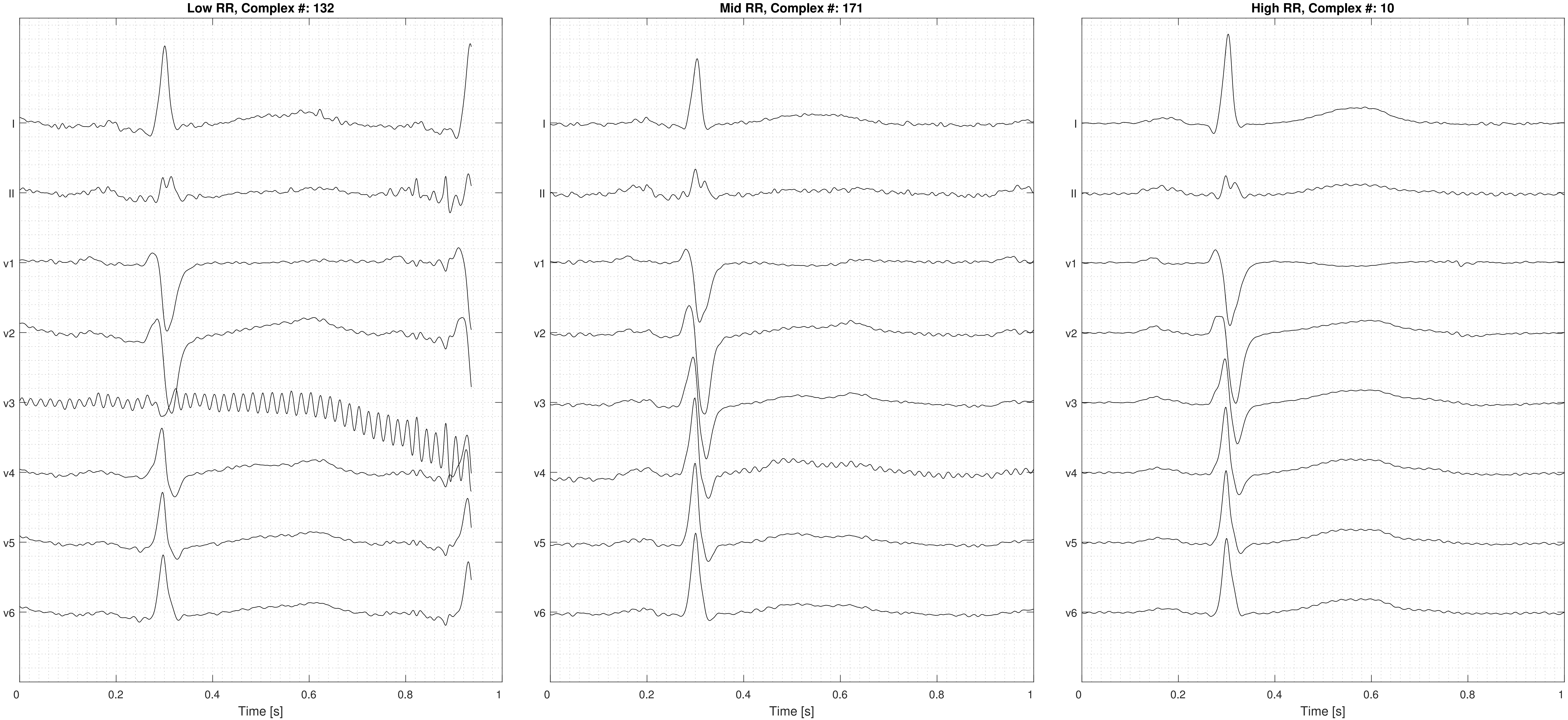

Low RR, Complex #: 132
Mid RR, Complex #: 171
High RR, Complex #: 10
I
II
v1
v2
v3
v4
v5
v6
0
0.2
0.4
0.6
0.8
1
Time [s]

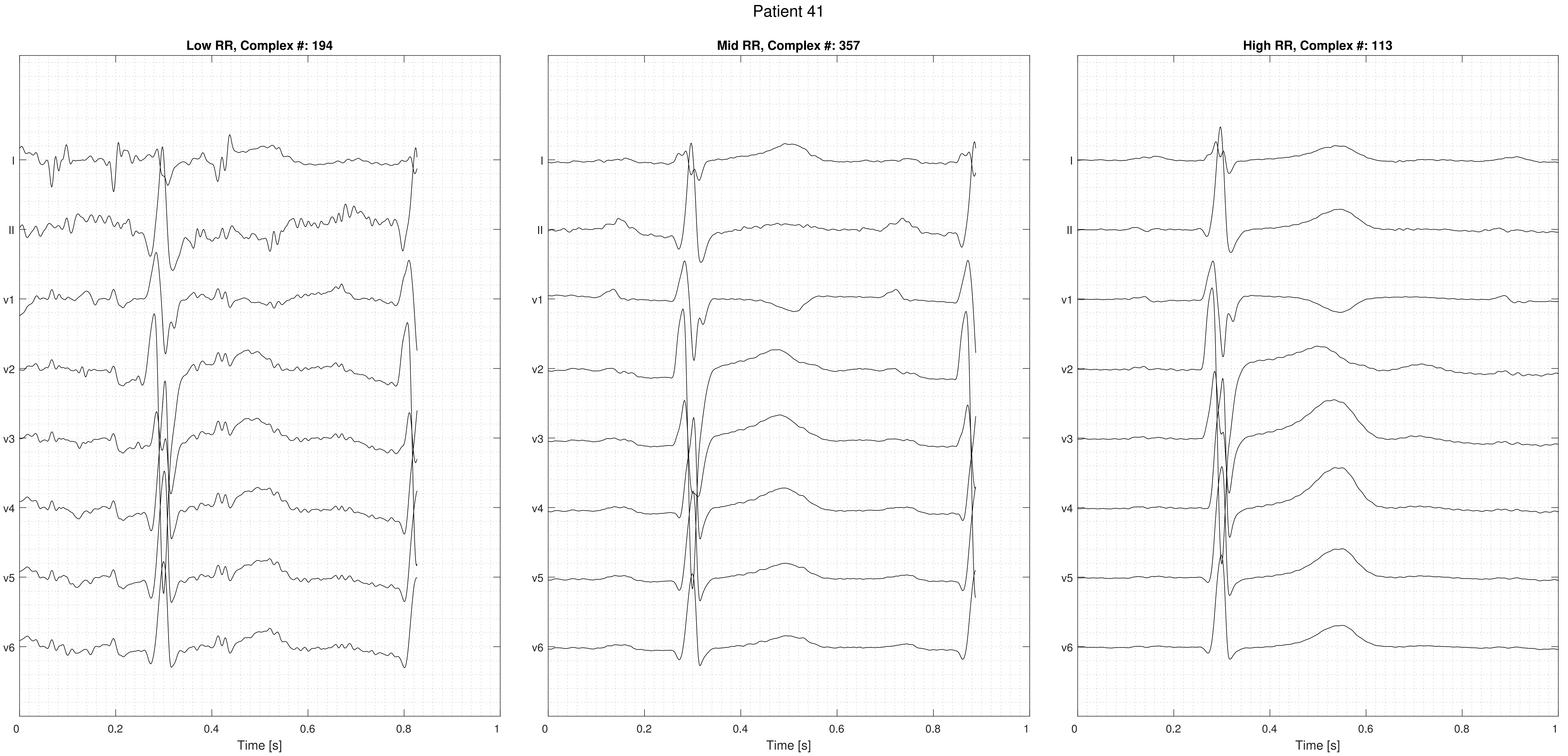
Patient 41
Low RR, Complex #: 194
Mid RR, Complex #: 357
High RR, Complex #: 113
I
II
v1
v2
v3
v4
v5
v6
0
0.2
0.4
0.6
0.8
1
Time [s]

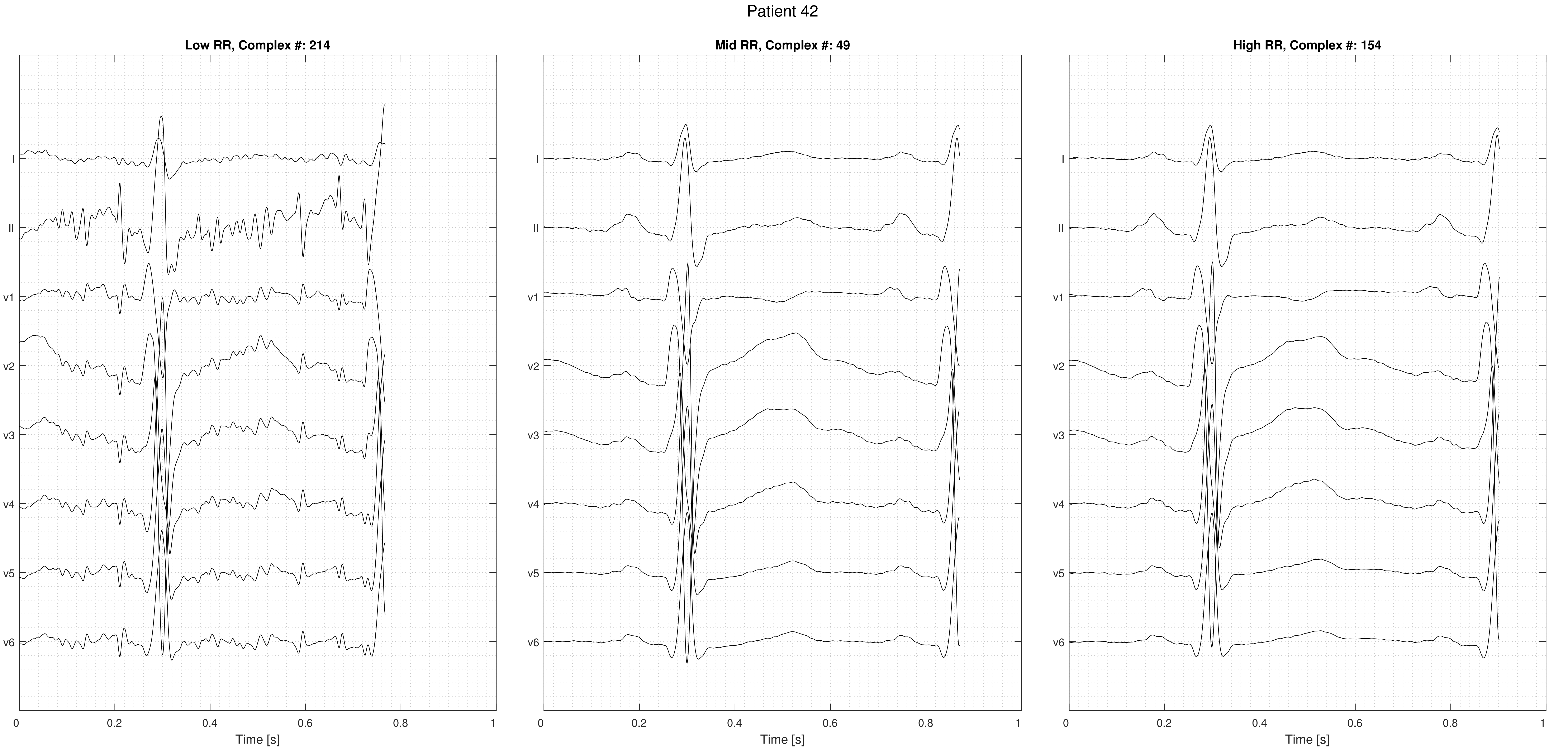
Patient 42
Low RR, Complex #: 214
Mid RR, Complex #: 49
High RR, Complex #: 154
I
II
v1
v2
v3
v4
v5
v6
0
0.2
0.4
0.6
0.8
1
Time [s]

Patient 43

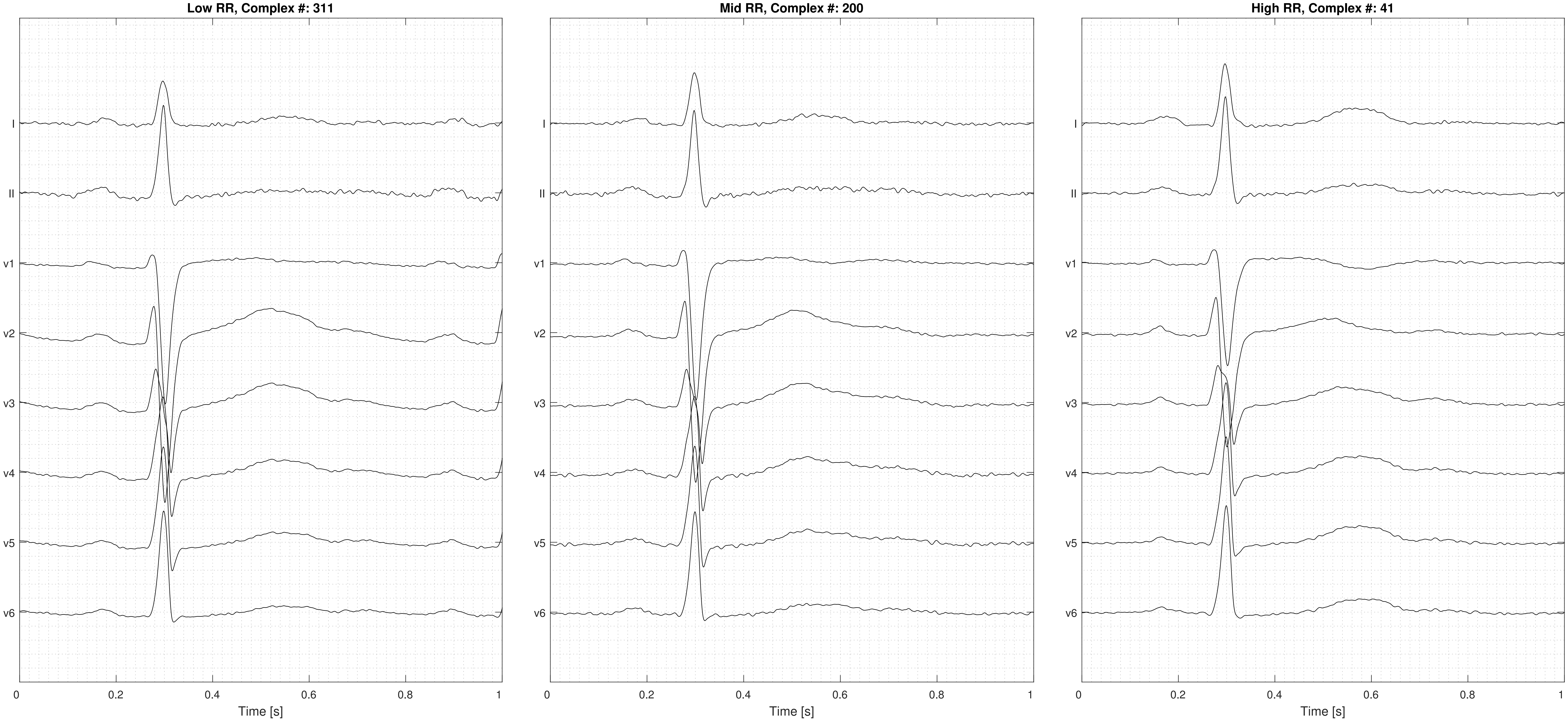

Low RR, Complex #: 311
Mid RR, Complex #: 200
High RR, Complex #: 41
I
II
v1
v2
v3
v4
v5
v6
0
0.2
0.4
0.6
0.8
1
Time [s]

Patient 44

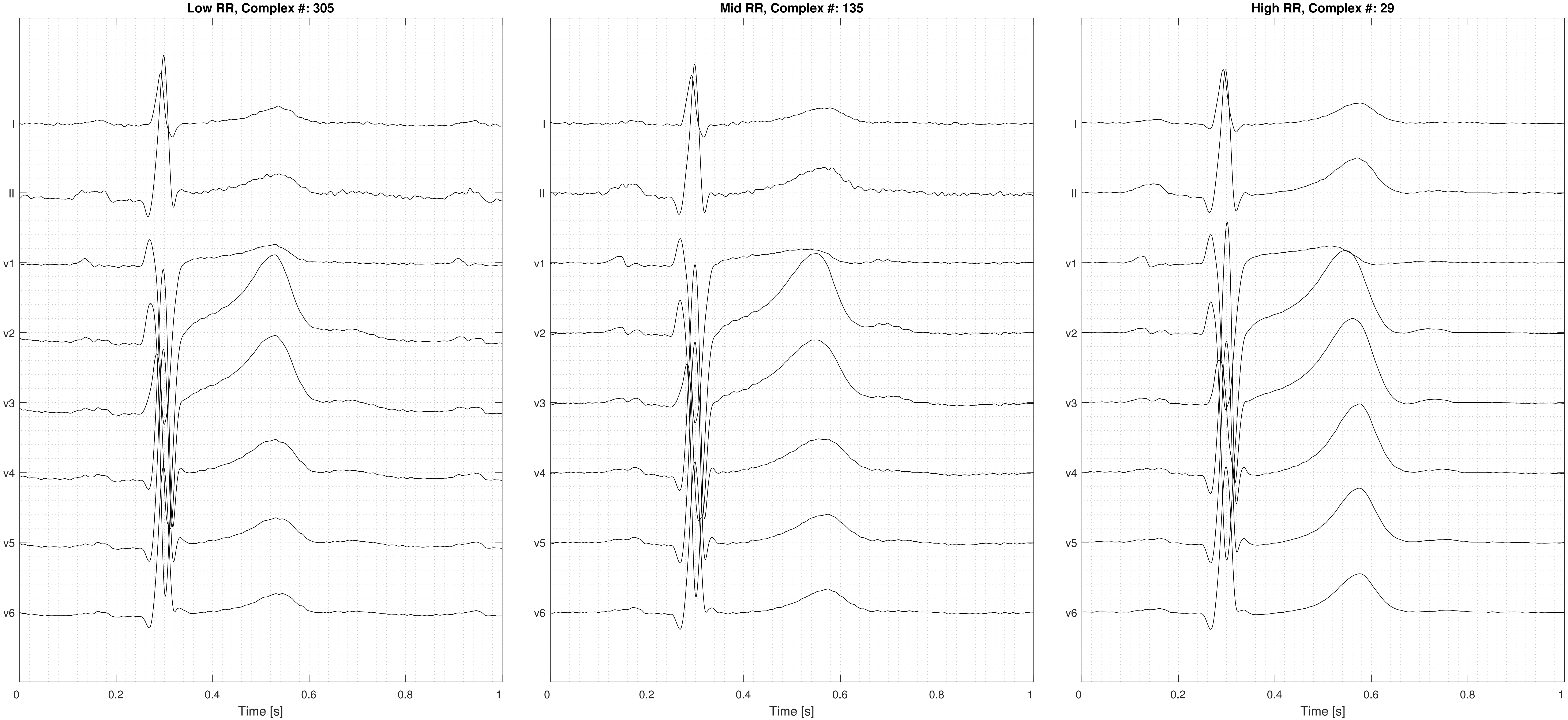

Low RR, Complex #: 305
Mid RR, Complex #: 135
High RR, Complex #: 29
I
II
v1
v2
v3
v4
v5
v6
0
0.2
0.4
0.6
0.8
1
Time [s]

Patient 45

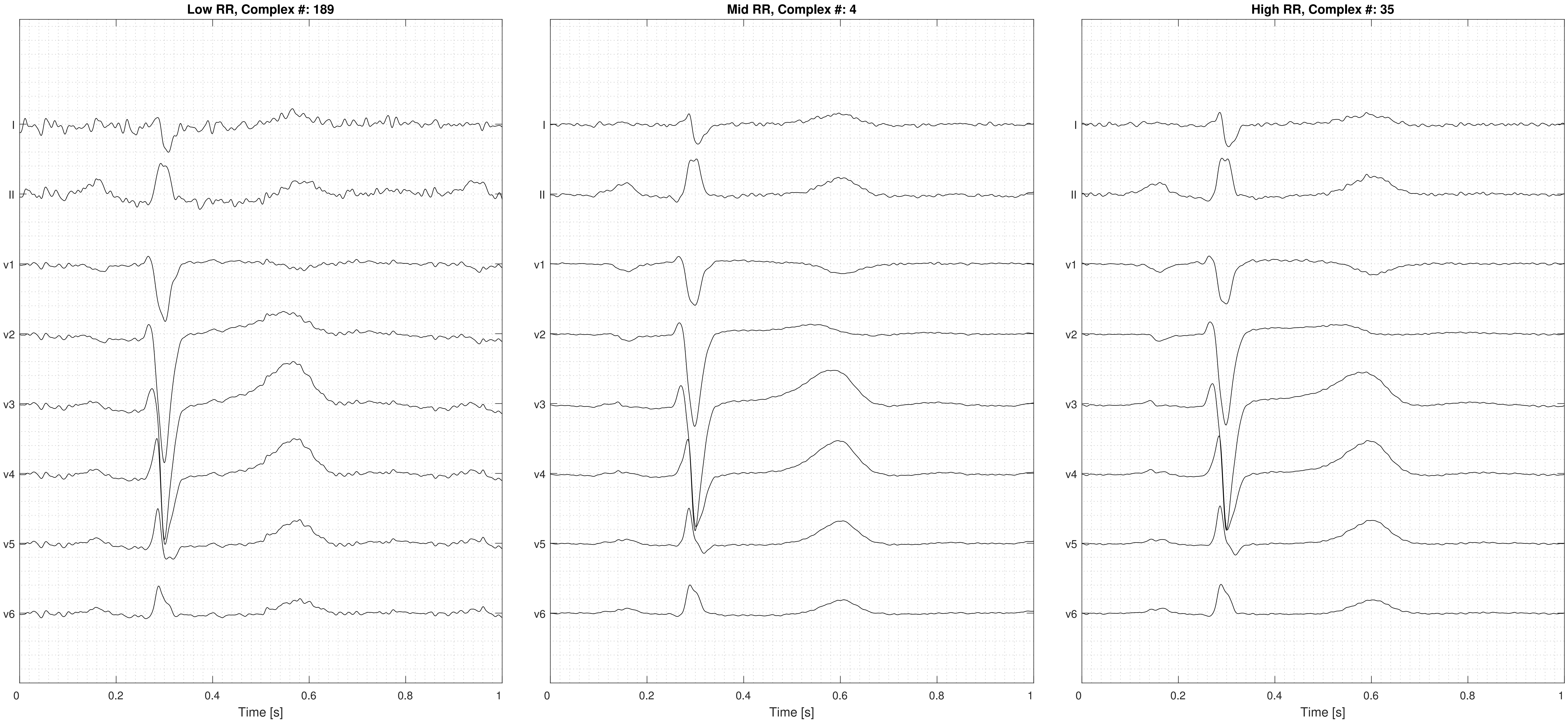

Low RR, Complex #: 189
Mid RR, Complex #: 4
High RR, Complex #: 35
I
II
v1
v2
v3
v4
v5
v6
0
0.2
0.4
0.6
0.8
1
Time [s]

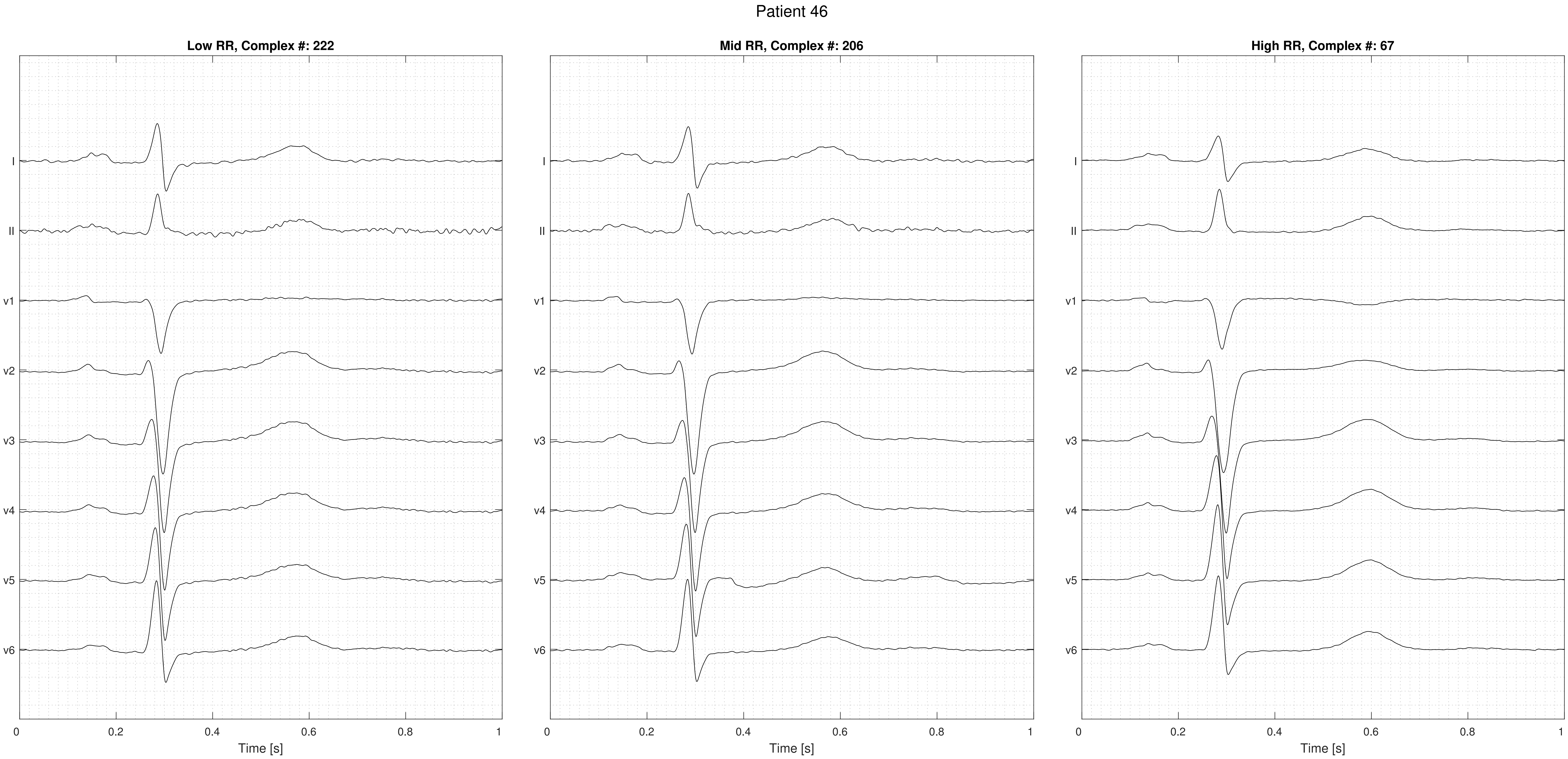
Patient 46
Low RR, Complex #: 222
Mid RR, Complex #: 206
High RR, Complex #: 67
I
II
v1
v2
v3
v4
v5
v6
0
0.2
0.4
0.6
0.8
1
Time [s]

Patient 47

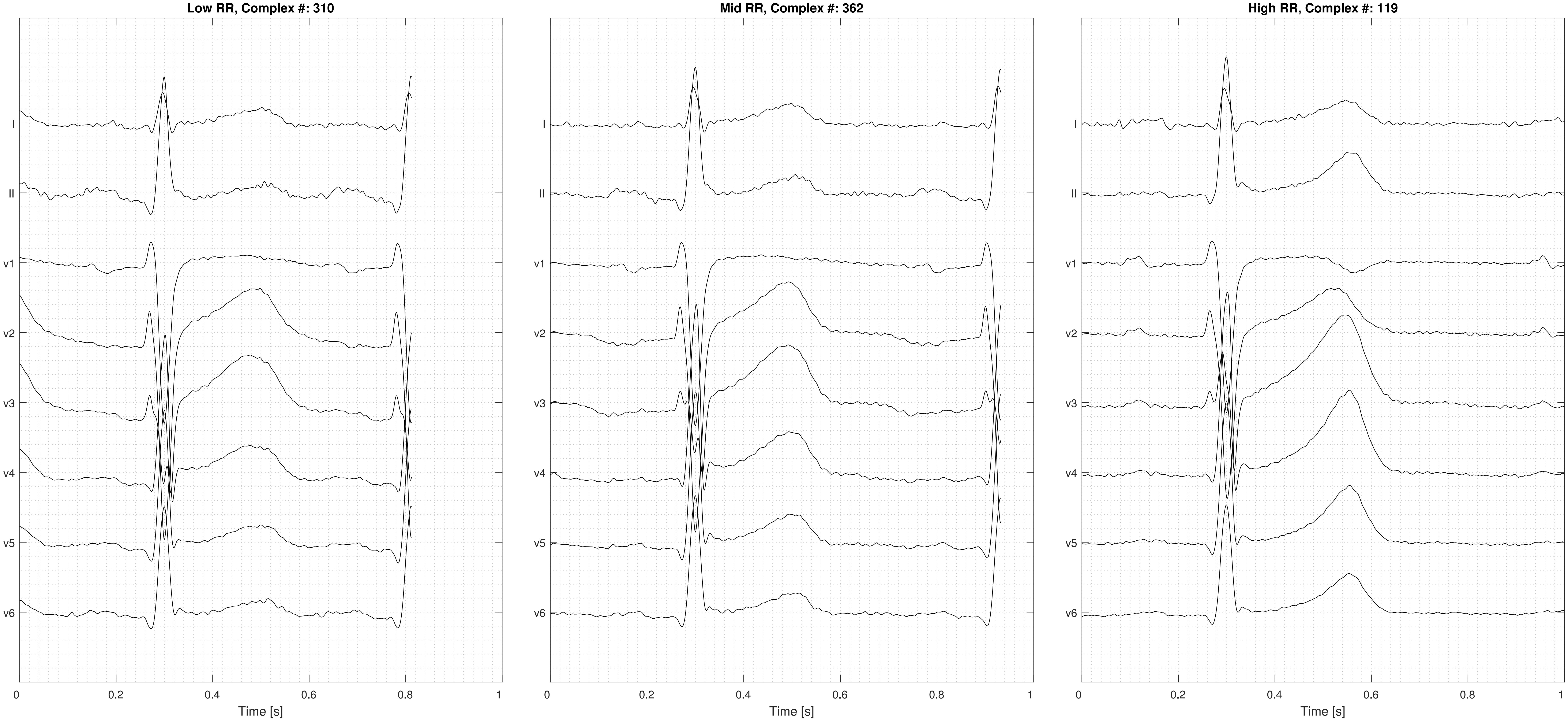

Low RR, Complex #: 310
Mid RR, Complex #: 362
High RR, Complex #: 119
I
II
v1
v2
v3
v4
v5
v6
0
0.2
0.4
0.6
0.8
1
Time [s]

Patient 48

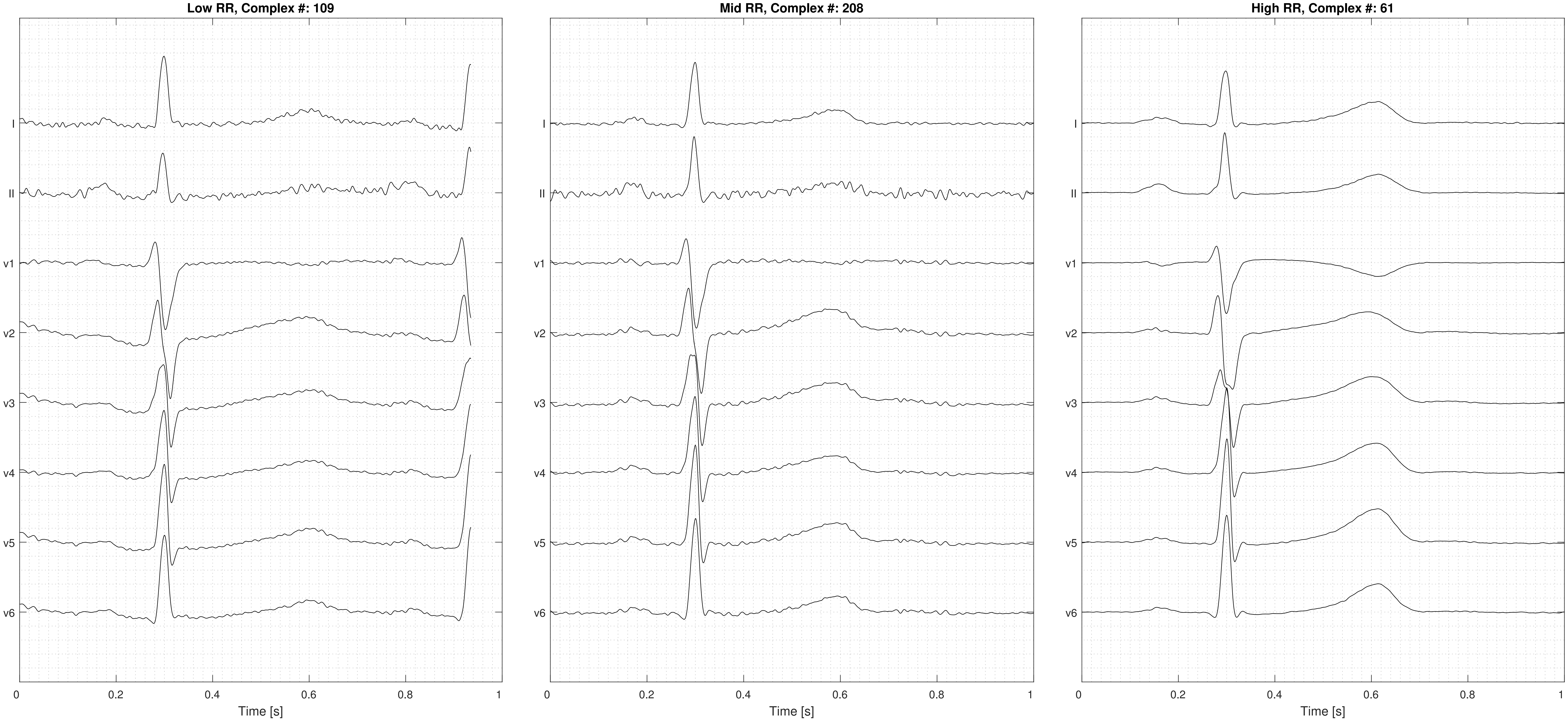

Low RR, Complex #: 109
Mid RR, Complex #: 208
High RR, Complex #: 61
I
II
v1
v2
v3
v4
v5
v6
0
0.2
0.4
0.6
0.8
1
Time [s]

Patient 49

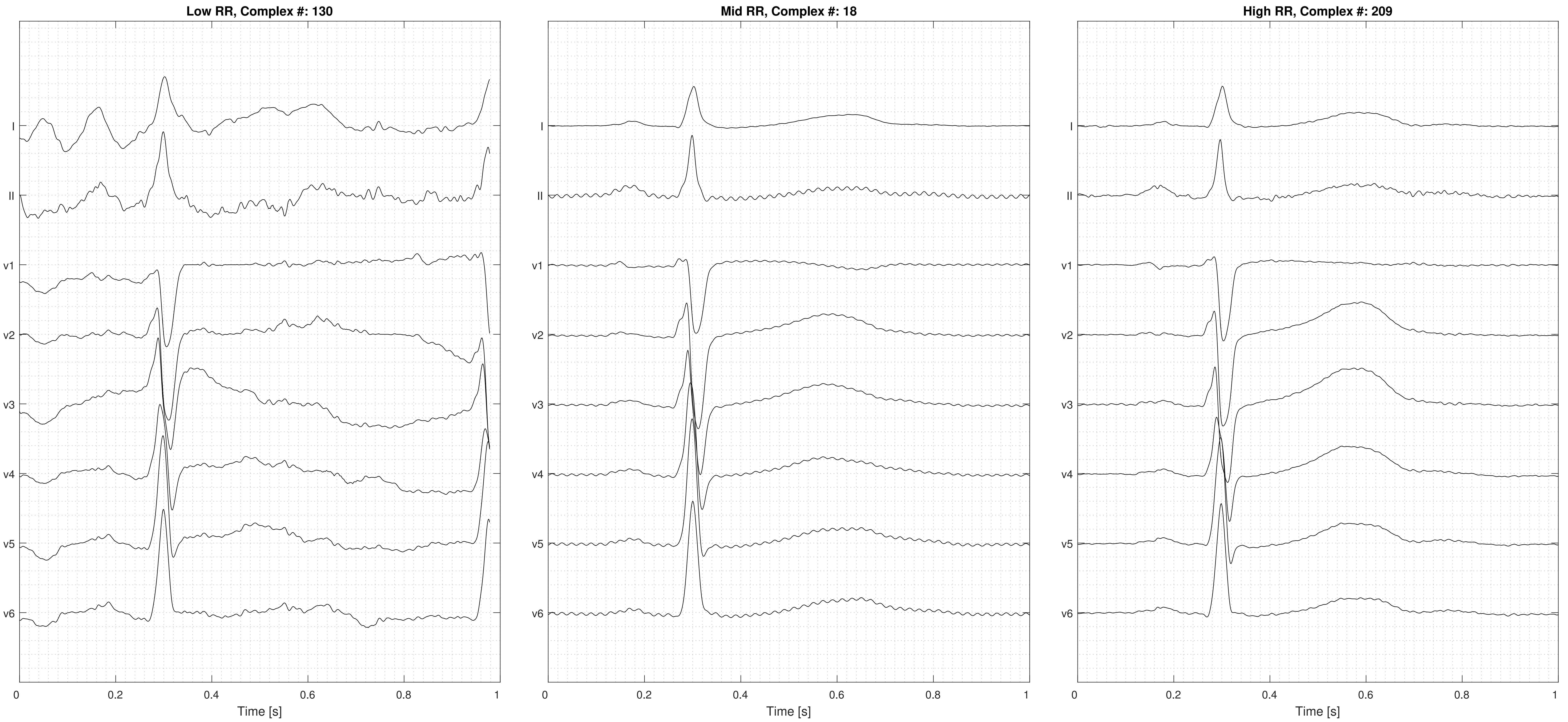
Low RR, Complex #: 130
Mid RR, Complex #: 18
High RR, Complex #: 209
I
II
v1
v2
v3
v4
v5
v6
0
0.2
0.4
0.6
0.8
1
Time [s]

Patient 50

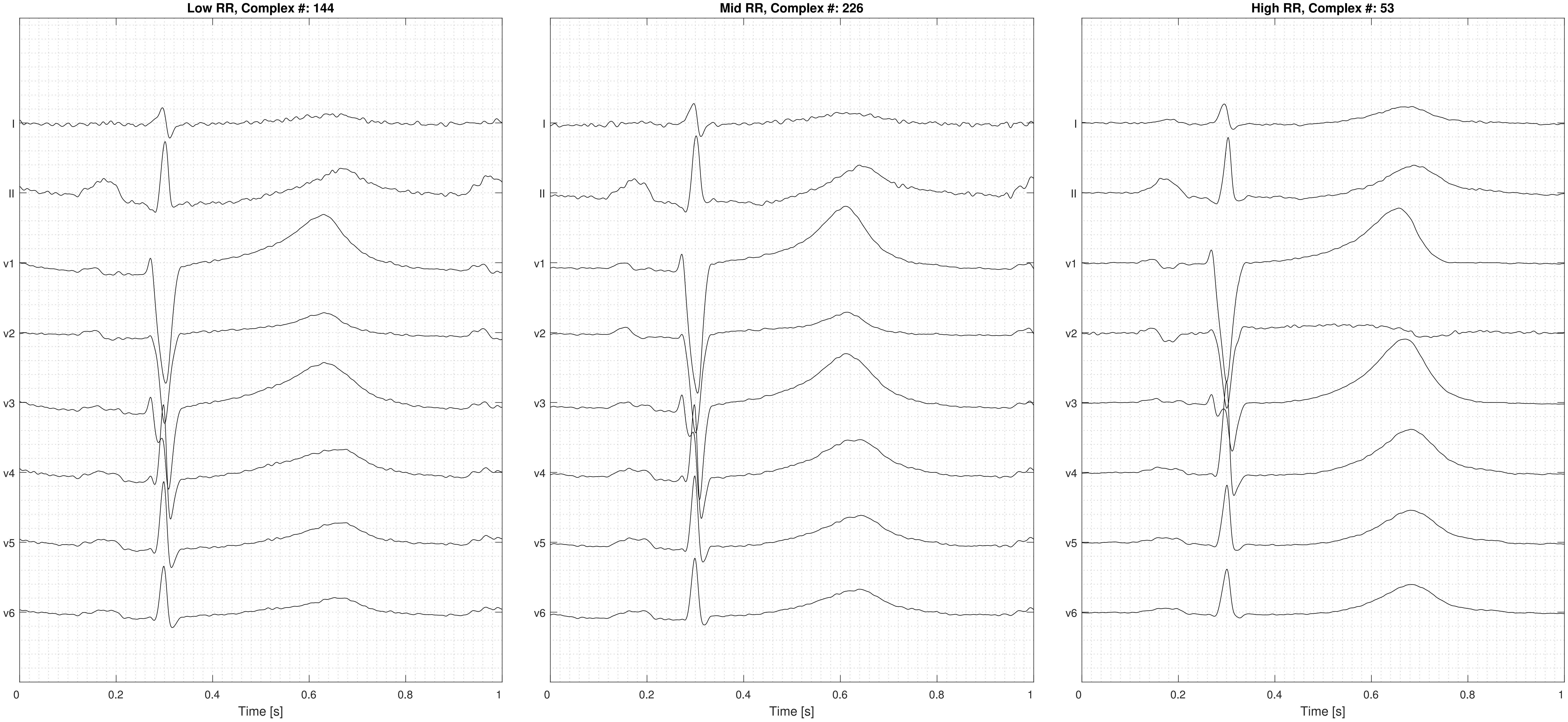

Low RR, Complex #: 144
Mid RR, Complex #: 226
High RR, Complex #: 53
I
II
v1
v2
v3
v4
v5
v6
0
0.2
0.4
0.6
0.8
1
Time [s]

Patient 51

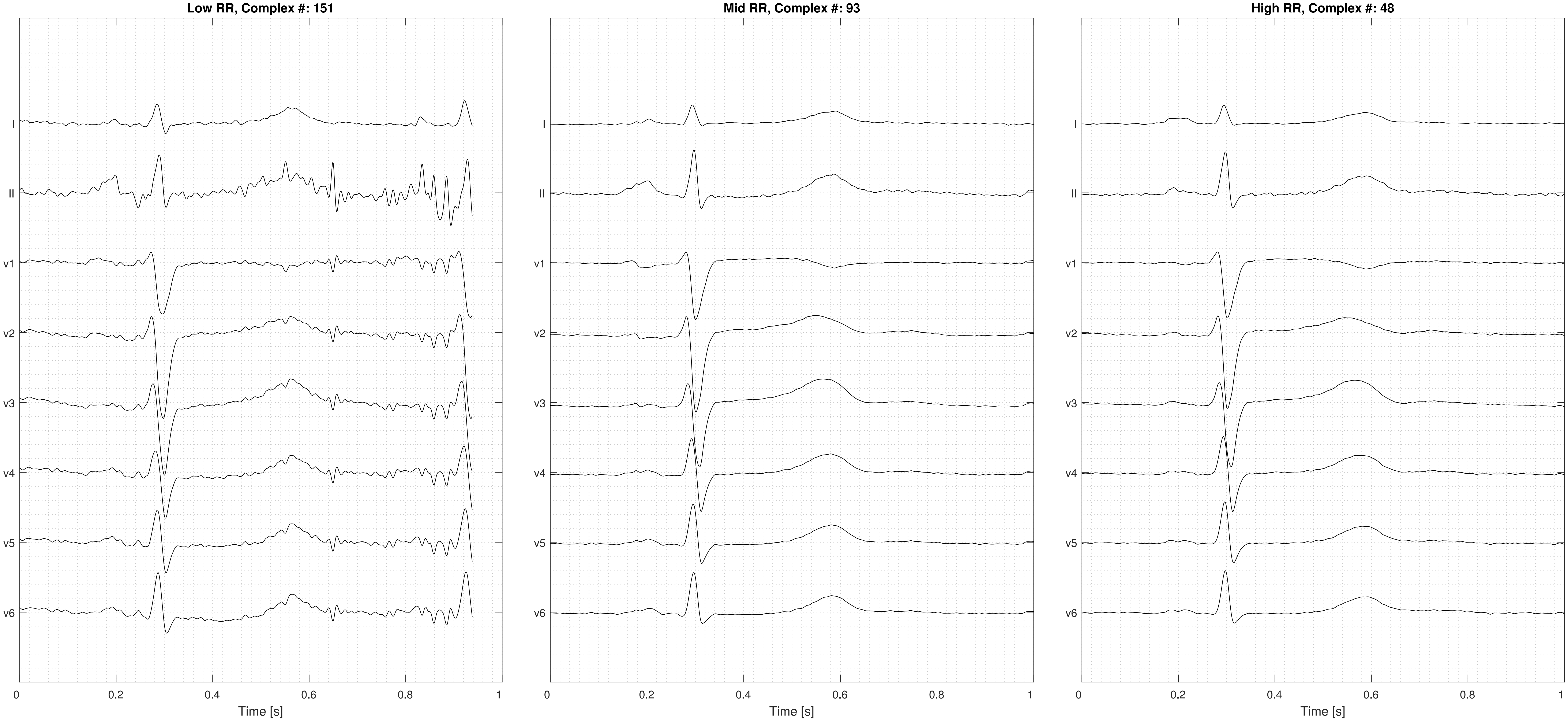

Low RR, Complex #: 151
Mid RR, Complex #: 93
High RR, Complex #: 48
I
II
v1
v2
v3
v4
v5
v6
0
0.2
0.4
0.6
0.8
1
Time [s]

Patient 52

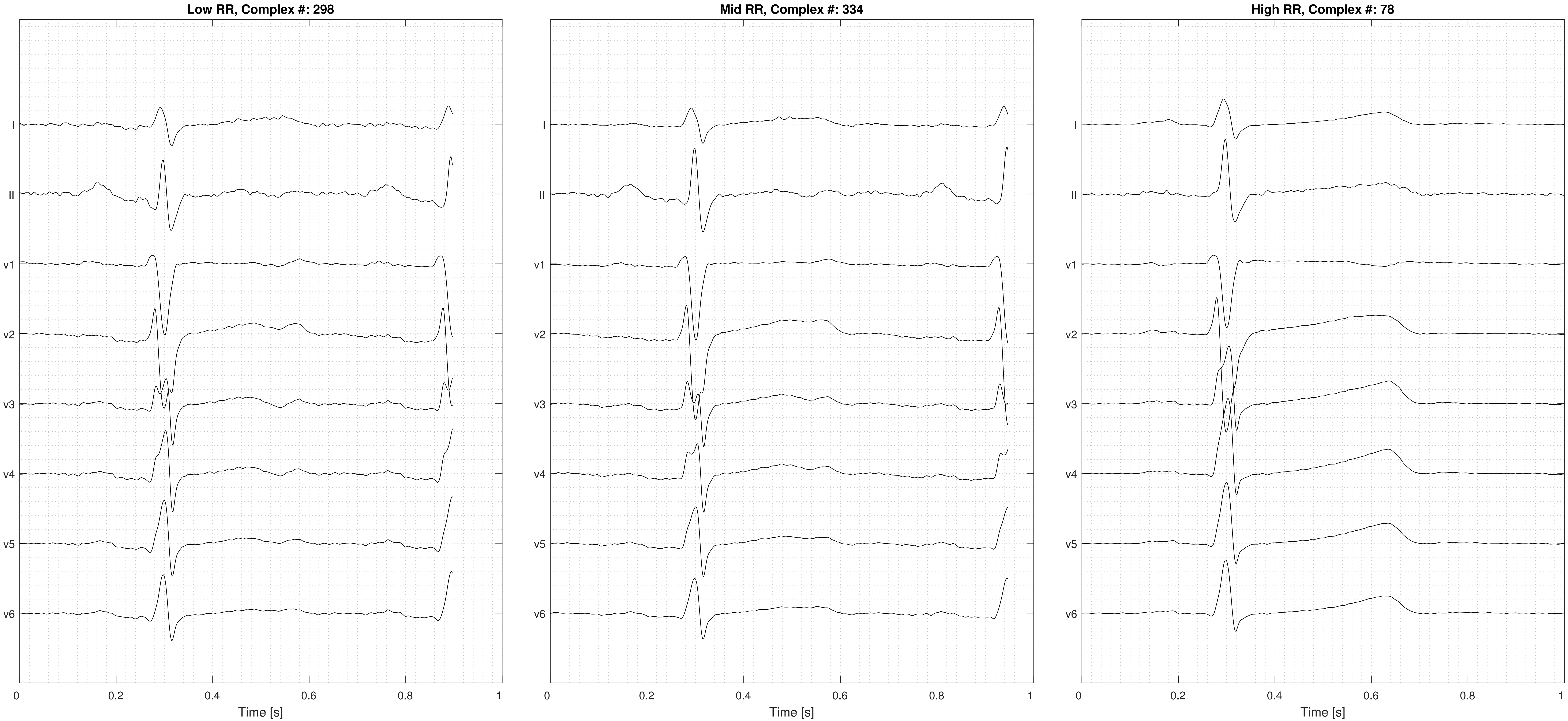

Low RR, Complex #: 298
Mid RR, Complex #: 334
High RR, Complex #: 78
I
II
v1
v2
v3
v4
v5
v6
0
0.2
0.4
0.6
0.8
1
Time [s]

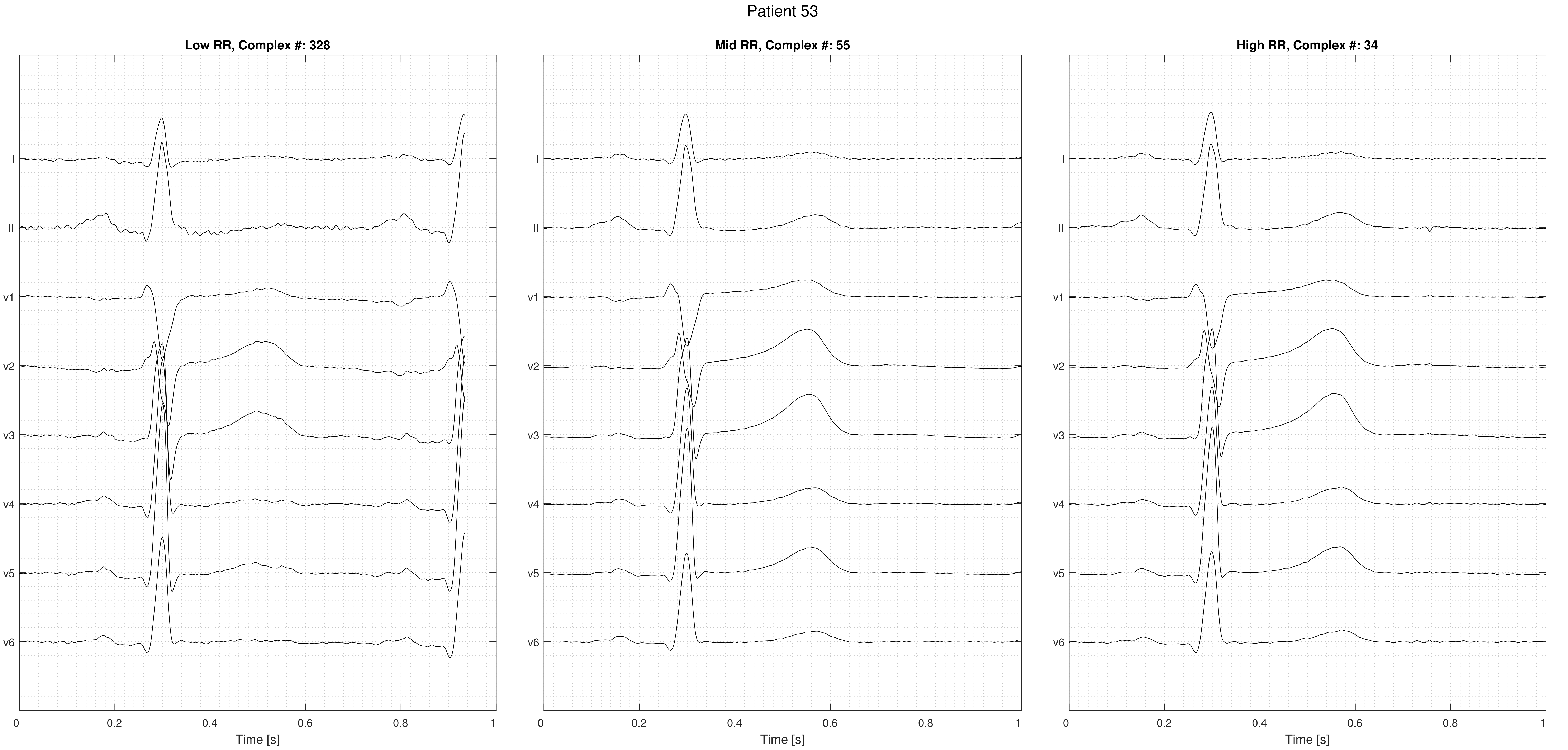

Patient 53
Low RR, Complex #: 328
Mid RR, Complex #: 55
High RR, Complex #: 34
I
II
v1
v2
v3
v4
v5
v6
0
0.2
0.4
0.6
0.8
1
Time [s]

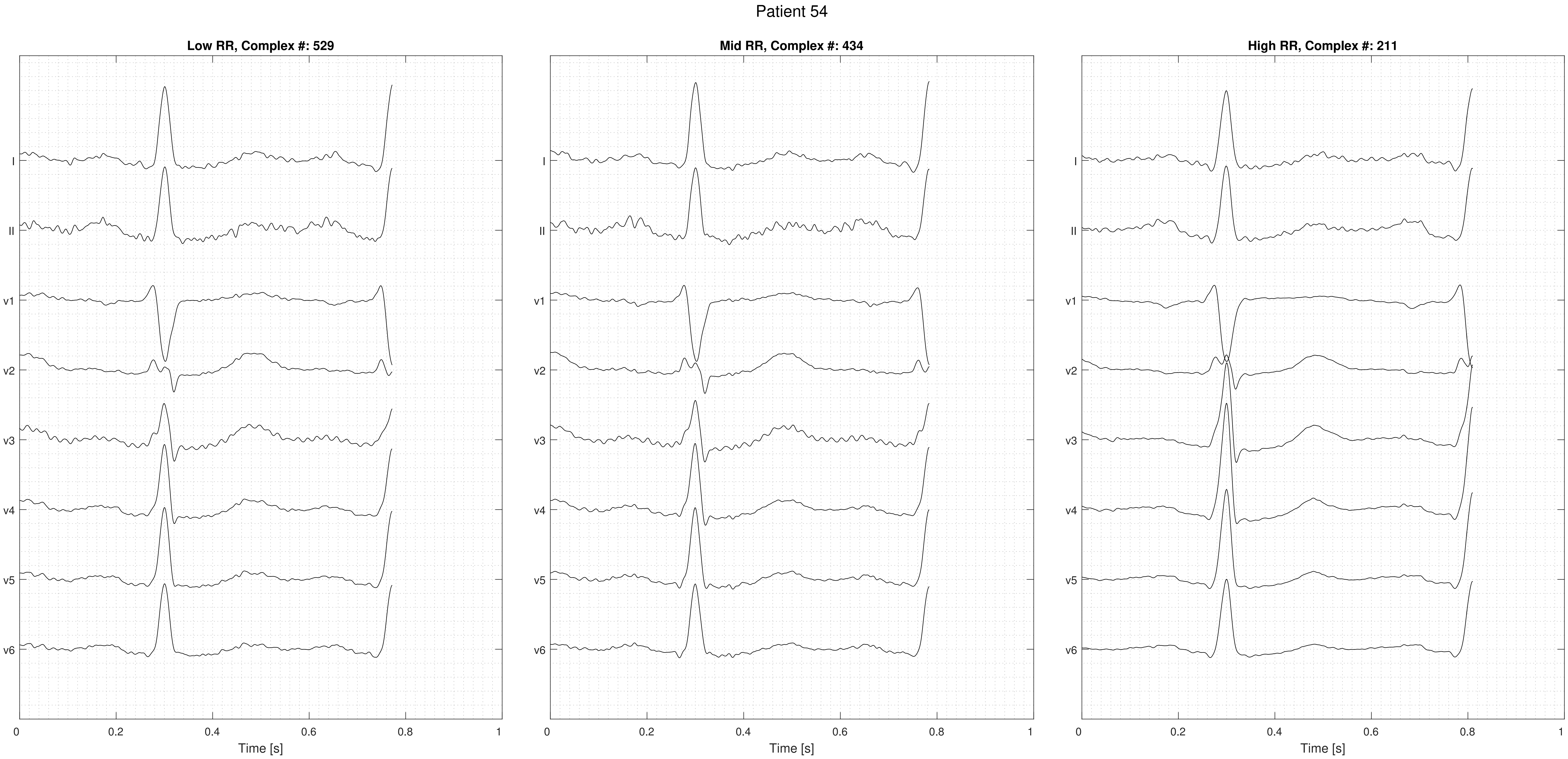

Patient 54
Low RR, Complex #: 529
Mid RR, Complex #: 434
High RR, Complex #: 211
I
II
v1
v2
v3
v4
v5
v6
0
0.2
0.4
0.6
0.8
1
Time [s]

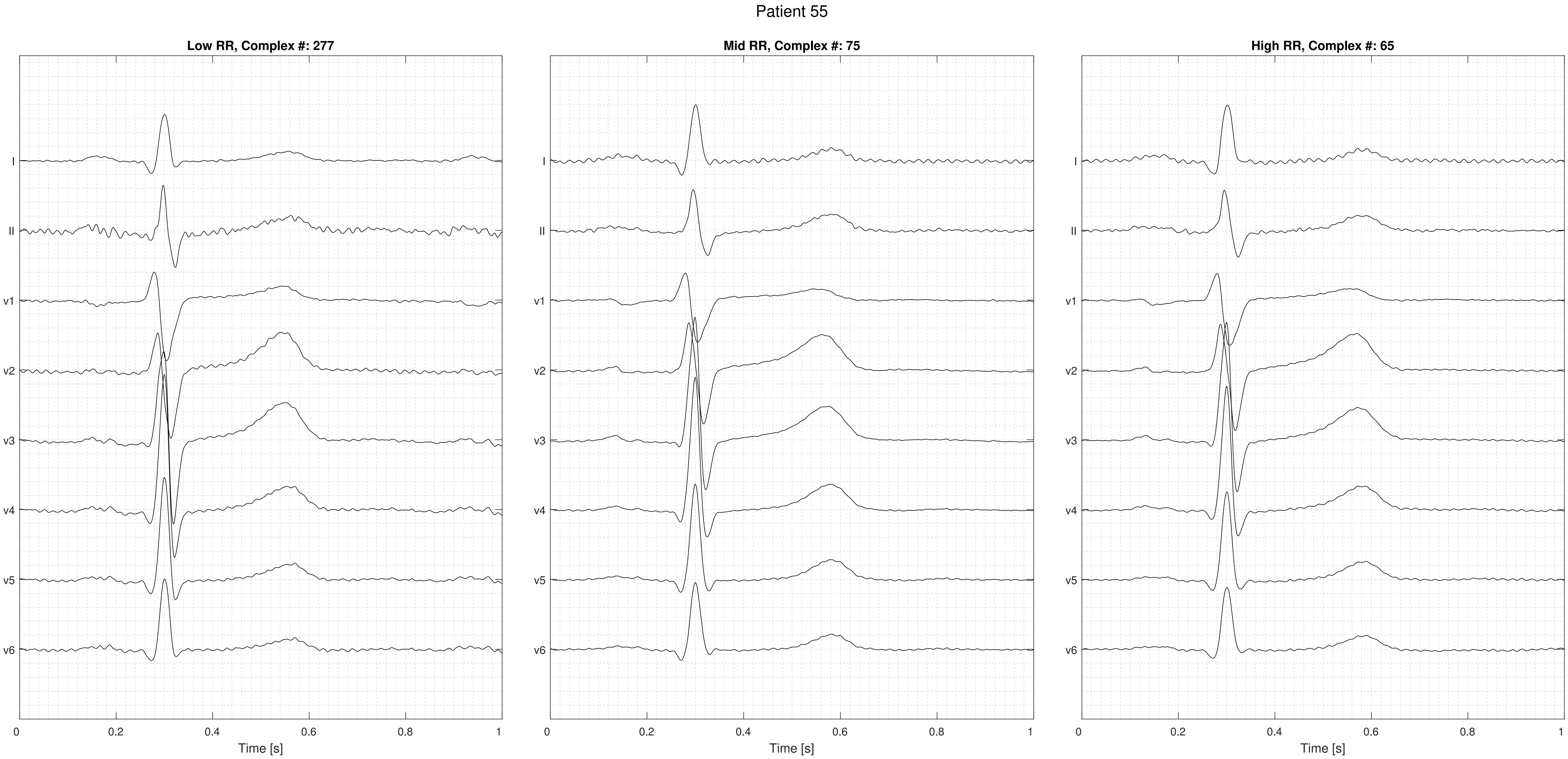
Patient 55
Low RR, Complex #: 277
Mid RR, Complex #: 75
High RR, Complex #: 65
I
II
v1
v2
v3
v4
v5
v6
0
0.2
0.4
0.6
0.8
1
Time [s]

Patient 56

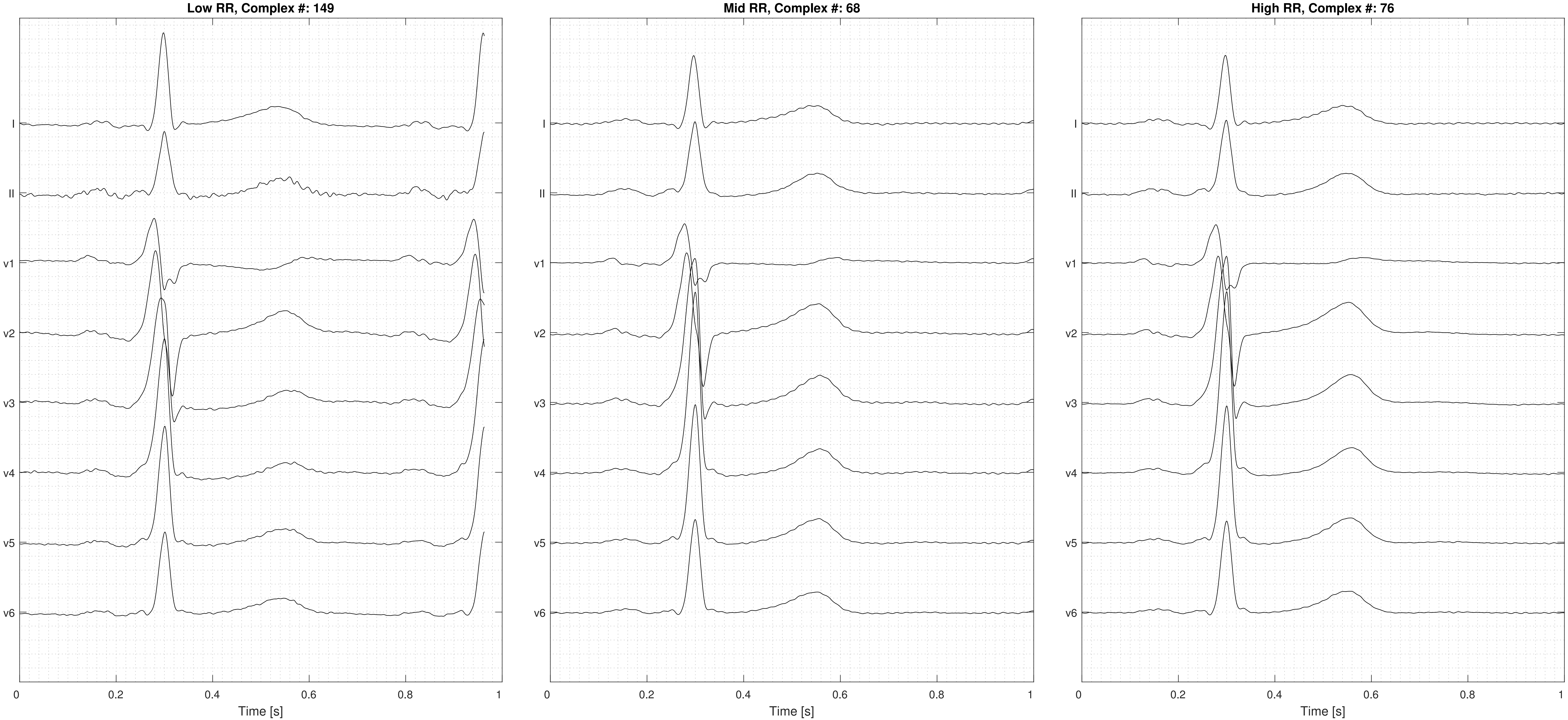

Low RR, Complex #: 149
Mid RR, Complex #: 68
High RR, Complex #: 76
I
II
v1
v2
v3
v4
v5
v6
0
0.2
0.4
0.6
0.8
1
Time [s]

Patient 57

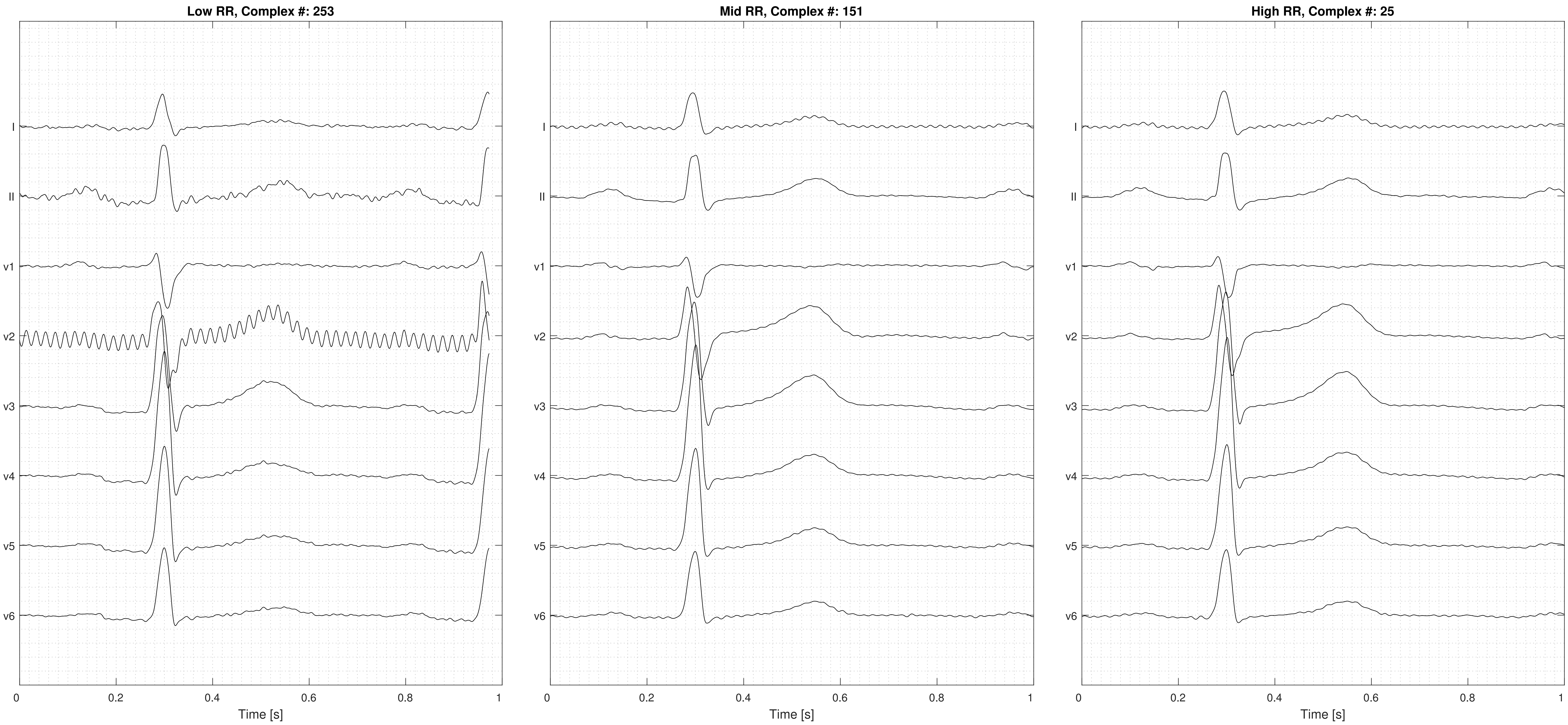

Low RR, Complex #: 253
Mid RR, Complex #: 151
High RR, Complex #: 25
I
II
v1
v2
v3
v4
v5
v6
0
0.2
0.4
0.6
0.8
1
Time [s]

Patient 58

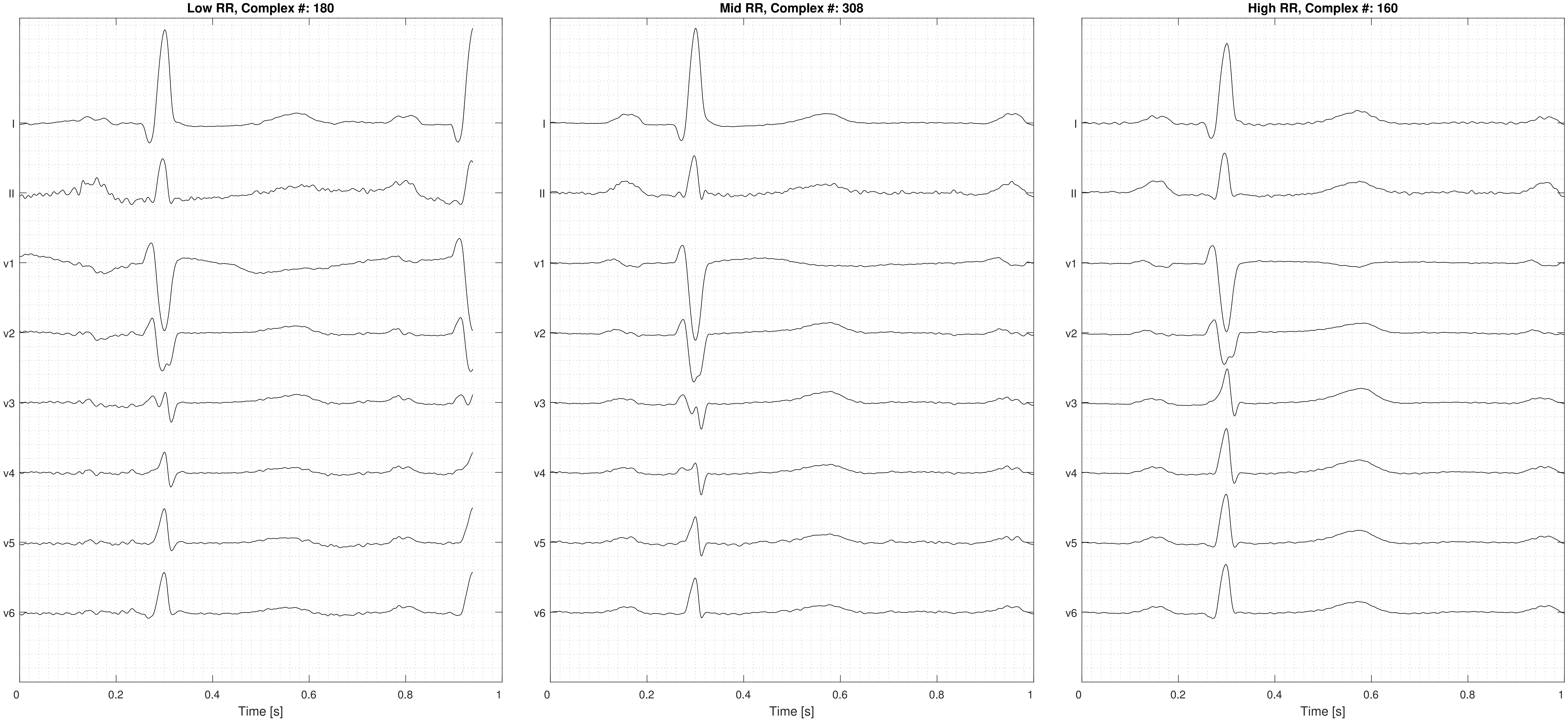

Low RR, Complex #: 180
Mid RR, Complex #: 308
High RR, Complex #: 160
I
II
v1
v2
v3
v4
v5
v6
0
0.2
0.4
0.6
0.8
1
Time [s]

Patient 59

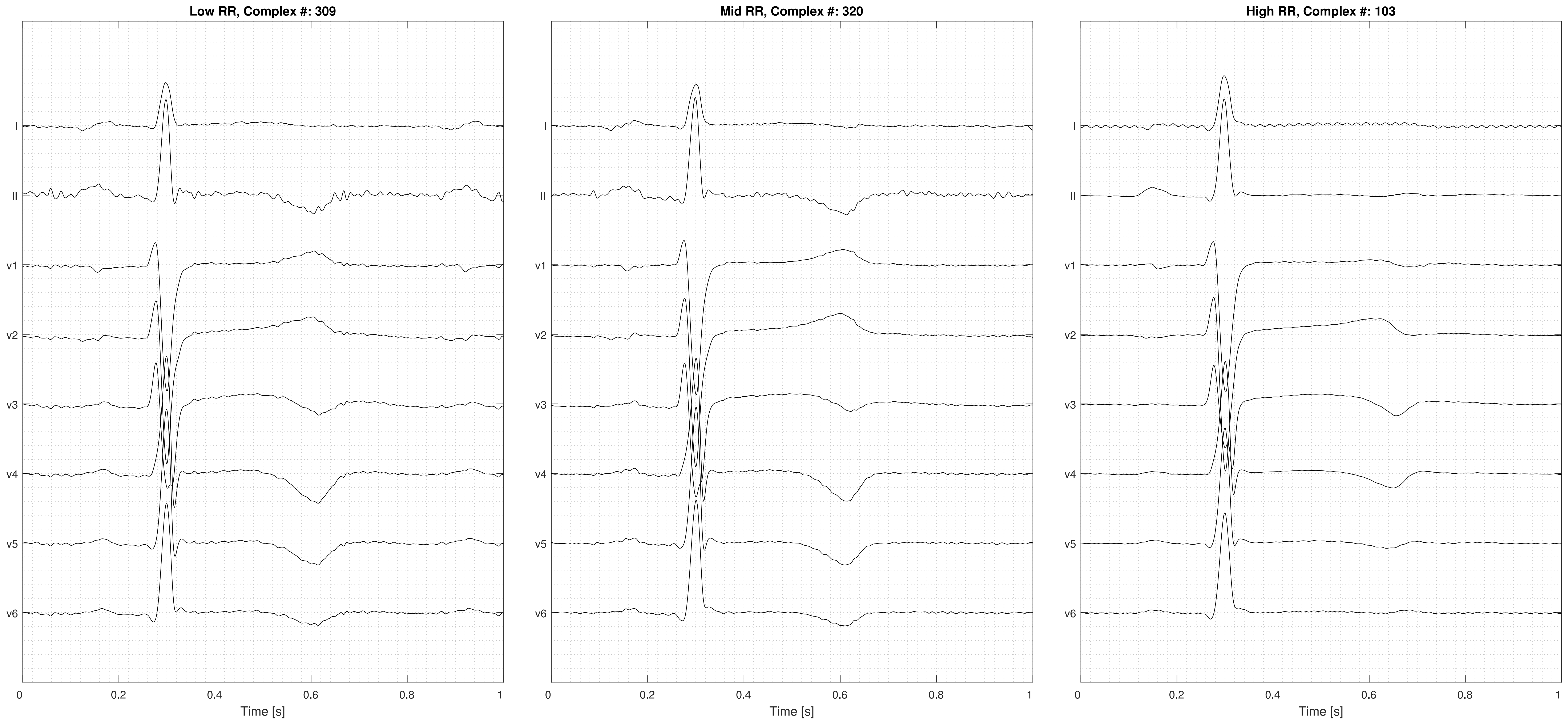

Low RR, Complex #: 309
Mid RR, Complex #: 320
High RR, Complex #: 103
I
II
v1
v2
v3
v4
v5
v6
0
0.2
0.4
0.6
0.8
1
Time [s]

Patient 60

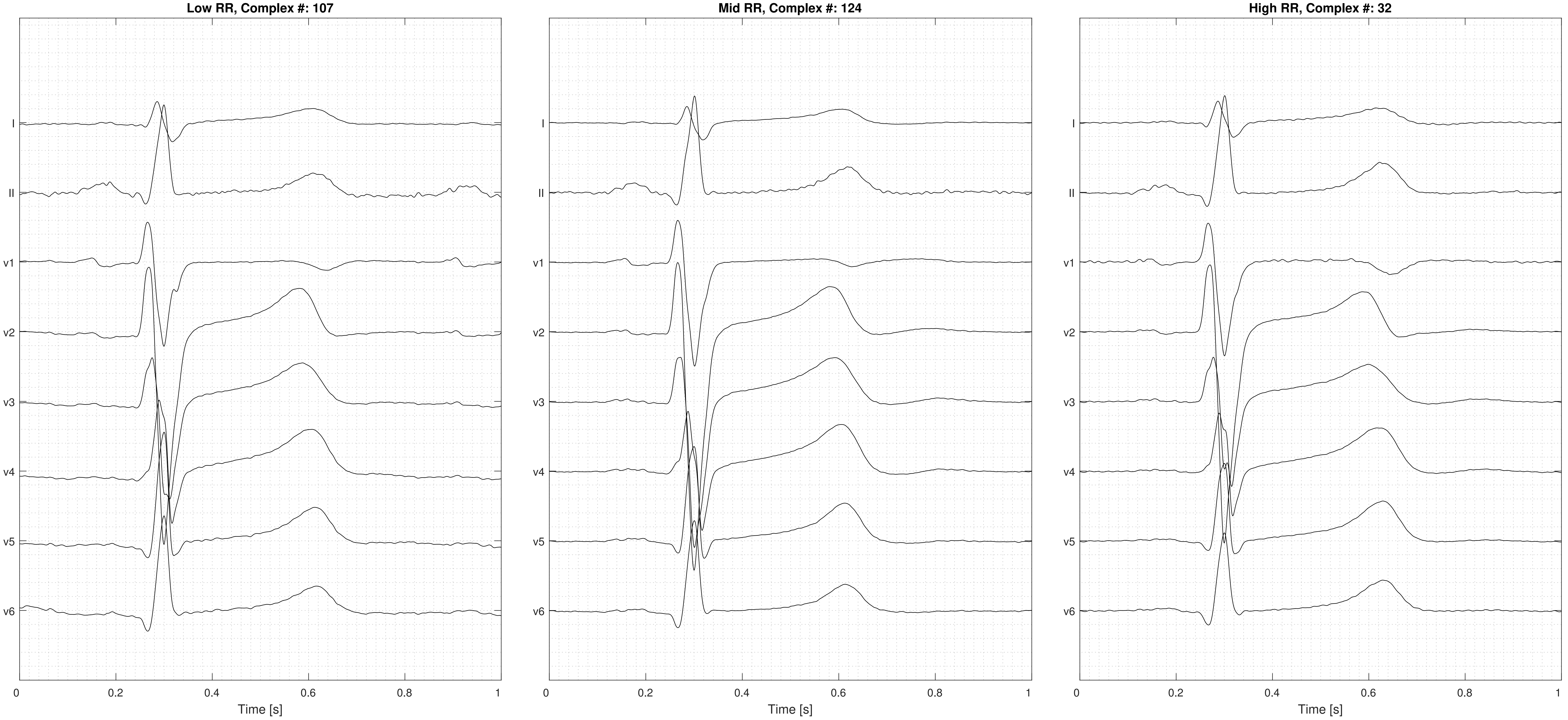
Low RR, Complex #: 107
Mid RR, Complex #: 124
High RR, Complex #: 32
I
II
v1
v2
v3
v4
v5
v6
0
0.2
0.4
0.6
0.8
1
Time [s]

Patient 61

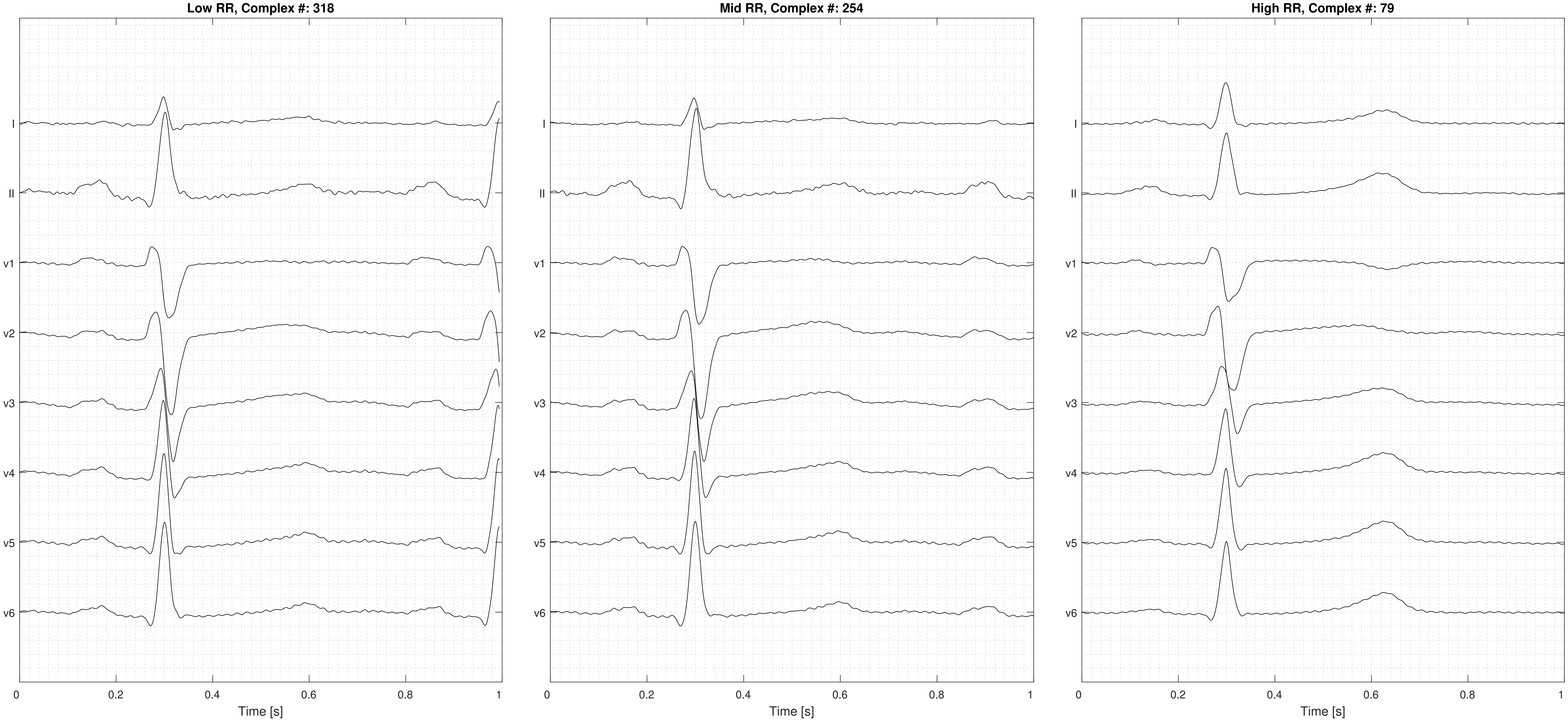

Low RR, Complex #: 318
Mid RR, Complex #: 254
High RR, Complex #: 79
I
II
v1
v2
v3
v4
v5
v6
0
0.2
0.4
0.6
0.8
1
Time [s]

Patient 62

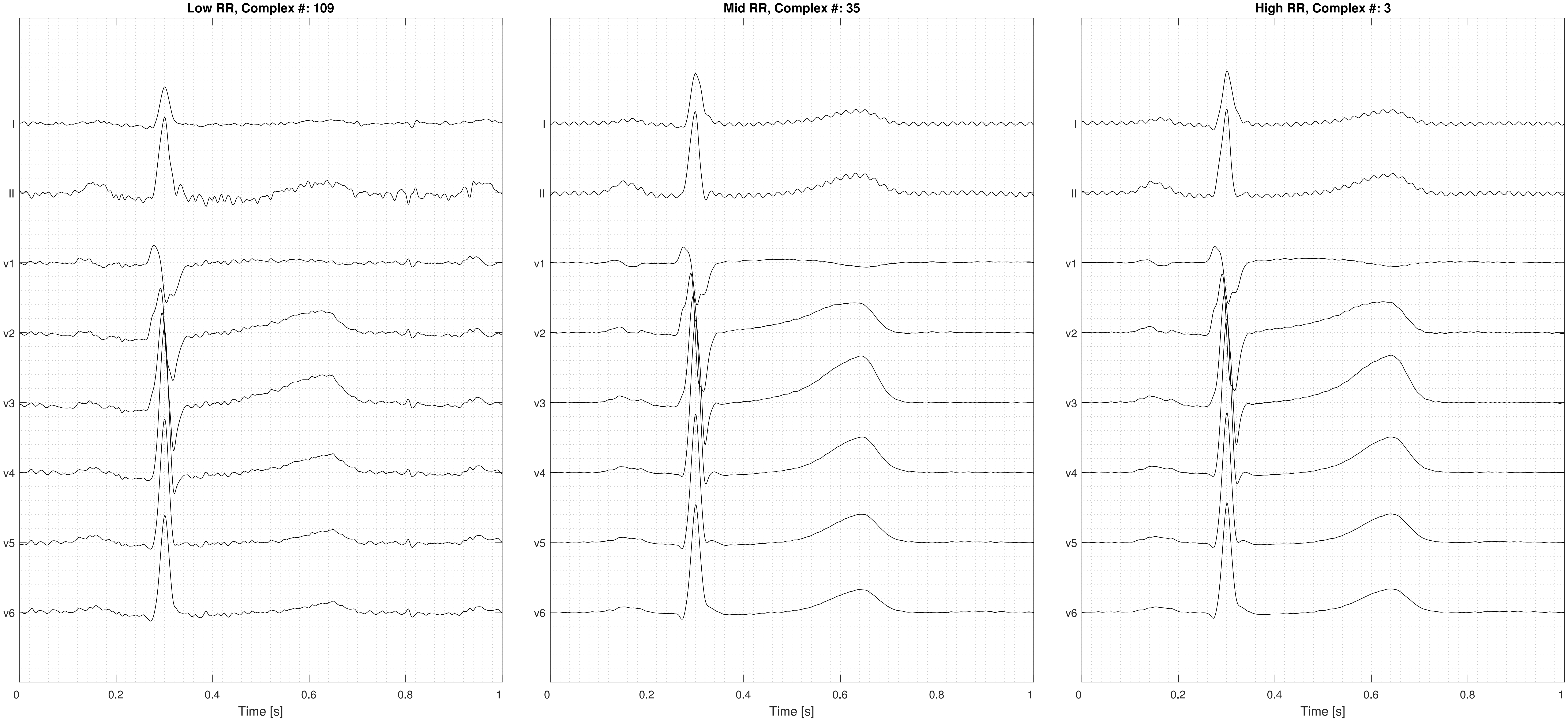

Low RR, Complex #: 109
Mid RR, Complex #: 35
High RR, Complex #: 3
I
II
v1
v2
v3
v4
v5
v6
0
0.2
0.4
0.6
0.8
1
Time [s]

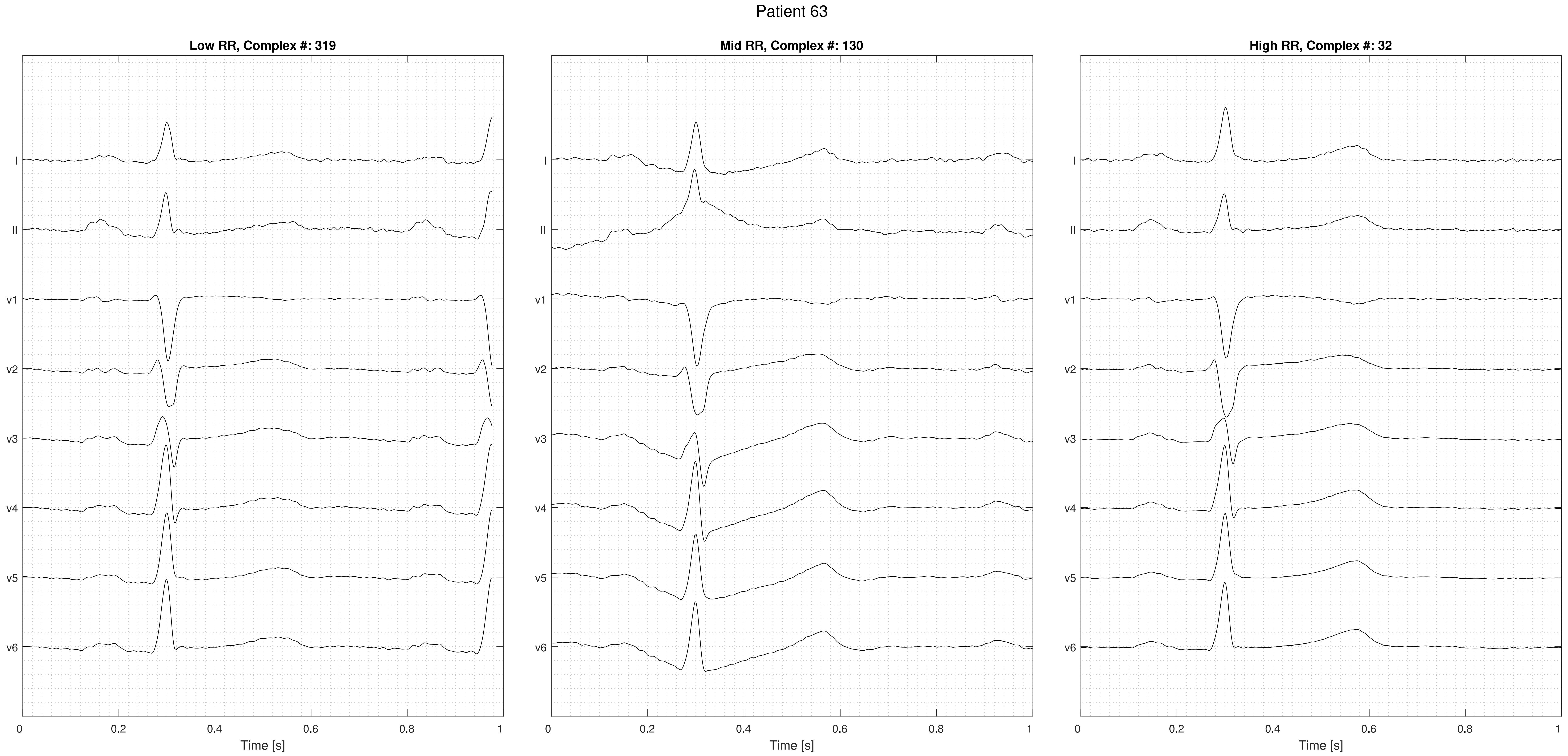

Patient 63
Low RR, Complex #: 319
Mid RR, Complex #: 130
High RR, Complex #: 32
I
II
v1
v2
v3
v4
v5
v6
0
0.2
0.4
0.6
0.8
1
Time [s]

Patient 64

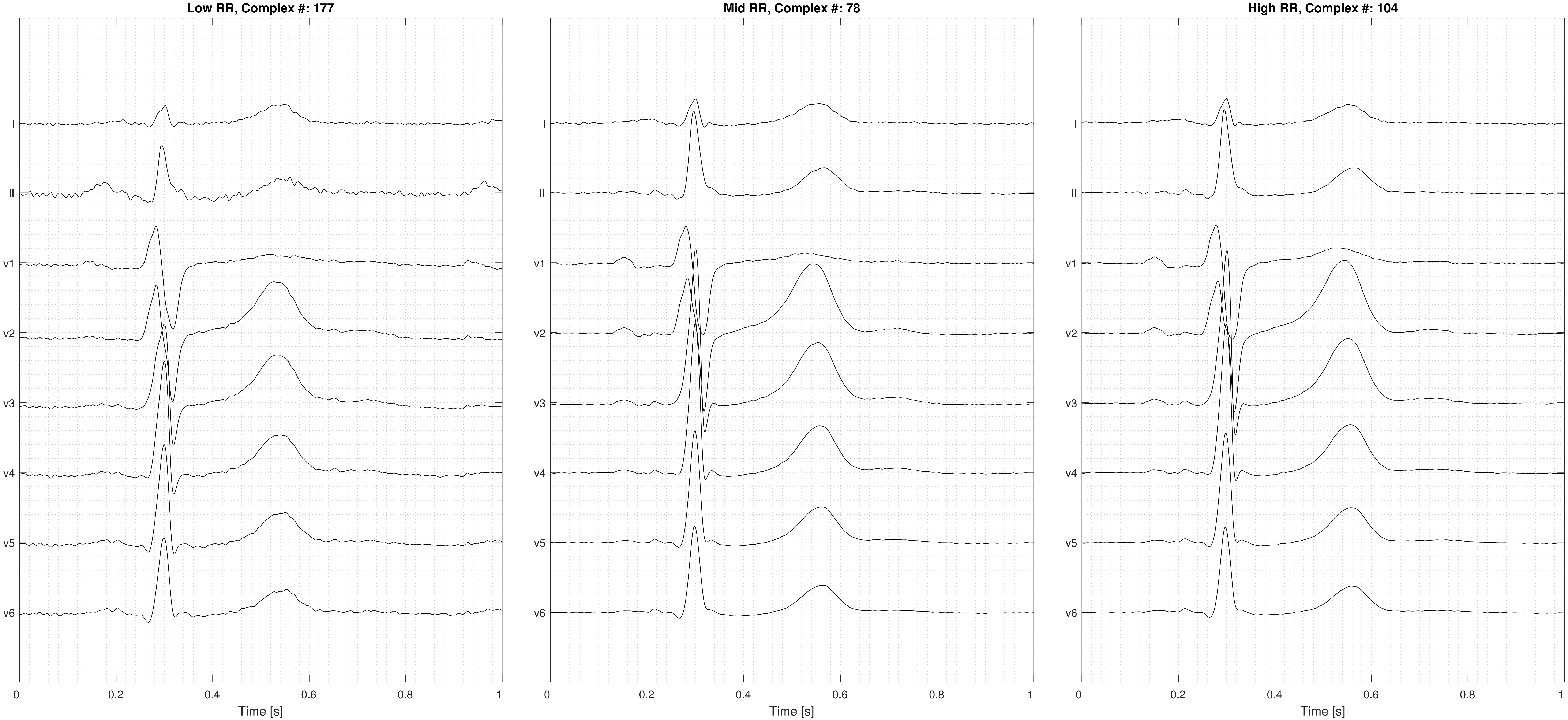

Low RR, Complex #: 177
Mid RR, Complex #: 78
High RR, Complex #: 104
I
II
v1
v2
v3
v4
v5
v6
0
0.2
0.4
0.6
0.8
1
Time [s]

Patient 65

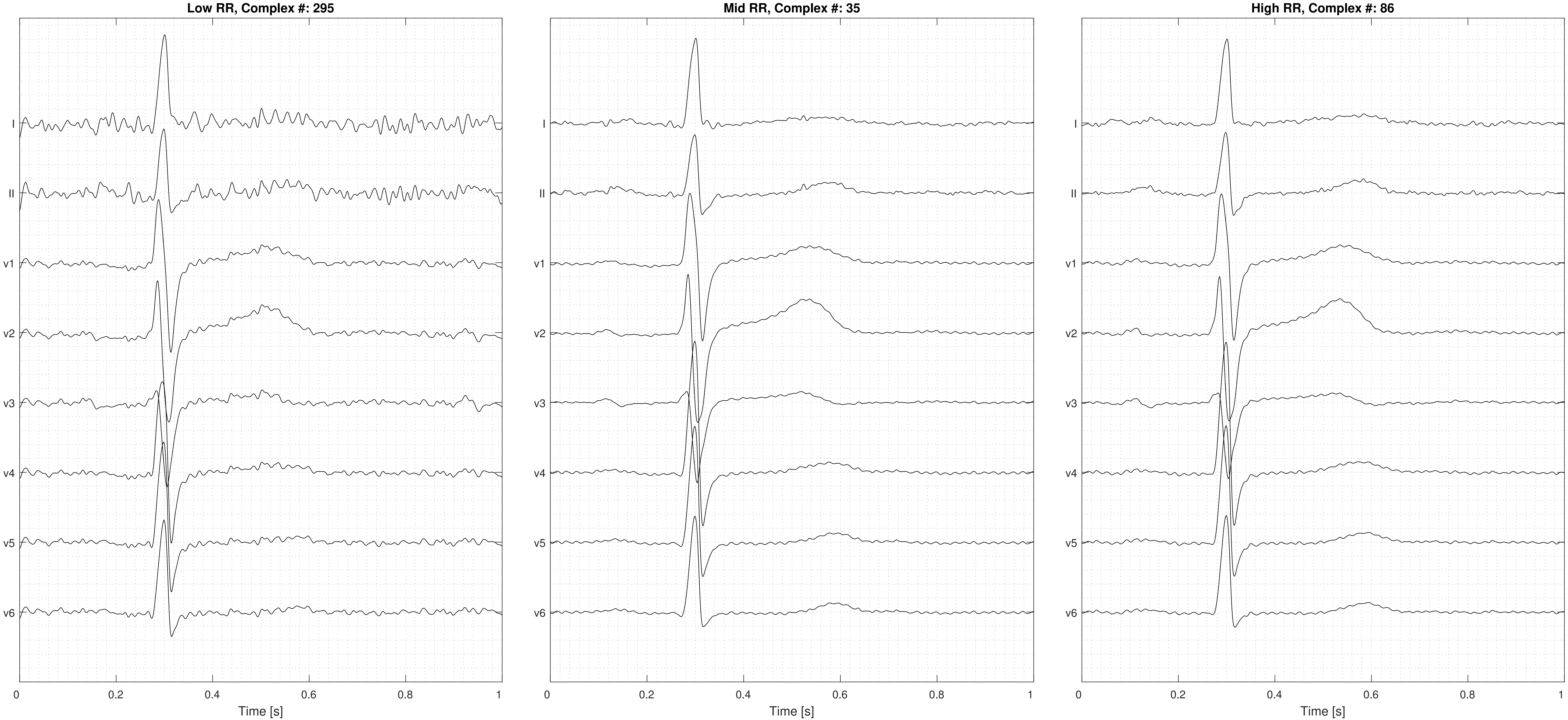

Low RR, Complex #: 295
Mid RR, Complex #: 35
High RR, Complex #: 86
I
II
v1
v2
v3
v4
v5
v6
0
0.2
0.4
0.6
0.8
1
Time [s]

Patient 66

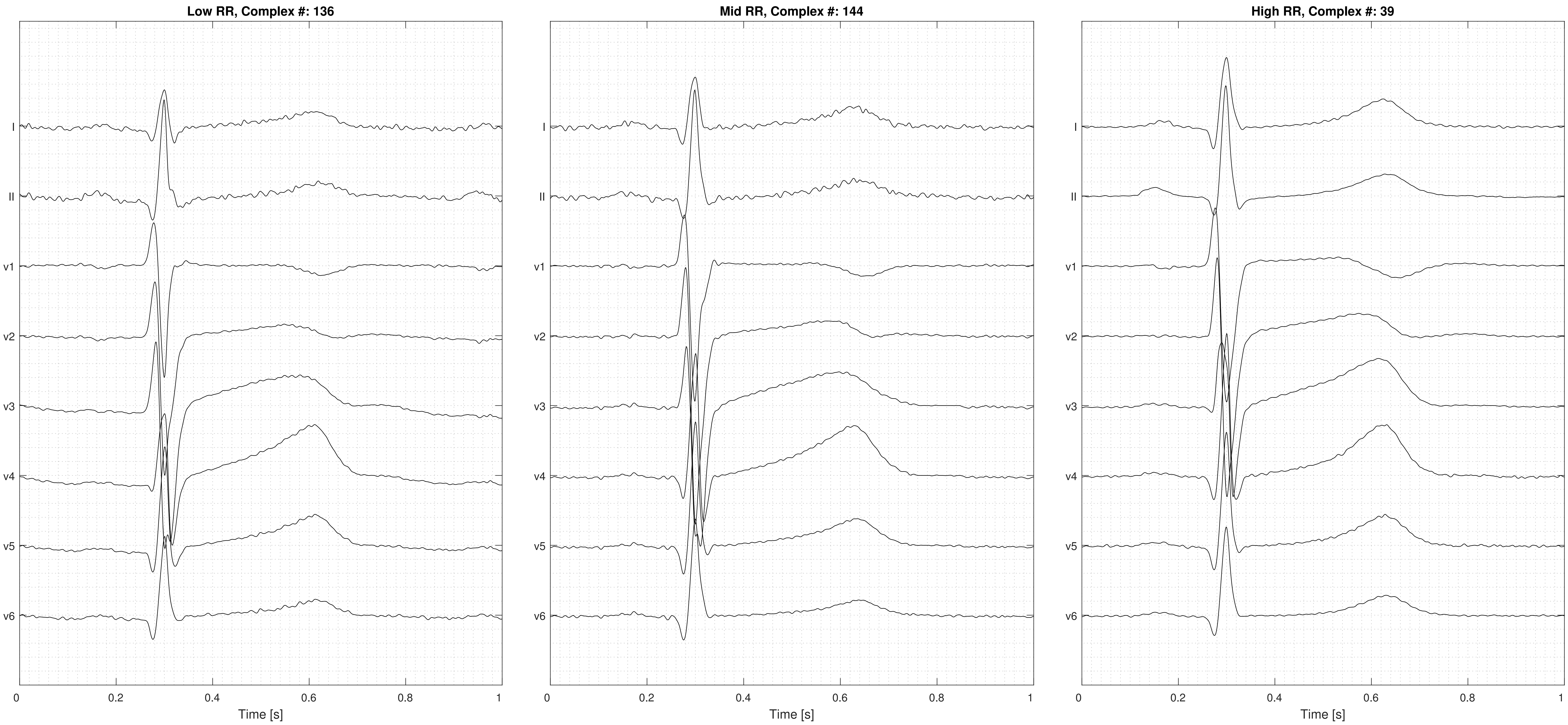

Low RR, Complex #: 136
Mid RR, Complex #: 144
High RR, Complex #: 39
I
II
v1
v2
v3
v4
v5
v6
0
0.2
0.4
0.6
0.8
1
Time [s]

Patient 67

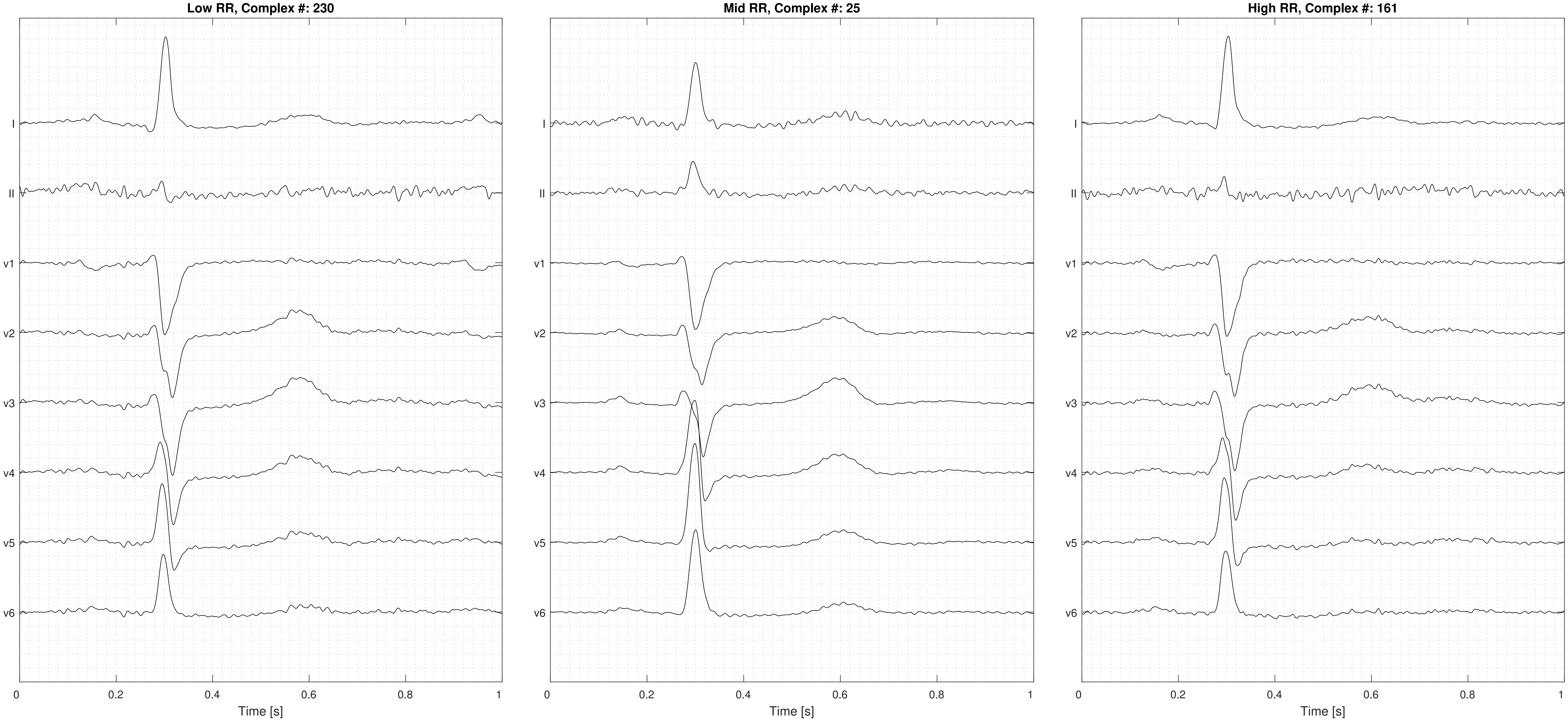

Low RR, Complex #: 230
Mid RR, Complex #: 25
High RR, Complex #: 161
I
II
v1
v2
v3
v4
v5
v6
0
0.2
0.4
0.6
0.8
1
Time [s]

Patient 68

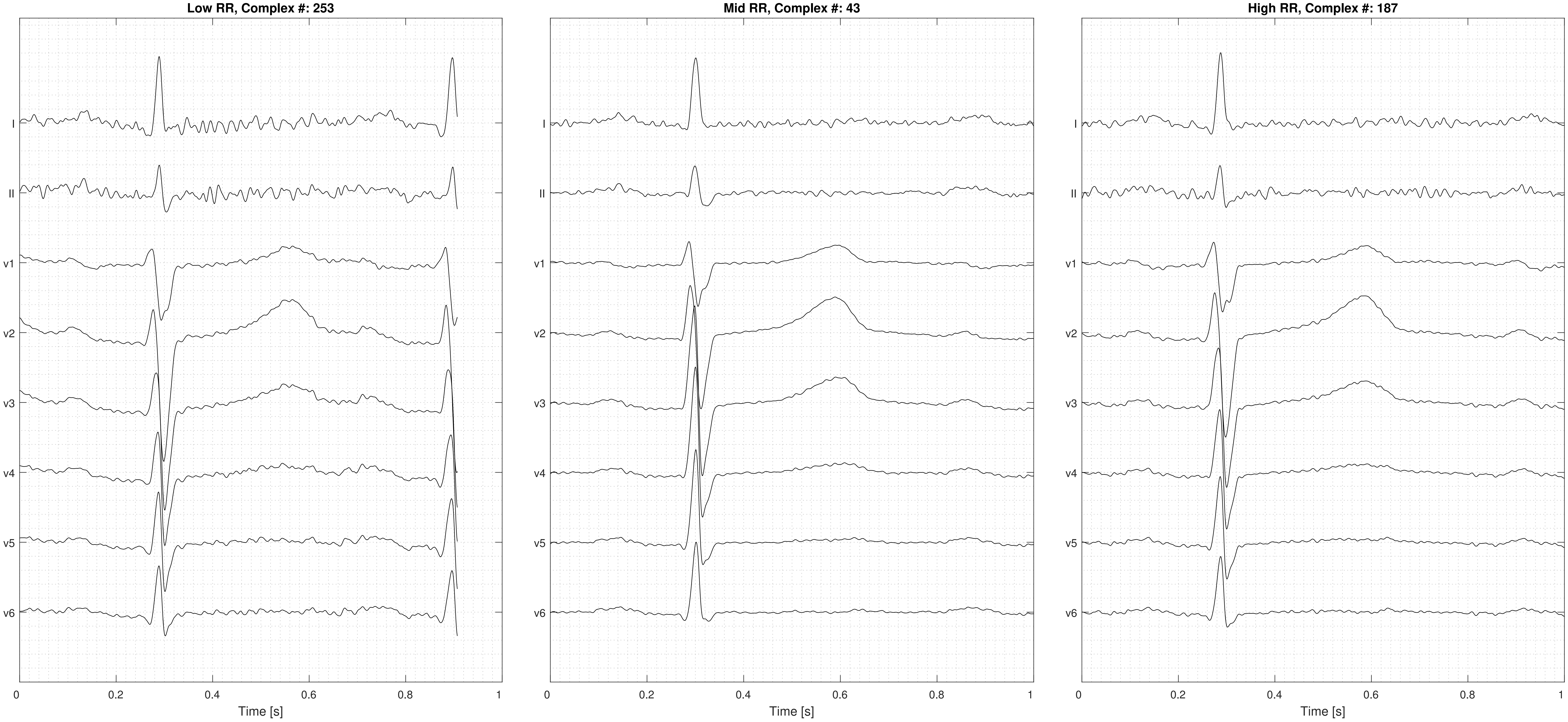
Low RR, Complex #: 253
Mid RR, Complex #: 43
High RR, Complex #: 187
I
II
v1
v2
v3
v4
v5
v6
0
0.2
0.4
0.6
0.8
1
Time [s]

Patient 69

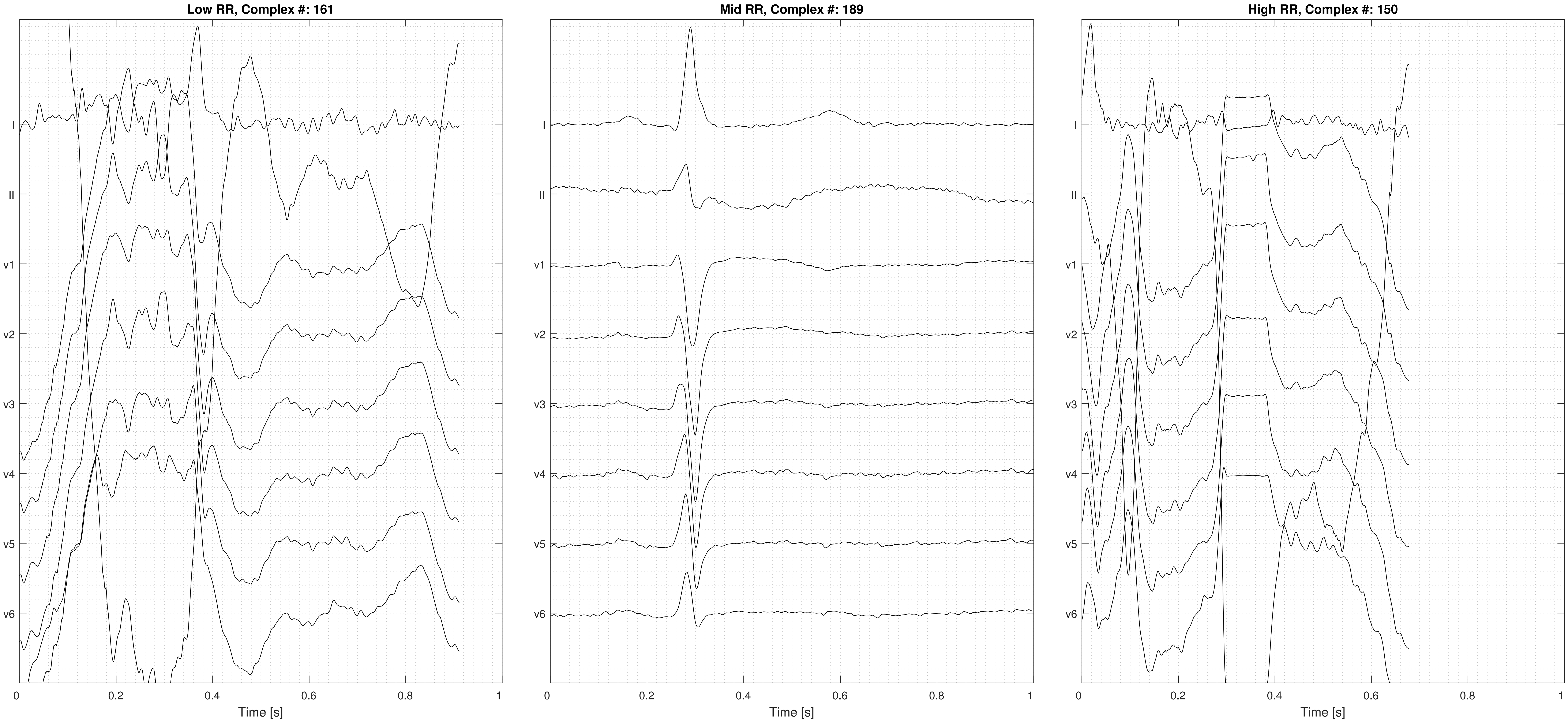

Low RR, Complex #: 161
Mid RR, Complex #: 189
High RR, Complex #: 150
I
II
v1
v2
v3
v4
v5
v6
0
0.2
0.4
0.6
0.8
1
Time [s]

Patient 70

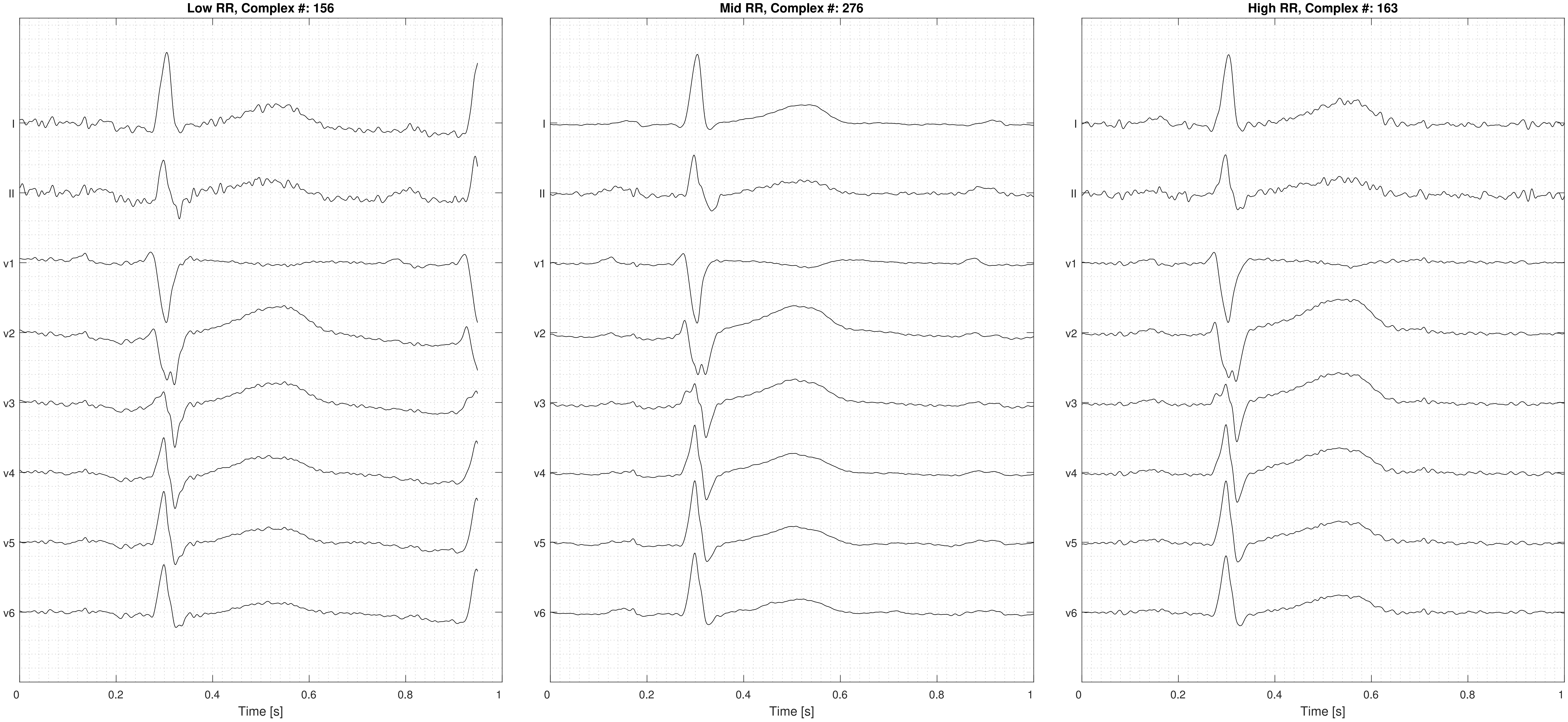

Low RR, Complex #: 156
Mid RR, Complex #: 276
High RR, Complex #: 163
I
II
v1
v2
v3
v4
v5
v6
0
0.2
0.4
0.6
0.8
1
Time [s]

Patient 71

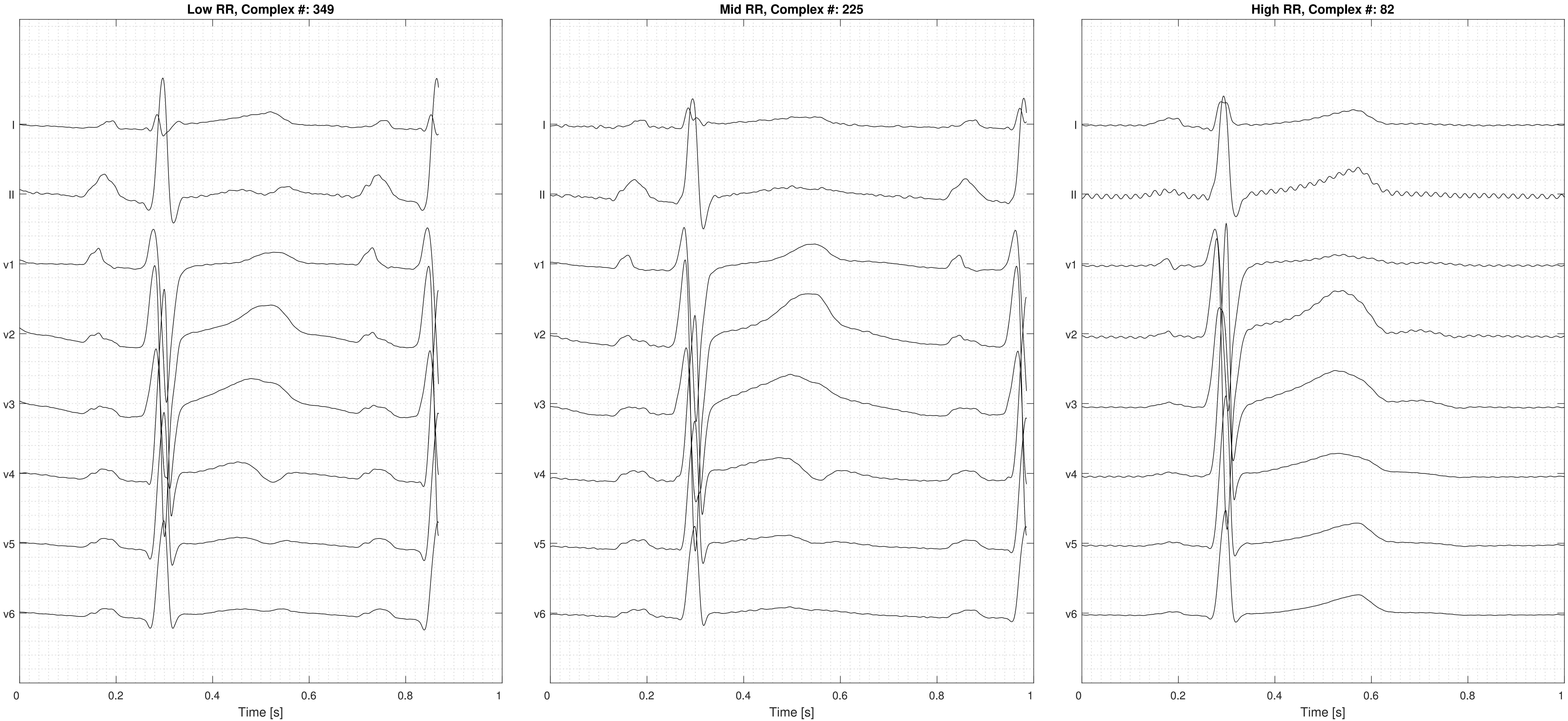

Low RR, Complex #: 349
Mid RR, Complex #: 225
High RR, Complex #: 82
I
II
v1
v2
v3
v4
v5
v6
0
0.2
0.4
0.6
0.8
1
Time [s]

Patient 72

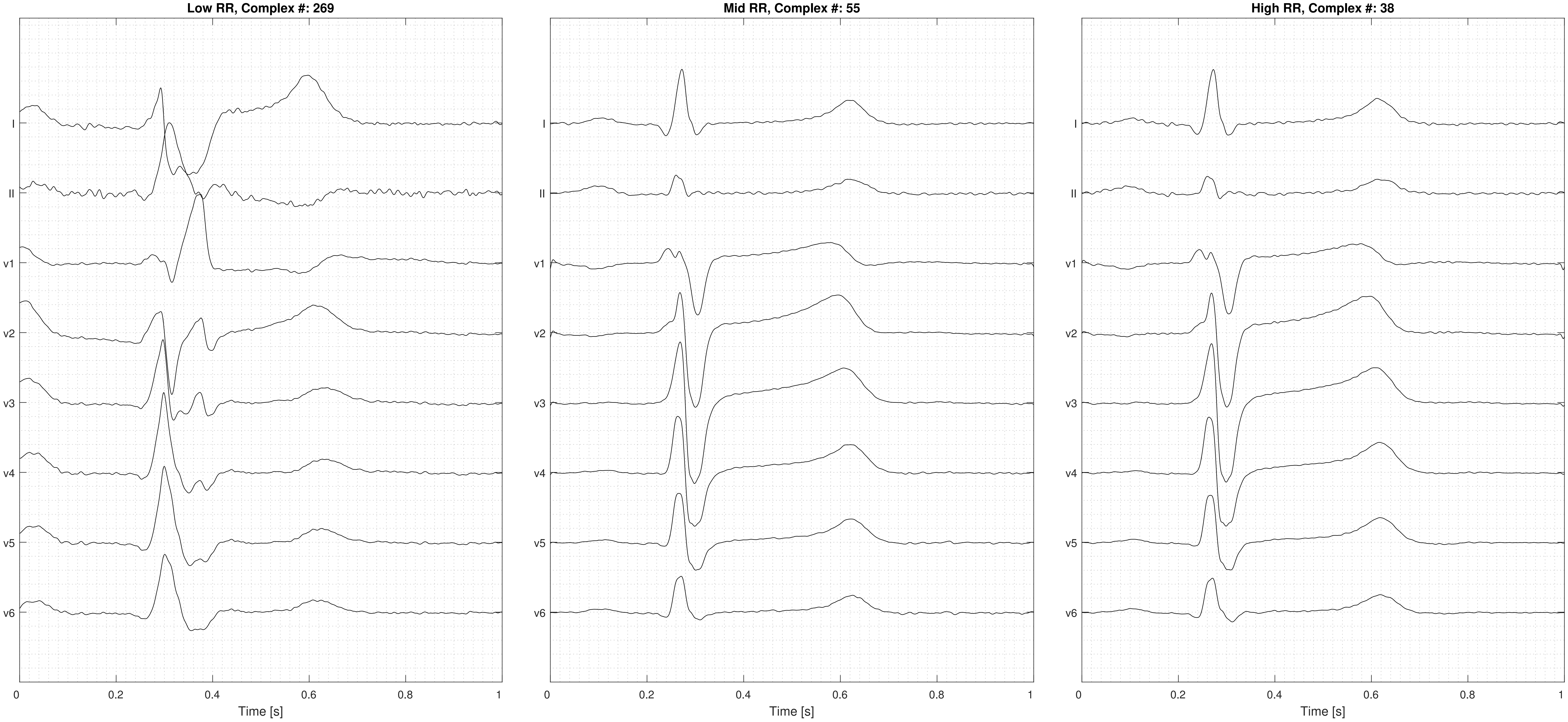

Low RR, Complex #: 269
Mid RR, Complex #: 55
High RR, Complex #: 38
I
II
v1
v2
v3
v4
v5
v6
0
0.2
0.4
0.6
0.8
1
Time [s]

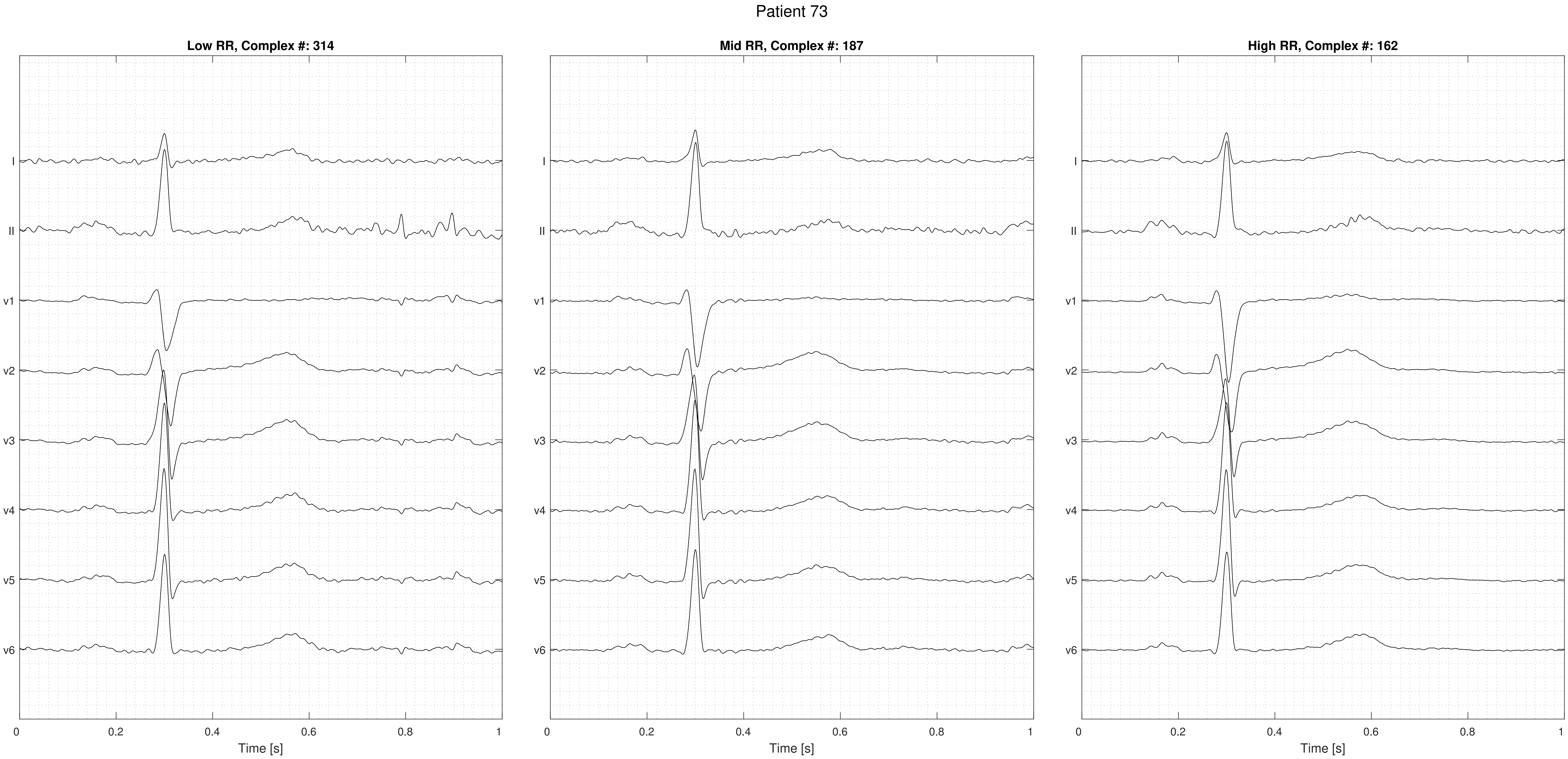
Patient 73
Low RR, Complex #: 314
Mid RR, Complex #: 187
High RR, Complex #: 162
I
II
v1
v2
v3
v4
v5
v6
0
0.2
0.4
0.6
0.8
1
Time [s]

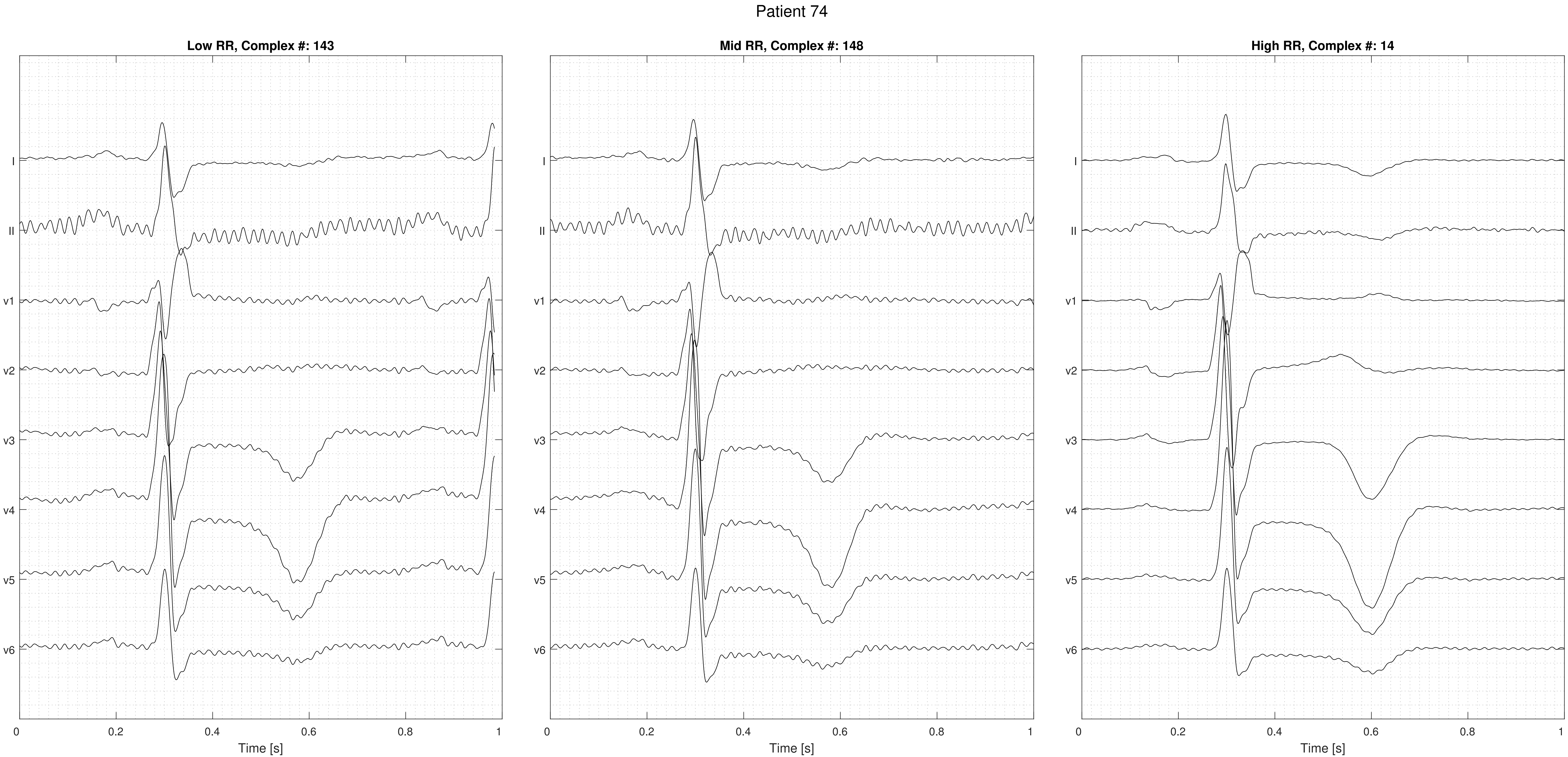

Patient 74
Low RR, Complex #: 143
Mid RR, Complex #: 148
High RR, Complex #: 14
I
II
v1
v2
v3
v4
v5
v6
0
0.2
0.4
0.6
0.8
1
Time [s]

Patient 75

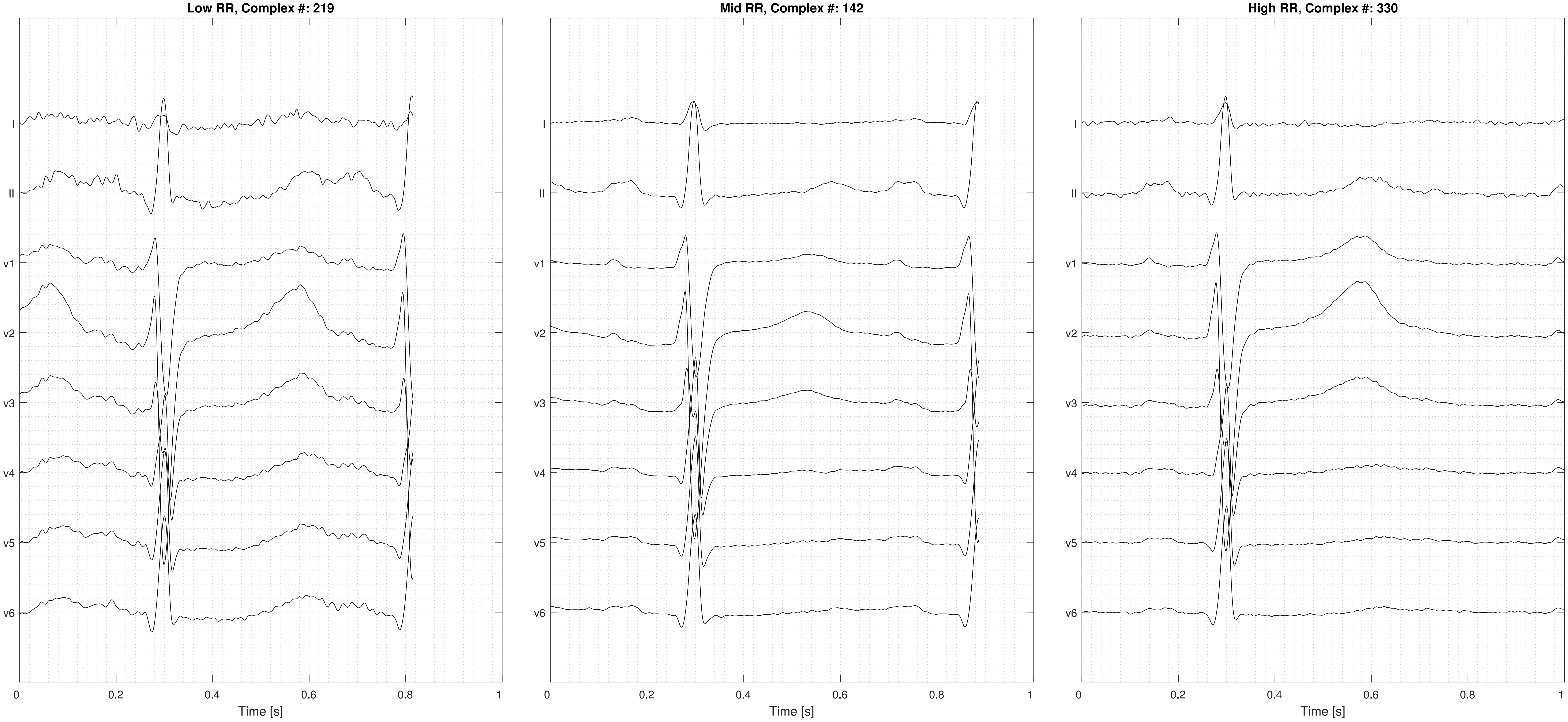

Low RR, Complex #: 219
Mid RR, Complex #: 142
High RR, Complex #: 330
I
II
v1
v2
v3
v4
v5
v6
0
0.2
0.4
0.6
0.8
1
Time [s]

Patient 76

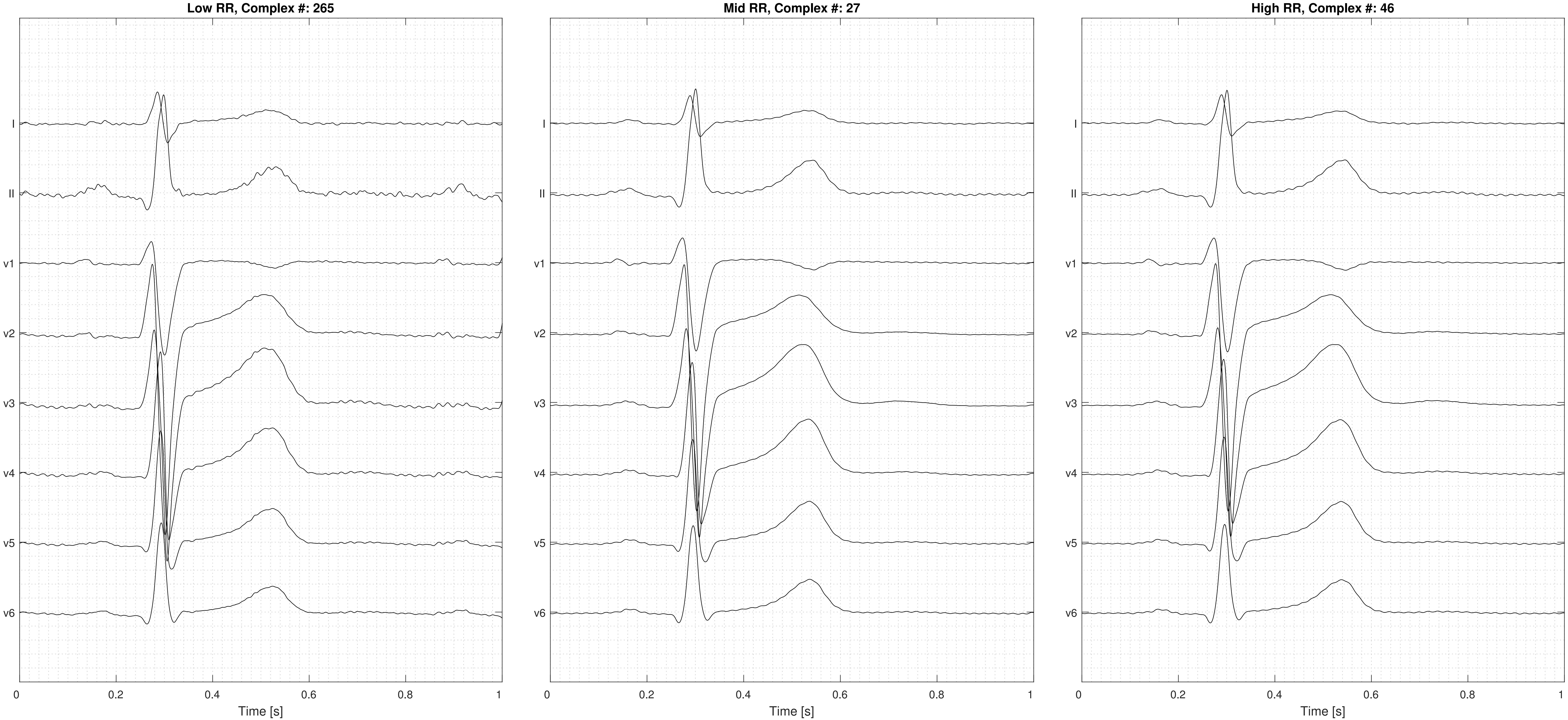

Low RR, Complex #: 265
Mid RR, Complex #: 27
High RR, Complex #: 46
I
II
v1
v2
v3
v4
v5
v6
0
0.2
0.4
0.6
0.8
1
Time [s]

Patient 77

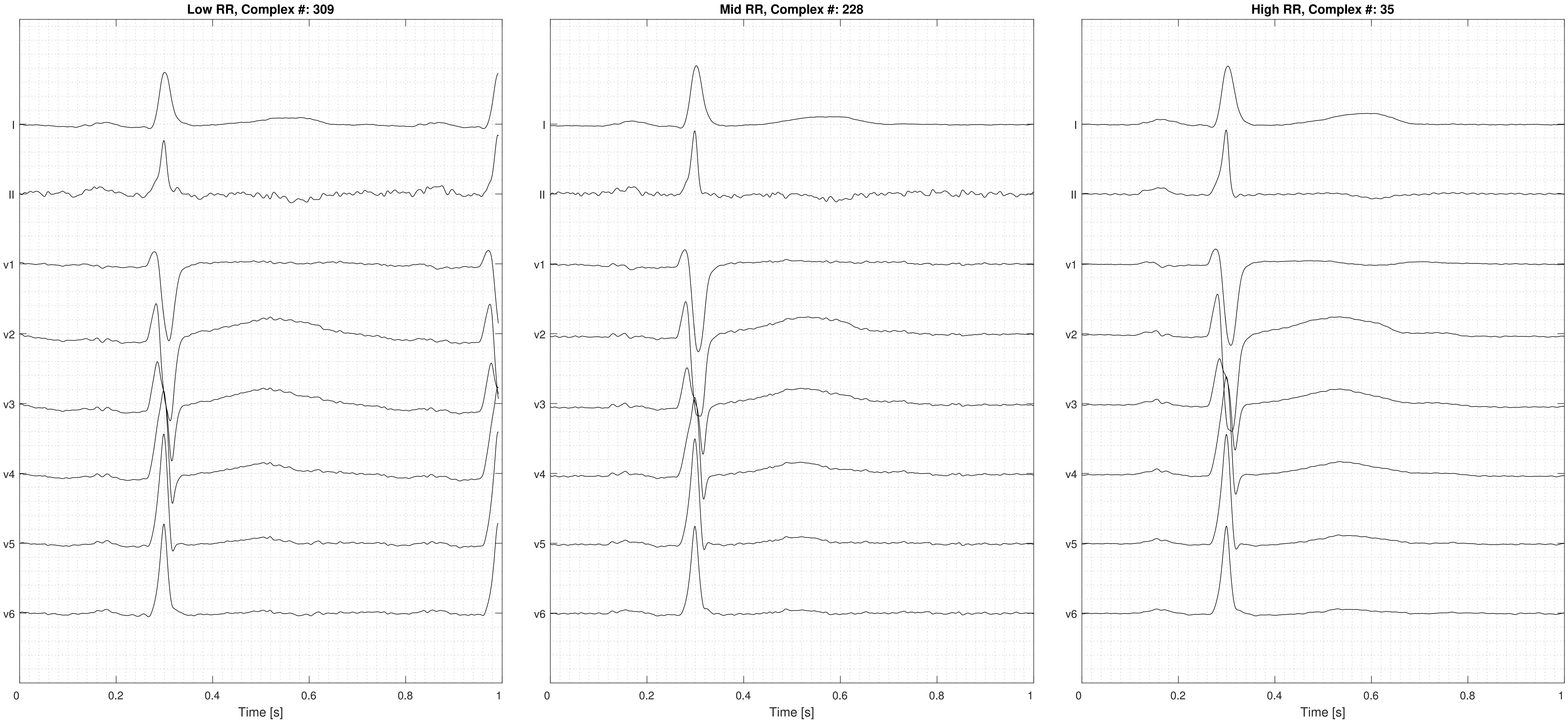

Low RR, Complex #: 309
Mid RR, Complex #: 228
High RR, Complex #: 35
I
II
v1
v2
v3
v4
v5
v6
0
0.2
0.4
0.6
0.8
1
Time [s]

Patient 78

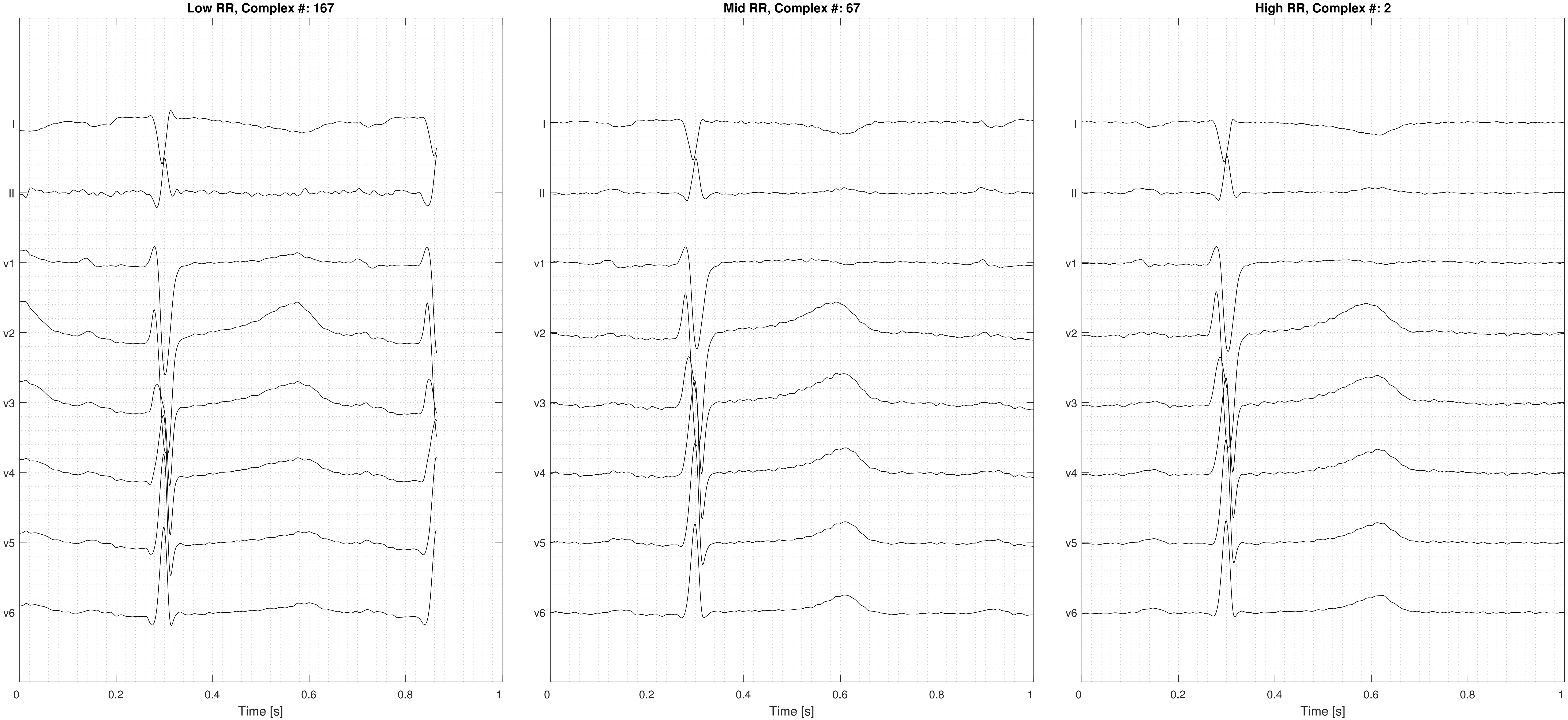

Low RR, Complex #: 167
Mid RR, Complex #: 67
High RR, Complex #: 2
I
II
v1
v2
v3
v4
v5
v6
0
0.2
0.4
0.6
0.8
1
Time [s]

Patient 79

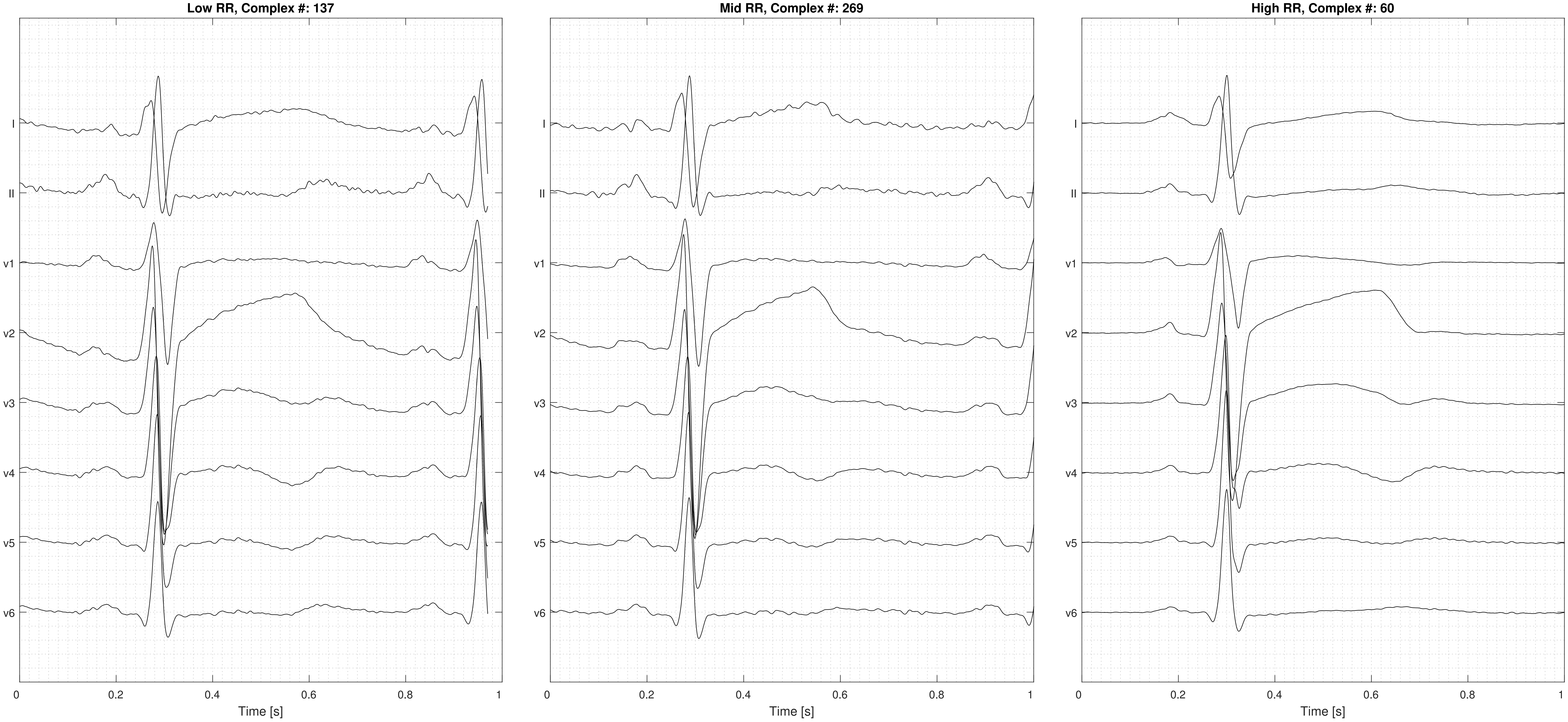

Low RR, Complex #: 137
Mid RR, Complex #: 269
High RR, Complex #: 60
I
II
v1
v2
v3
v4
v5
v6
0
0.2
0.4
0.6
0.8
1
Time [s]

Patient 80

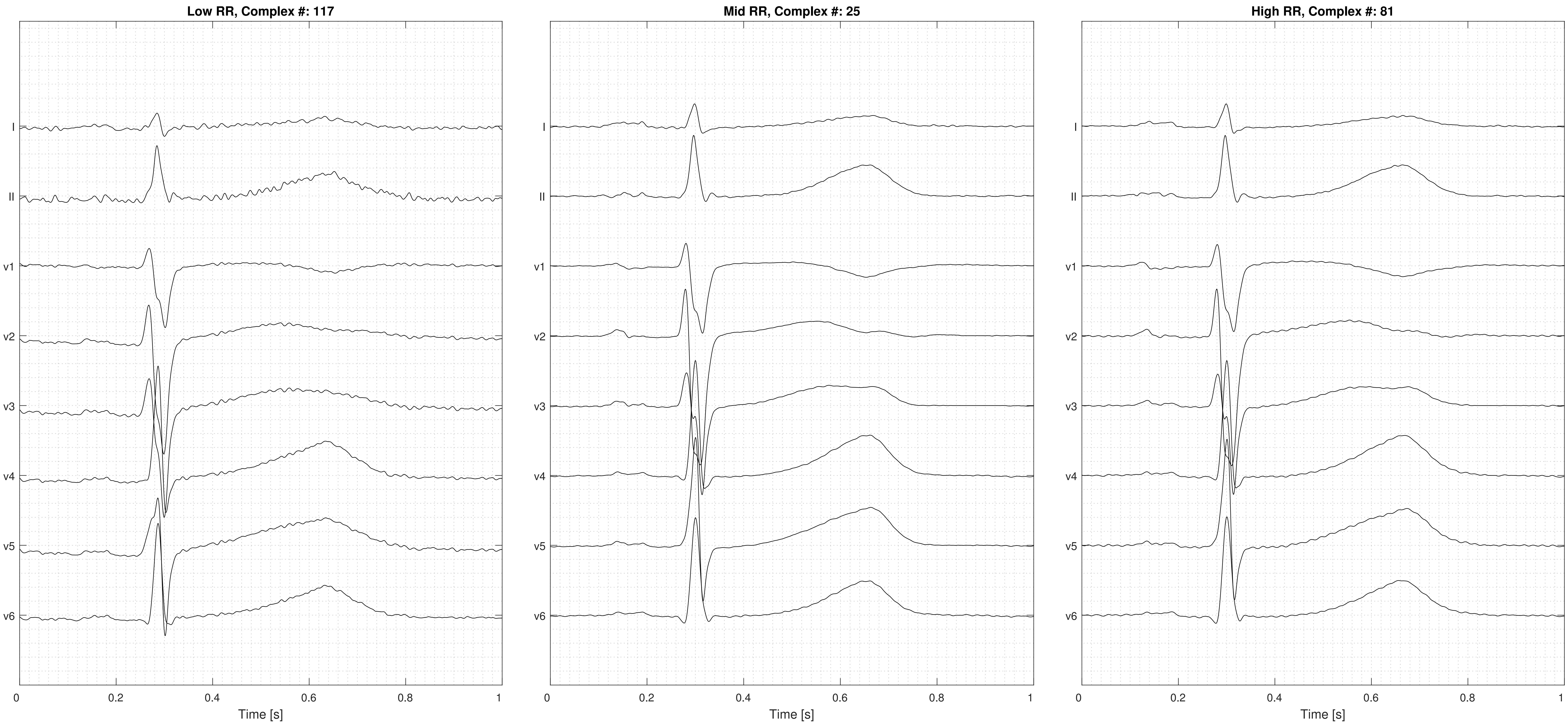
Low RR, Complex #: 117
Mid RR, Complex #: 25
High RR, Complex #: 81
I
II
v1
v2
v3
v4
v5
v6
0
0.2
0.4
0.6
0.8
1
Time [s]

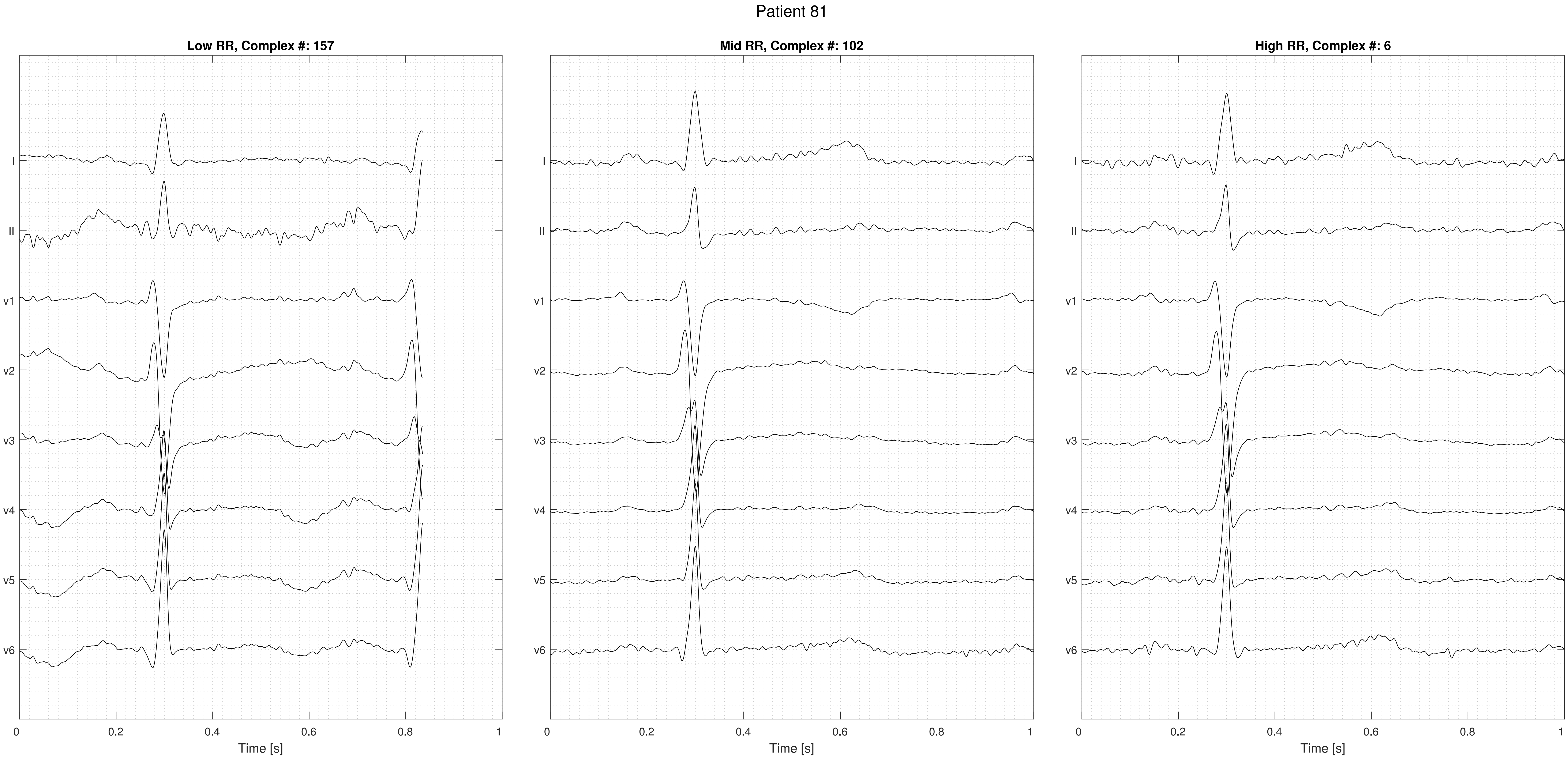

Patient 81
Low RR, Complex #: 157
Mid RR, Complex #: 102
High RR, Complex #: 6
I
II
v1
v2
v3
v4
v5
v6
0
0.2
0.4
0.6
0.8
1
Time [s]

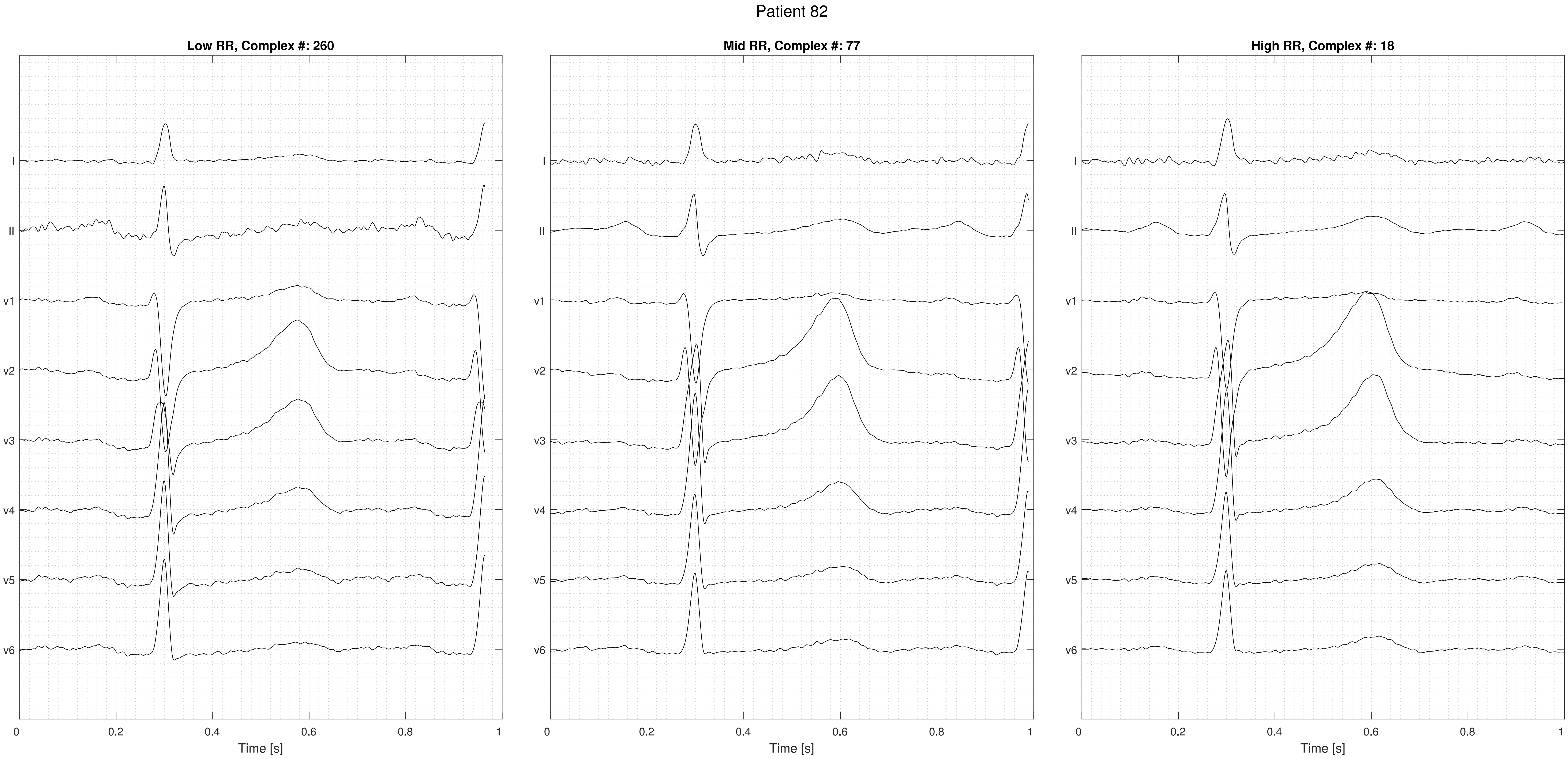
Patient 82
Low RR, Complex #: 260
Mid RR, Complex #: 77
High RR, Complex #: 18
I
II
v1
v2
v3
v4
v5
v6
0
0.2
0.4
0.6
0.8
1
Time [s]

Patient 83

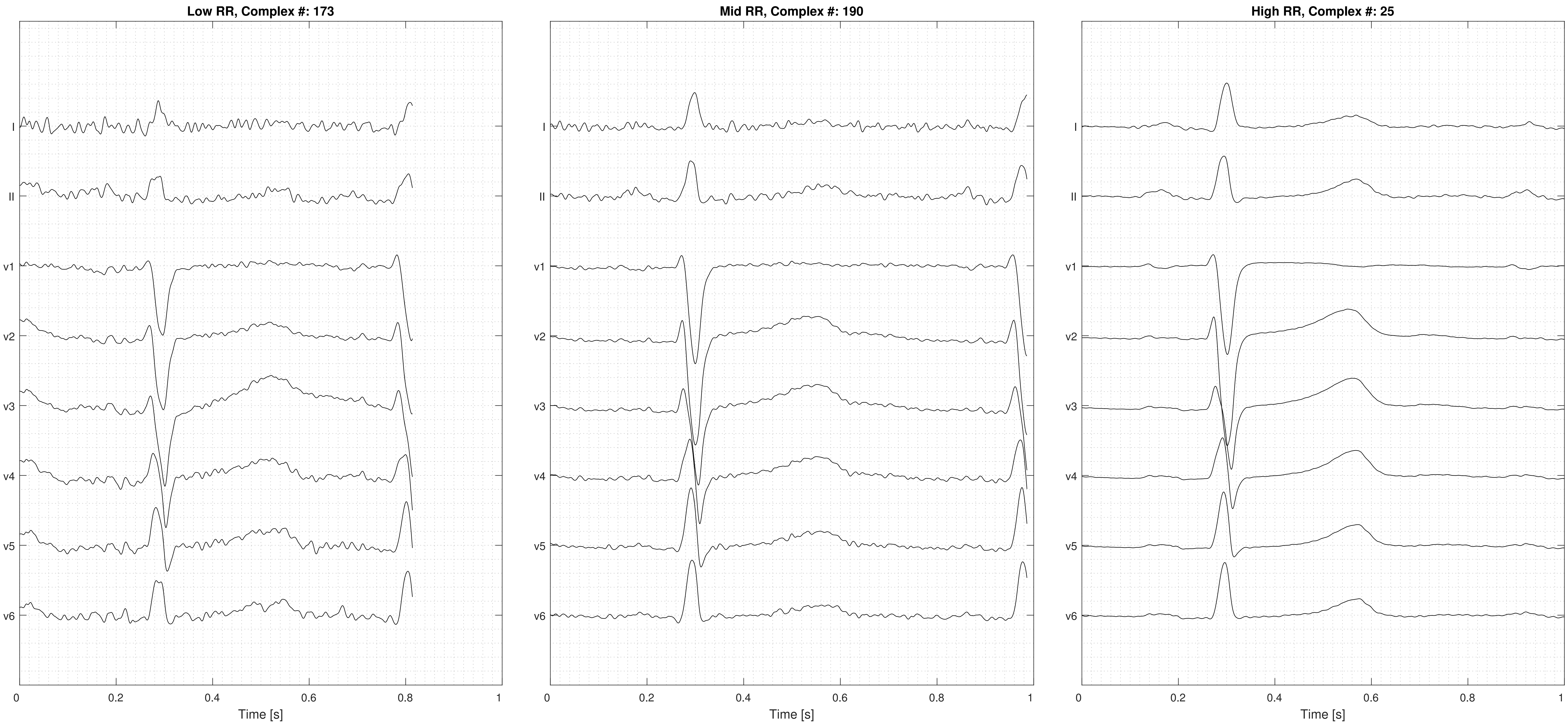

Low RR, Complex #: 173
Mid RR, Complex #: 190
High RR, Complex #: 25
I
II
v1
v2
v3
v4
v5
v6
0
0.2
0.4
0.6
0.8
1
Time [s]

Patient 84

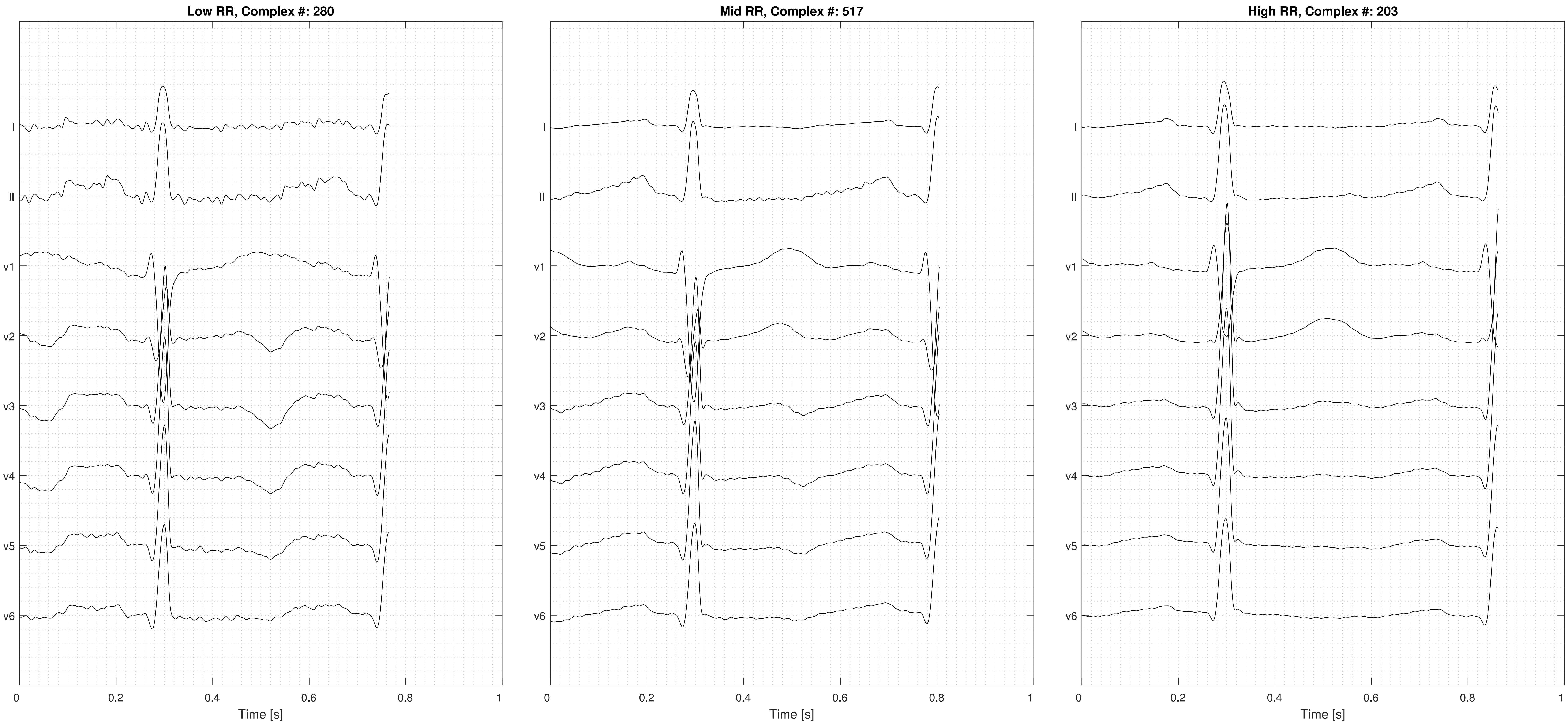
Low RR, Complex #: 280
Mid RR, Complex #: 517
High RR, Complex #: 203
I
II
v1
v2
v3
v4
v5
v6
0
0.2
0.4
0.6
0.8
1
Time [s]

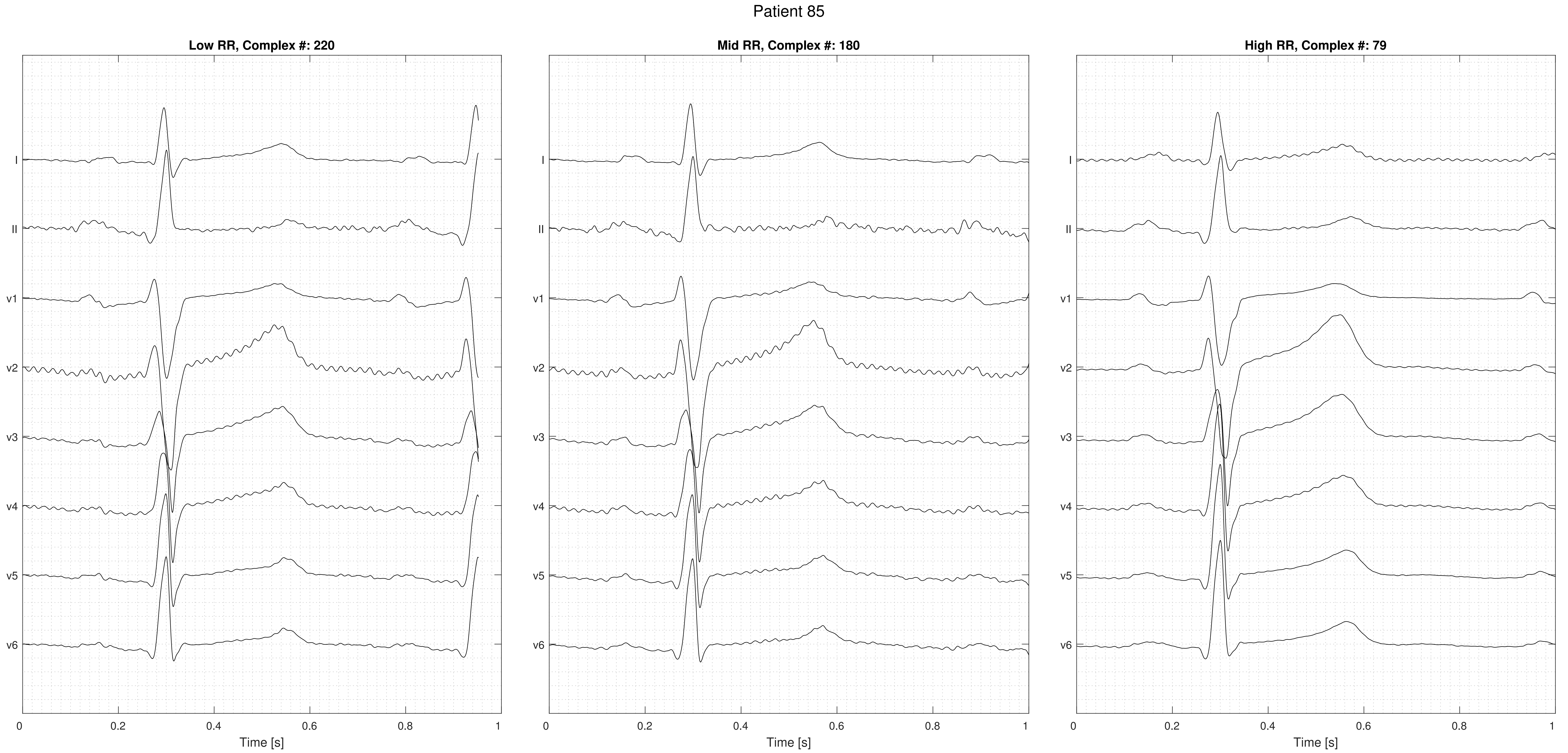
Patient 85
Low RR, Complex #: 220
Mid RR, Complex #: 180
High RR, Complex #: 79
I
II
v1
v2
v3
v4
v5
v6
0
0.2
0.4
0.6
0.8
1
Time [s]

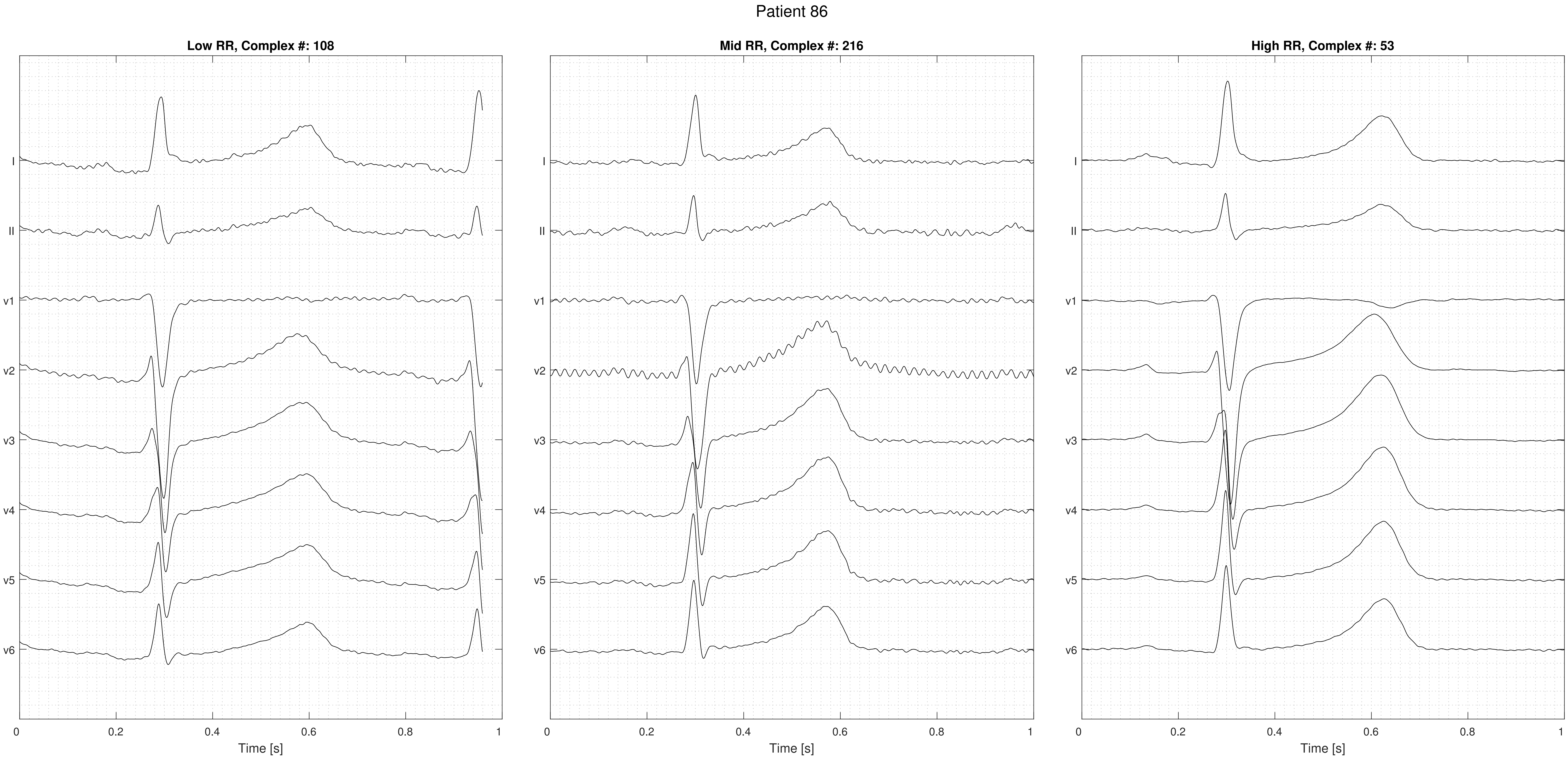
Patient 86
Low RR, Complex #: 108
Mid RR, Complex #: 216
High RR, Complex #: 53
I
II
v1
v2
v3
v4
v5
v6
0
0.2
0.4
0.6
0.8
1
Time [s]

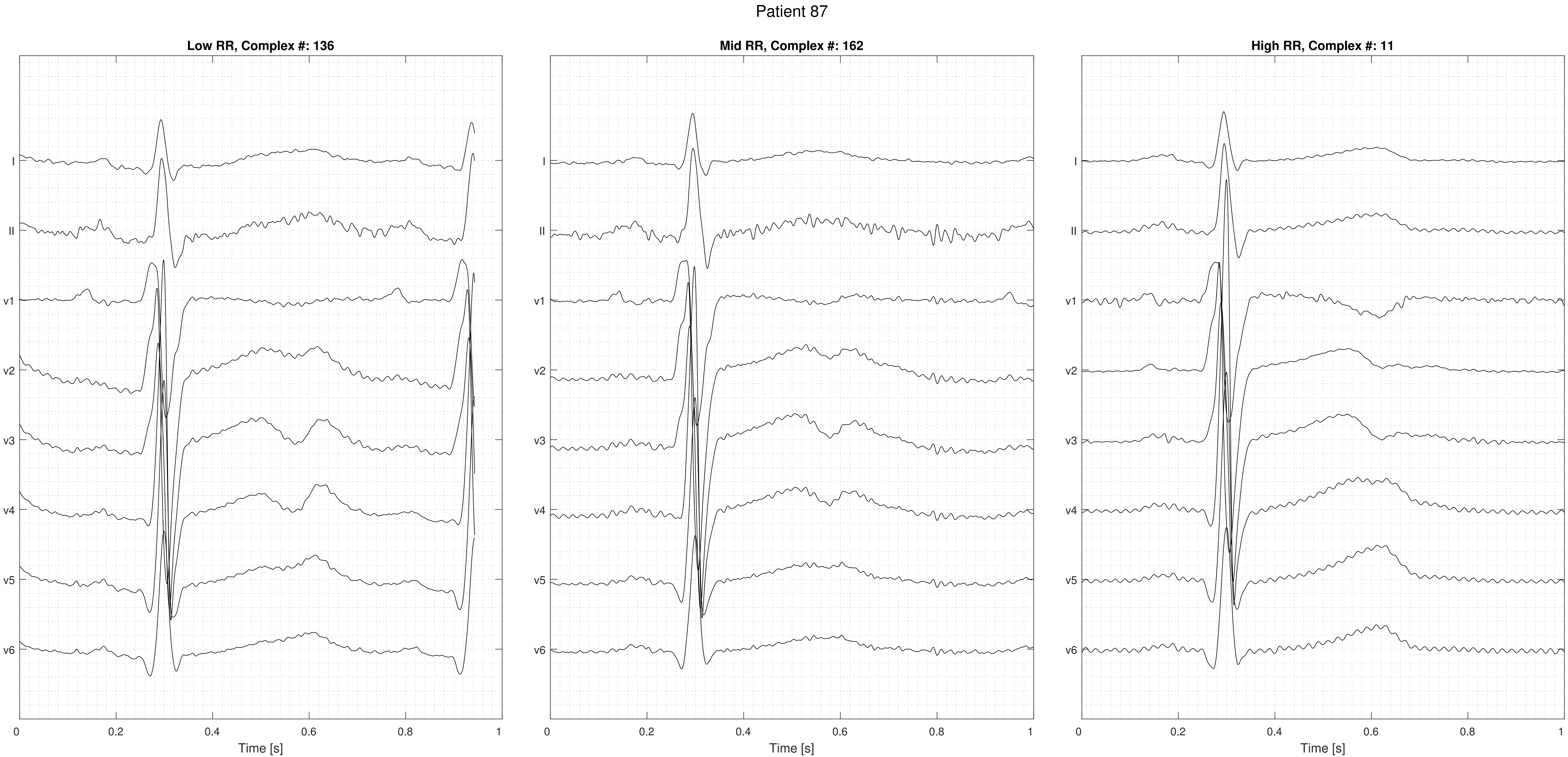
Patient 87
Low RR, Complex #: 136
Mid RR, Complex #: 162
High RR, Complex #: 11
I
II
v1
v2
v3
v4
v5
v6
0
0.2
0.4
0.6
0.8
1
Time [s]

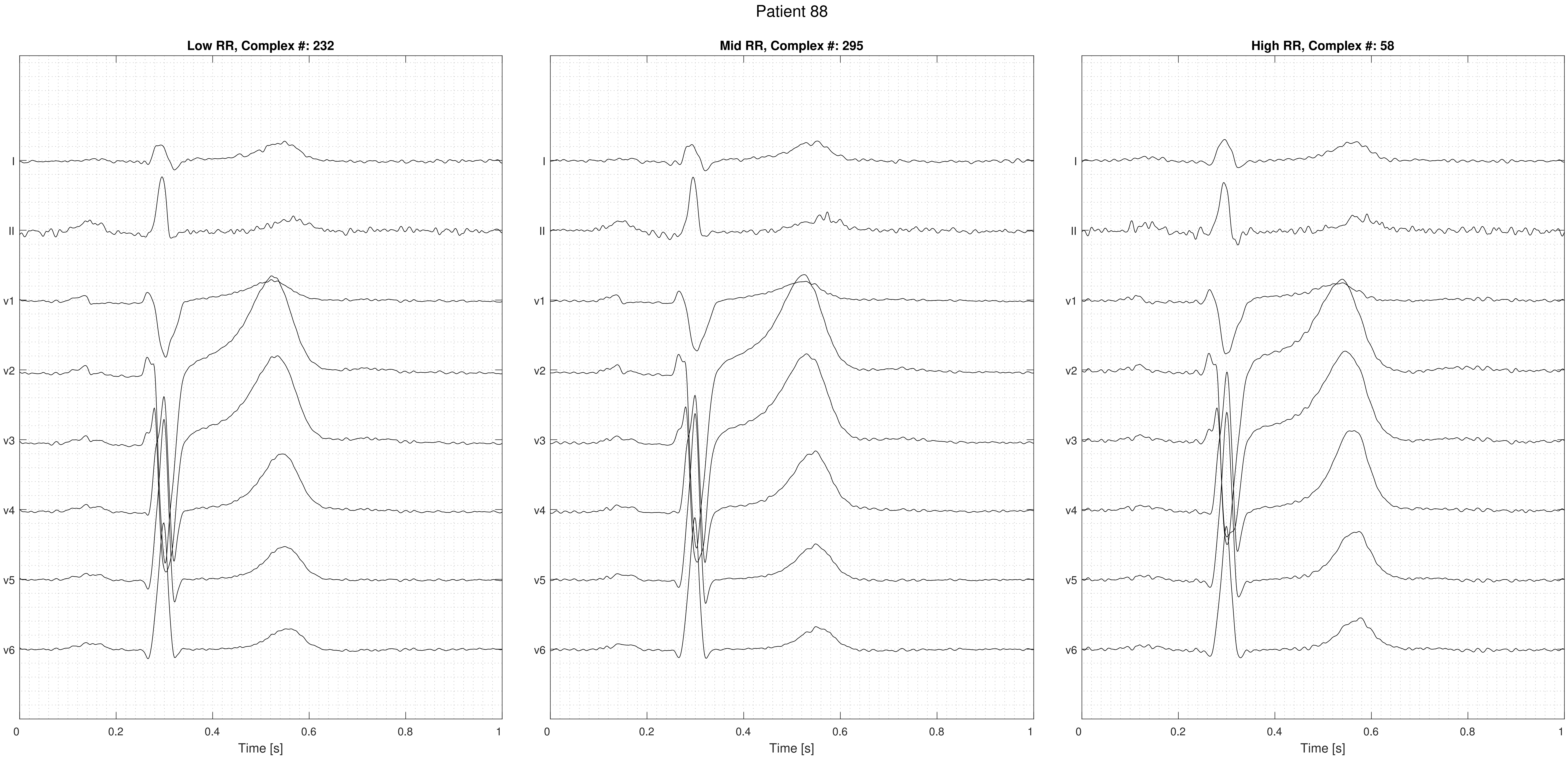
Patient 88
Low RR, Complex #: 232
Mid RR, Complex #: 295
High RR, Complex #: 58
I
II
v1
v2
v3
v4
v5
v6
0
0.2
0.4
0.6
0.8
1
Time [s]

Patient 89

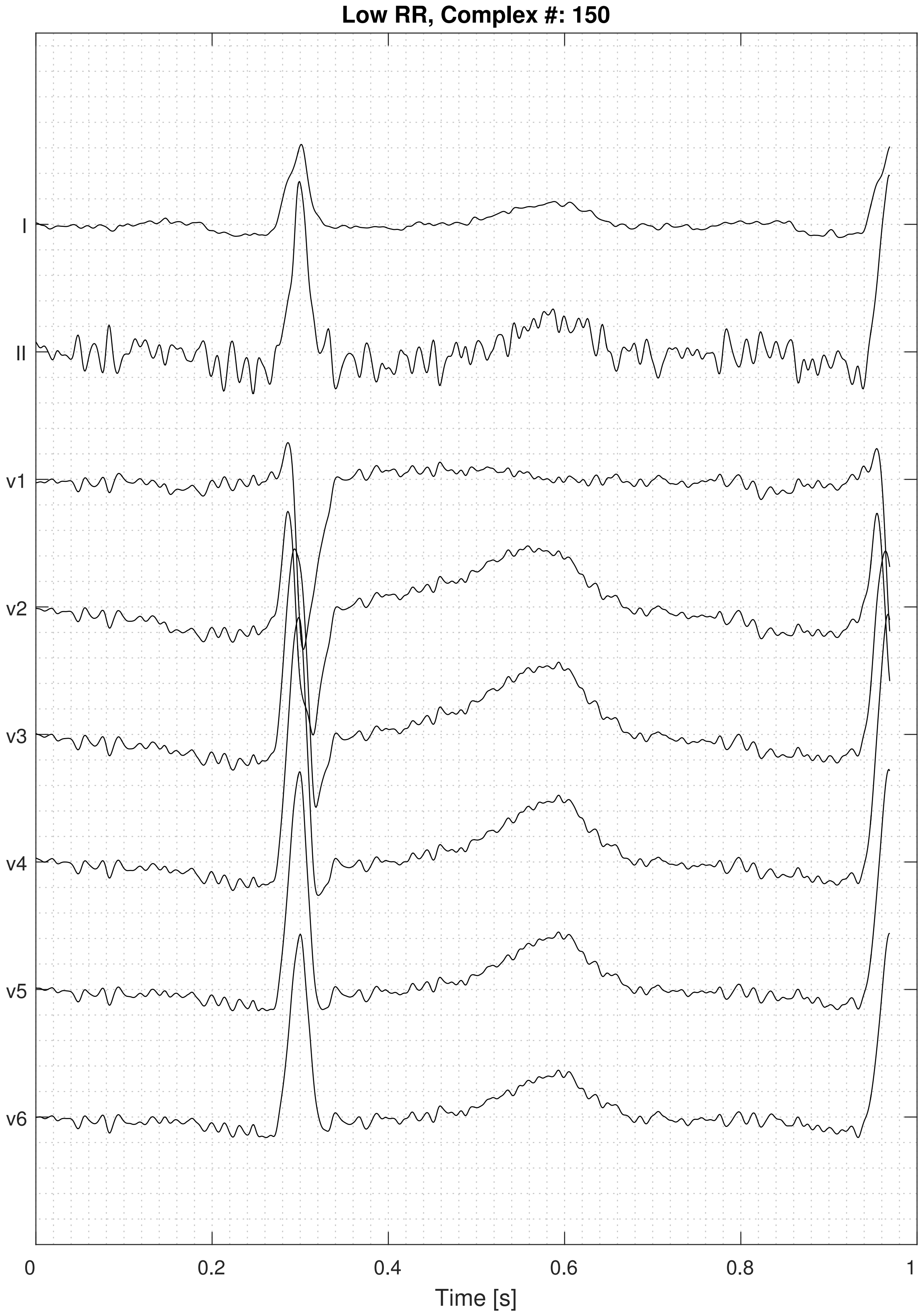

Low RR, Complex #: 150
I
II
v1
v2
v3
v4
v5
v6
0
0.2
0.4
0.6
0.8
1
Time [s]

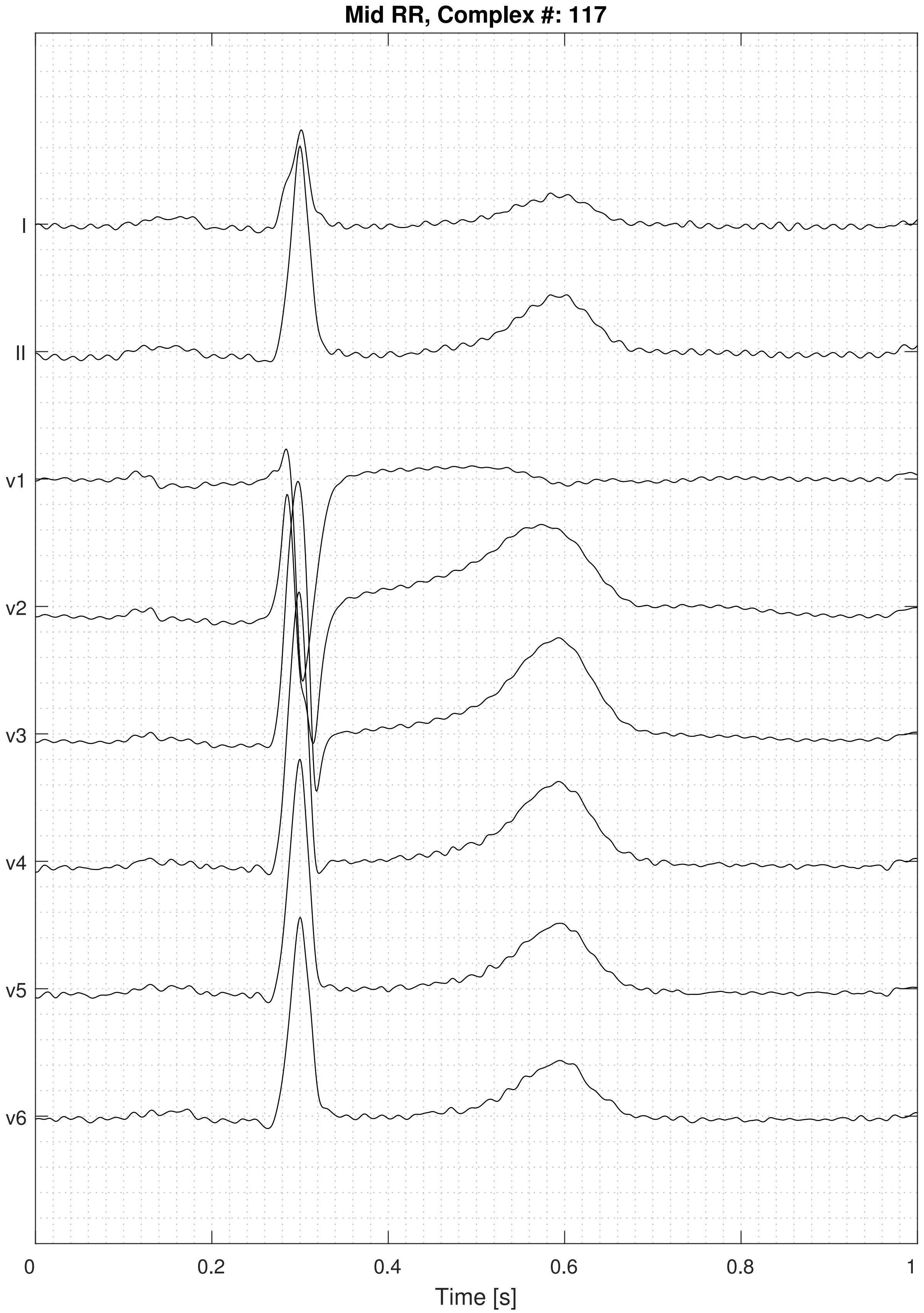

Mid RR, Complex #: 117
I
II
v1
v2
v3
v4
v5
v6
0
0.2
0.4
0.6
0.8
1
Time [s]

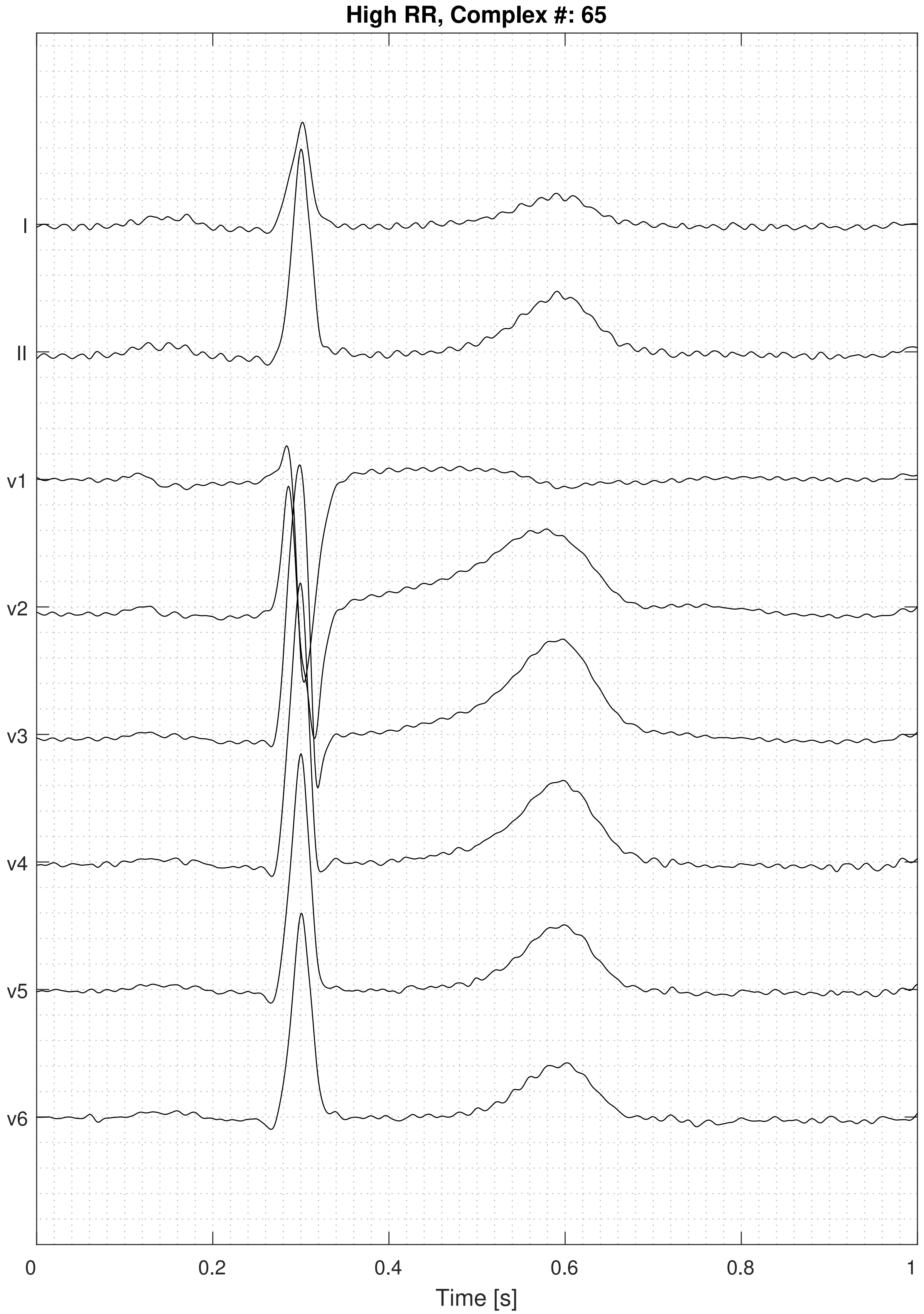

High RR, Complex #: 65
I
II
v1
v2
v3
v4
v5
v6
0
0.2
0.4
0.6
0.8
1
Time [s]

Patient 90

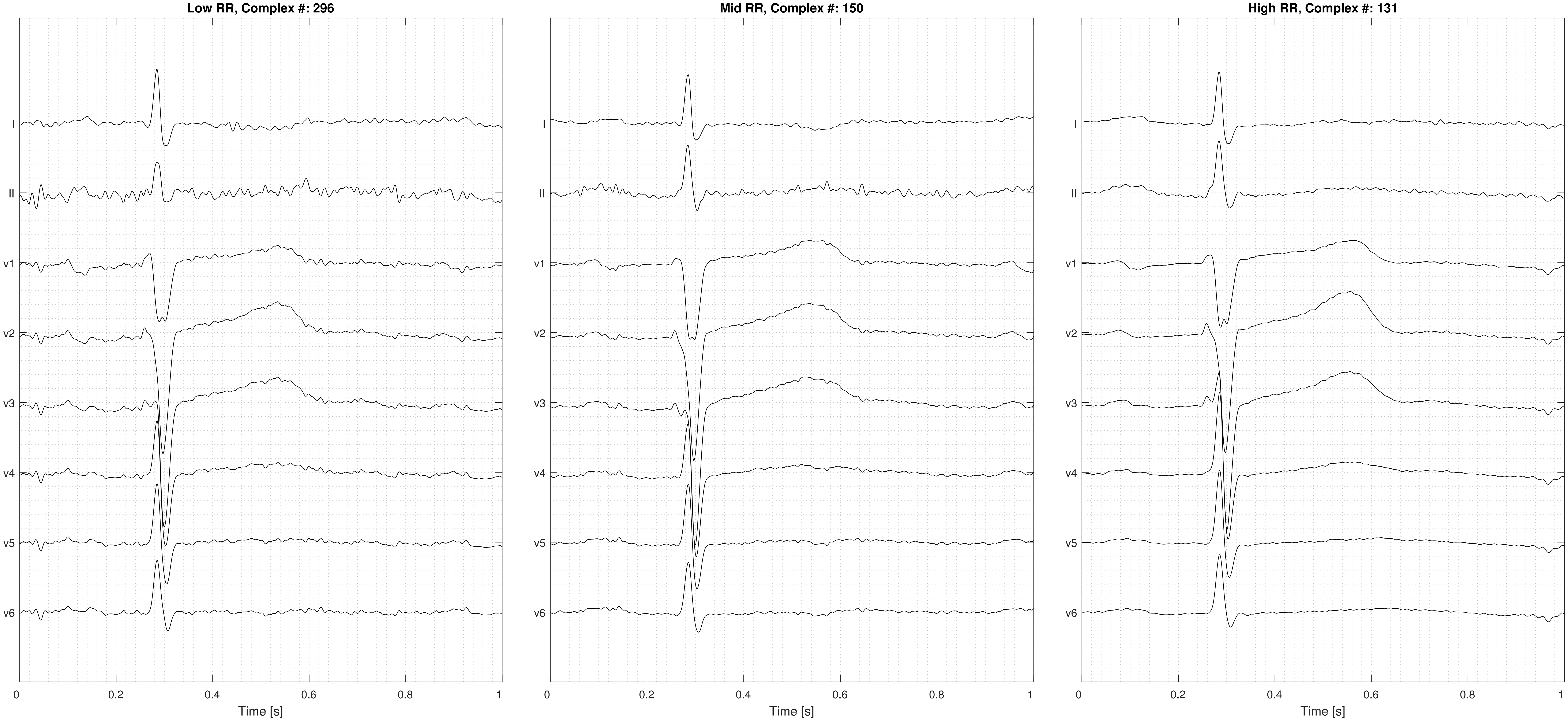
Low RR, Complex #: 296
Mid RR, Complex #: 150
High RR, Complex #: 131
I
II
v1
v2
v3
v4
v5
v6
0
0.2
0.4
0.6
0.8
1
Time [s]

Patient 91

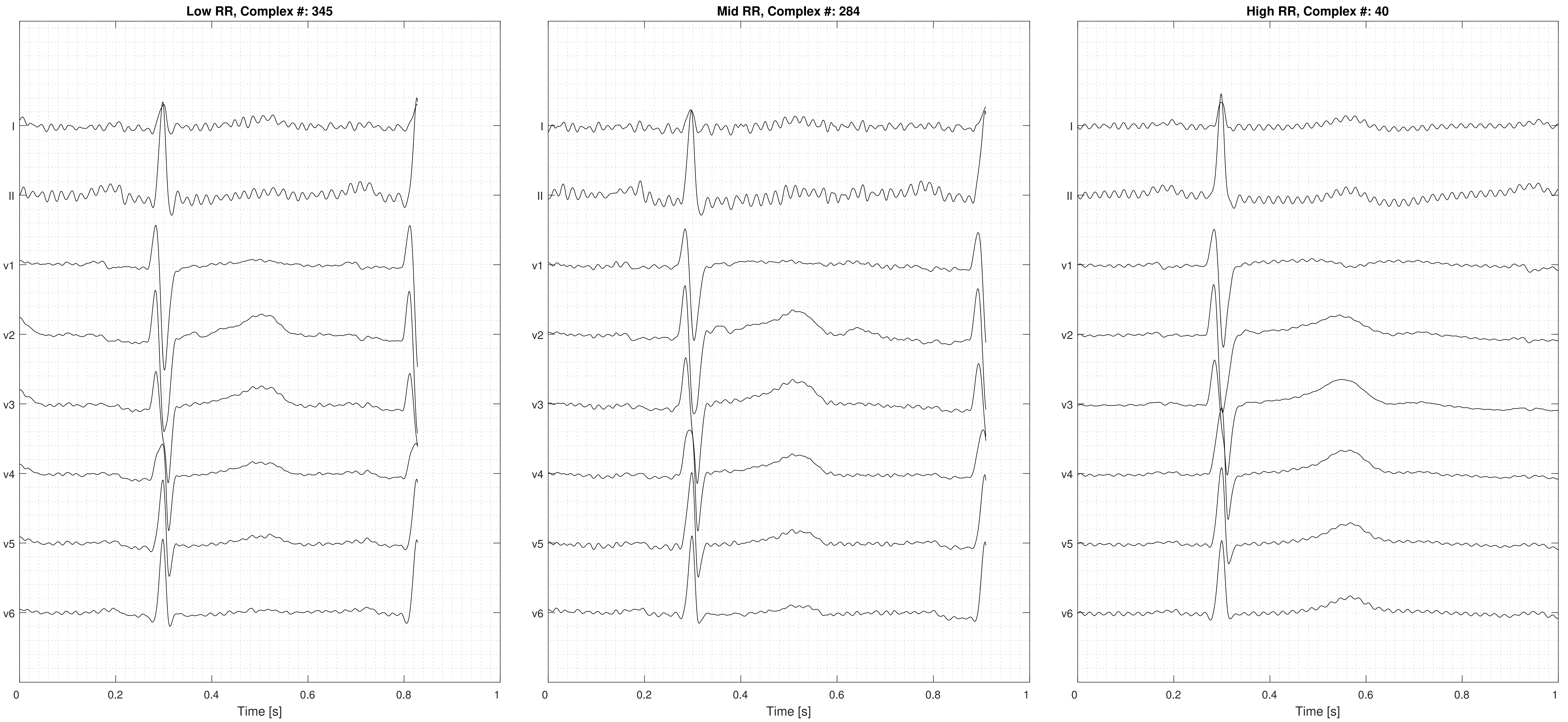

Low RR, Complex #: 345
Mid RR, Complex #: 284
High RR, Complex #: 40
I
II
v1
v2
v3
v4
v5
v6
0
0.2
0.4
0.6
0.8
1
Time [s]

Patient 92

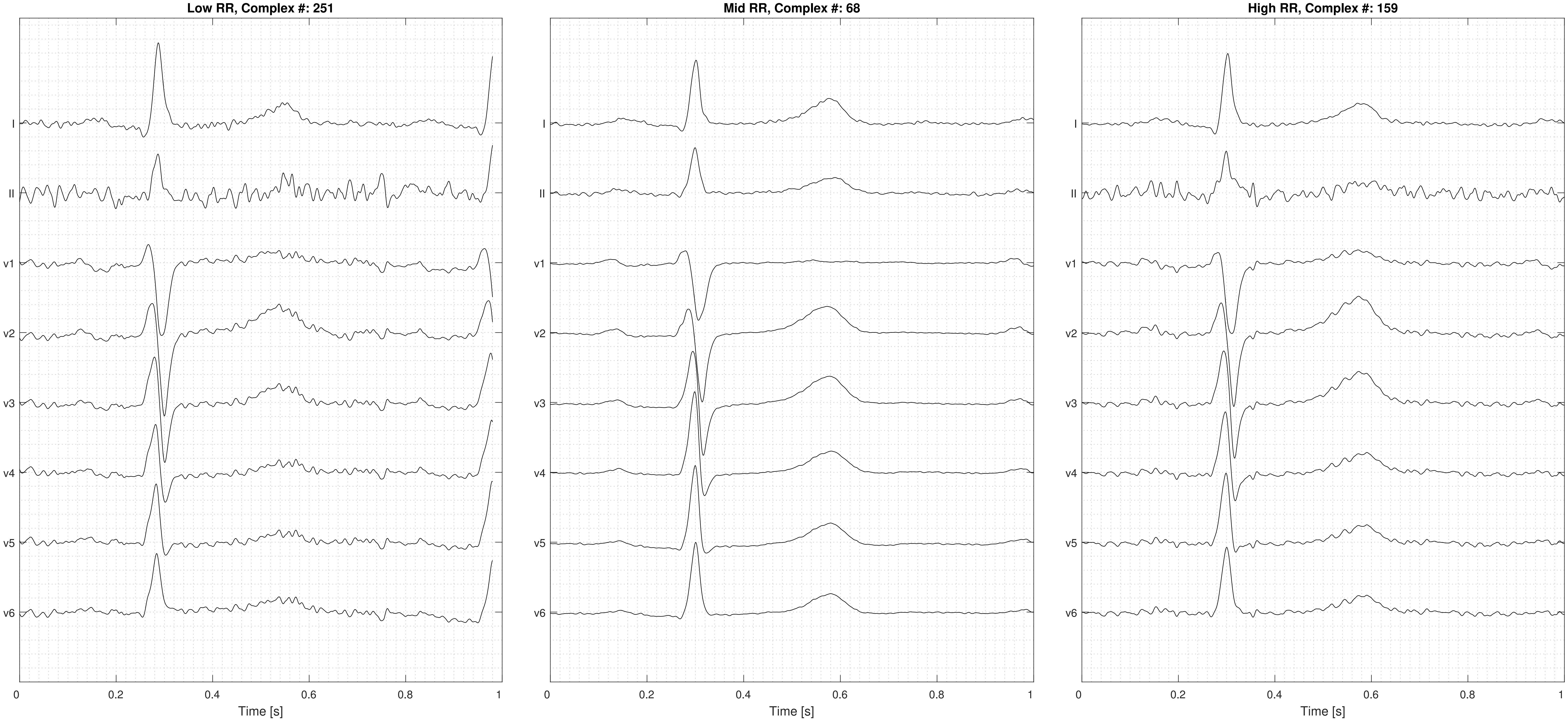

Low RR, Complex #: 251
Mid RR, Complex #: 68
High RR, Complex #: 159
I
II
v1
v2
v3
v4
v5
v6
0
0.2
0.4
0.6
0.8
1
Time [s]

Patient 93

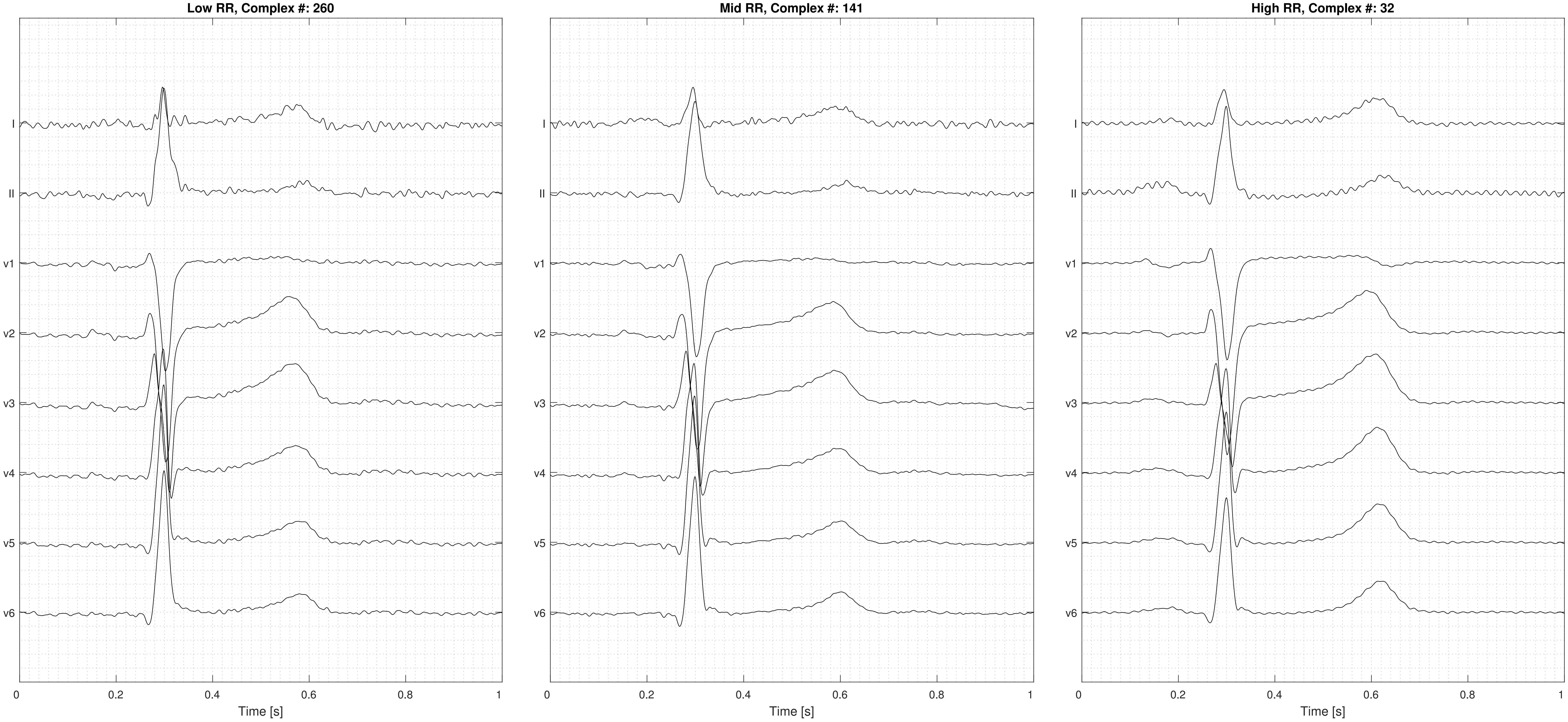

Low RR, Complex #: 260
Mid RR, Complex #: 141
High RR, Complex #: 32
I
II
v1
v2
v3
v4
v5
v6
0
0.2
0.4
0.6
0.8
1
Time [s]

Patient 94

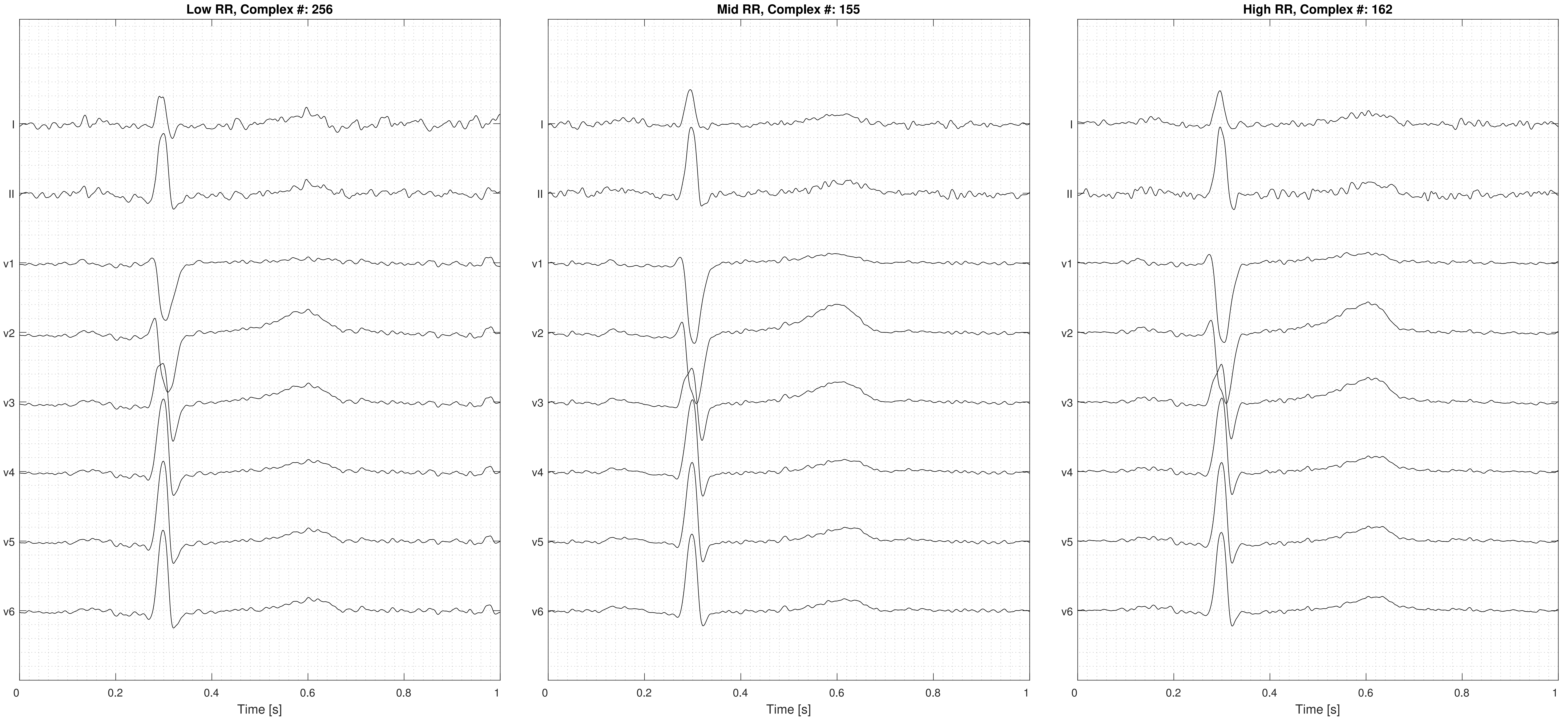

Low RR, Complex #: 256
Mid RR, Complex #: 155
High RR, Complex #: 162
I
II
v1
v2
v3
v4
v5
v6
0
0.2
0.4
0.6
0.8
1
Time [s]

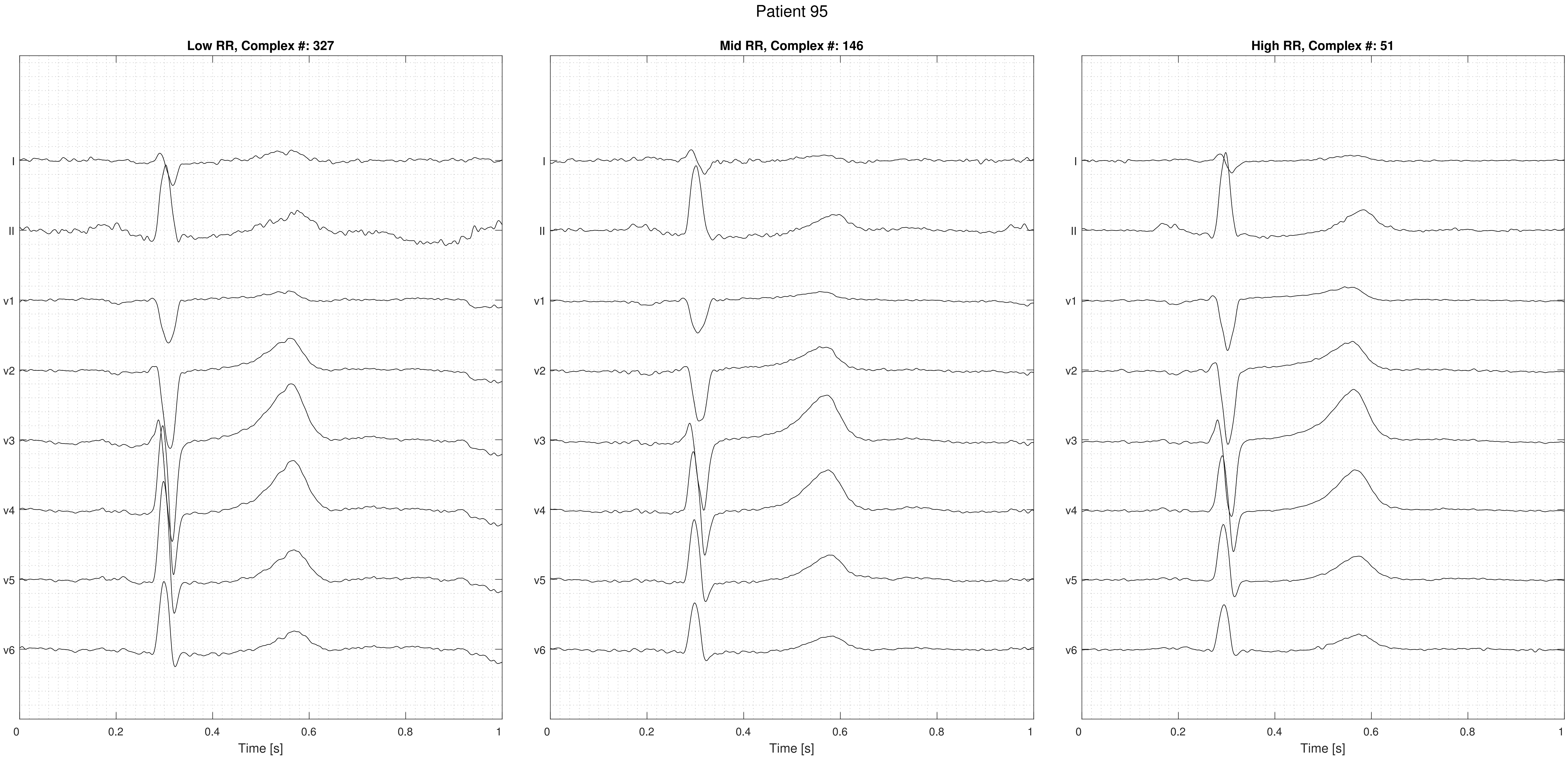

Patient 95
Low RR, Complex #: 327
Mid RR, Complex #: 146
High RR, Complex #: 51
I
II
v1
v2
v3
v4
v5
v6
0
0.2
0.4
0.6
0.8
1
Time [s]

Patient 96

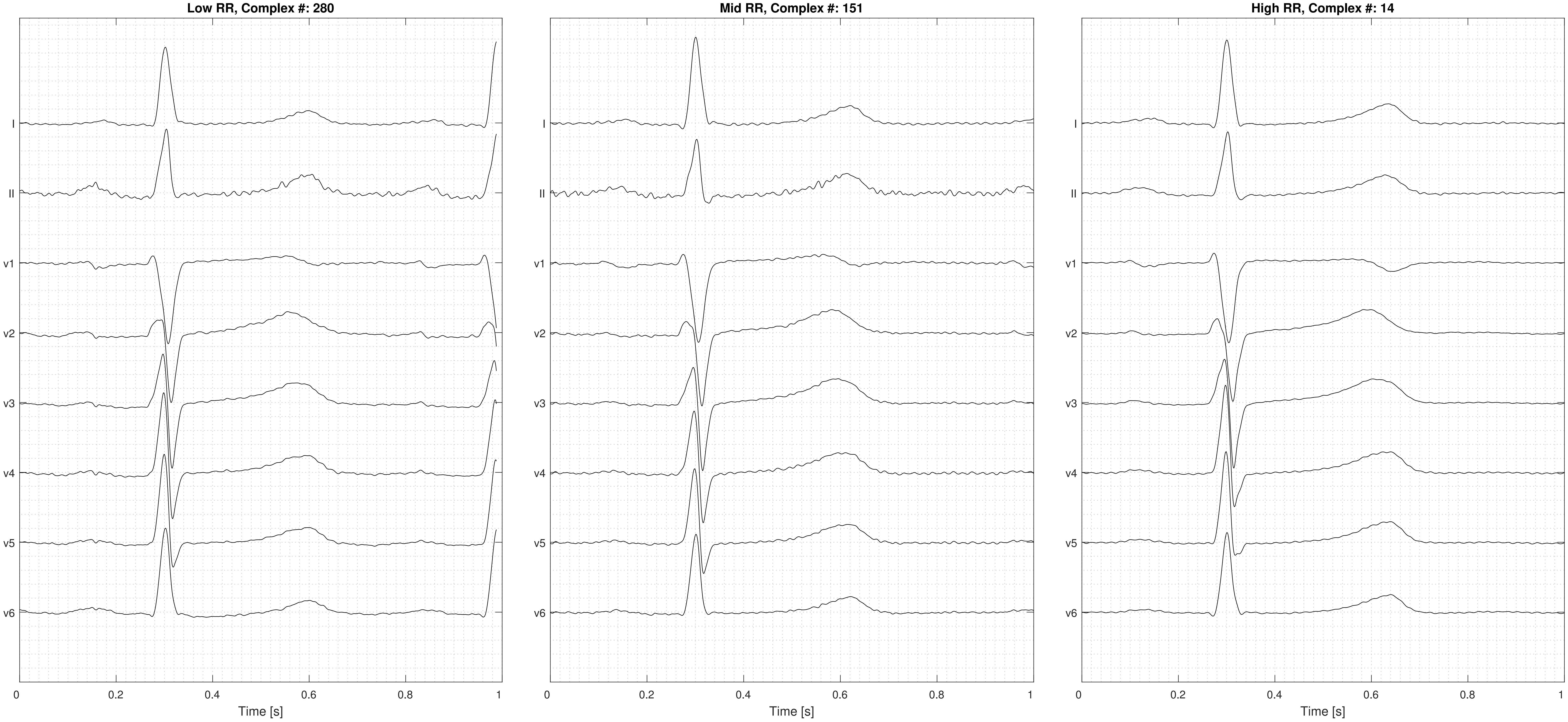

Low RR, Complex #: 280
Mid RR, Complex #: 151
High RR, Complex #: 14
I
II
v1
v2
v3
v4
v5
v6
0
0.2
0.4
0.6
0.8
1
Time [s]

Patient 97

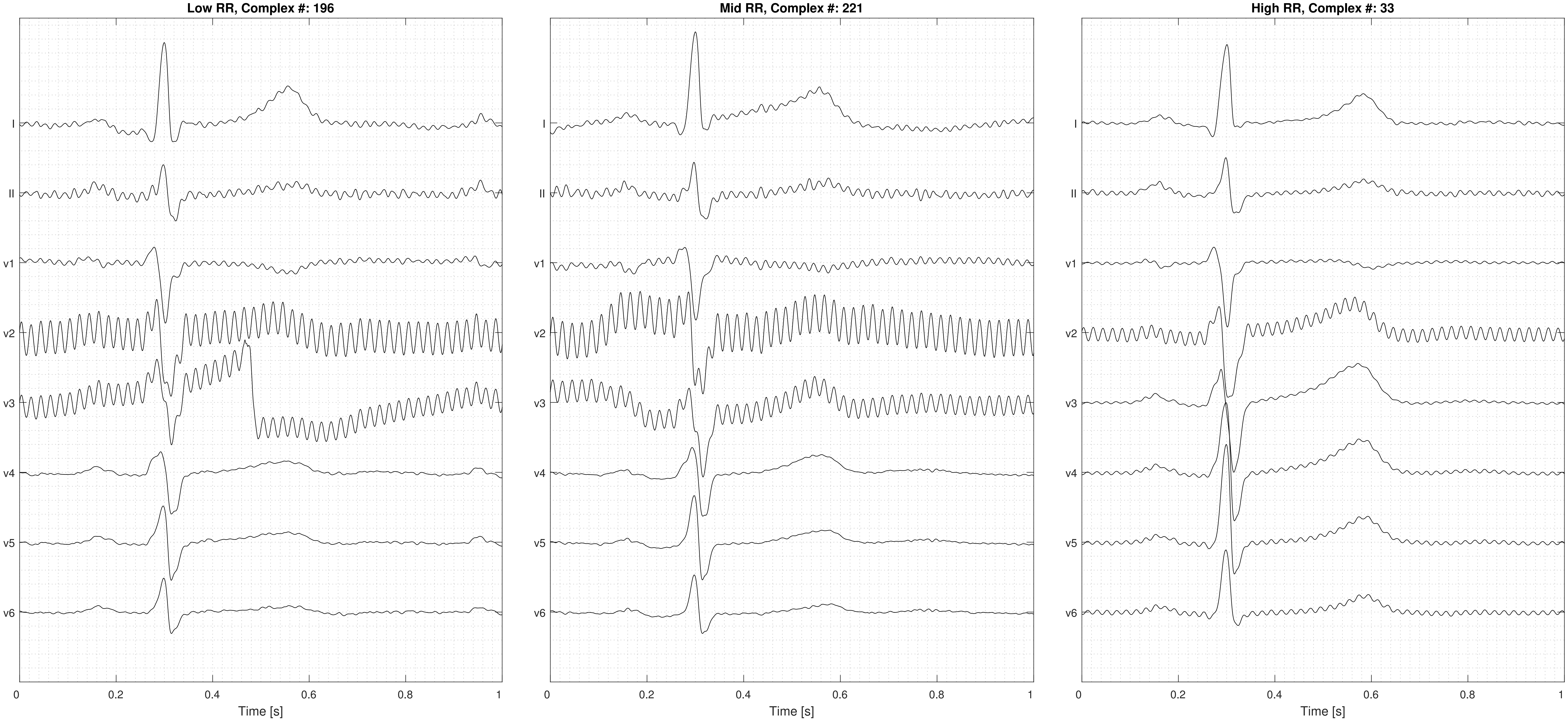

Low RR, Complex #: 196
Mid RR, Complex #: 221
High RR, Complex #: 33
I
II
v1
v2
v3
v4
v5
v6
0
0.2
0.4
0.6
0.8
1
Time [s]

Patient 98

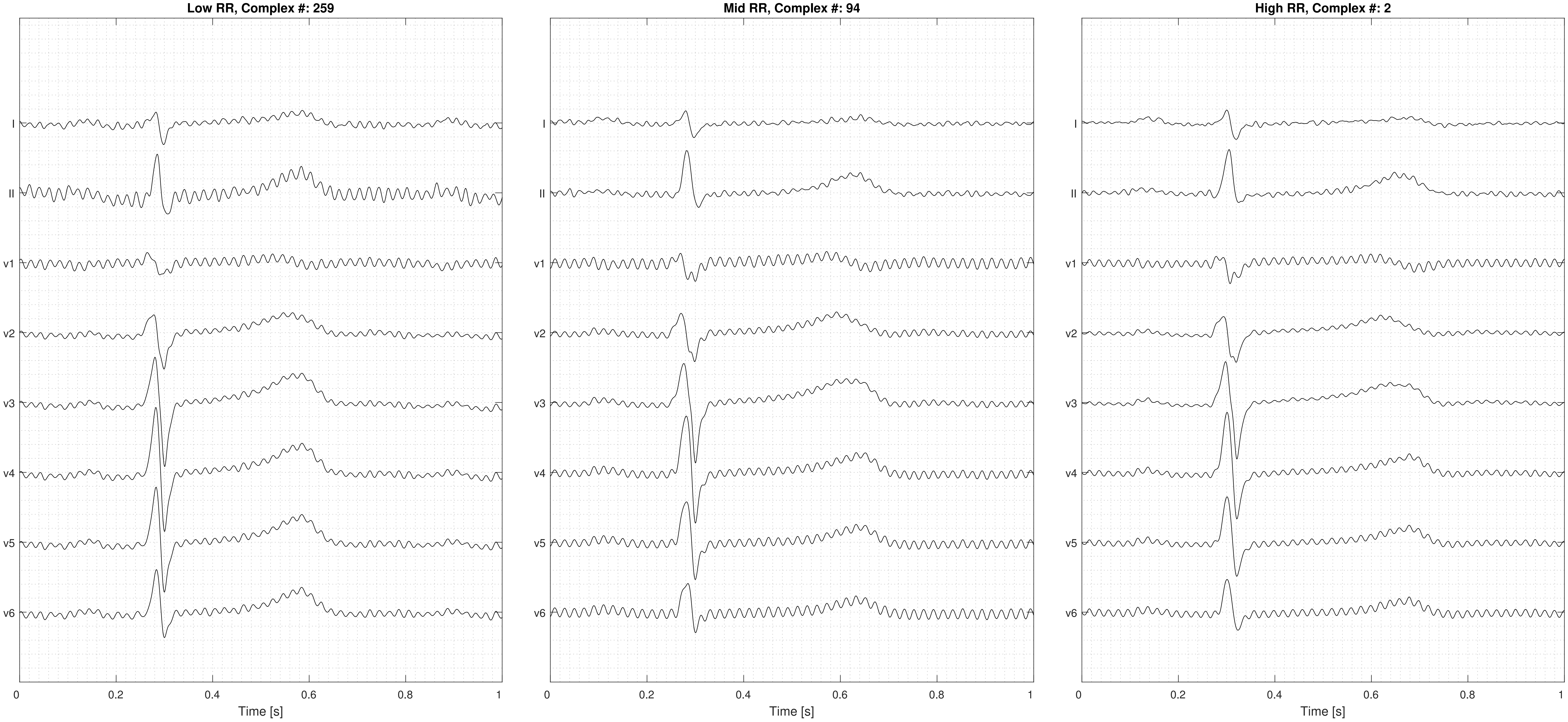

Low RR, Complex #: 259
Mid RR, Complex #: 94
High RR, Complex #: 2
I
II
v1
v2
v3
v4
v5
v6
0
0.2
0.4
0.6
0.8
1
Time [s]

Patient 99

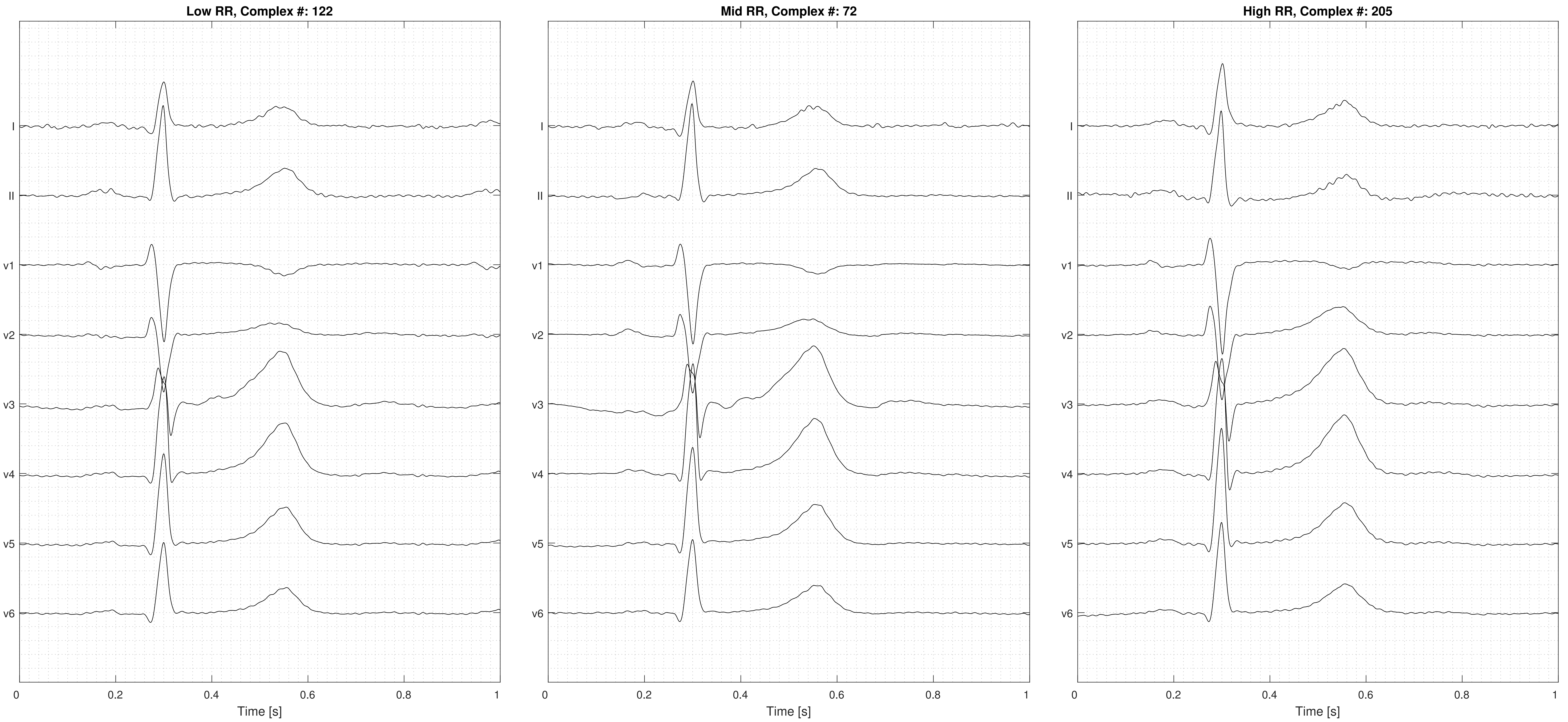

Low RR, Complex #: 122
Mid RR, Complex #: 72
High RR, Complex #: 205
I
II
v1
v2
v3
v4
v5
v6
0
0.2
0.4
0.6
0.8
1
Time [s]

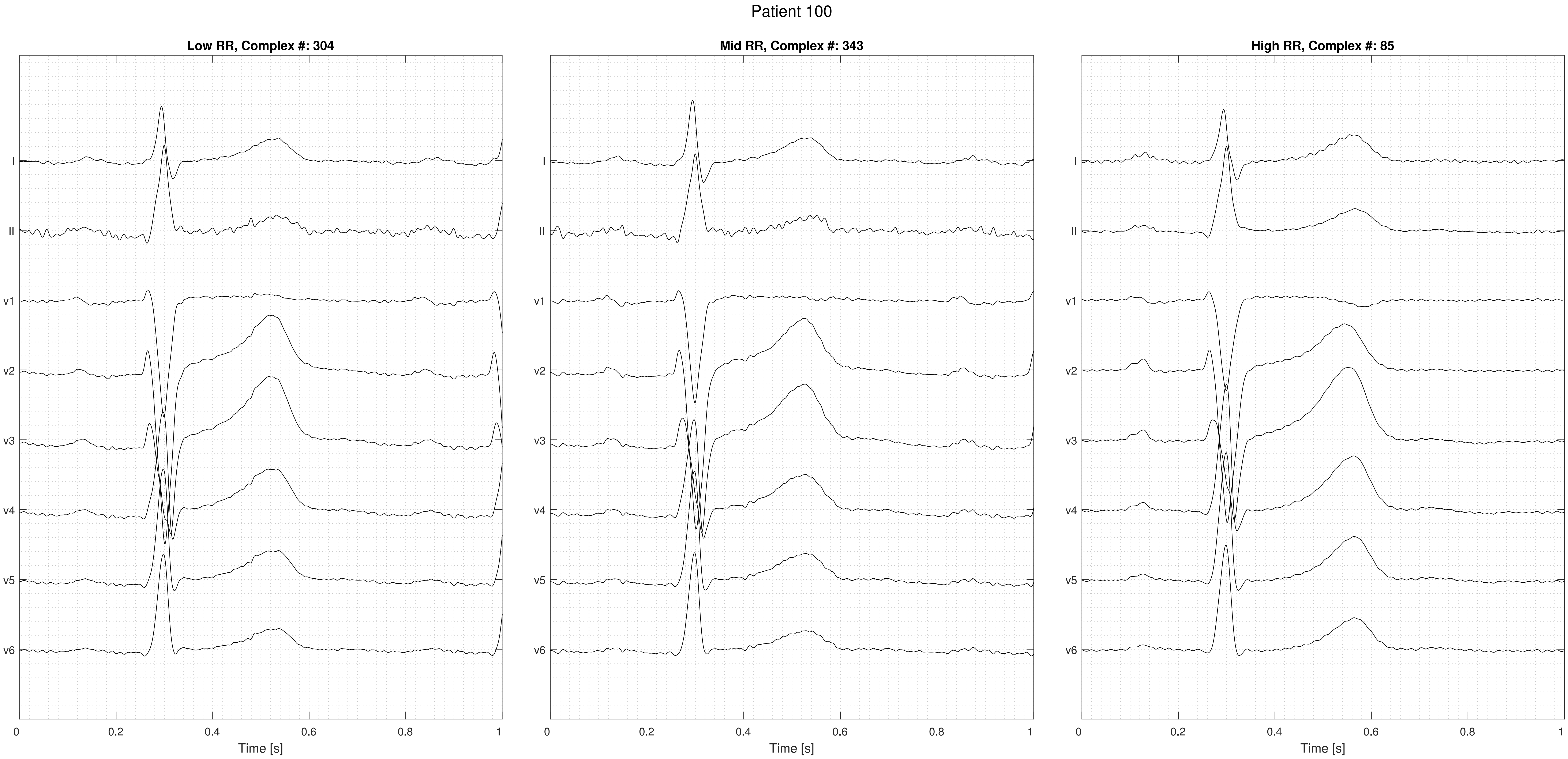
Patient 100
Low RR, Complex #: 304
Mid RR, Complex #: 343
High RR, Complex #: 85
I
II
v1
v2
v3
v4
v5
v6
0
0.2
0.4
0.6
0.8
1
Time [s]

Patient 101

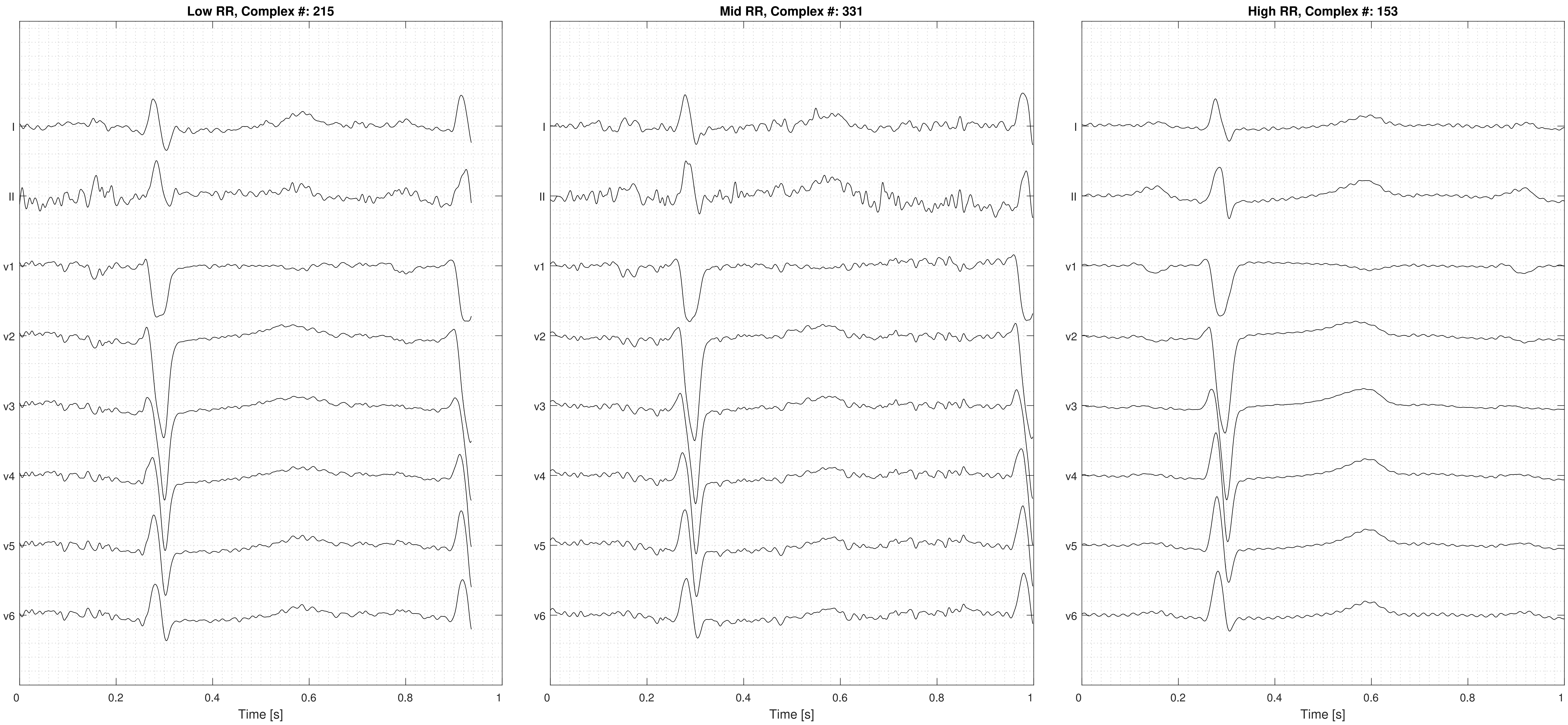

Low RR, Complex #: 215
Mid RR, Complex #: 331
High RR, Complex #: 153
I
II
v1
v2
v3
v4
v5
v6
0
0.2
0.4
0.6
0.8
1
Time [s]

Patient 102

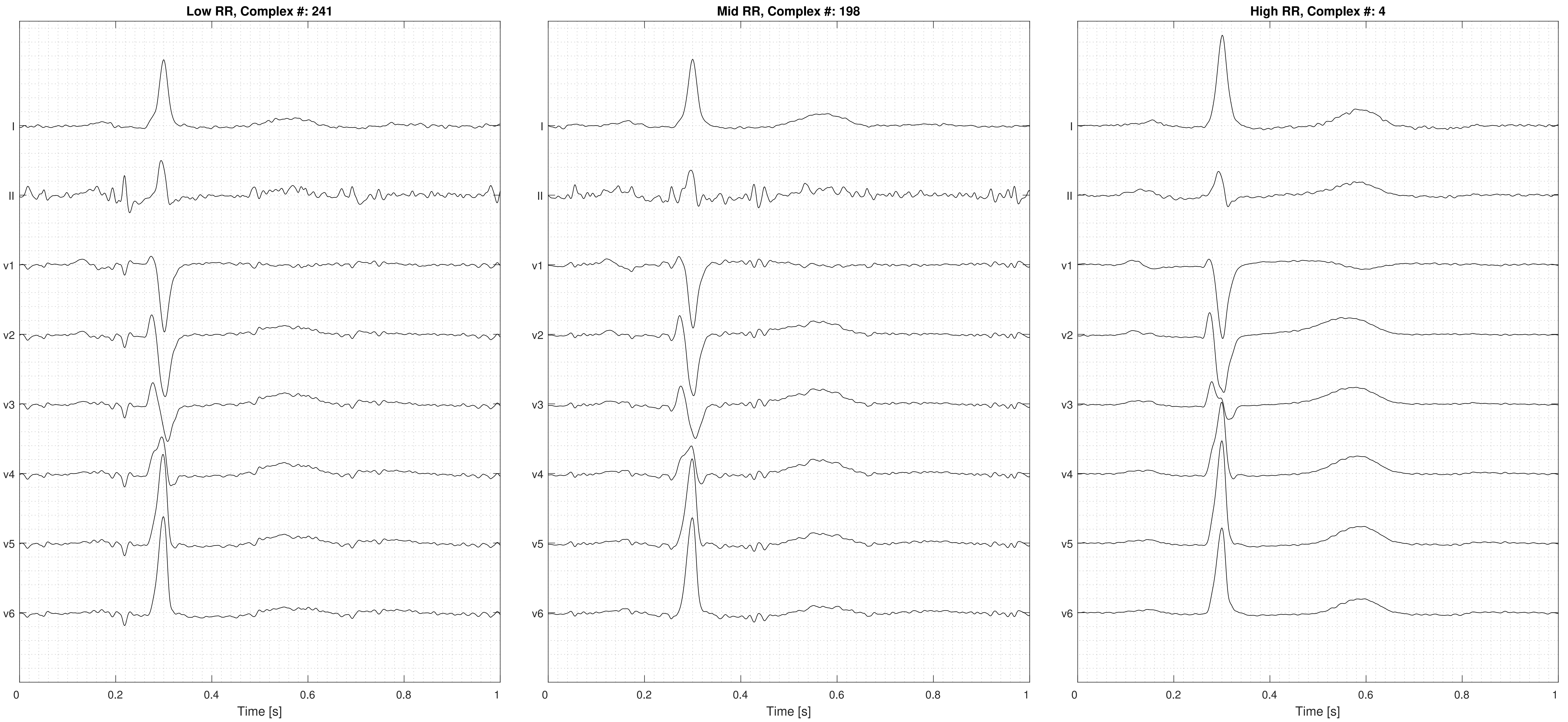

Low RR, Complex #: 241
Mid RR, Complex #: 198
High RR, Complex #: 4
I
II
v1
v2
v3
v4
v5
v6
0
0.2
0.4
0.6
0.8
1
Time [s]

Patient 103

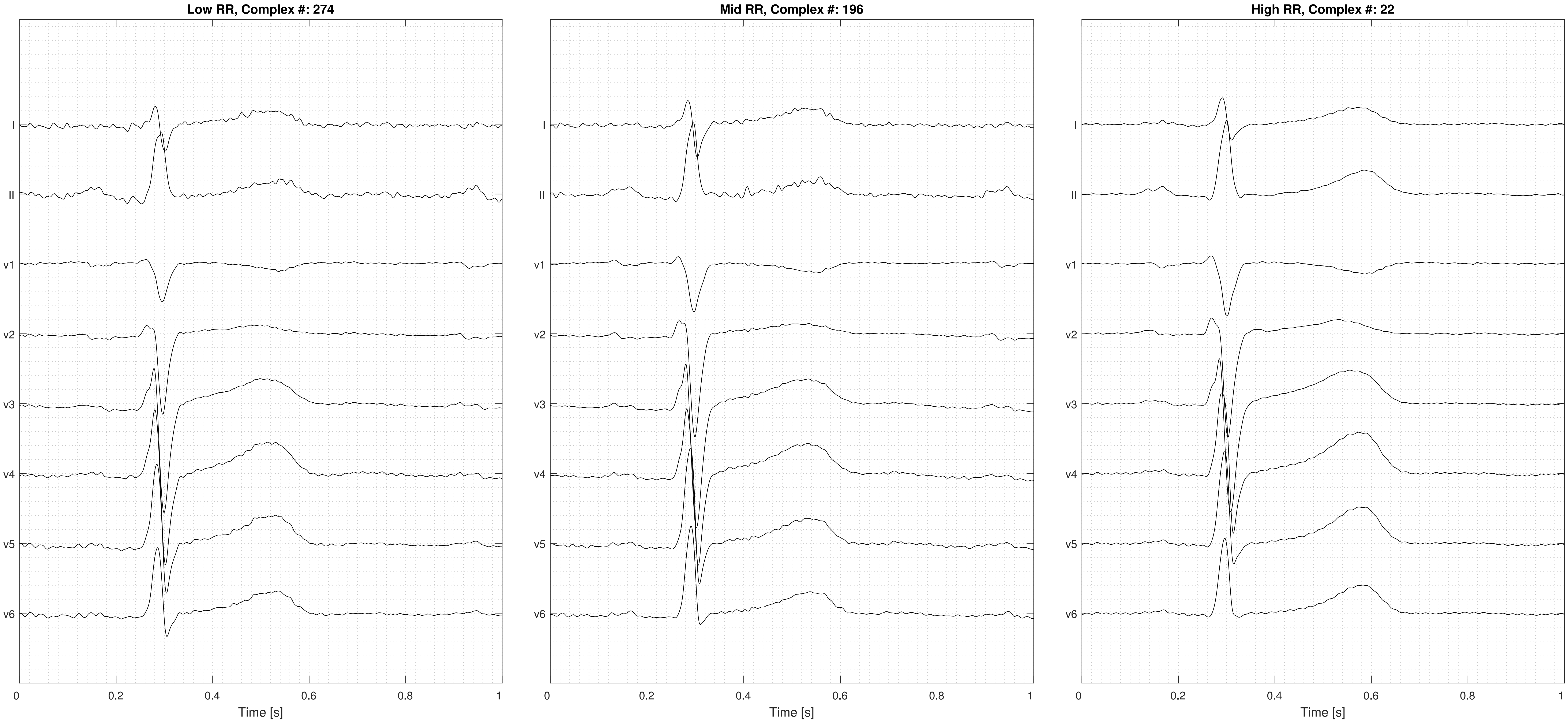
Low RR, Complex #: 274
Mid RR, Complex #: 196
High RR, Complex #: 22
I
II
v1
v2
v3
v4
v5
v6
0
0.2
0.4
0.6
0.8
1
Time [s]

Patient 104

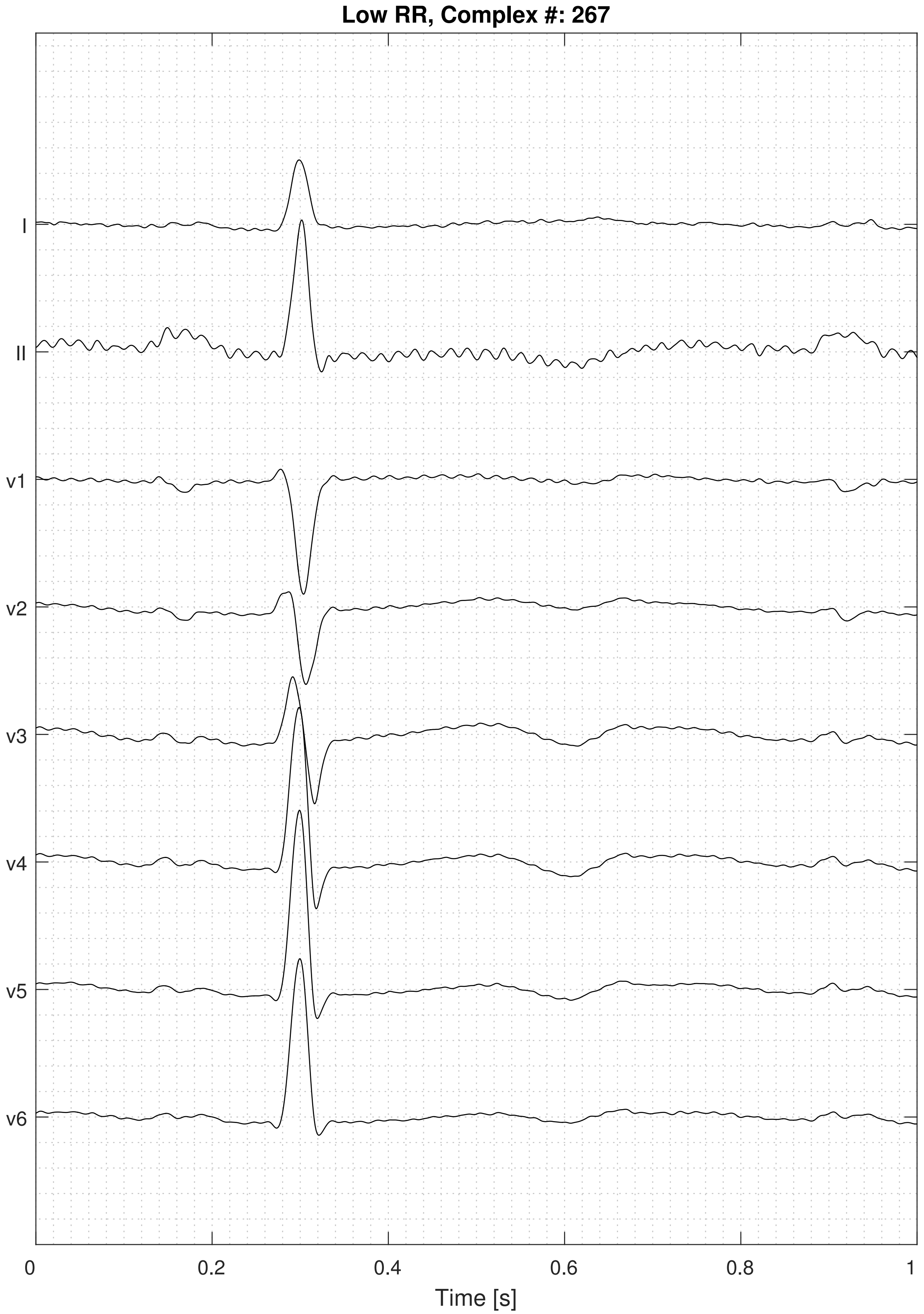

Low RR, Complex #: 267
I
II
v1
v2
v3
v4
v5
v6
0
0.2
0.4
0.6
0.8
1
Time [s]

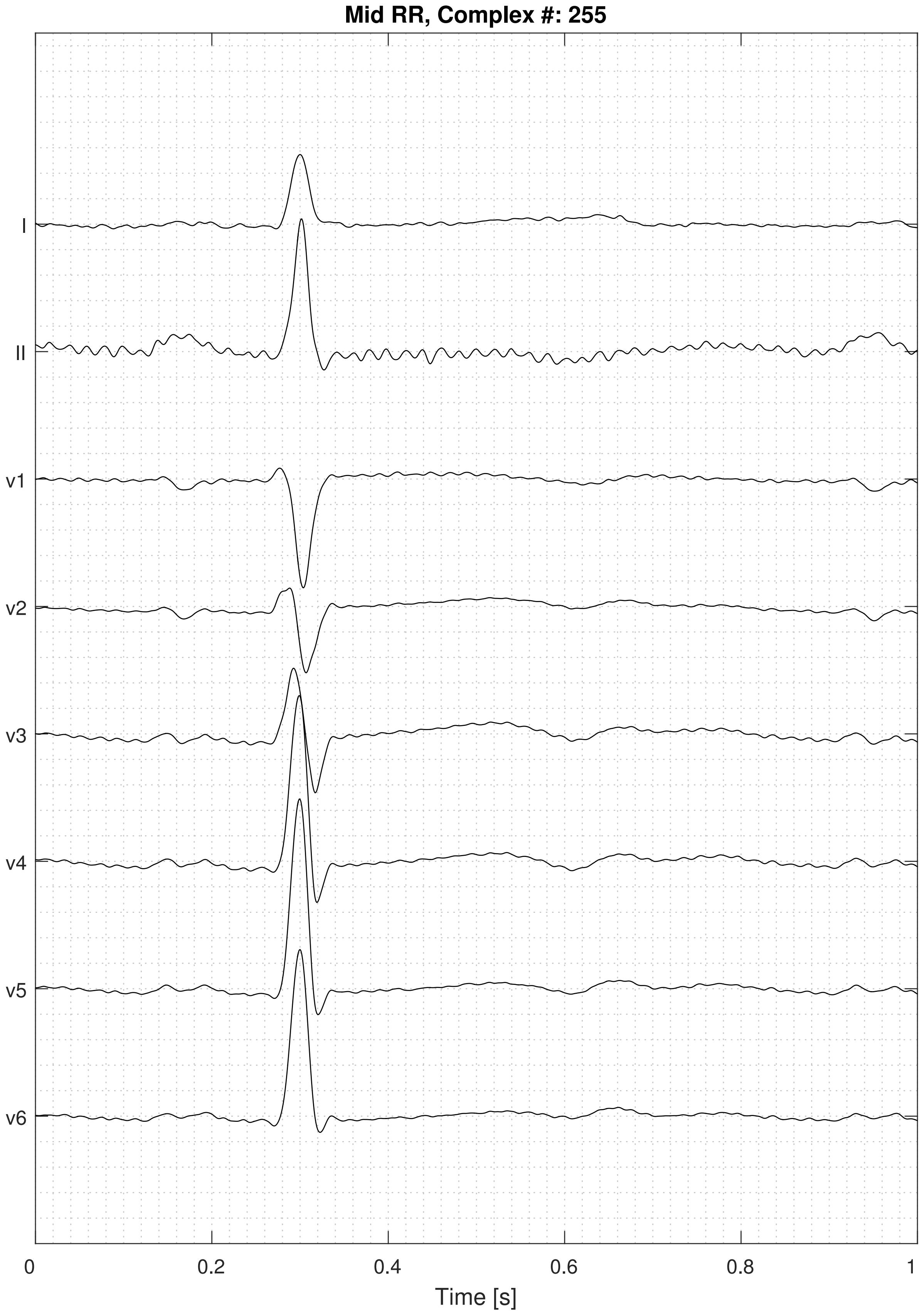

Mid RR, Complex #: 255
I
II
v1
v2
v3
v4
v5
v6
0
0.2
0.4
0.6
0.8
1
Time [s]

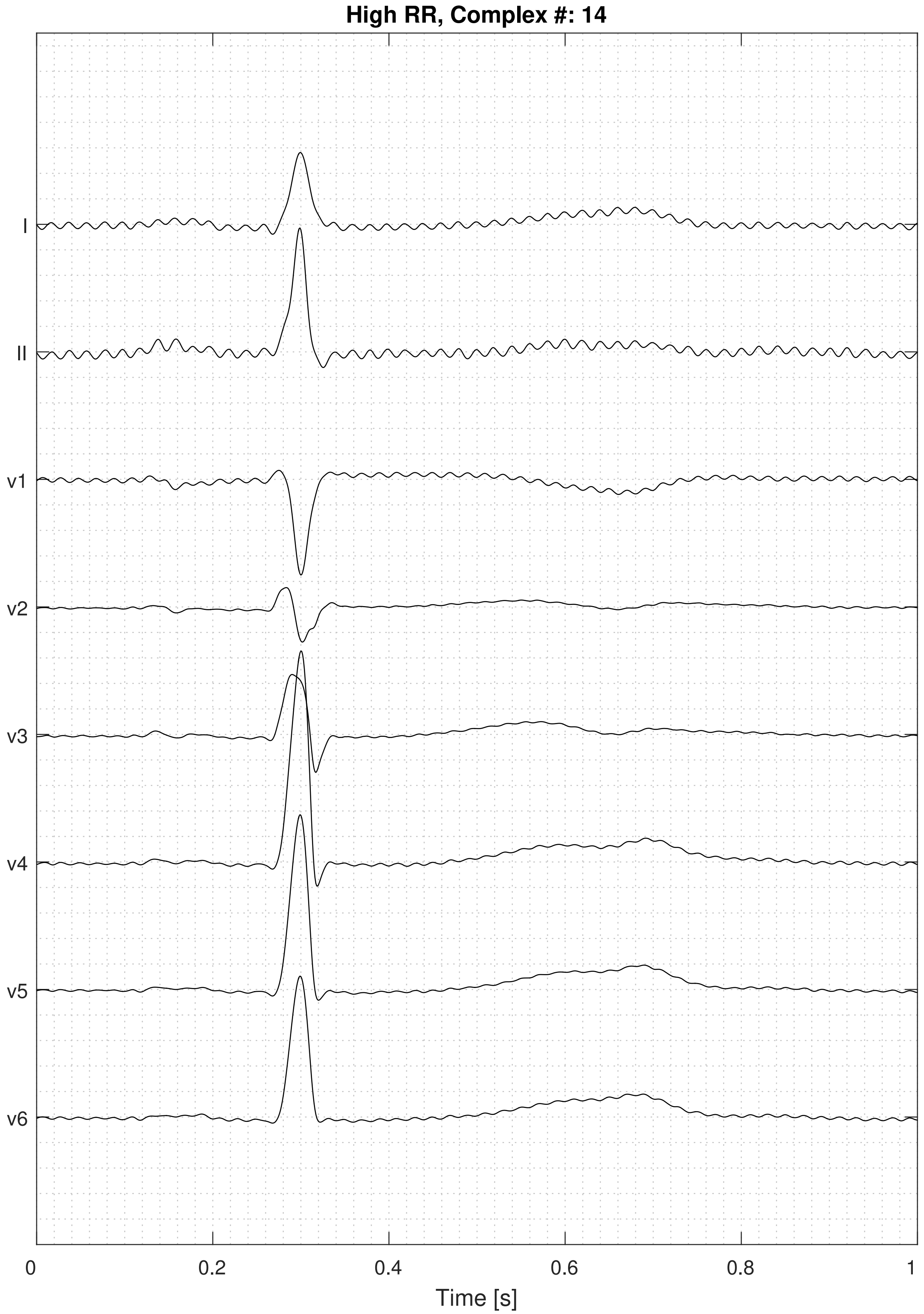

High RR, Complex #: 14
I
II
v1
v2
v3
v4
v5
v6
0
0.2
0.4
0.6
0.8
1
Time [s]

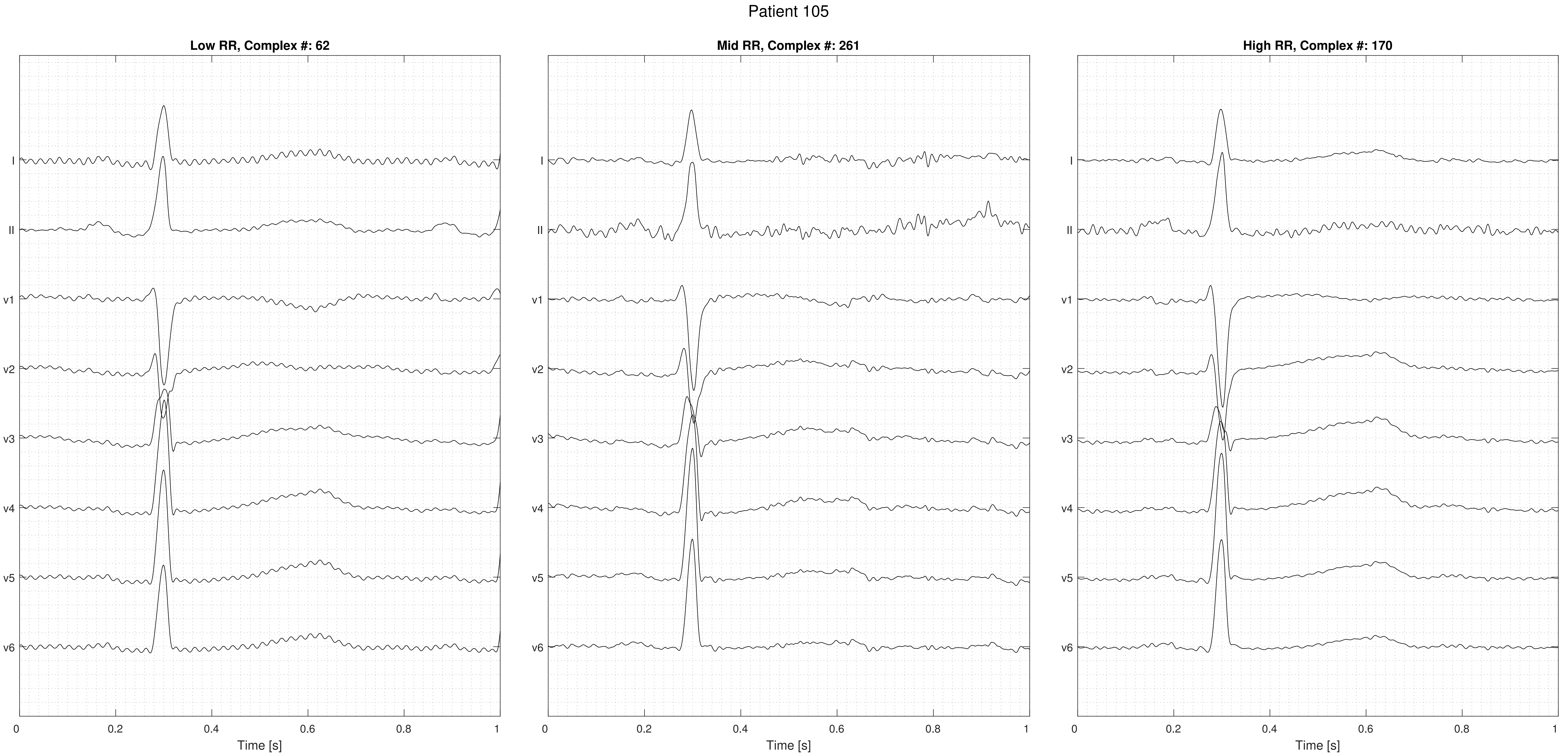
Patient 105
Low RR, Complex #: 62
Mid RR, Complex #: 261
High RR, Complex #: 170
I
II
v1
v2
v3
v4
v5
v6
0
0.2
0.4
0.6
0.8
1
Time [s]

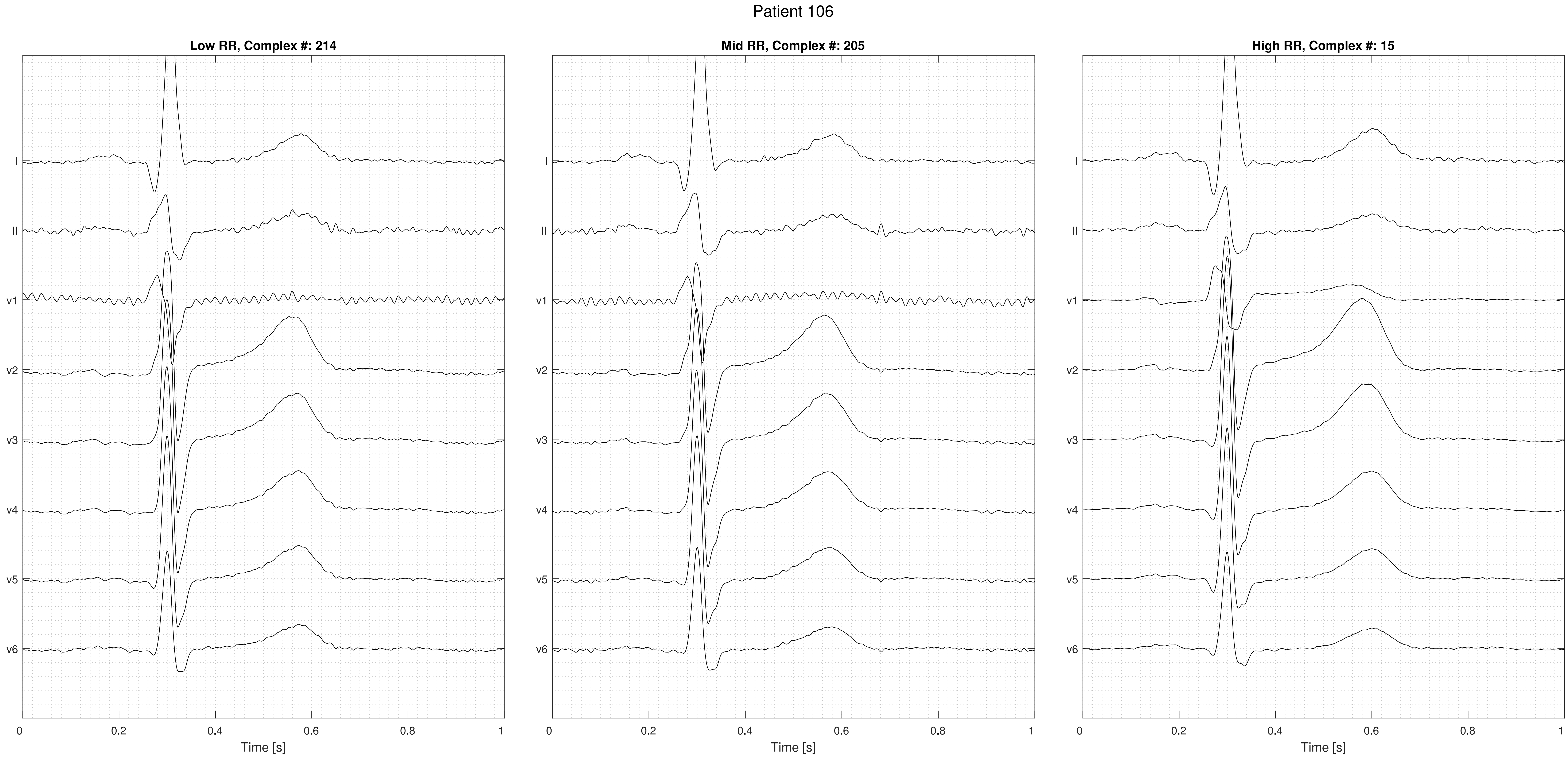

Patient 106
Low RR, Complex #: 214
Mid RR, Complex #: 205
High RR, Complex #: 15
I
II
v1
v2
v3
v4
v5
v6
0
0.2
0.4
0.6
0.8
1
Time [s]

Patient 107

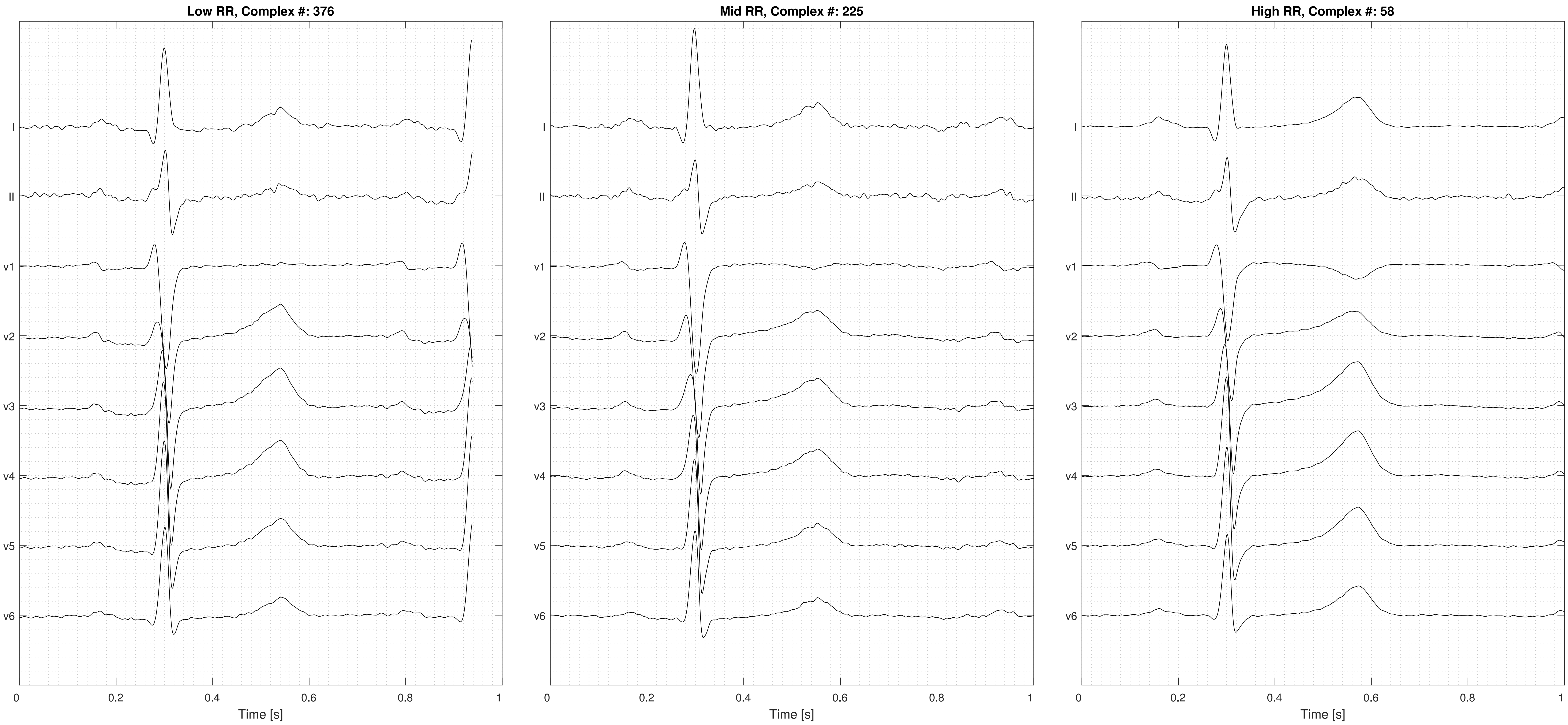

Low RR, Complex #: 376
Mid RR, Complex #: 225
High RR, Complex #: 58
I
II
v1
v2
v3
v4
v5
v6
0
0.2
0.4
0.6
0.8
1
Time [s]

Patient 108

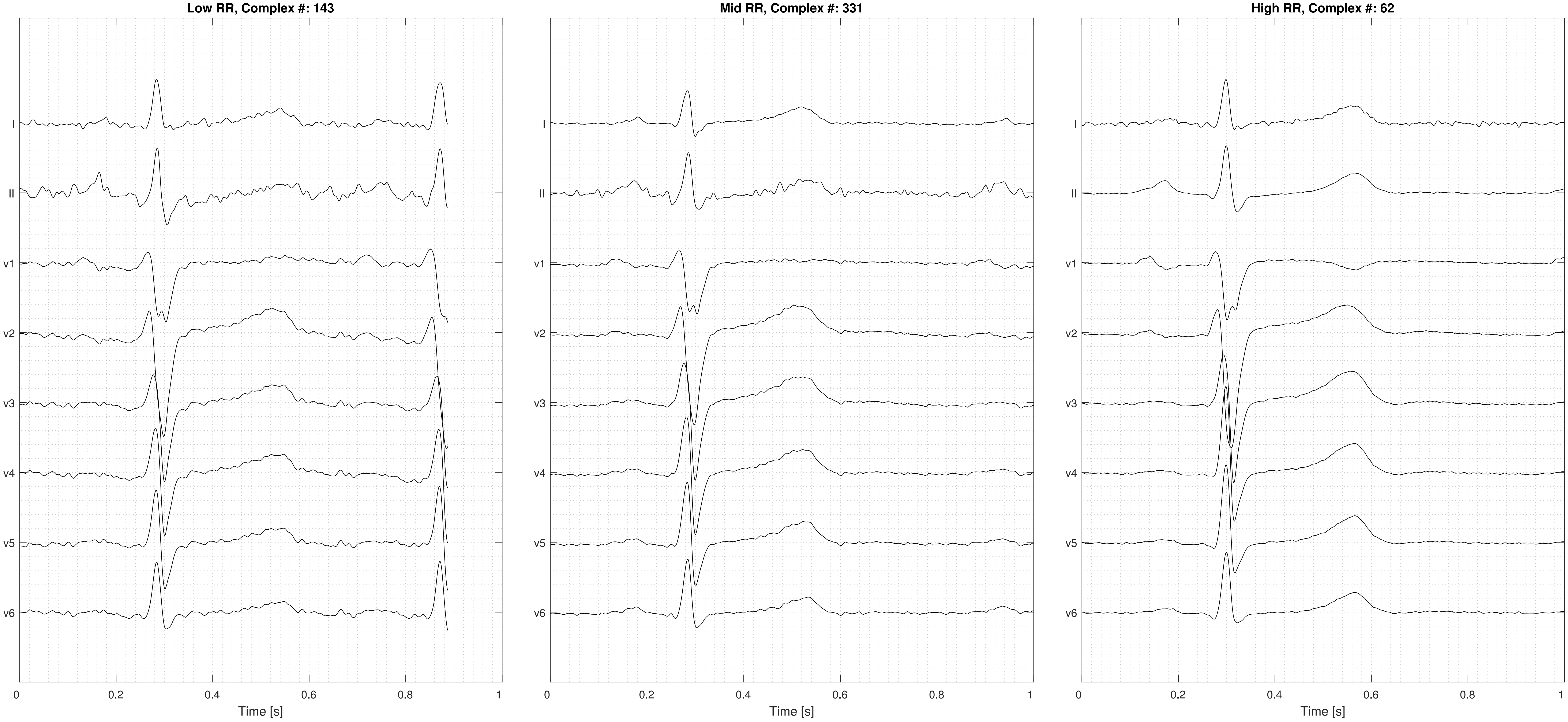

Low RR, Complex #: 143
Mid RR, Complex #: 331
High RR, Complex #: 62
I
II
v1
v2
v3
v4
v5
v6
0
0.2
0.4
0.6
0.8
1
Time [s]

Patient 109

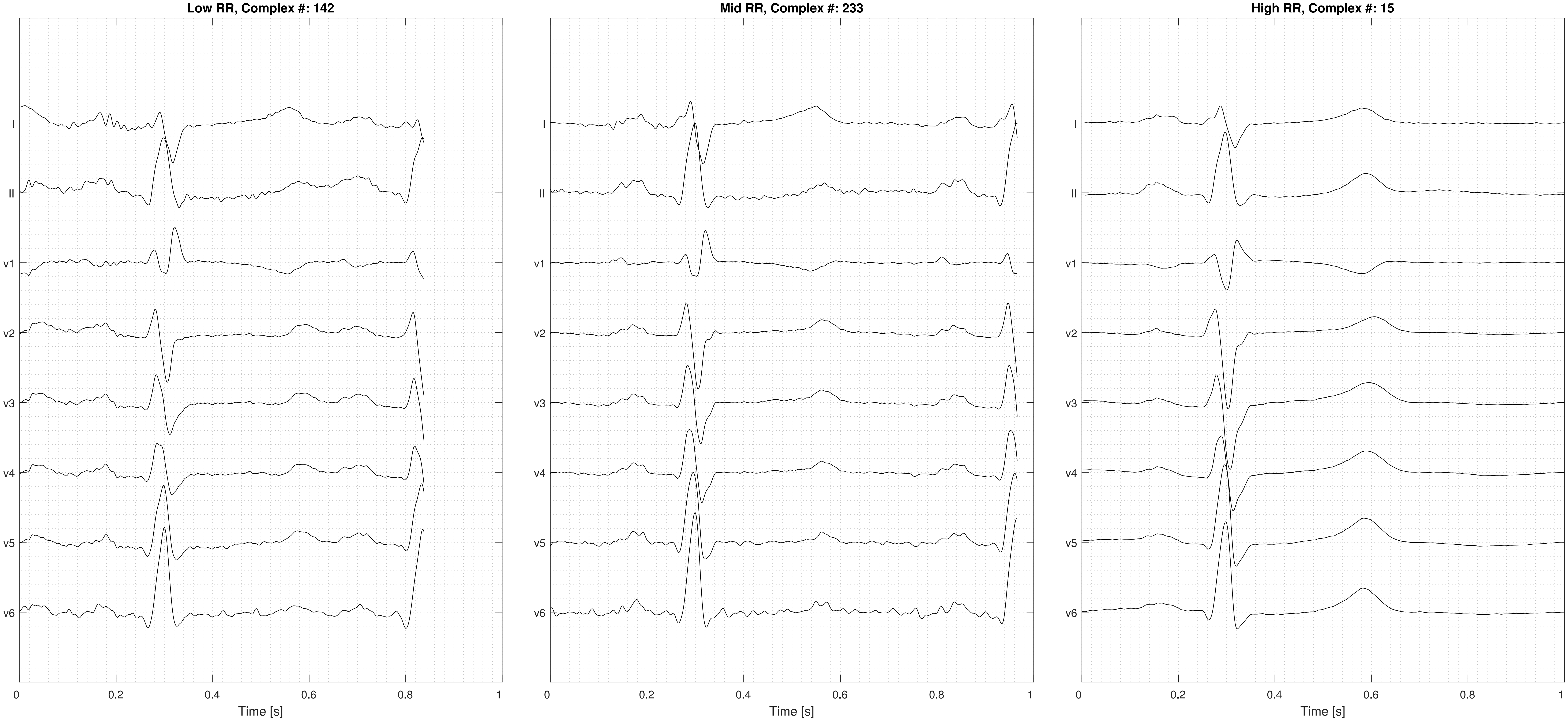

Low RR, Complex #: 142
Mid RR, Complex #: 233
High RR, Complex #: 15
I
II
v1
v2
v3
v4
v5
v6
0
0.2
0.4
0.6
0.8
1
Time [s]

Patient 110

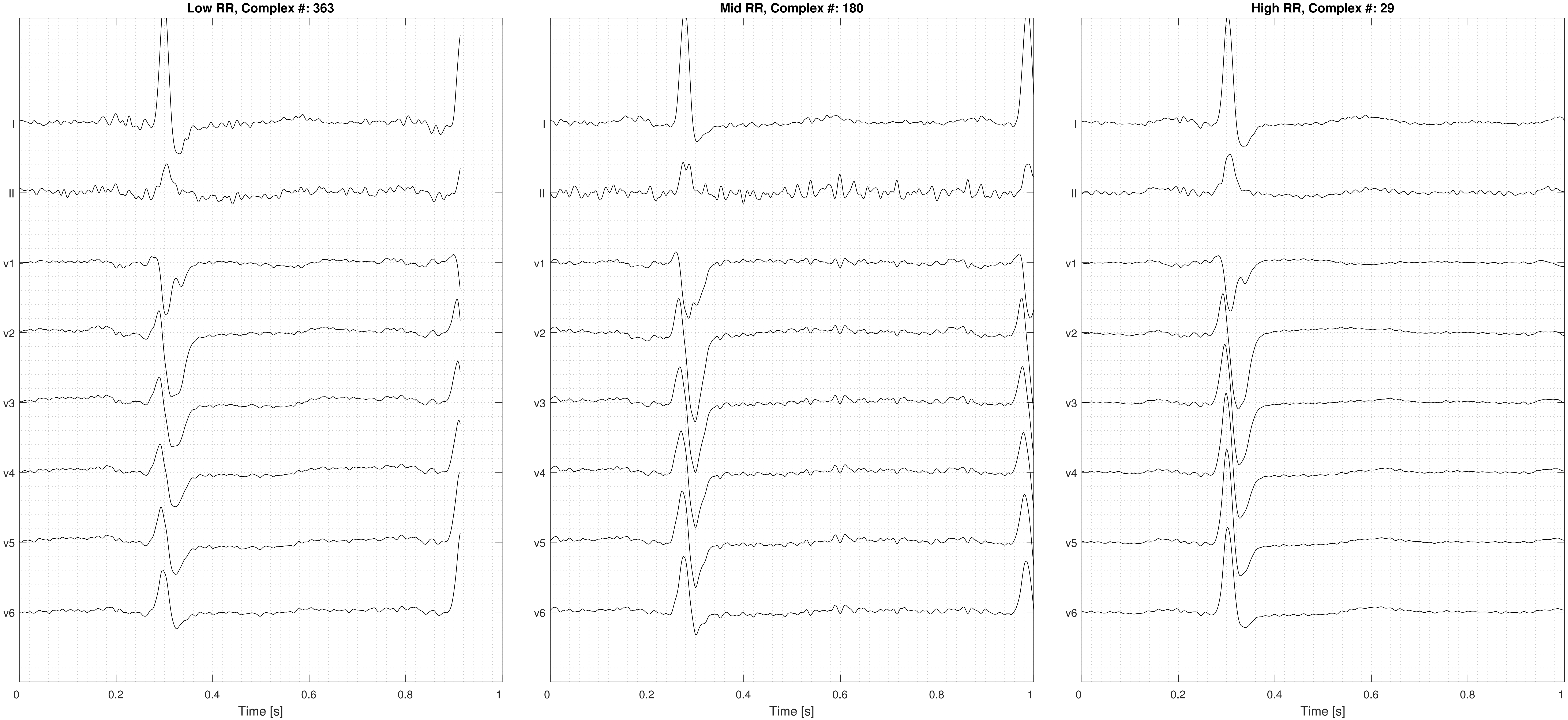

Low RR, Complex #: 363
Mid RR, Complex #: 180
High RR, Complex #: 29
I
II
v1
v2
v3
v4
v5
v6
0
0.2
0.4
0.6
0.8
1
Time [s]

Patient 111

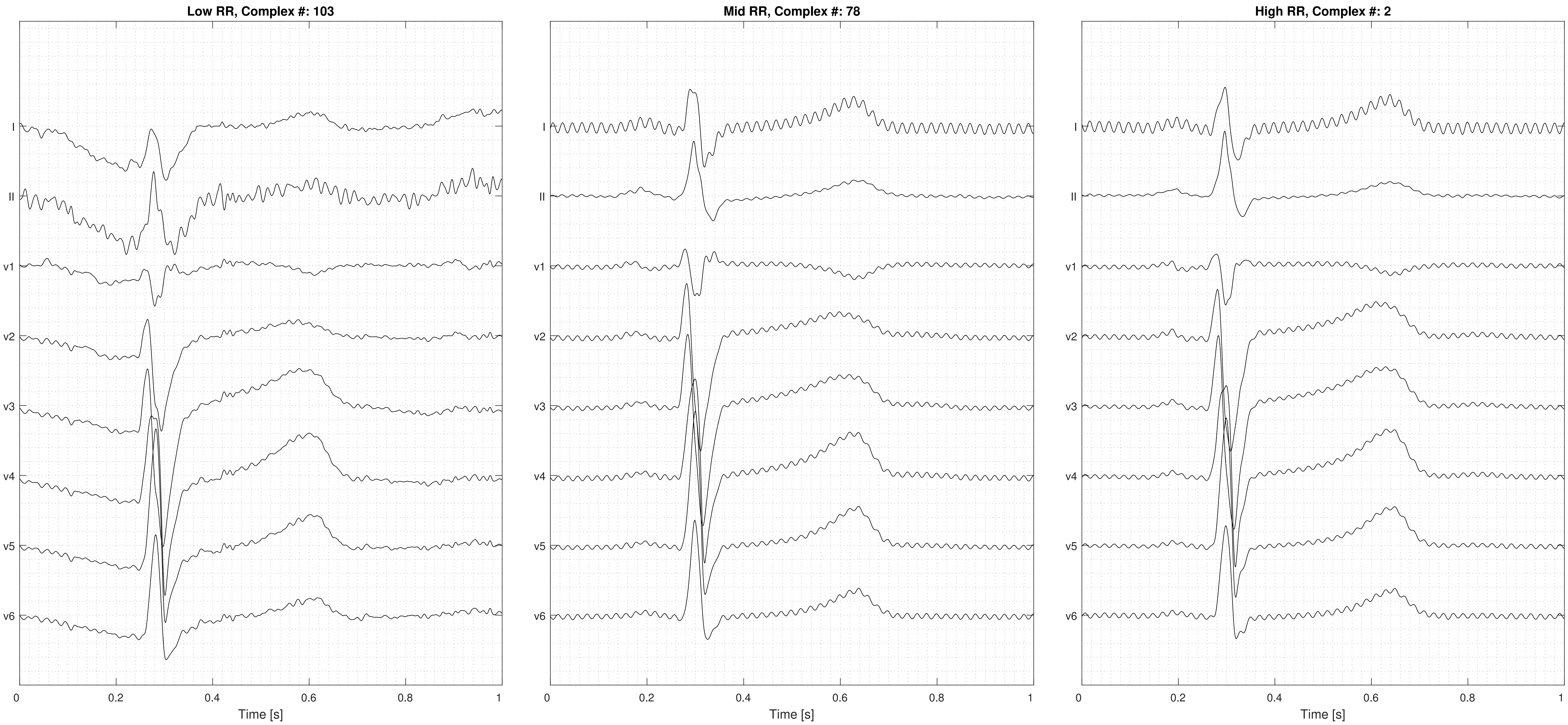

Low RR, Complex #: 103
Mid RR, Complex #: 78
High RR, Complex #: 2
I
II
v1
v2
v3
v4
v5
v6
0
0.2
0.4
0.6
0.8
1
Time [s]

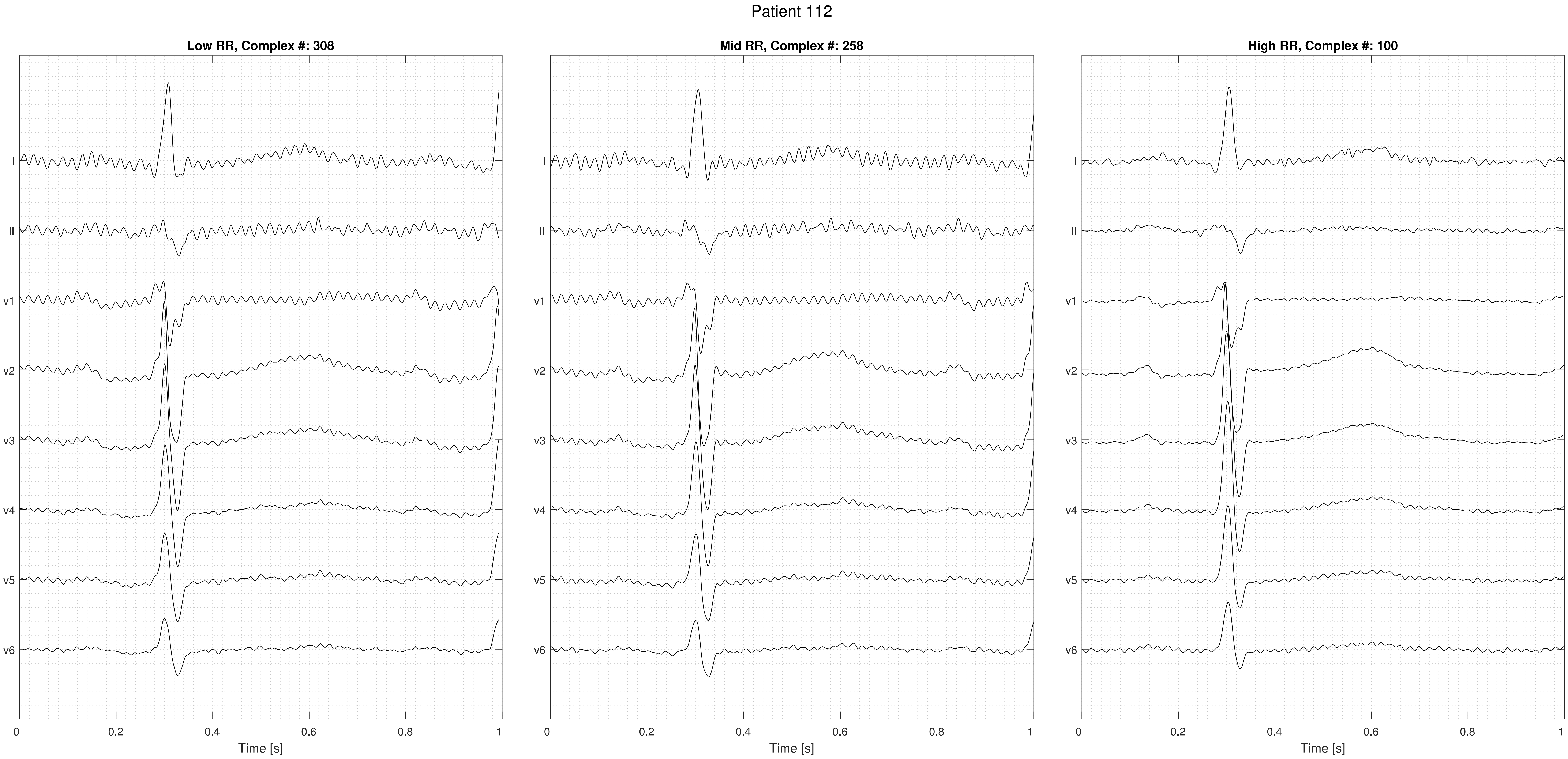

Patient 112
Low RR, Complex #: 308
Mid RR, Complex #: 258
High RR, Complex #: 100
I
II
v1
v2
v3
v4
v5
v6
0
0.2
0.4
0.6
0.8
1
Time [s]

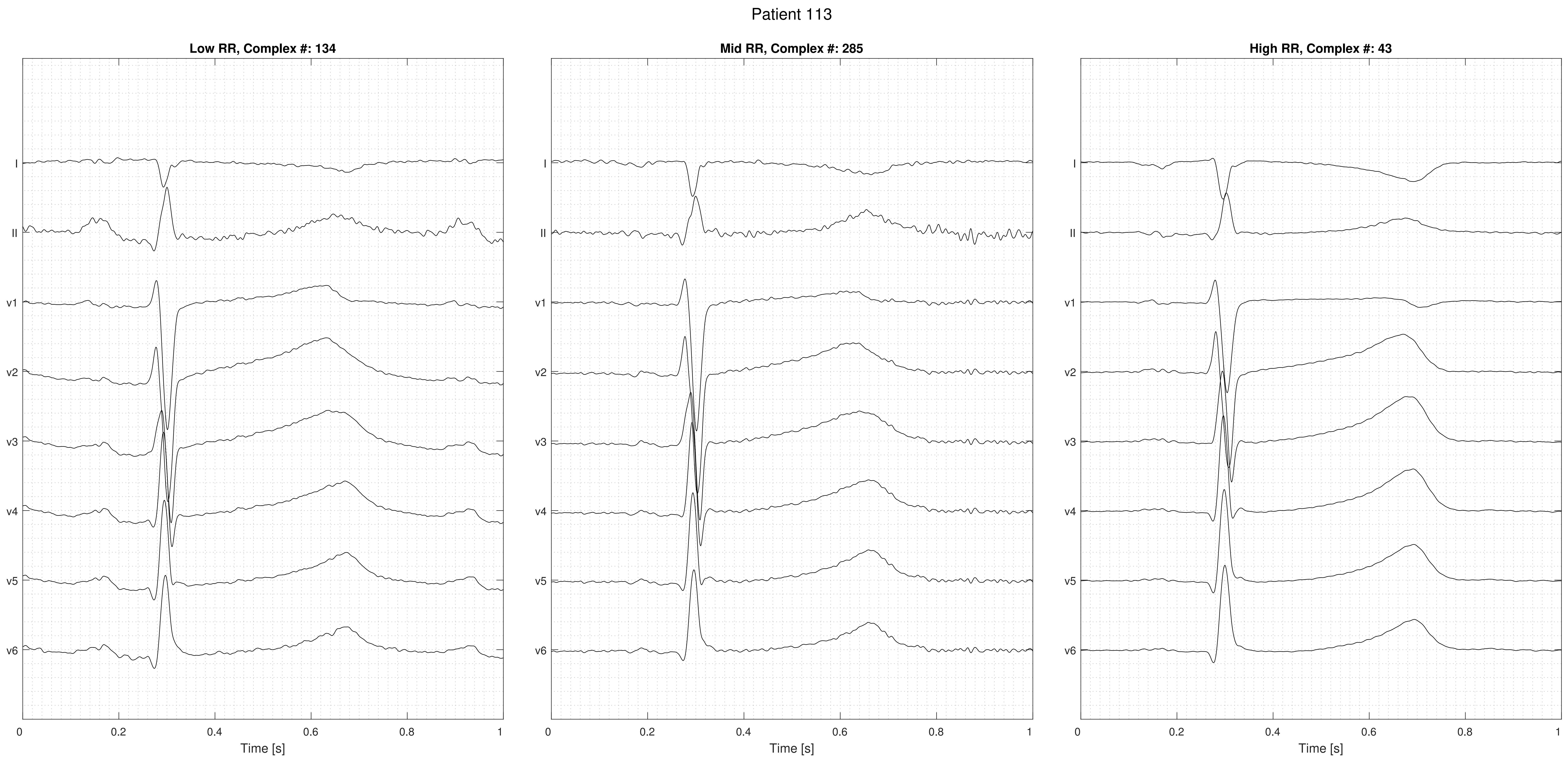
Patient 113
Low RR, Complex #: 134
Mid RR, Complex #: 285
High RR, Complex #: 43
I
II
v1
v2
v3
v4
v5
v6
0
0.2
0.4
0.6
0.8
1
Time [s]

Patient 114

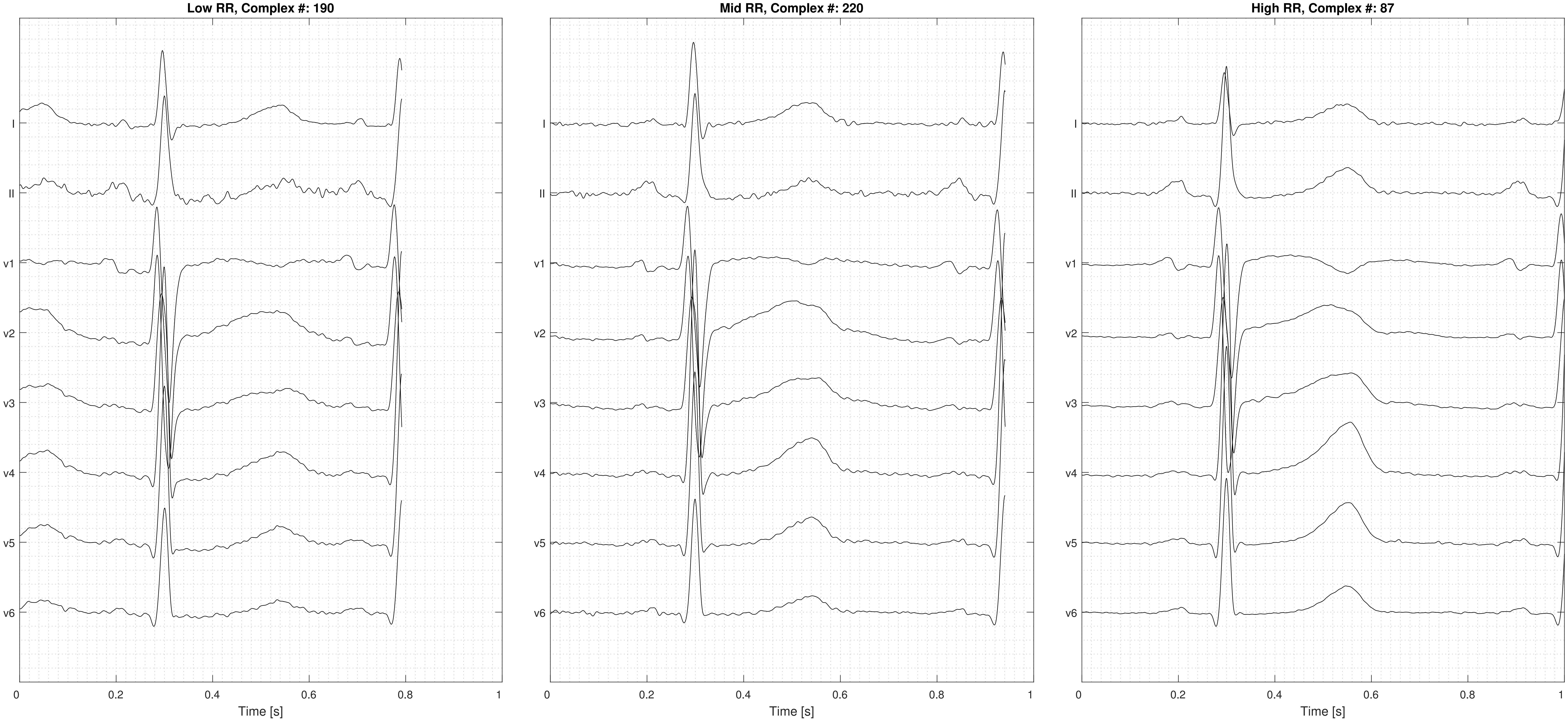

Low RR, Complex #: 190
Mid RR, Complex #: 220
High RR, Complex #: 87
I
II
v1
v2
v3
v4
v5
v6
0
0.2
0.4
0.6
0.8
1
Time [s]

Patient 115

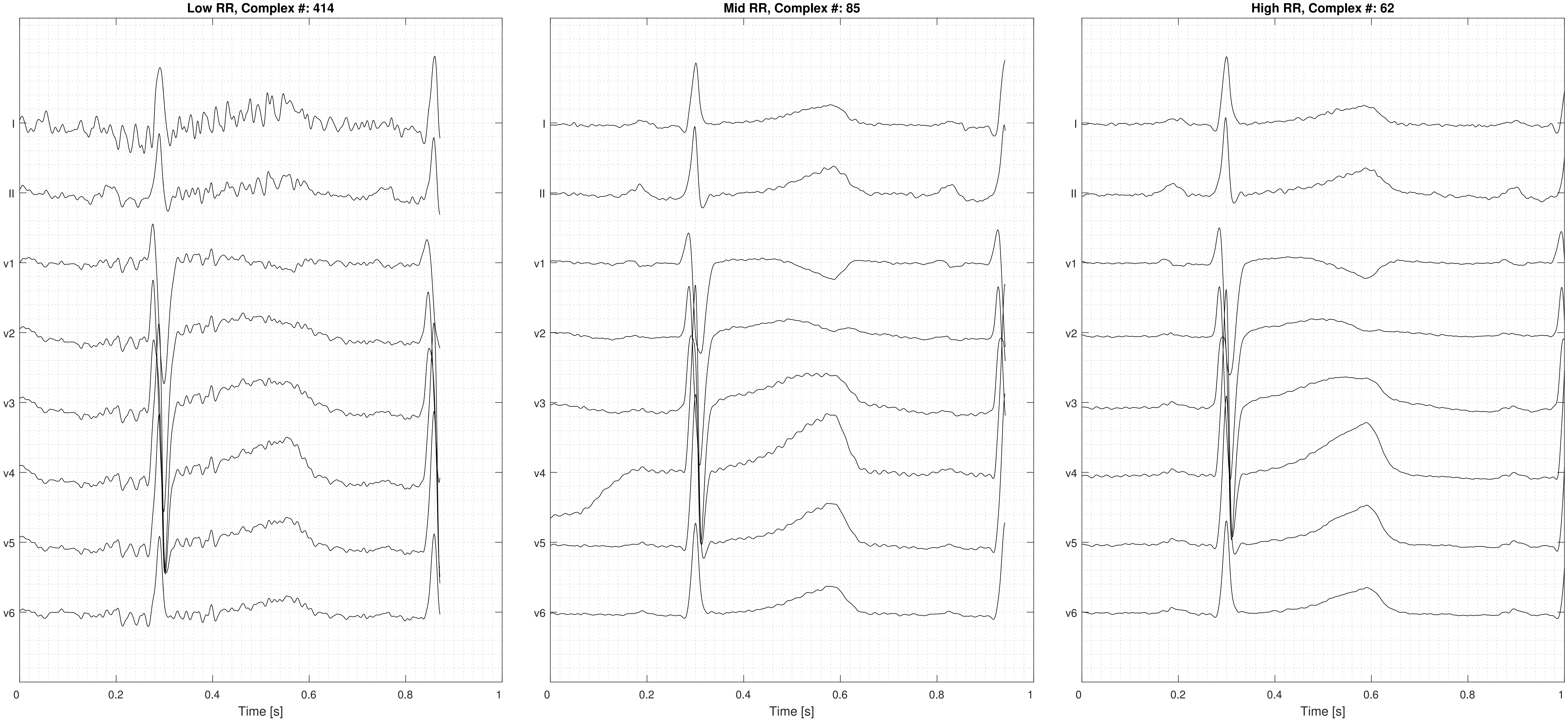

Low RR, Complex #: 414
Mid RR, Complex #: 85
High RR, Complex #: 62
I
II
v1
v2
v3
v4
v5
v6
0
0.2
0.4
0.6
0.8
1
Time [s]

Patient 116

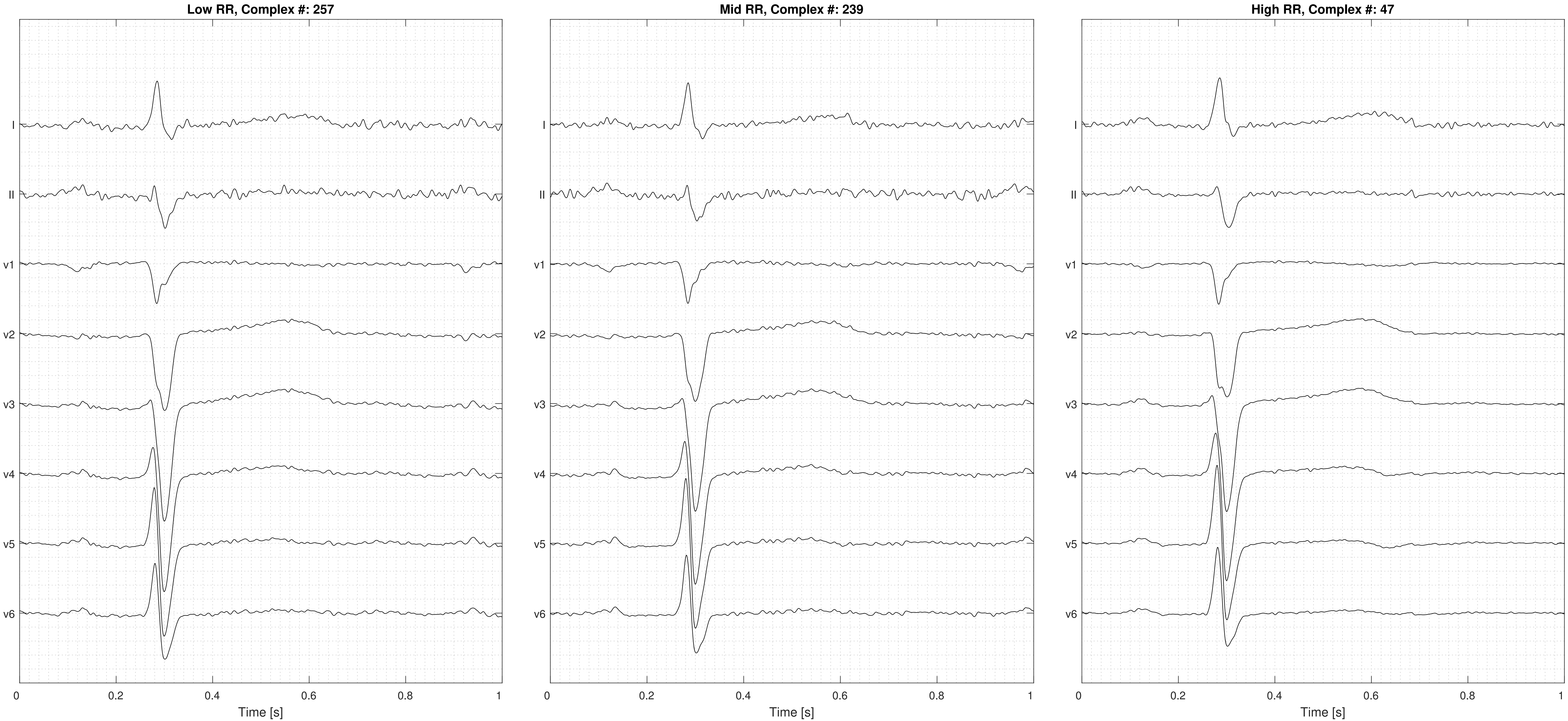

Low RR, Complex #: 257
Mid RR, Complex #: 239
High RR, Complex #: 47
I
II
v1
v2
v3
v4
v5
v6
0
0.2
0.4
0.6
0.8
1
Time [s]

Patient 117

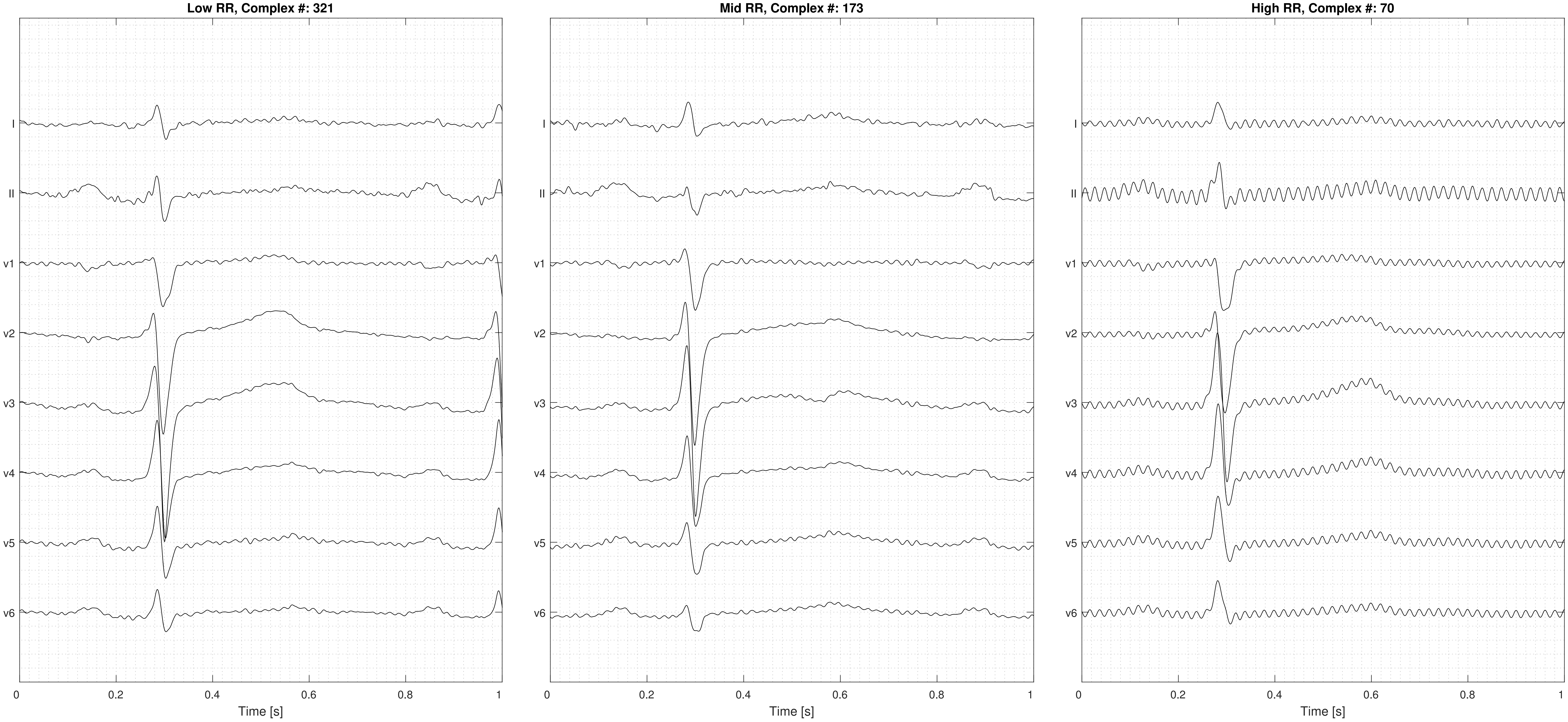

Low RR, Complex #: 321
Mid RR, Complex #: 173
High RR, Complex #: 70
I
II
v1
v2
v3
v4
v5
v6
0
0.2
0.4
0.6
0.8
1
Time [s]

Patient 118

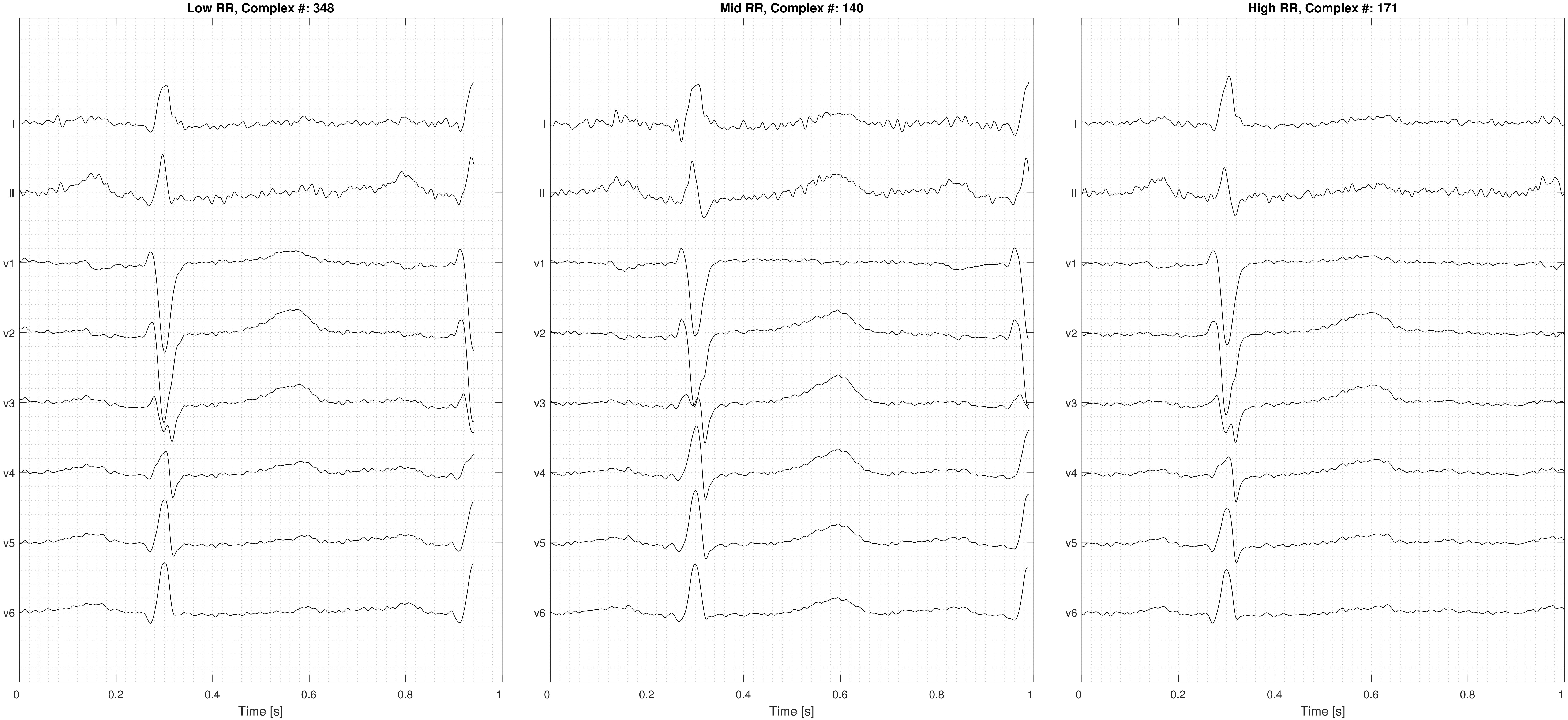

Low RR, Complex #: 348
Mid RR, Complex #: 140
High RR, Complex #: 171
I
II
v1
v2
v3
v4
v5
v6
0
0.2
0.4
0.6
0.8
1
Time [s]

Patient 119

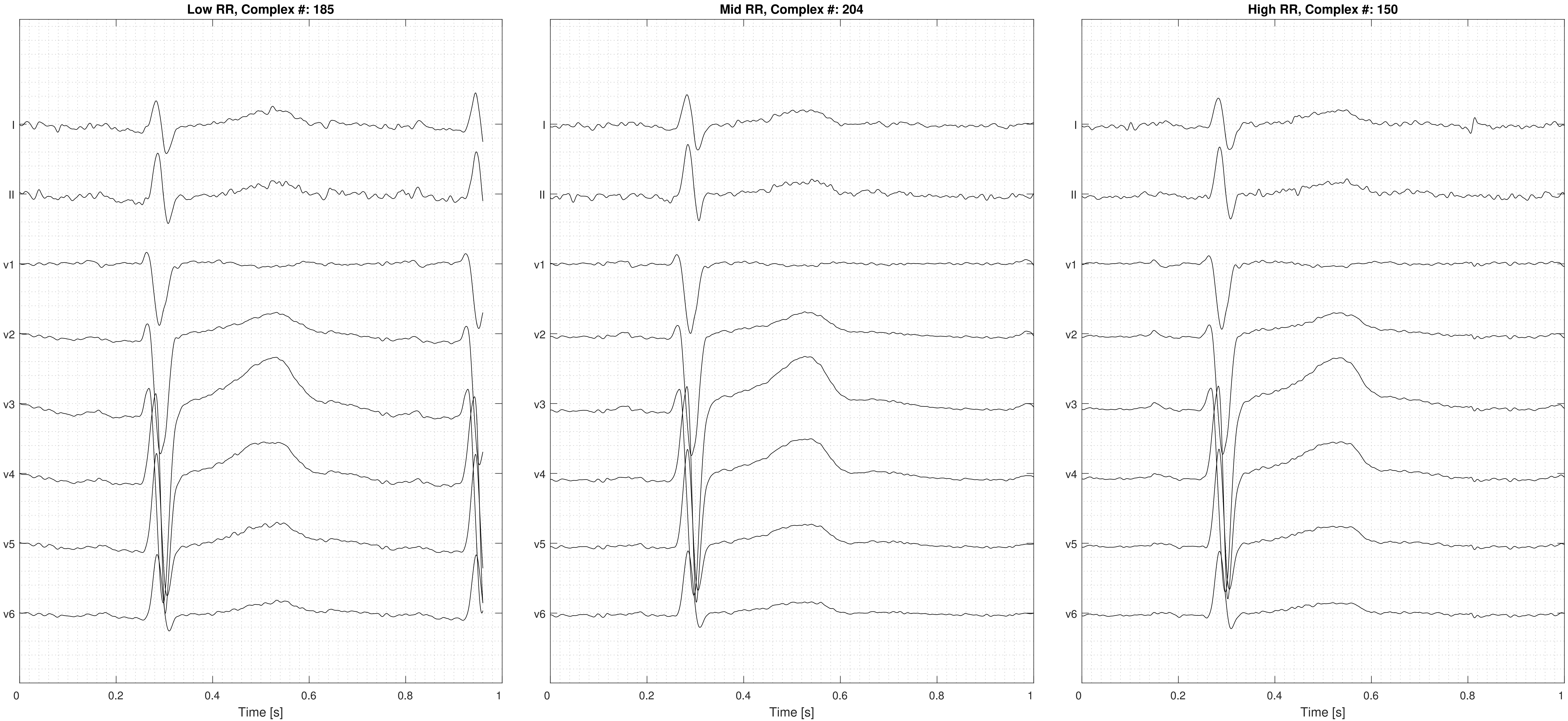

Low RR, Complex #: 185
Mid RR, Complex #: 204
High RR, Complex #: 150
I
II
v1
v2
v3
v4
v5
v6
0
0.2
0.4
0.6
0.8
1
Time [s]

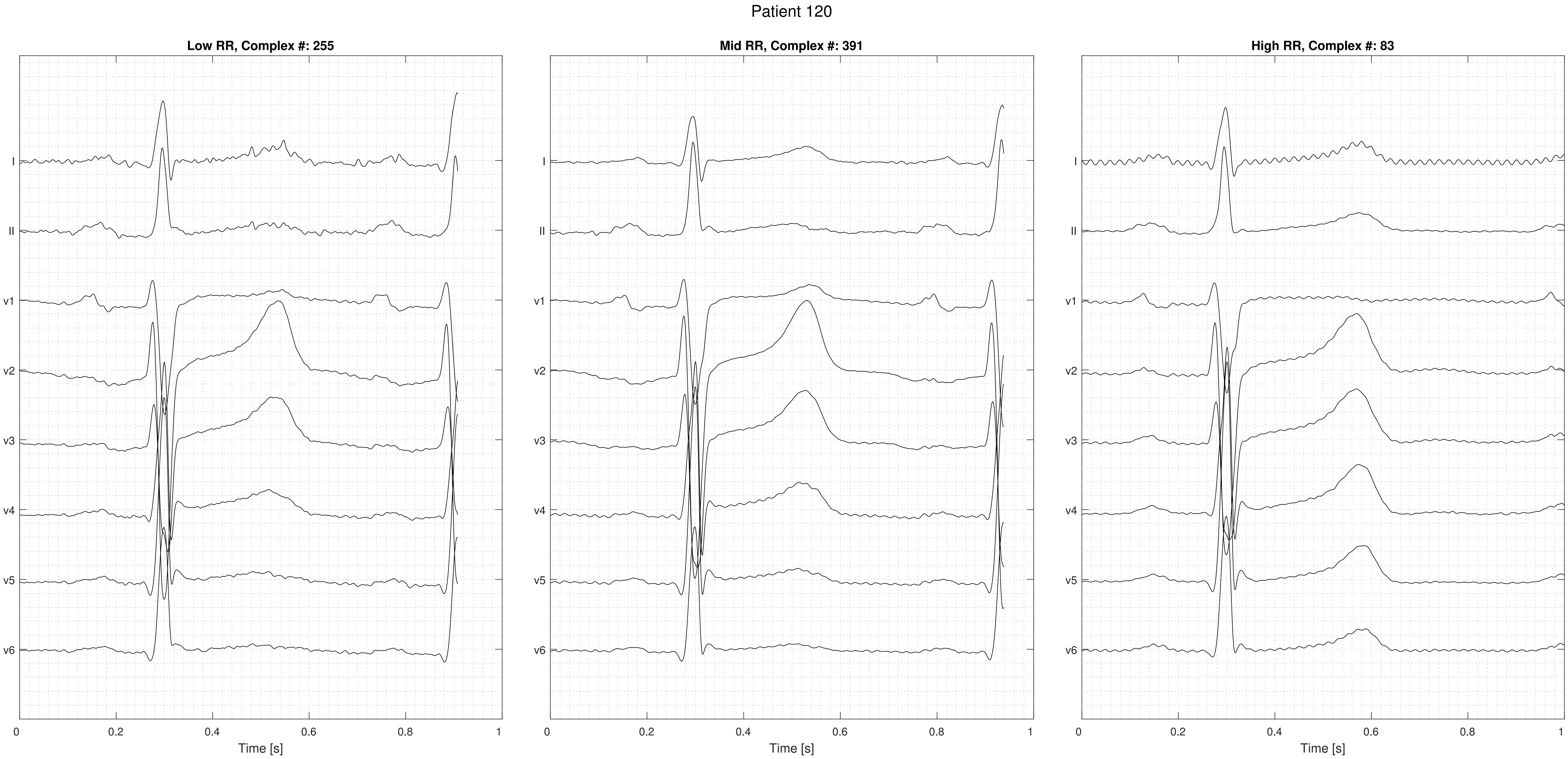
Patient 120
Low RR, Complex #: 255
Mid RR, Complex #: 391
High RR, Complex #: 83
I
II
v1
v2
v3
v4
v5
v6
0
0.2
0.4
0.6
0.8
1
Time [s]

Patient 121

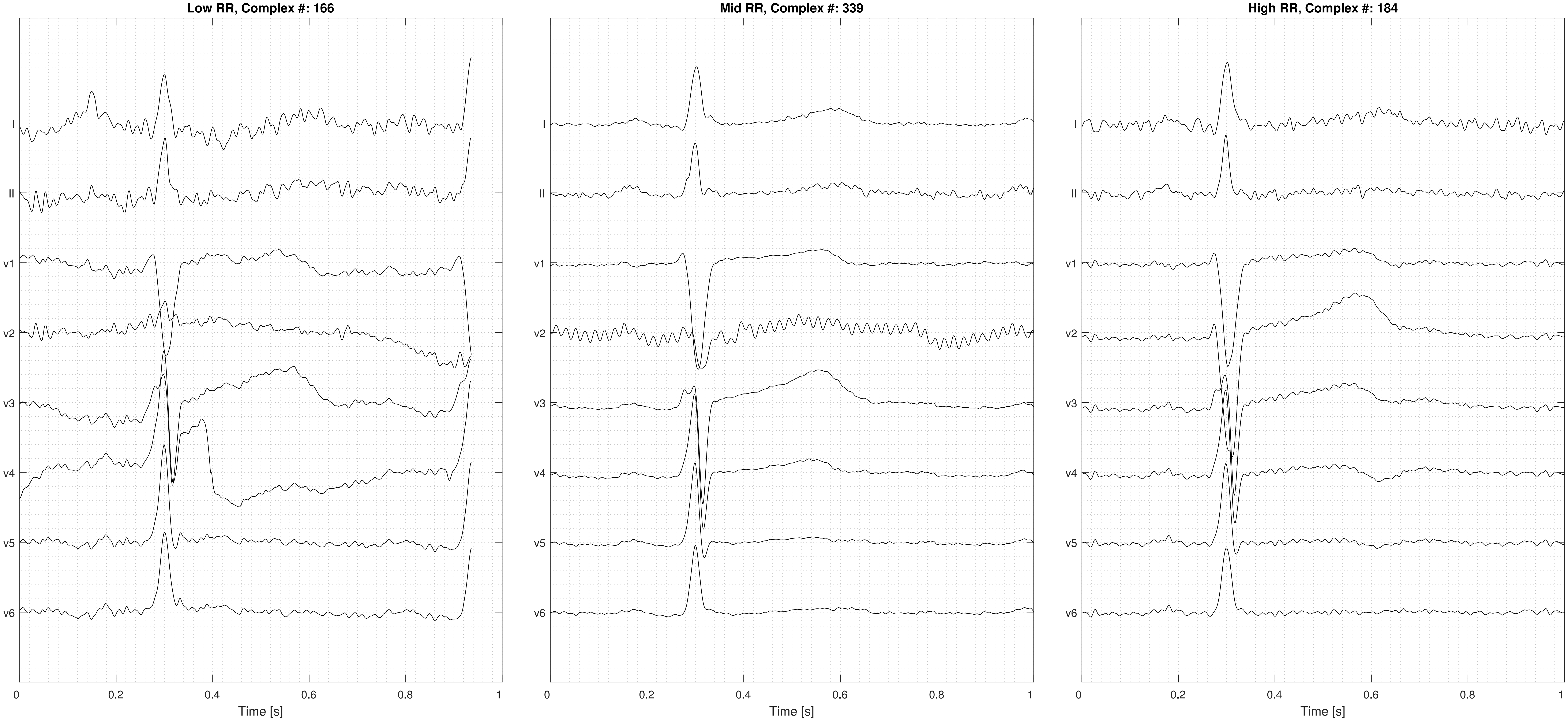

Low RR, Complex #: 166
Mid RR, Complex #: 339
High RR, Complex #: 184
I
II
v1
v2
v3
v4
v5
v6
0
0.2
0.4
0.6
0.8
1
Time [s]

Patient 122

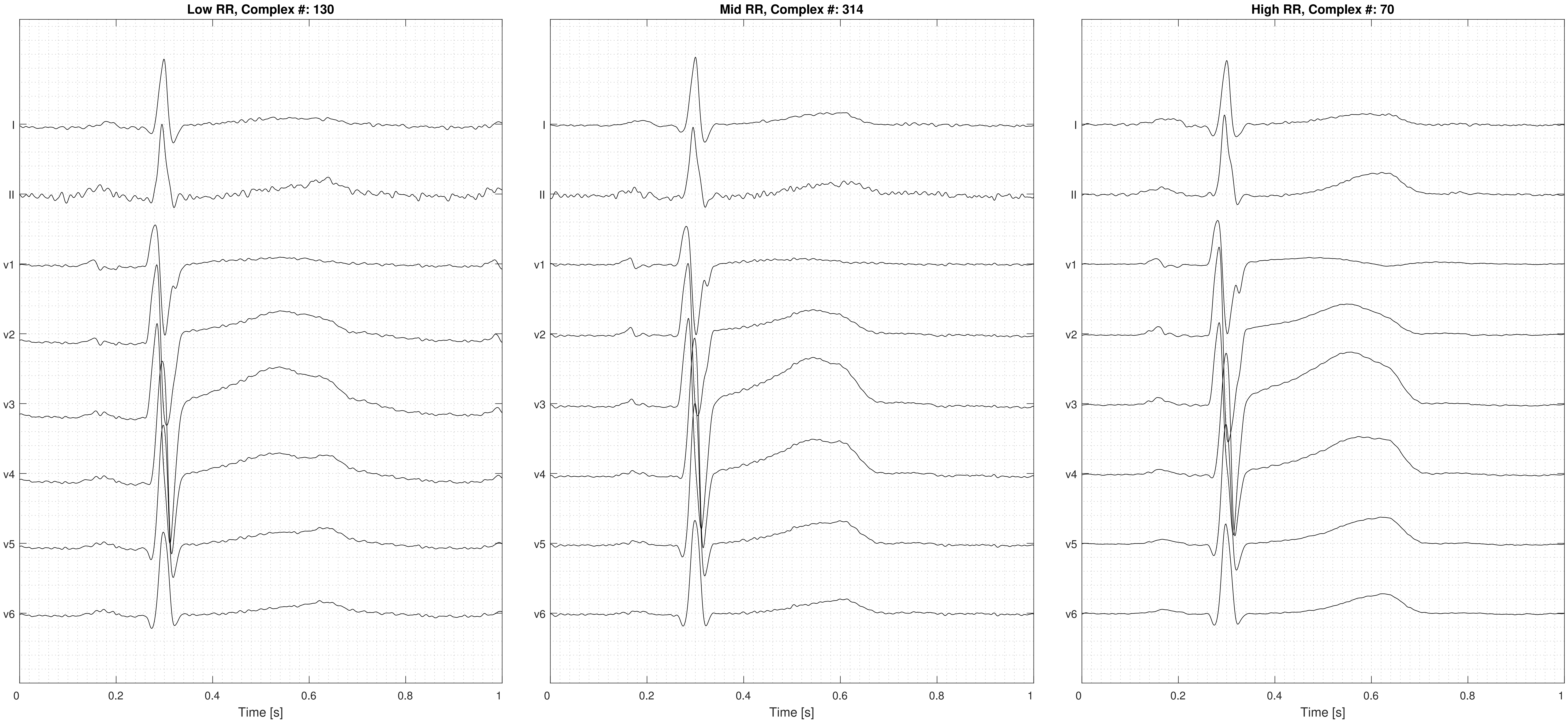
Low RR, Complex #: 130
Mid RR, Complex #: 314
High RR, Complex #: 70
I
II
v1
v2
v3
v4
v5
v6
0
0.2
0.4
0.6
0.8
1
Time [s]

Patient 123

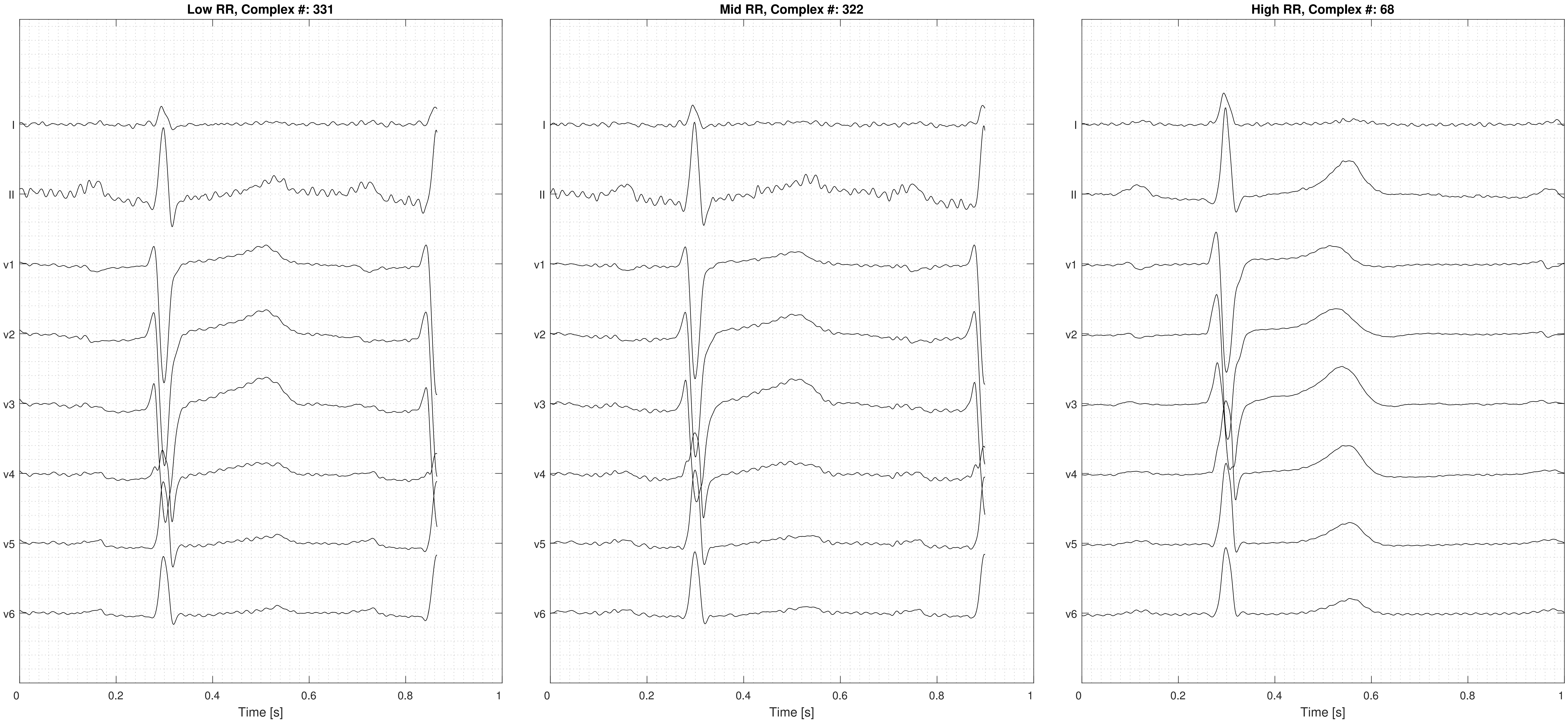

Low RR, Complex #: 331
Mid RR, Complex #: 322
High RR, Complex #: 68
I
II
v1
v2
v3
v4
v5
v6
0
0.2
0.4
0.6
0.8
1
Time [s]

Patient 124

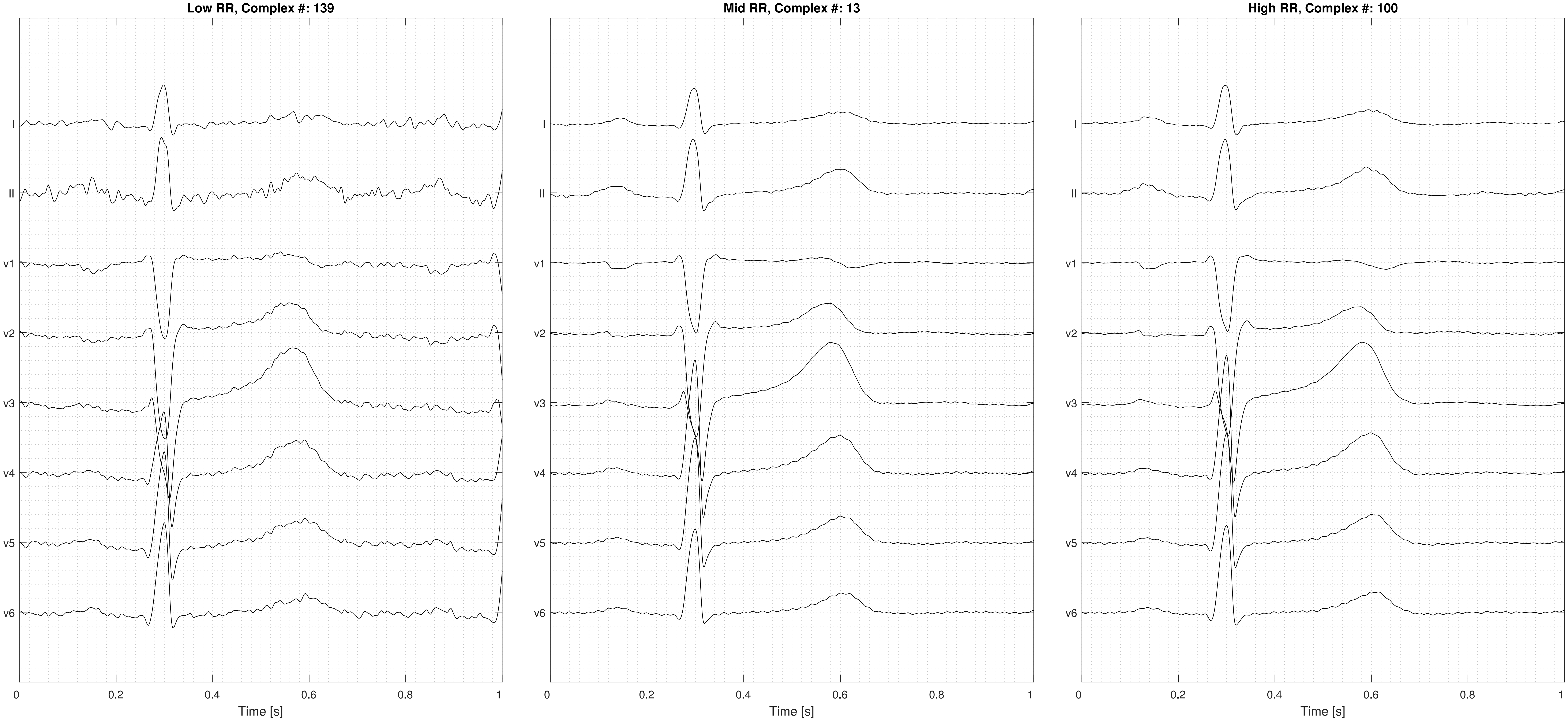
Low RR, Complex #: 139
Mid RR, Complex #: 13
High RR, Complex #: 100
I
II
v1
v2
v3
v4
v5
v6
0
0.2
0.4
0.6
0.8
1
Time [s]

Patient 125

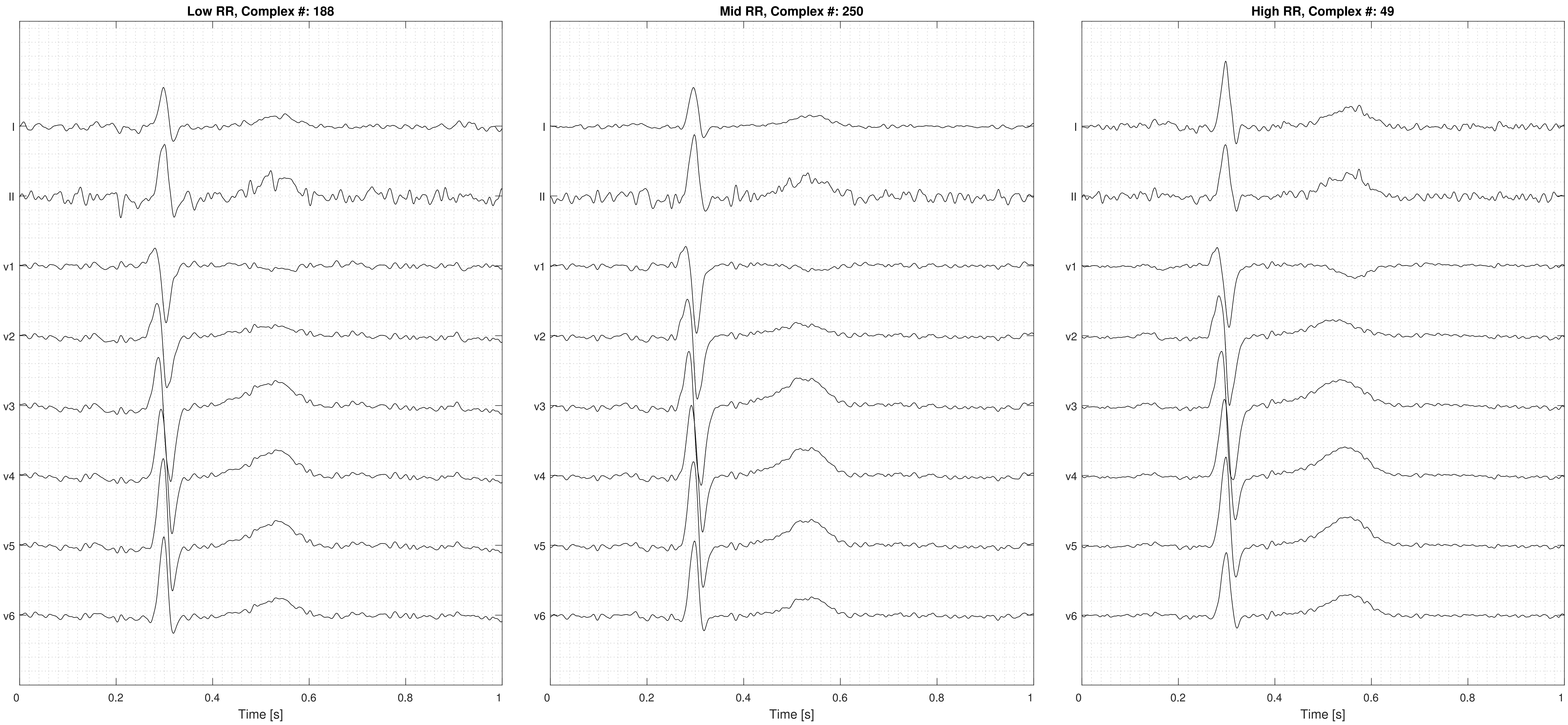

Low RR, Complex #: 188
Mid RR, Complex #: 250
High RR, Complex #: 49
I
II
v1
v2
v3
v4
v5
v6
0
0.2
0.4
0.6
0.8
1
Time [s]

Patient 126

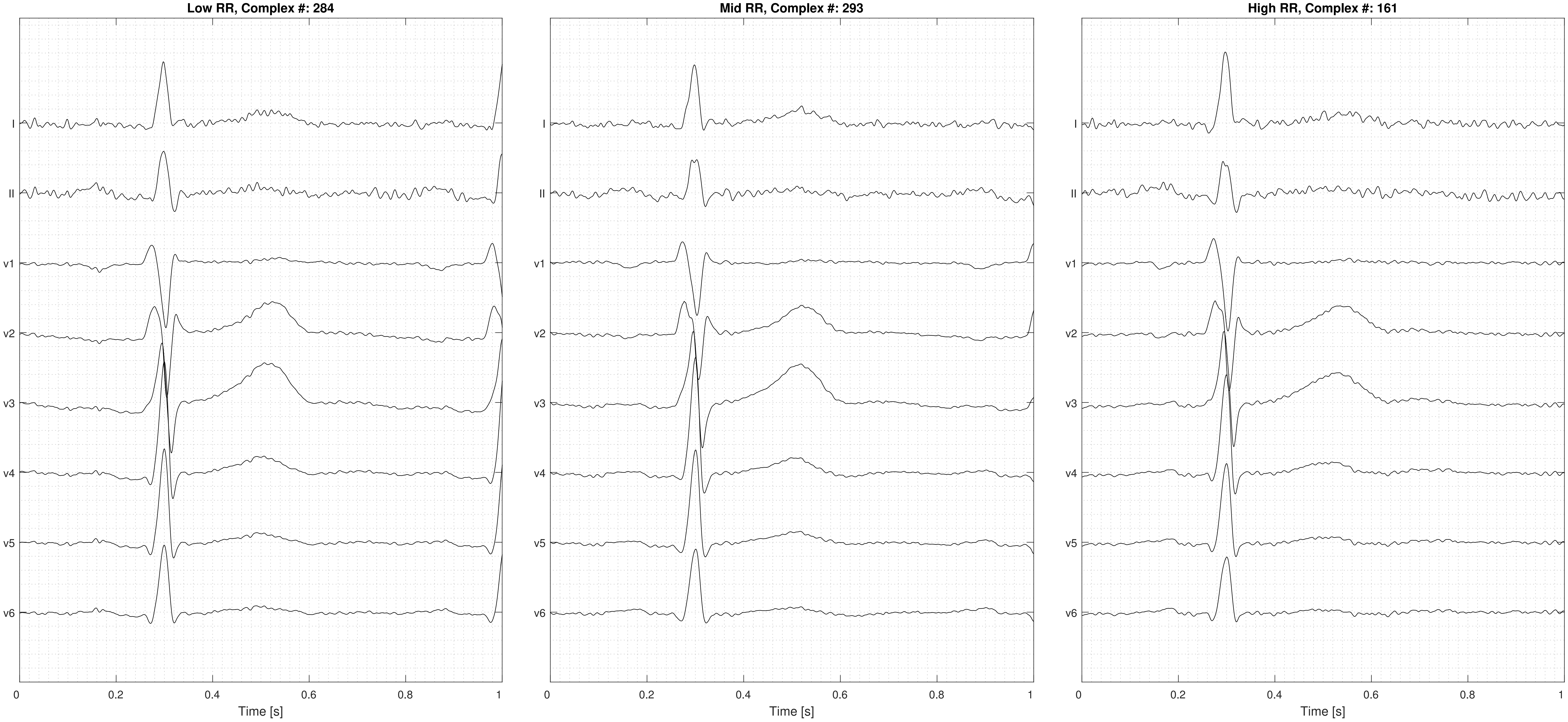

Low RR, Complex #: 284
Mid RR, Complex #: 293
High RR, Complex #: 161
I
II
v1
v2
v3
v4
v5
v6
0
0.2
0.4
0.6
0.8
1
Time [s]

Patient 127

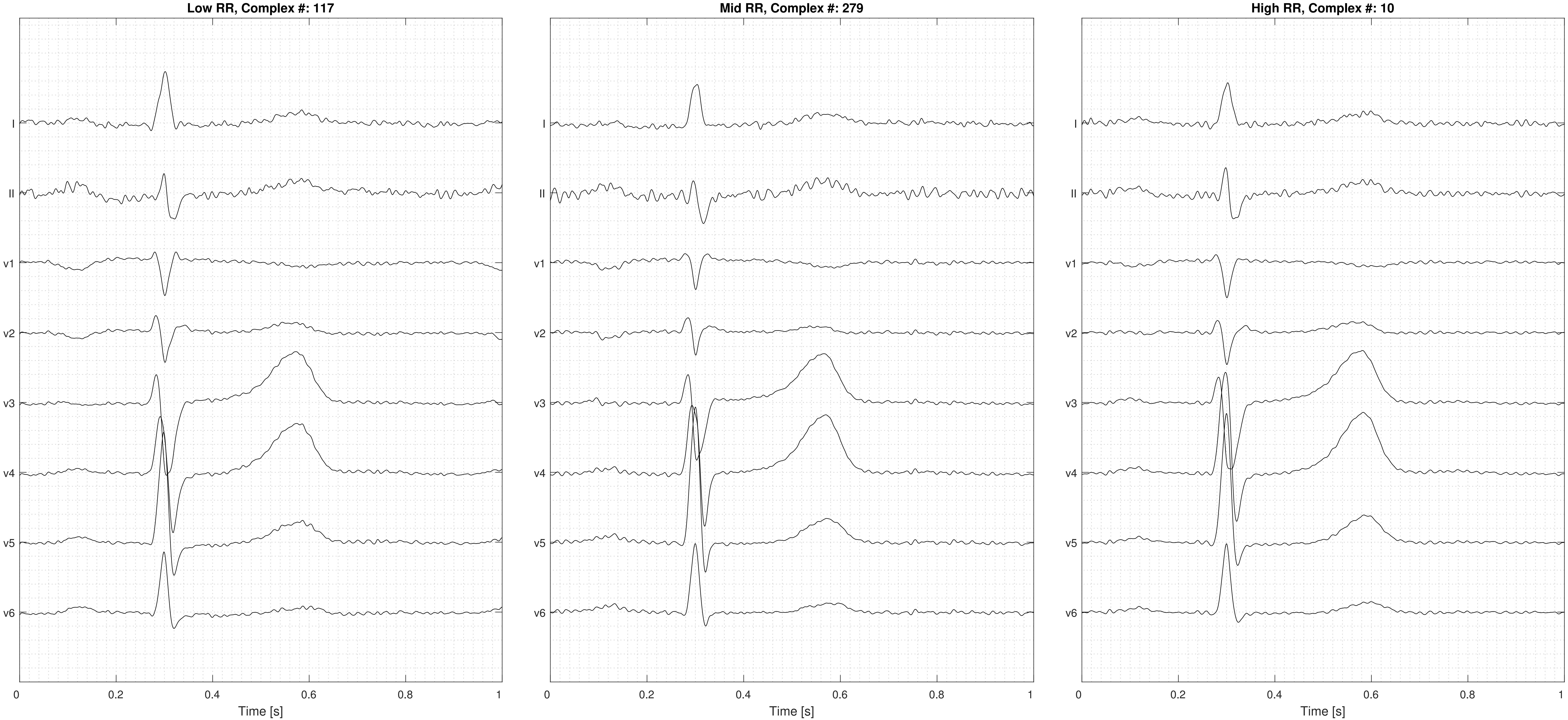

Low RR, Complex #: 117
Mid RR, Complex #: 279
High RR, Complex #: 10
I
II
v1
v2
v3
v4
v5
v6
0
0.2
0.4
0.6
0.8
1
Time [s]
